# Alleviation of Dyslipidemia via a Traditional Balanced Korean Diet Represented by a Low Glycemic and Low Cholesterol Diet in Obese Women in a Randomized Controlled Trial

**DOI:** 10.3390/nu14020235

**Published:** 2022-01-06

**Authors:** Min Jung Kim, Sunmin Park, Hye Jeong Yang, Phil-Kyung Shin, Haeng Jeon Hur, Seon-Joo Park, Kyun-Hee Lee, Moonju Hong, Jin Hee Kim, Sang-Woon Choi, Hae-Jeung Lee, Myung-Sunny Kim

**Affiliations:** 1 Research Group of Personalized Diet, Korea Food Research Institute, Wanju 55365, Korea; kmj@kfri.re.kr (M.J.K.); yhj@kfri.re.kr (H.J.Y.); mistltoe@kfri.re.kr (H.J.H.); kyunhee1234@gmail.com (K.-H.L.); lvnutrition1702@gmail.com (M.H.); kjh@kfri.re.kr (J.H.K.); 2 Department of Food and Nutrition, Obesity/Diabetes Research Center, Hoseo University, Asan 31499, Korea; smpark@hoseo.edu; 3 CHA Bio Complex, CHA University, Seongnam 13488, Korea; pkshin34@gmail.com; 4 Department of Food and Nutrition, College of BioNano Technology, Gachon University, Seongnam 13120, Korea; chris0825@hanmail.net; 5 Department of Food Biotechnology, Korea University of Science & Technology, Wanju 55365, Korea; 6 Chaum Life Center, CHA University, Seoul 06062, Korea; sangwoon.choi@gmail.com; 7 Department of Nutrition, School of Public Health and Health Sciences, University of Massachusetts Amherst, Amherst, MA 01003, USA

**Keywords:** traditional balanced Korean diet, dyslipidemia, insulin sensitivity, cholesterol, metabolites

## Abstract

A traditional balanced Korean diet (K-diet) may improve energy, glucose, and lipid metabolism. To evaluate this, we conducted a randomized crossover clinical trial, involving participants aged 30–40 years, who were randomly assigned to two groups—a K-diet or westernized Korean control diet daily, with an estimated energy requirement (EER) of 1900 kcal. After a 4-week washout period, they switched the diet and followed it for 4 weeks. The carbohydrate, protein, and fat ratios based on energy intake were close to the target values for the K-diet (65:15:20) and control diet (60:15:25). The glycemic index of the control diet and the K-diet was 50.3 ± 3.6 and 68.1 ± 2.9, respectively, and daily cholesterol contents in the control diet and K-diet were 280 and 150 mg, respectively. Anthropometric and biochemical parameters involved in energy, glucose, and lipid metabolism were measured while plasma metabolites were determined using UPLC-QTOF-MS before and after the 4-week intervention. After the four-week intervention, both diets improved anthropometric and biochemical variables, but the K-diet significantly reduced them compared to the control diet. Serum total cholesterol, non-high-density lipoprotein cholesterol, and triglyceride concentrations were significantly lower in the K-diet group than in the control diet group. The waist circumference (*p* = 0.108) and insulin resistance index (QUICKI, *p* = 0.089) tended to be lower in the K-diet group than in the control diet group. Plasma metabolites indicated that participants in the K-diet group tended to reduce insulin resistance compared to those in the control diet group. Amino acids, especially branched-chain amino acids, tyrosine, tryptophan, and glutamate, and L-homocysteine concentrations were considerably lower in the K-diet group than in the control diet group (*p* < 0.05). Plasma glutathione concentrations, an index of antioxidant status, and 3-hydroxybutyric acid concentrations, were higher in the K-diet group than in the control diet group. In conclusion, a K-diet with adequate calories to meet EER alleviated dyslipidemia by decreasing insulin resistance-related amino acids and increasing ketones in the circulation of obese women.

## 1. Introduction

Obesity is a growing epidemic worldwide. Approximately two-thirds of adults are overweight or obese in the USA; the prevalence of obesity is lower in Asians than in Caucasians [1]. However, a higher body fat percentage is exhibited by Asians than Caucasians with the same body mass index (BMI). Furthermore, Asians are more susceptible to developing metabolic diseases than Caucasians with the same BMI. Cutoffs of overweight and obesity are lower in Asians (23–25 and ≥25 kg/m^2^, respectively) than those in Caucasians (25–30 and ≥30 kg/m^2^, respectively) [2]. Obesity attenuates insulin signaling in various tissues to reduce energy, glucose, lipid metabolism, and immunity [3]. Therefore, obesity represents an underlying cause of various metabolic diseases, and weight loss may attenuate their symptoms.

Obesity is caused by an imbalance of energy intake and energy expenditure but does not correlate with a simple summation described in previous studies [4,5]. An unhealthy diet associated with low Korean health eating index (KHEI) scores increases obesity, especially abdominal obesity, and metabolic syndrome risk compared to that associated with a healthy diet, as reported in the Korea National Health and Nutrition Examination Survey (KNHANES) 2013–2017 and the Third National Health and Nutrition Examination Survey. However, the calorie intake is low or comparable between healthy and unhealthy diets [4,5]. These results suggest that an insufficient nutrient intake is potentially involved in reducing energy expenditure. Therefore, a healthy diet is essential for maintaining body fat within a normal range and optimal metabolism.

Different dietary trends are observed in each country. A Mediterranean diet was shown to reduce the rates and mortality of cardiovascular diseases in the 1950s [6]. This diet consists of a high intake of fruits and vegetables, olive oils, and cereals, a moderate intake of poultry, fish, and dairy products, and a low intake of red meat [7]. The Mediterranean diet has been reported to have long-term health benefits in metabolism and weight loss [7]. During the Korean War, Korean soldiers were revealed to have a considerably lower plaque deposition in the arteries than American soldiers; a rice-based diet may effectively reduce the plaque content [8]. Koreans traditionally consumed whole grains instead of refined rice, but they have switched to consuming refined rice since the 1980s. A rice-based diet has been found to be positively related to the development of metabolic diseases based on the Korean National Health and Nutrition Examination Survey and the Korean Genome and Epidemiology Study (KoGES) conducted in 1998–2014, since refined rice has a high glycemic index (GI) and contains insufficient nutrients despite low energy intake [9,10].

The dietary trend has shifted from the intake of a traditional balanced Korean diet (K-diet), a low-GI diet with low cholesterol, to the intake of a Westernized control diet (control diet), which is a high-GI diet with high cholesterol, due to economic growth [9]. Dietary patterns influence the gut microbiome of individuals [9]. Adults who consume a high proportion of K-diet have lower waist circumferences and risk of developing metabolic syndromes than those who consume a low proportion of K-diet [11,12]. According to a principal component analysis of the diet of Korean adults, the K-diet includes beans, potatoes, Kimchi, vegetables, fish, seafood, seaweeds, fruits, and fermented foods [12,13]. In contrast, a control diet contains high proportions of meat, noodles, bread, and fast foods [12,13]. Individuals who consume a high portion of K-diet have a higher KHEI than those who consume a low proportion. In contrast, individuals who consume a control diet have a relatively higher risk of developing metabolic syndromes than those who consume a K-diet, suggesting that the K-diet is healthier than the control diet.

Our previous two-week randomized clinical trial has shown that a K-diet (*n* = 5) reduces the inflammatory status in obese women aged 50–60 years by reducing levels of proinflammatory markers, lowering serum total cholesterol concentrations, and regulating the expression of blood-derived microRNAs linked to diabetes compared to those in individuals that consume control diet (*n* = 5) [14,15]. These results suggest that the K-diet intake may be more beneficial for healthy metabolism than control diet intake. We scaled up the present study to test the hypothesis that the K-diet with adequate calories to meet estimated energy requirement (EER) can improve energy, glucose, and lipid metabolism to prevent metabolic diseases by modulating the metabolome in obese women. The hypothesis was tested in a randomized crossover clinical trial involving a four-week K-diet and control diet intake in 52 obese women aged 30–40 years. These findings elucidate the health benefits of the K-diet.

## 2. Materials and Methods

### 2.1. Participants

Obese (25–30 kg/m^2^) Korean women aged 30–50 years were recruited via local advertisements at the Korea Food Research Institute, Wanju, South Korea. Exclusion criteria during the initial screening included: metabolic diseases, including type 2 diabetes, dyslipidemia, hypertension, thyroid disorders, and gastrointestinal tract diseases, and individuals under treatment with corresponding medications, including antibiotics, oral contraceptives, and hormonal replacement therapy. Individuals who showed >10% weight loss and had participated in another clinical trial in the previous three months were also excluded. None of the participants was in a menopausal state. They freely consumed about 2200 kcal/day. The Institutional Review Board at CHA University, Seongnam, Republic of South Korea (1044308-201801-HR-033-04), approved the study protocol, and the study was conducted according to the Helsinki Declaration. All participants provided written informed consent. The study was registered at the Korean Clinical Trial Registry (trial number, KCT0005340).

### 2.2. Sample Size Calculation

An intervention study with diets of different compositions [16] was calculated by power calculations, using the results of the significantly changed inflammatory markers. At the significance level of α = 0.05 and power of 0.80, the standard deviation was 0.80, and a difference of 1.3 or more in the inflammatory index was clinically meaningful. The sample size was calculated as 24 people in each group for crossover design. Our previous studies [14,15,17] reported a low dropout rate; therefore, a dropout rate of 10% was applied. Twenty-seven participants were enrolled in each intervention group, and one participant dropped out of each group.

### 2.3. Study Design

**Figure 1:**
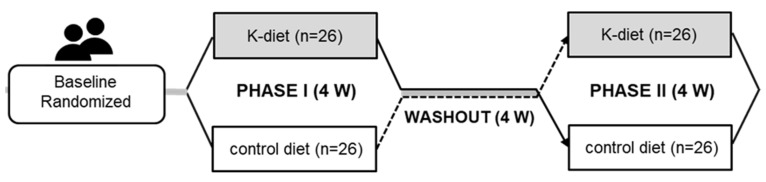
Scheme of the dietary intervention. 4 W, 4 weeks.

Participants voluntarily enrolled in the study from 3 January 2019, to 1 April 2020. A randomized, two-period crossover clinical trial was conducted with one washout period (Figure 1). The participants reported general information via interviews and diet intake via a 24-h dietary recall before the study began. The participants were randomly assigned to two groups using computer-generated random numbers, and the K-diet or control diet was randomly allocated for the first trial. After the first trial, a four-week washout period was performed, and participants were interviewed for diet intake via a 24-h dietary recall during the washout period. The diet was switched for the four-week second trial. The participants lived without restrictions, and meals, including beverages and snacks, were delivered to the house, as participants preferred each meal to be prepared by the food company. Compliance with meal consumption was assessed by submitting photographs before and after each meal to the researcher. The leftovers were estimated from the photographs and were reflected in the estimated food intake of the participants. The nutrient intake was calculated from the estimated food intake by subtracting the leftovers in the photos using a Computer-Aided Nutritional analysis program (CAN-pro 3.0; Korean Nutrition Society, Seoul, South Korea). Participants were encouraged to maintain their physical activity levels and restrict alcohol consumption. Energy and nutrient intake were estimated during the washout period via a 24-h dietary recall.

### 2.4. Diets

Each meal in the K-diet and control diet was prepared under the supervision of a dietician. Both diets were designed based on the Korean Food Guide of the Dietary Reference Intake for Koreans (KDRI). The K-diet was composed of cooked whole grains as a staple, soup, Kimchi, side dishes such as cooked vegetables, and seasoned fish or meat in each meal [17]. The participants in the K-diet group were provided one serving of grains, one serving of fish or poultry, two servings of cooked vegetables, one serving of Kimchi, and 1–2 servings of fermented soybeans in each meal. The dishes were prepared using Korean-style cooking methods, including fermenting, boiling, blanching, steaming, and pickling, and the seasonings comprised fermented soybeans, sesame oil, perilla oil, red pepper, and sesame. The control diet included refined rice instead of whole grains and more red meats, processed meats, bread, noodles, uncooked vegetables with salad dressing, and vegetable oil than in the K-diet. The control diet included 1 serving of refined rice, bread or noodles, soup, uncooked vegetables with salad dressing, 1 serving of meat, 0.5 servings of fermented soybeans, and 0.6 servings of Kimchi in each meal; the dishes were mainly cooked via baking, stir-frying, sauté, and frying methods. The sample menu used is shown in Supplemental Table S1. In both the K-diet and control diet groups, the calorie intake was estimated as 1900 kcal/day, based on the EER for Korean women aged 30–49 years. The K-diet featured 65% energy (En%) from carbohydrates, 15 En% from protein, and 20 En% from fat, whereas the control diet provided 60 En% from carbohydrates, 15 En% from protein, and 25 En% from fat. Sodium content in the diets was equivalent to 3.5–3.7 g/day.

GI and glycemic load (GL) of foods in the K-diet and control diet were extracted from the international tables for GI in 2021 [18]. Since they provided GI and GL for various foods from different countries, their values assessed in Asian countries were applied to calculate the GI and GL of the meals in the present study. GI and GL of the daily meal were calculated by the equation as follows: GI for daily intake = $\sum_{k=1}^{n}\left(\mathrm{each}\;\mathrm{food}\;\mathrm{of}\;\mathrm{GI}\;X\;\mathrm{carbohydrate}\;\mathrm{intake}\;\mathrm{from}\;\mathrm{the}\;\mathrm{food}\right)k\;$/ total carbohydrate intake for one day; GL for daily intake = $\sum_{k=1}^{n}\left(\mathrm{each}\;\mathrm{food}\;\mathrm{of}\;\mathrm{GI}\;X\;\mathrm{carbohydrate}\;\mathrm{intake}\;\mathrm{from}\;\mathrm{the}\;\mathrm{food}\right)k$ /100 [19]. K indicated the number of foods consumed for a day. Means and standard deviations were calculated for 28-day meals.

### 2.5. Anthropometric Parameters, Blood Pressure Measurements, and Blood Collection

Body weight and height were measured with the participants wearing a light gown. BMI was calculated by dividing the body weight value by the height value squared (kg/m^2^). Lean body mass and fat mass were measured using InBody 4.0 (Cheonan, Korea). Blood pressure was measured in the left arm using an automatic blood pressure monitor after the patients were seated and resting for 10 min. Serum total cholesterol, high-density lipoprotein (HDL), low-density lipoprotein (LDL), triglyceride, alanine aminotransferase (ALT), aspartate aminotransferase (AST), white blood cell (WBC), and platelet and plasma glucose levels were measured using a Hitachi 7600 AutoAnalyzer (Hitachi Ltd., Tokyo, Japan). Plasma insulin and serum high-sensitive C-reactive protein (CRP) concentrations were analyzed using an enzyme-linked immunosorbent assay kit (Crystal Chem, Elk Grove Village, IL, USA). Homeostatic model assessment (HOMA)-insulin resistance (IR), HOMA-B, and quantitative insulin sensitivity check index (QUICKI) were also calculated using plasma glucose and insulin concentrations in the fasting state.

### 2.6. Metabolomic Analysis Using Ultra-High-Performance Liquid Chromatography-Quadrupole Time-of-Flight Mass Spectrometry (UPLC-QTOF-MS)

Ice-cold methanol was added to plasma (2:1), and the mixture was vortexed for 1 min and sonicated for 15 min. The mixture was centrifuged at 13,000 rpm at 4 °C for 10 min, and the separated supernatants were dried entirely. These were then dissolved in 20% methanol and isolated after centrifugation. Plasma metabolites were measured via UPLC-QTOF-MS analysis. A quality control sample was prepared by mixing a small amount from each sample to verify the validity and reliability of the analysis. The supernatants were injected into the UPLC-QTOF-MS system (Synapt G2-Sil Waters, Milford, MA, USA) equipped with an ACQUTY^®^ BEH C18 column (2.1 × 100 mm, 1.7 µm) at 40 °C column temperature and 4 °C autosampler temperature. Elution was performed at a flow rate of 0.3 mL min^−1^ for 17 min using A (0.1% formic acid in water) and B (0.1% formic acid in acetonitrile) as gradients.

### 2.7. Statistical Analysis

A statistical analysis was conducted using SAS for Windows version 6.9.4 (SAS Institute, Cary, NC, USA). Results are presented as numbers and percentages for categorical variables and as mean ± standard deviations for continuous variables. The normal distribution of each continuous variable was confirmed in pooled data by Proc Univariate. Baseline data were analyzed before each intervention using a two-sample *t*-test for continuous variables and a chi-squared test for categorical variables. The changes of various variables before and after each intervention were analyzed by paired *t*-test. After the four-week intervention between the four-week washout periods, the results were analyzed via Proc mixed with adjustment for age, since this study was a crossover design. Statistical significance was set at *p* < 0.05.

## 3. Results

### 3.1. General Characteristics of the Participants

Before the intervention, the age, BMI, waist circumference, lean body mass, and fat mass did not differ significantly between the K-diet and control diet groups (*p* > 0.05; Table 1). Systolic and diastolic blood pressures (SBP and DBP, respectively) did not differ significantly between the two groups. Biochemical parameters including glucose, insulin, lipid profiles, AST, ALT, WBC count, CRP level, and platelet count in the blood did not differ between the two groups. HOMA-IR and HOMA-B, which indicate insulin resistance and capacity, were similar between individuals in the control diet and K-diet groups (Table 1).

### 3.2. Food and Nutrient Consumption

Whole and refined grains, vegetables, fruits, Kimchi, fermented soybeans, soybeans including tofu, fish, seafood, meats, seaweeds, nuts, and perilla oil intake were significantly different between the control diet and K-diet groups (Table 2). The intake of meats and refined grains was higher in the control diet group than in the K-diet group, whereas the intake of the other foods was higher in the K-diet group than in the control diet group (Table 2). K-diet belonged to low GI, and the control diet was moderate GI (Table 2). GL was also lower in K-diet than control diet (Table 2).

Nutrient intake was calculated from the daily food intake in the four-week intervention period, and the average daily nutrient intake is shown in Table 3. The daily energy intake was approximately 1850 kcal/d, similar to the EER of the participants, and there was no significant difference between the control diet and K-diet groups (*p* = 0.139). Regarding nutrient intake, polyunsaturated fatty acid (PUFA) intake did not differ significantly, whereas the intake of other nutrients differed significantly between the control diet and K-diet groups. Fat and protein levels in plant-based foods were higher in the K-diet than in the control diet (Table 3). As a result, cholesterol intake was considerably lower in the K-diet group (146 ± 1.3 mg/day) than in the control diet group (272 ± 1.1 mg/day). The carbohydrate, protein, and fat ratio based on energy intake was close to the target values 65:15:20 for the K-diet and 60:15:25 for the control diet (Table 3). Although protein intake was slightly lower than the target in both groups, the protein intake in both groups was higher than the recommended intake specified in the KDRI. Dietary fiber intake was considerably higher in the K-diet group than in the control diet group; however, the intake in the control diet group was higher than the adequate intake described in the KDRI. Sodium intake was approximately 3600 mg/day in both groups, similar to the typical Na intake of Koreans, and was considerably higher than the adequate intake specified in the KDRI. Potassium intake was higher in the K-diet group than in the control diet group, and it did not meet the adequate intake of KDRI (3500 mg/day). The sodium to potassium ratio was approximately 1:1 in the K-diet group and 1.4:1 in the control diet group (Table 3). Calcium intake was considerably lower than the recommended intake in both diets, similar to Korean intake. The K-diet did not include milk and milk products, and the control diet contained only milk with a sandwich for lunch. Both groups consumed higher amounts of vitamin A, thiamine, riboflavin, vitamin C, and folate than the recommended intake specified in the KDRI (Table 3).

### 3.3. Anthropometric and Biochemical Measurements at the End of the Intervention

BMI and waist circumference significantly decreased in K-diet and control diet, but the decrease tended to be greater in the K-diet than the control diet. BMI (*p* = 0.116) and waist circumference (*p* = 0.108) tended to be lower in the K-diet group than those in the control diet group. Fat mass, but not muscle mass, significantly decreased after K-diet and control diet interventions, and the muscle and fat mass did not differ between the two groups (Table 4). Systolic blood pressure, white blood cells, and platelets decreased after K-diet intervention but not control diet. However, blood pressure, WBC counts, and serum CRP concentrations were not significantly different between the groups after intervention. Lipid profiles were significantly decreased in both diet interventions, but serum triglyceride concentrations were reduced in the K-diet only (Table 4). The decrement was more substantial in the K-diet than the control diet: serum total cholesterol, non-HDL cholesterol, and triglyceride concentrations were considerably lower in the K-diet group than those in the control diet group, whereas serum HDL cholesterol concentrations were not significantly different between the K-diet and control diet groups (*p* = 0.069; Table 4). Serum glucose and insulin concentrations in the fasting state decreased in both diet interventions, but they were similar between the groups after intervention. Although HOMA-IR was reduced after both interventions, HOMA-IR and QUICKI did not differ significantly between the K-diet and control diet groups. However, QUICKI was slightly higher in the K-diet group than in the control diet group (*p* = 0.089). It indicated that the K-diet intake tended to lower insulin resistance than the control diet. HOMA-B (an insulin secretion index) decreased in both diet interventions, but it was not significantly different between the two groups (Table 4).

### 3.4. Analysis of Serum Metabolites via UPLC-QTOF-MS

Serum concentrations of most amino acids, except valine, significantly decreased in the K-diet group, but those of leucine and isoleucine were reduced in the control diet after a 4-week intervention (Table 5). Serum amino acid concentrations except glutamine arginine were significantly lower in the K-diet than in the control diet (Table 5). The serum concentration of L-homocysteine, a methionine derivative and insulin resistance index, decreased in both diets, but the decrement was much higher in the K-diet than in the control diet. Serum creatine and glutathione concentrations decreased only in K-diet after the intervention (Table 5). Serum glutathione concentration, an antioxidant index, was higher in the K-diet group than in the control diet group (Table 5). Serum concentrations of uric acid and uridine, nucleic acid derivatives, were lower in the K-diet group than in the control diet group. Notably, the serum concentration of the ketone 3-hydroxybutyric acid increased only in the K-diet after the intervention, and it was higher in the K-diet group than in the control diet group. Serum concentrations of isocitric acid were elevated in both diet interventions, but there was no difference between the two groups (Table 5).

## 4. Discussion

The incidence of obesity has markedly increased in adults until 2010; the increase in incidence remains relatively low in Korea [20]. Since obesity rates are closely related to the incidence of metabolic syndrome and cardiovascular diseases, the prevention and treatment of these disorders are considered to ameliorate obesity. Lifestyle modification is associated with maintaining normal body weight. In dietary modulation, changes in energy, carbohydrate, and fat intake have been applied for facilitating weight loss; however, these regimes are difficult to maintain for long periods [21]. A specific dietary pattern has recently been found to assist the weight loss regime. For example, the Mediterranean diet is an optimal regime for long-term weight loss [22], although the daily energy intake in the Mediterranean diet may be higher than that in other diets. Consumption of a traditional balanced Korean diet is inversely associated with metabolic syndrome development, including waist circumference, hypertension, and hypertriglyceridemia, as reported in the KNHANES, a Korean population study [23,24]. Therefore, in a randomized clinical trial, we aimed to determine whether a four-week K-diet intervention with proper calories meeting EER can promote energy, glucose, and lipid metabolism in obese women aged 30–50 years, compared to consumption of the control diet. When obese women consumed K-diet with adequate calories to satisfy EER, they improved dyslipidemia and promoted ketone production. The health benefit came from consuming more carbohydrates with a low GI and a lower cholesterol intake in the K-diet than in the control diet.

Obesity represents the primary cause of chronic metabolic diseases, and weight loss improves energy, glucose, and lipid metabolism, thereby reducing the risk of disease development. The K-diet and control diet contained the recommended carbohydrate, protein, and fat ratio reported in the KDRI, and the participants received the recommended EER (1900 kcal/day). The participants consumed about 2200 kcal/day prior to entering the intervention, and they had fewer calories during the intervention. This calorie restriction contributed to reducing body weight and metabolic improvement in both groups. However, K-diet showed better efficacy in reducing body fat and lipid metabolism than the control diet. The K-diet contained a large portion of carbohydrates (65 En%) with low GI and 20 En% fat with low saturated fatty acids (SFA, 2.5 En%) and low cholesterol (150 mg/day), whereas the control diet contained carbohydrates (60 En%) with high GI and 25 En% fat with SFA (6.5 En%) and high cholesterol (280 mg/day). The monounsaturated fatty acid (MUFA) content was higher in the K-diet than in the control diet, whereas both diets contained similar polyunsaturated fatty acid (PUFA) contents (approximately 5 En%). Both diets contained more than the recommended or adequate amount of all nutrients, except calcium and potassium. Both diets contained an identical amount of sodium (3600 mg/day); however, the potassium content was considerably lower in the control diet than in K-diet. The primary dietary differences between the K-diet and control diet were GI and cholesterol content, which may have shifted lipid profiles and insulin sensitivity toward improving the participants consuming the K-diet.

Dietary GI is a measure of the glycemic response after consumption of carbohydrates. A low-GI diet containing moderate fat content is known to be more effective for weight loss and glucose metabolism than a high GI and low-fat diet for overweight and obese adults in a six-month randomized, parallel, controlled clinical trial (GLYNDIET study) [25]. In a randomized five-week intervention, a low GI diet was found to decrease body weight considerably and serum concentrations of total and LDL cholesterol compared to those in a high GI diet group [26]. However, there was no significant increase in insulin sensitivity. In a meta-analysis of observational studies, low-GI diets for a short period (5–12 weeks) are reported to have an inverse relationship with serum total cholesterol and LDL concentration. However, there is no significant effect on serum HDL or triglyceride concentration [27]. In the present study, serum total cholesterol and non-HDL cholesterol concentrations were lower in the K-diet group than in the control diet group; however, weight loss and insulin sensitivity in the K-diet group were not significantly different from those in the control diet group.

Obesity can cause metabolic diseases by elevating insulin resistance. Insulin resistance is linked to attenuating insulin activity and increasing insulin secretion to normalize serum glucose concentrations. Obese Caucasian individuals, but not Asians, exhibit hyperinsulinemia with normoglycemia; however, obese Asians quickly develop type 2 diabetes when insulin resistance increases. Insulin resistance is usually determined based on HOMA-IR and QUICKI, whereas insulin secretion is determined based on HOMA-B. HOMA-IR is considerably lower in Asians than Caucasians, whereas the β-cell response is higher in Caucasians than Asians [28]. Therefore, in long-term prospective cohort studies, decreased β-cell function is initially observed in Asians, whereas decreased insulin sensitivity is initially observed in Caucasians when switching from normoglycemia to type 2 diabetes [29]. The present study demonstrated that HOMA-IR for insulin resistance index did not show a significant difference between K-diet and control diet, whereas HOMA-B for insulin secretion tended to be significantly lower in the K-diet group than in the control diet group. These results indicated that the K-diet intake might take more time to reduce insulin resistance, although the insulin secretion capacity tended to decrease.

Insulin resistance is associated with the serum concentrations of amino acids, especially branched-chain amino acid (BCAA), glutamate, tyrosine, and aspartate [30,31]. However, the relation between serum concentrations of BCAA and insulin resistance is still controversial in healthy persons [32]. Serum L-homocysteine concentration represents insulin resistance in non-diabetic and diabetic individuals and increased vascular risk [33,34]. L-homocysteine abolishes glutathione content and it is restored by improving insulin-receptor signaling [35]. It is associated with L-homocysteine reduction by activating glutathione peroxidase activity [36]. Glutathione is also protected with antioxidants like vitamin C and vitamin E [37]. In a cross-sectional study, serum L-homocysteine, insulin, and c-peptide concentrations were inversely associated with whole-grain intake [34]. Consistent with our findings, whole-grain intake has been shown to improve lipid profiles, including serum total, LDL, and HDL cholesterol concentrations [38]. The lower serum L-homocysteine may be linked to a higher intake of folate involved in one-carbon metabolism in the K-diet compared to the control diet [39]. Furthermore, serum glutathione concentrations were much higher in the K-diet than control diet, reducing oxidative stress. K-diet contained higher vitamin A and C than the control diet. Therefore, the improvement in insulin resistance might be linked to high folate and vitamin C intake in K-diet in the present study.

The association between cholesterol intake and dyslipidemia remains controversial. Serum concentrations of total and LDL cholesterol are considerably higher in individuals with dyslipidemia that have high cholesterol intake after adjustment for potential confounders; the replacement of carbohydrates with unsaturated fat has been shown to reduce serum LDL cholesterol concentrations in a nine-year follow-up of KoGES [40]. In a 12-week randomized clinical trial, a low-GI diet improved serum LDL, HDL, and triglyceride profiles in obese women [33]. The present study demonstrated that intake of a low cholesterol-containing K-diet (150 mg/day) decreased serum total and non-HDL cholesterol concentrations compared to that in individuals that consumed high cholesterol (300 mg/day)-containing control diet. The control diet had fewer carbohydrates and more fats (SFA: MUFA: PUFA = 1:1:1) than the K-diet. Therefore, high cholesterol intake may represent a primary factor in increasing serum total cholesterol and non-HDL cholesterol concentrations in the participants consuming the K-diet.

A low-carbohydrate diet, intermittent fasting, and prolonged fasting can increase ketone production to promote weight and fat loss as well as glucose and lipid metabolism in experimental animals and humans [41,42]. Ketone body and cholesterol synthesis are performed in the mitochondria and cytosol of the liver, respectively, from acetyl CoA. However, ketone bodies and cholesterol are synthesized in an opposite trend: the ketone body is produced in a fasting state, whereas cholesterol is produced in a fed state. The present study suggested that carbohydrates with a low GI promoted insulin sensitivity to inhibit cholesterol synthesis and enhance ketone production. Notably, even in a high-carbohydrate and low GI diet, consumption of the K-diet resulted in higher serum β-hydroxybutyrate concentrations than those obtained after consuming the control diet. Ketone production is inversely associated with serum triglyceride concentration and insulin resistance [43]. Therefore, a high-carbohydrate diet with a low GI can promote ketone production.

In conclusion, the K-diet, which contains high carbohydrates with low GI and low cholesterol, decreased serum total cholesterol and non-HDL cholesterol concentrations compared to after intake of the control diet in obese women. The K-diet, with adequate calories satisfying EER, decreased serum BCAA and L-homocysteine concentrations and increased serum β-hydroxybutyrate concentrations in obese women. Therefore, a K-diet with calories to meet EER alleviated dyslipidemia with increasing ketones and reduced insulin resistance-related amino acids in the circulation in obese women.

## Figures and Tables

**Table 1:** Baseline characteristics in control diet and K-diet groups.

Variable	Control Diet (*n* = 52)	K-Diet (*n* = 52)	*p*-Value ^2^
Age (years)	38.7 ± 5.62 ^1^	38.7 ± 5.55	1.0000
Weight (kg)	65.3 ± 6.39	65.5 ± 6.32	0.8825
Body mass index (kg/m^2^)	25.4 ± 1.78	25.5 ± 1.74	0.8333
Waist circumferences (cm)	80.3 ± 5.55	80.4 ± 5.62	0.8935
Lean body mass (kg)	22.0 ± 2.36	21.9 ± 2.24	0.7432
Fat mass (%)	38.2 ± 3.92	38.2 ± 4.32	0.9919
Systolic blood pressure (mmHg)	114 ± 11.4	117 ± 11.1	0.2070
Diastolic blood pressure (mmHg)	74.0 ± 7.93	73.7 ± 7.21	0.8091
White blood cell (×10^3^/uL)	5.66 ± 1.30	5.85 ± 1.44	0.4622
Platelet (×10^3^/uL)	25.4 ± 4.62	25.0 ± 4.83	0.7437
Serum triglyceride (mg/dL)	96.4 ± 53.9	100 ± 54.7	0.7172
Serum total cholesterol (mg/dL)	183 ± 31.9	186 ± 30.7	0.5955
Serum HDL-C (mg/dL)	61.1 ± 16.4	60.5 ± 14.0	0.8474
Serum LDL-C (mg/dL)	102 ± 28.5	105 ± 30.0	0.5975
Fasting plasma glucose (mg/dL)	97.8 ± 7.21	97.6 ± 6.99	0.8482
Fasting plasma Insulin (mU/L)	9.86 ± 3.61	10.4 ± 3.89	0.8075
HOMA-IR	2.40 ± 1.03	2.52 ± 1.01	0.5493
HOMA-B	104 ± 36.3	111 ± 37.7	0.3299
Serum CRP (mg/dL)	1.71 ± 1.89	1.94 ± 1.97	0.4871

^1^ Values presented means ± standard deviations. ^2^ Statistical analysis with a two-sample *t*-test. LDL-C, low-density lipoprotein cholesterol; HDL-C, high-density lipoprotein cholesterol; HOMA-IR, homeostasis model assessment of insulin resistance; HOMA-B, HOMA of beta-cell function; CRP, high-sensitive C-reactive protein.

**Table 2:** Food consumption during the intervention (unit: g/day).

Food Group	Control Diet (*n* = 52)	K-Diet (*n* = 52)	*p*-Value ^2^
Total grains	195 ± 8.61 ^1^	240 ± 18.5	<0.0001
Whole grains	1.7 ± 0.11	197 ± 16.2	<0.0001
Vegetables	278 ± 11.4	395 ± 20.6	<0.0001
Fruits	69.7 ± 1.73	75.4 ± 1.44	<0.0001
Kimchi	109 ± 12.8	171 ± 30.2	<0.0001
Fermented soybeans	12.0 ± 0.42	22.2 ± 1.19	<0.0001
Soybeans and tofu	16.8 ± 1.24	76.8 ± 4.20	<0.0001
Fishes and seafood	26.1 ± 1.47	57.8 ± 5.07	<0.0001
Meats	30.9 ± 1.18	12.9 ± 0.26	<0.0001
Seaweeds	4.8 ± 0.41	7.4 ± 0.58	<0.0001
Nuts	2.3 ± 0.01	7.4 ± 0.27	<0.0001
Perilla	0.3 ± 0.06	5.7 ± 0.47	<0.0001
Glycemic index of the meal	68.1 ± 2.89	50.3 ± 3.55	<0.0001
Glycemic load of the meal	192 ± 1.21	145 ± 1.53	<0.0001

^1^ Means ± standard deviations. ^2^ Statistical analysis with a two-sample *t*-test.

**Table 3:** Daily nutrient intake during the intervention.

Nutrient	Control Diet (*n* = 52)	K-Diet (*n* = 52)	*p*-Value ^2^
Energy (kcal)	1859 ± 60.5 ^1^	1834 ± 106	0.1385
Carbohydrates (En%)	62.9 ± 0.40	66.5 ± 0.50	<0.0001
Dietary fiber (g)	21.3 ± 0.83	36.7 ± 2.22	<0.0001
Protein (En%)	13.0 ± 0.10	14.0 ± 0.17	<0.0001
Plant protein (En%)	7.35 ± 0.07	10.3 ± 0.09	<0.0001
Animal protein (En%)	5.68 ± 0.09	3.69 ± 0.13	<0.0001
Fat (En%)	24.1 ± 0.33	19.5 ± 0.36	<0.0001
Saturated fatty acids (En%)	6.3 ± 0.48	2.45 ± 0	<0.0001
MUFA (En%)	5.3 ± 0.48	2.94 ± 0	<0.0001
PUFA (En%)	5.3 ± 0.48	4.91 ± 0.49	NS
Plant fat (En%)	14.5 ± 0.17	15.6 ± 0.27	<0.0001
Animal fat (En%)	9.54 ± 0.03	3.88 ± 0.02	<0.0001
Cholesterol (mg)	272 ± 7.84	146 ± 9.32	<0.0001
Calcium (mg)	490 ± 19.9	544 ± 38.4	<0.0001
Iron (mg)	17.3 ± 0.78	18.9 ± 1.36	<0.0001
Sodium (mg)	3632 ± 148	3573 ± 226	0.1136
Potassium (mg)	2531 ± 95.4	3432 ± 196	<0.0001
Vitamin A (μg RE)	497 ± 20.8	648 ± 39.7	<0.0001
Thiamin (mg)	1.69 ± 0.06	2.15 ± 0.12	<0.0001
Riboflavin (mg)	1.37 ± 0.05	1.28 ± 0.13	<0.0001
Niacin (mg)	14.6 ± 0.54	23.1 ± 1.59	<0.0001
Vitamin C (mg)	112 ± 4.08	124 ± 6.28	<0.0001
Folate (μg)	503 ± 21.9	670 ± 48.4	<0.0001

^1^ Values presented means ± standard deviations. ^2^ Statistical analysis with a two-sample *t*-test. En %, energy percent; MUFA, monounsaturated fatty acids; PUFA, polyunsaturated fatty acids; RE, retinol equivalent.

**Table 4:** Anthropometric and biochemical parameters in the control diet and K-diet groups after a 4-week intervention.

Variable	Control Diet (*n* = 52)	Changes ^1^ by Control Diet	K-Diet (*n* = 52)	Changes by K-Diet	*p*-Value ^2^
Body mass index (kg/m^2^)	25.1 ± 1.81 ^3^	−0.32 ± 0.43 *** ^4^	24.6 ± 0.25	−0.76 ± 0.58 *** ^5^	0.1158
Waist circumferences (cm)	79.1 ± 5.26	−1.08 ± 4.33	77.6 ± 0.73	−2.62 ± 4.69 ***	0.1084
Muscle mass (%)	33.7 ± 2.60	−0.11 ± 0.87	33.5 ± 0.38	−0.20 ± 0.79	0.7826
Fat mass (%)	37.4 ± 4.11	−0.88 ± 2.02 **	36.9 ± 0.56	−1.12 ± 1.73 **	0.4618
Systolic blood pressure (mmHg)	112 ± 10.5	−2.77 ± 12.8	113 ± 1.44	−4.50 ± 10.9 **	0.4738
Diastolic blood pressure (mmHg)	74.2 ± 6.63	−0.08 ± 8.43	73.8 ± 0.91	−0.12 ± 8.08	0.7679
White blood cells (×10^3^/uL)	5.3 ± 1.30	−0.38 ± 1.08	5.21 ± 0.18	−0.64 ± 1.23 ***	0.5827
Platelet (×10^3^/uL)	24.5 ± 4.83	−0.92 ± 3.10 *	24.0 ± 0.66	−1.08 ± 2.74 **	0.5244
Serum triglyceride (mg/dL)	93.2 ± 40.4	−6.58 ± 3.46	76.8 ± 5.6	−26.35 ± 43.6 ***	0.0177
Serum total cholesterol (mg/dL)	168 ± 27.9	−12.8 ± 20.0 ***	155 ± 3.84	−30.02 ± 19.1 ***	0.0067
Serum HDL-C (mg/dL)	54.8 ± 11.3	−5.98 ± 10.4 ***	51.3 ± 1.55	−8.92 ± 10.6 ***	0.0693
Serum LDL-C (mg/dL)	94.3 ± 24.8	−5.52 ± 16.6 *	88.3 ± 3.41	−15.88 ± 16.5 ***	0.1288
Serum non-HDL (mg/dL)	112 ± 26.0	−6.79 ± 17.5 **	104 ± 3.58	−21.10 ± 17.6 ***	0.0435
Fasting plasma glucose (mg/dL)	94.0 ± 6.92	−3.75 ± 7.43 ***	93.7 ± 0.96	−3.73 ± 6.63 ***	0.7593
Fasting plasma Insulin (mU/L)	7.96 ± 3.53	−2.09 ± 3.25 ***	7.24 ± 0.48	−3.20 ± 3.39 ***	0.2136
HOMA-IR	1.87 ± 0.94	−0.58 ± 0.87 ***	1.71 ± 0.13	−0.83 ± 0.87 ***	0.2576
HOMA-B	94.1 ± 35.1	−12.0 ± 36.5 *	84.3 ± 4.83	−28.0 ± 36.7 ***	0.0997
QUICKI	0.36 ± 0.02	0.02 ± 0.02	0.36 ± 0.00	0.03 ± 0.02	0.0885
Serum C-reactive protein (mg/dL)	1.29 ± 1.66	−0.52 ± 2.52	1.44 ± 0.22	−0.54 ± 6.71	0.5134

^1^ Changes of values from before and after intervention. ^2^ Statistical analysis using Proc mixed with sequence and treatment with adjusting for age using random sequencing. ^3^ Values presented means ± standard deviations. ^4^ Paired *t*-test before and after the intervention of control diet * at *p* < 0.05, ** at *p* < 0.01, and *** at *p* < 0.001. ^5^ Paired *t*-test before and after the intervention of K-diet * at *p* < 0.05, ** at *p* < 0.01, and *** at *p* < 0.001. HDL-C, high-density lipoprotein cholesterol, LDL-C, low-density lipoprotein cholesterol; non-HDL, total cholesterol minus HDL-C; HOMA-IR, homeostasis model assessment of insulin resistance; HOMA-B, HOMA of beta-cell function; QUICKI, quantitative insulin sensitivity check index; CRP, high-sensitive C-reactive protein.

**Table 5:** Serum metabolites in the control diet and K-diet groups after a 4-week intervention.

	Classification	Control Diet	Changes ^1^ by Control Diet	VIP ^2^	K-Diet	Changes ^1^ by K-Diet	VIP ^2^	*p*-Value ^3^
Amino acid	Valine	50.3 ± 4.01 ^4^	−9.7 ± 8.01	0.29	39.8 ± 4.00	−10.5 ± 11.8	0.37	0.0190
	Leucine	302 ± 8.52	−33.0 ± 84.7 *** ^5^	0.24	269 ± 8.47	−57.6 ± 51.1 *** ^6^	0.43	0.0049
	Isoleucine	12.3 ± 0.74	−3.7 ± 3.1 ***	0.12	14.2 ± 0.73	−2.6 ± 8.3 *	0.05	0.0747
	BCAA	363 ± 9.48	−27.1 ± 24.1 *	0.35	323 ± 9.34	−29.2 ± 33.2 ***	0.42	0.0040
	γ-glutamyl isoleucine	58.8 ± 4.73	−2.7 ± 48.5	0.03	44.4 ± 9.39	−35.9 ± 66.8 ***	0.39	0.0389
	Glutamate	15.3 ± 1.97	3.7 ± 19.3	0.06	8.15 ± 1.97	−8.4 ± 24.2 *	0.17	0.0187
	Glutamine	229 ± 5.63	12.1 ± 59.3	0.1	225 ± 5.60	16.7. ± 36.7 *	0.2	0.5433
	Arginine	467 ± 38.4	28.1 ± 36.3	0.09	385 ± 38.3	−74.9 ± 20.6 ***	0.69	0.1223
	Tyrosine	940 ± 23.3	−31.9 ± 10.1	0.14	845 ± 23.2	−40.6 ± 66.6 **	0.44	0.0047
	Tryptophan	8331 ± 169	−4.71 ± 47.8	0.01	7462 ± 168	−21.9 ± 30.2 ***	1.58	0.0003
	Glycine	21.8 ± 0.62	−0.5 ± 5.4	0.01	19.2 ± 0.62	−3.3 ± 4.8 ***	0.09	0.0051
	L-homocysteine	58.2 ± 11.5	−39.3 ± 69.4 ***	0.40	19.2 ± 11.4	−70.7 ± 85.0 ***	0.61	0.0168
	Creatine	2403 ± 119.7	71.7 ± 15.4	0.14	2169 ± 119	−112.0 ± 29.0 **	1.28	0.1043
	Glutathione	26.8 ± 3.77	−3.47 ± 40.5	0.04	41.8 ± 3.76	12.0 ± 33.9 *	0.16	0.0088
Carnitines	Carnitine	11,181 ± 584	383.0 ± 100.5 *	1.66	9593 ± 854	−258.1 ± 118	2.06	0.0663
	Acylcarnitine	38,910 ± 2621	933 ± 338 *	5.17	39,400 ± 2612	743 ± 416 *	3.33	0.8901
	Ratio of carnitine and acylcarnitine	0.309 ± 0.017	−0.08 ± 0.2 ***	0.10	0.287 ± 0.017	−0.10 ± 0.2 **	0.10	0.3048
Nucleosides	Uric acid	12,578 ± 338	185 ± 195	0.47	11,561 ± 336	−137 ± 127	0.73	0.0249
	Uridine	119 ± 3.41	−14.6 ± 37.7 **	0.16	107 ± 3.39	−26.2 ± 34.1 ***	0.33	0.0144
Organic acid	2-Ketobutyric acid	229 ± 11.39	44.0 ± 24.2 *	0.27	199 ± 11.41	−19.2 ± 28.6	0.26	0.0789
	Pyruvic acid	231 ± 5.55	−0.9 ± 10.5 *	0.0	217.8 ± 5.52	18.8 ± 37.8 **	0.17	0.068
	3-Hydroxybutyric acid	6.23 ± 1.93	3.12 ± 12.2	0.06	16.2 ± 1.92	12.2 ± 15.9 ***	0.17	0.0009
	Isocitric acid	6041 ± 154	84.5 ± 70.0 ***	0.01	5857 ± 153	51.2 ± 105 *	0.96	0.3296

^1^ Changes of values from before and after the intervention. ^2^ Variable importance in projection value. ^3^ Statistical analysis using Proc mixed with sequence and treatment with adjusting for age using random sequencing. ^4^ Values presented means ± standard deviations. ^5^ Paired *t*-test before and after the intervention of control diet * at *p* < 0.05, ** at *p* < 0.01, and *** at *p* < 0.001. ^6^ Paired *t*-test before and after the intervention of K-diet * at *p* < 0.05, ** at *p* < 0.01, and *** at *p* < 0.001. VIP, Variable importance in projection; BCAA, branched amino acids.

## Data Availability

All datasets used and analyzed during the current study are available from the corresponding author on reasonable request.

## Supplementary Materials

The following are available online at https://www.mdpi.com/article/10.3390/nu14020235/s1, Table S1: A one-day typical menu of control and K-diet.
