# Supplementary material for: Alleviation of Dyslipidemia via a Traditional Balanced Korean Diet Represented by a Low Glycemic and Low Cholesterol Diet in Obese Women in a Randomized Controlled Trial
Source: Nutrients. 2022 Jan 6;14(2):235. doi: 10.3390/nu14020235 (PMC8781638; doi:10.3390/nu14020235)
Supplement: Supplementary file 1 [file nutrients-14-00235-s001.zip › nutrients-1525701-supplementary.pdf]

Online Supplemental Material - Atkinson FS, Brand-Miller JC, Foster-Powell K, Buyken AE, Goletzke J. International tables of glycemic index and glycemic load values 2021.

Supplemental Table S1. Glycemic index (GI) values and glycemic load (GL) values determined in studies using method consistent with ISO 26642:2010.

Explanatory note: Values included in Supplemental Table S1 were tested using a methodology consistent with the ISO Standard (26642:2010). Test food portions had to contain either 25 or 50 g of available carbohydrate. Included data was tested in ≥10 healthy adults (allowing for one outlier to be excluded for GI determination) with reported normal glucose tolerance, aged 18 – 65 years. Blood sampling timepoints were those specified in the ISO Standard (0, 15, 30, 45, 60, 90 and 120 min), although additional time points were allowed (eg 75, 105, 150, and 180 min). Recommended analysis methods according to the ISO Standard (26642:2010) are spectrophotometry or electrochemical detection-coupled enzyme systems. If glucometers were used, in accordance with the ISO Standard only studies that used glucometers with a laboratory inter-assay CV on standard solutions <3.6% were included in Supplemental Table S1. Glycemic index categorization: low GI food ≤55, medium GI food 56 – 69, high GI food ≥70. Glycemic load (GL) is calculated by multiplying the known amount of available carbohydrate contained in the specified serving size by the GI value of that food (using glucose as the reference food), then dividing by 100. A standardized available carbohydrate portion for each food category was used to calculate the nominal GL value shown. The standardized available carbohydrate portion used for each food category is shown underneath the major food category headings. The GL value for each food is intended as a guide only.

TABLE OF CONTENTS FOR SUPPLEMENTAL TABLE S1

BAKERY PRODUCTS: pages 2- 6
BEVERAGES: pages 6 – 11
BREADS: pages 11 - 25
BREAKFAST CEREALS: pages 26 - 37
CEREAL GRAINS: pages 37 - 49
COOKIES: pages 50 - 57
CRACKERS: pages 58 - 60
DAIRY PRODUCTS AND ALTERNATIVES: pages 60 - 72
FRUIT AND FRUIT PRODUCTS: pages 73 - 83
INFANT FORMULA AND WEANING FOODS: pages 84 - 86
LEGUMES: pages 87 - 89
MEAL REPLACEMENT & WEIGHT MANAGEMENT PRODUCTS: pages 89 - 93
NUTRITIONAL SUPPORT PRODUCTS: pages 93 - 97
NUTS: page 97
PASTA AND NOODLES: pages 97 – 102
SNACK FOODS AND CONFECTIONERY: pages 102 - 115
SOUPS: pages 115 - 116
SUGARS AND SYRUPS: pages 116 - 119
VEGETABLES: pages 120 - 130
REGIONAL OR TRADITIONAL FOODS: pages 130 – 138
FOOTNOTES: page 139

Supplemental Table S1. Glycemic index (GI) values and glycemic load (GL) values determined in studies using method consistent with ISO 26642:2010.

Food Number and Item		Country of food production	Year of test[1]	GI[2] ± SEM (Glu = 100)	GL	Subjects (type & number)	Avail carb (Test portion)	Test portion[3] (g)	Reference food & time period	Timepoints	Sample collection	Sample analysis method[4]	Ref.
	**BAKERY PRODUCTS**												
	Average available carbohydrate portion = 30 g, this value was used to determine the nominal GL for each item in this category.												
	**Cakes**												
1	Cake, NS, decreased GI variant, sugar-to-flour ratio: 0.80 (Bakery School, Herk-de-Stad, Belgium)	Belgium	2010*	20±4	6	Normal, 10	25	54.4	Glucose, 2h	Standard	Venous, plasma/serum	Enzymatic	1
2	Carrot cake, prepared with wheat flour and coconut flour	Philippines	2002	37±2	11	Normal, 10	50	NS	Bread, 2h	Standard	Capillary, serum	Enzymatic	2
3	Chocolate cake made from packet mix with chocolate frosting (Betty Crocker, General Mills Inc., Minneapolis, USA)	USA	2000	38±3	11	Normal, 10	50	92.4	Glucose, 2h	Standard	Capillary, plasma	Enzymatic	UO[5]
4	Chocolate mudcake	Australia	2009	43±4	13	Normal, 9	50	109.4	Glucose, 2h	Standard	Capillary, plasma	Enzymatic	UO[5]
5	Christmas fruit cake	Australia	2009	53±3	16	Normal, 10	50	90.6	Glucose, 2h	Standard	Capillary, plasma	Enzymatic	UO[5]
6	Cupcake, strawberry-iced (Squiggles, Farmland, Grocery Holdings, Tooronga, Australia)	Australia	2000	73±12	22	Normal, 10	50	74.2	Glucose, 2h	Standard	Capillary, plasma	Enzymatic	UO[5]
7	Plumcake, prepared with wheat flour and yogurt (Mulino Bianco, Barilla, Italy)[6]	Italy	2005	47±6	14	Normal, 10	50	100	Glucose, 2h	Standard	Capillary, whole blood	YSI	3
8	Pound cake 0% (Bimbo S.A de C.V, Mexico)	Mexico	2006	38±5	11	Normal, 12	50	122.2	Glucose, 2h	Standard	Capillary, plasma	Enzymatic	UO[5]
9	Raspberry Coffee cake, President's Choice® Blue Menu™ (Loblaw Brands Limited, Canada)	Canada	2007	50±4	15	Normal, 10	50	113.6	Bread, 2h	Standard	Capillary, whole blood	YSI	UO[7]
10	Sponge cake, filled with marmalade and yogurt cream (Yogo brioss, Ferrero, Italy)	Italy	2014	57±5	17	Normal, 10	25	50	Glucose, 2h	Standard	Capillary, whole blood	YSI	3
11	Sponge cake with cocoa, milk cream-filled, coated with chocolate (Kinder Délice, Italy)[6]	Italy	2012	58±8	17	Normal, 10	50	114.8	Glucose, 2h	Standard	Capillary, whole blood	YSI	3
12	Sponge cake with cocoa, milk cream-filled, coated with chocolate and coconut pieces (Kinder Délice Cocco, Kinder, Ferrero, Italy)	Italy	2012	56±6	17	Normal, 10	25	62	Glucose, 2h	Standard	Capillary, whole blood	YSI	3
13	Sponge cake, soft wheat flour, carrots (14%), almonds (7%) (Camille, Mulino Bianco, Barilla, Italy)	Italy	2007	51±3	15	Normal, 10	50	70	Glucose, 2h	Standard	Capillary, whole blood	YSI	3

Food Number and Item		Country of food production	Year of test[1]	GI[2] ± SEM (Glu = 100)	GL	Subjects (type & number)	Avail carb (Test portion)	Test portion[3] (g)	Reference food & time period	Timepoints	Sample collection	Sample analysis method[4]	Ref.
14	Sponge cake, soft wheat flour, filled with chocolate cream (Kinder Pan e Cioc Cacao, Kinder, Ferrero, Italy)	Italy	2014	61±3	18	Normal, 10	25	56	Glucose, 2h	Standard	Capillary, whole blood	YSI	3
15	Sponge cake, soft wheat flour, ground cereals (rice, barley, rye, oat, wheat) and cocoa, filled with barley malt cream (Kinder colazione più, Kinder, Ferrero, Italy)	Italy	2014	66±8	20	Normal, 10	25	57	Glucose, 2h	Standard	Capillary, whole blood	YSI	3
16	Sponge cake, soft wheat flour, orange cream-filled, coated with chocolate (Fiesta, Ferrero, Italy)	Italy	2012	60±6	18	Normal, 10	25	48	Glucose, 2h	Standard	Capillary, whole blood	YSI	3
17	Sponge cake, soft wheat flour, with almond and chocolate pieces (Brioche Cereali e Cioccolato, Grancereale, Barilla, Italy)	Italy	2012	66±3	20	Normal, 10	50	107	Glucose, 2h	Standard	Capillary, whole blood	YSI	3
18	Sponge cake, wheat flour, rolled barley and oat (2.5%), filled with apricot jam (Brioss Albicocca ecereali, Ferrero Italy)[6]	Italy	2012	60±7	18	Normal, 10	50	80	Glucose, 2h	Standard	Capillary, whole blood	YSI	4
19	Vanilla cake, made from packet mix with vanilla frosting (Betty Crocker, General Mills Inc., Minneapolis, USA)	USA	2000	42±4	13	Normal, 10	50	96.8	Glucose, 2h	Standard	Capillary, plasma	Enzymatic	UO[5]
	**Cakes, gluten-free**												
20	Cake, prepared with yoghurt, gluten-free (Plum cake, Dr Shaer AG/SPA, Italy)[8]	Italy	2015*	42±6	13	Normal, 10	50	102	Glucose, 2h	Standard	Capillary, whole blood	YSI	5
21	Cake, prepared with brown rice, corn, millet and buckwheat, gluten-free (Gallette Chicchi al vento, Grancereale, Barilla, Italy)	Italy	2015*	67±6	20	Normal, 10	50	62	Glucose, 2h	Standard	Capillary, whole blood	YSI	5
	**Croissant**												
22	Croissant, all butter (Coles Supermarkets Pty Ltd, Australia)	Australia	2013	45±6	14	Normal, 10	50	123.2	Glucose, 2h	Standard	Capillary, plasma	Enzymatic	UO[5]
23	Croissant	Oman	2020*	67±2	20	Normal, 12	50	NS	Glucose, 2h	Standard	Capillary, whole blood	Glucometer[9]	6
	Croissant, mean of two foods			56									
	**Desserts**												
24	Apple Berry crumble, President's Choice® Blue Menu™ (Loblaw Brands Limited, Canada)	Canada	2007	41±3	12	Normal, 10	50	242.6	Bread, 2h	Standard	Capillary, whole blood	YSI	UO[7]

Food Number and Item		Country of food production	Year of test[1]	GI[2] ± SEM (Glu = 100)	GL	Subjects (type & number)	Avail carb (Test portion)	Test portion[3] (g)	Reference food & time period	Timepoints	Sample collection	Sample analysis method[4]	Ref.
25	Bavarian (mousse filling on biscuit base), Chocolate honeycomb, light (Sara Lee Bakery, Australia)	Australia	2006-2007	31±5	9	Normal, 9	50	184.5	Glucose, 2h	Standard	Capillary, whole blood	HemoCue	UO[10]
26	Danish Pastry, Apple & Peach, light (Sara Lee Bakery, Australia)	Australia	2006-2007	50±4	15	Normal, 10	50	114.4	Glucose, 2h	Standard	Capillary, whole blood	HemoCue	UO[10]
27	Fruit mince pies, shortcrust pastry case filled with dried fruit	Australia	2009	58±6	17	Normal, 11	50	80.9	Glucose, 2h	Standard	Capillary, plasma	Enzymatic	UO[5]
28	Pavlova, meringue base topped with whipped cream, fresh strawberries, banana and passionfruit pulp	Australia	2010	49±5	15	Normal, 9	50	193.3	Glucose, 2h	Standard	Capillary, plasma	Enzymatic	UO[5]
	**Gingerbread, cake-style**												
29	Gingerbread[11]	France	2019*	81±5	24	Normal, 15	50	68.2	Glucose, 2h	Standard	Capillary, plasma	Enzymatic	7
30	Gingerbread[11]	France	2019*	88±5	26	Normal, 15	50	68.2	Glucose, 2h	Standard	Capillary, whole blood	YSI	7
31	Gingerbread[11]	France	2019*	89±8	27	Normal, 13	50	68.2	Glucose, 2h	Standard	Capillary, plasma	Enzymatic	7
	Gingerbread, mean of three studies			86									
	**Muffins**												
32	Apple Blueberry muffin (Sara Lee Bakery, Australia)	Australia	20062007	49±4	15	Normal, 9	50	119.0	Glucose, 2h	Standard	Capillary, whole blood	HemoCue	UO[10]
33	Apple, oat, sultana muffin	Australia	2000	54±4	16	Normal, 9	50	92.6	Bread, 2h	Standard	Capillary, whole blood	HemoCue	UO[5]
34	Apricot, coconut and honey muffin	Australia	2000	60±4	18	Normal, 9	50	102.4	Bread, 2h	Standard	Capillary, whole blood	HemoCue	UO[5]
35	Banana, oat and honey muffin	Australia	2000	65±11	20	Normal, 10	50	98.3	Bread, 2h	Standard	Capillary, whole blood	HemoCue	UO[5]
36	Blueberry muffin (Sara Lee Bakery, Australia)	Australia	2006-2007	50±3	15	Normal, 10	50	96.9	Glucose, 2h	Standard	Capillary, whole blood	HemoCue	UO[10]
37	Blueberry (Wild) 10-Grain muffin, President's Choice® Blue Menu™ (Loblaw Brands Limited, Canada)	Canada	2007	57±7	17	Normal, 10	50	89.7	Bread, 2h	Standard	Capillary, whole blood	YSI	UO[7]

38	Chocolate butterscotch muffin	Australia	2001	53±5	16	Normal, 10	50	89.2	Bread, 2h	Standard	Capillary, whole blood	HemoCue	UO[5]
39	Chocolate chip muffin (Sara Lee Bakery, Australia)	Australia	2006-2007	52±6	16	Normal, 11	50	94.0	Glucose, 2h	Standard	Capillary, whole blood	HemoCue	UO[10]
40	Cranberry & Orange Soy muffin, President's Choice® Blue Menu™ (Loblaw Brands Limited, Canada)	Canada	2007	48±6	14	Normal, 10	50	120.7	Bread, 2h	Standard	Capillary, whole blood	YSI	UO[7]
41	Cranberry Raisin muffin	Australia	2013	43±3	13	Normal, 10	50	140.4	Glucose, 2h	Standard	Capillary, plasma	Enzymatic	UO[5]
42	Double chocolate muffin (Sara Lee Bakery, Australia)	Australia	2006-2007	46±4	14	Normal, 10	50	89.0	Glucose, 2h	Standard	Capillary, whole blood	HemoCue	UO[10]
43	Raisin Bran Flax muffin, President's Choice® Blue Menu™ (Loblaw Brands Limited, Canada)	Canada	2007	52±3	16	Normal, 10	50	106.1	Bread, 2h	Standard	Capillary, whole blood	YSI	UO[7]
44	Raspberry Pomegranate muffin, President's Choice® Blue Menu™ (Loblaw Brands Limited, Canada)	Canada	2007	58±7	17	Normal, 10	50	106.1	Bread, 2h	Standard	Capillary, whole blood	YSI	UO[7]
	Muffins, mean of 13 foods			53									

Food Number and Item		Country of food production	Year of test[1]	GI[2] ± SEM (Glu = 100)	GL	Subjects (type & number)	Avail carb (Test portion)	Test portion[3] (g)	Reference food & time period	Timepoints	Sample collection	Sample analysis method[4]	Ref.
	**Muffin, plain, made with different flours**												
45	Muffin, made with 14% soft wheat flour, 7% lentil flour, 5% resistant starch and 3% dextrins	Spain	2009	39±6	12	Normal, 10	50	133	Glucose, 2h	Standard	Venous, whole blood[12]	Enzymatic	8
46	Muffin made with oat flour	Singapore	2015*	54±5	16	Normal, 12	50	146.8	Glucose, 2h	Standard	Capillary, whole blood	HemoCue	9
47	Muffin made with barley flour	Singapore	2015*	55±5	17	Normal, 12	50	139.7	Glucose, 2h	Standard	Capillary, whole blood	HemoCue	9
48	Muffin made with corn flour	Singapore	2015*	74±5	22	Normal, 12	50	136.9	Glucose, 2h	Standard	Capillary, whole blood	HemoCue	9
49	Muffin made with wheat flour	Singapore	2015*	74±8	22	Normal, 12	50	126.1	Glucose, 2h	Standard	Capillary, whole blood	HemoCue	9
50	Muffin made with rice flour	Singapore	2015*	79±6	24	Normal, 12	50	119.4	Glucose, 2h	Standard	Capillary, whole blood	HemoCue	9

	**Pancakes**												
51	Pancakes, prepared with wheat flour and coconut flour	Philippines	2002	46±2	14	Normal, 10	50	NS	Bread, 2h	Standard	Capillary, serum	Enzymatic	2
52	Pancakes, prepared from wheat flour and water (Coles Smart Buy Plain Flour, Coles Supermarkets Pty Ltd) (78.9 g flour + 108.8 mL water)	Australia	2009	61±7	18	Normal, 10	50	78.9	Glucose, 2h	Standard	Capillary, plasma	Enzymatic	10
53	Pancakes, prepared from shake mix (Green's General Foods, Glendenning, Australia)	Australia	2000	67±5	20	Normal, 10	50	69.7	Glucose, 2h	Standard	Capillary, plasma	Enzymatic	UO[5]
54	Pancakes, millet (100% foxtail millet flour)	China	2016*	76±11	23	Normal, 10	50	121	Glucose, 2h	Standard	Venous, plasma/serum	Enzymatic	11
55	Pancakes, prepared from wheat flour	China	2005	80±4	24	Normal, 10	50	NS	Glucose, 2h	Standard	Venous, NS	NS	12
56	Pancakes, millet (75% foxtail millet flour, 25% extrusion flour (w/w, dry basis)	China	2016*	83±10	25	Normal, 10	50	141	Glucose, 2h	Standard	Venous, plasma/serum	Enzymatic	11
	**Pancakes, gluten-free**												
57	Pancakes, buckwheat, gluten-free, made from packet mix (Orgran Natural Foods, Carrum Downs, Australia)	Australia	2000	102±11	31	Normal, 10	50	69.4	Glucose, 2h	Standard	Capillary, plasma	Enzymatic	UO[5]
58	Pancakes, gluten-free, made from packet mix (Freedom Foods, Cheltenham, Australia)	Australia	2006-2007	61±6	18	Normal, 10	50	187.3	Glucose, 2h	Standard	Capillary, whole blood	HemoCue	UO[10]
	**Other bakery products**												
59	Brownies, containing wheat flour and coconut flour	Philippines	2002	43±4	13	Normal, 10	50	NS	Bread, 2h	Standard	Capillary, serum	Enzymatic	2

**Food Number and Item**		**Country of food production**	**Year of test[1]**	**GI[2] ± SEM (glu = 100)**	**GL**	**Subjects (type & number)**	**Avail carb (Test portion)**	**Test portion[3] (g)**	**Reference food & time period**	**Timepoints**	**Sample collection**	**Sample analysis method[4]**	**Ref.**
60	Cinnamon bread, prepared with wheat flour and coconut flour	Philippines	2002	45±3	14	Normal, 10	50	NS	Bread, 2h	Standard	Capillary, serum	Enzymatic	2
61	Doughnut	Oman	2020*	75±3	23	Normal, 12	50	NS	Glucose, 2h	Standard	Capillary, whole blood	Glucometer[9]	6
62	Hot cross buns, spiced sweet bun with dried fruit (Tip Top Bakeries, Chatswood, Australia)	Australia	2012	59±7	18	Normal, 10	50	89.8	Glucose, 2h	Standard	Capillary, plasma	Enzymatic	UO[5]
63	Hot cross buns, spiced sweet bun with dried fruit	Australia	2012	66±6	20	Normal, 9	50	112.8	Glucose, 2h	Standard	Capillary, plasma	Enzymatic	UO[5]

64	Pan de sal (sweet bread roll), containing wheat flour and coconut flour	Philippines	2002	62±4	19	Normal, 10	50	NS	Bread, 2h	Standard	Capillary, serum	Enzymatic	2
65	Pastry, puff (Pampas™, Australia)	Australia	2006	56±5	17	Normal, 10	50	127.2	Glucose, 2h	Standard	Capillary, plasma	Enzymatic	UO[5]
66	Pastry, puff, with wheat flour and sugar (Nastrine, Italy)[6]	Italy	2005	47±6	14	Normal, 9	50	102	Glucose, 2h	Standard	Capillary, whole blood	YSI	3
67	Pikelets, Golden brand (Tip Top Bakeries, Chatswood, Australia)	Australia	2000	85±14	26	Normal, 10	50	144.5	Glucose, 2h	Standard	Capillary, plasma	Enzymatic	UO[5]
68	Pizza base, baked in oven at 220°C for 9 min (Boboli, Orograin Bakeries Manufacturing Inc, PA, USA)	USA	2015	52±4	16	Normal, 10	50	104.2	Glucose, 2h	Standard	Capillary, plasma	Enzymatic	UO[5]
69	Scones, plain, made from packet mix (Defiance Milling Co, Australia)	Australia	2000	92±8	28	Normal, 10	50	105.3	Glucose, 2h	Standard	Capillary, plasma	Enzymatic	UO[5]
70	Sweet bread, with chocolate chips (Pangoccioli, Italy)[6]	Italy	2009	50±5	15	Normal, 10	50	81.4	Glucose, 2h	Standard	Capillary, whole blood	YSI	3
71	Tarte, made with wheat flour and apricot jam (Crostatina all'albicocca, Italy)[6]	Italy	2005	44±4	13	Normal, 9	50	77.5	Glucose, 2h	Standard	Capillary, whole blood	YSI	3
	**BEVERAGES**												
	Average available carbohydrate portion = 25 g, this value was used to determine the nominal GL for each item in this category, except for beer where 10 g was used.												
	**Beer**												
72	Beer (4.5% alcohol by volume) (Nikolai Lager, Sinebrychoff Ltd, Finland)	Finland	2012*	119±10	12	Normal, 10	25	510	Glucose, 2h	Standard	Capillary, whole blood	HemoCue	13
73	Beer (4.4% alcohol by volume), Pilsner Urquell® (Plzeňský Prazdroj, A.S., Czech Republic)[11]	Czech Republic	2016*	89±2	9	Normal, 10	25	554.3	Glucose, 2h	Standard	Capillary, plasma	Enzymatic	14
	Beer, mean of two foods			104									

Food Number and Item		Country of food production	Year of test[1]	GI[2] ± SEM (Glu = 100)	GL	Subjects (type & number)	Avail carb (Test portion)	Test portion[3] (g)	Reference food & time period	Timepoints	Sample collection	Sample analysis method[4]	Ref.
74	Beer, non-alcoholic (0.0% alcohol by volume) (Nikolai Lager,	Finland	2012*	80±6	8	Normal, 10	25	640	Glucose, 2h	Standard	Capillary, whole blood	HemoCue	13

	Sinebrychoff Ltd, Finland)												
	**Carbonated beverages/soft drinks**												
75	Coca Cola®, soft drink (Coca Cola Amatil, Sydney, Australia)	Australia	2006	53±7	13	Normal, 10	50	480.8	Bread, 2h	Standard	Capillary, plasma	Enzymatic	UO[5]
76	Coca Cola®, soft drink	Italy	2011	73±7	18	Normal, 9	50	472	Glucose, 2h	Standard	Capillary, whole blood	YSI	3
	Coca Cola®, mean of two studies			63									
77	Cola, Pepsi®, sucrose-sweetened (Pepsi, Hartwall Ltd, Finland)	Finland	2011	72±7	18	Normal, 12	50	450	Glucose, 2h	Standard	Capillary, whole blood	HemoCue	15
78	Ginger beer, carbonated beverage (Bundaberg Brewed Drinks Pty Ltd, Australia)	Australia	2017	65±5	16	Normal, 10	25	231.5	Glucose, 2h	Standard	Capillary, plasma	Enzymatic	UO[5]
79	Lemon flavored soft drink, Lemonade, Schweppes® (Cadbury Schweppes, Sydney, Australia)	Australia	2006	54±5	14	Normal, 10	50	454.5	Glucose, 2h	Standard	Capillary, plasma	Enzymatic	UO[5]
80	Lemon flavored soft drink, Solo™, lemon squash (Cadbury Schweppes, Australia)	Australia	2001	58±5	15	Normal, 10	25	190.8	Glucose, 2h	Standard	Capillary, whole blood	HemoCue	UO[5]
81	Lemon flavored soft drink Sprite™ (The Coca-Cola Company, Australia)	Australia	2011	64±3	16	Normal, 10	50	495.0	Glucose, 2h	Standard	Capillary, plasma	Enzymatic	16
	Lemon flavored soft drinks, mean of three foods			59									
	**Fruit drinks**												
82	Blackcurrant fruit syrup, Ribena™ reconstituted with 315.5 mL water (GlaxoSmithKline Group, Australia)	Australia	2006	52±5	13	Normal, 10	50	63.1	Glucose, 2h	Standard	Capillary, whole blood	HemoCue	UO[5]
83	Fruit drink, decreased GI variant (containing 8% isomaltose)	Belgium	2010*	48±8	12	Normal, 10	25	306	Glucose, 2h	Standard	Venous, plasma/serum	Enzymatic	1
84	Fruit drink, increased GI variant (containing 8% sucrose)	Belgium	2010*	86±14	22	Normal, 10	25	306	Glucose, 2h	Standard	Venous, plasma/serum	Enzymatic	1
85	Fruit flavored beverage	Australia	2014*	67±5	17	Normal 10	50	769	Glucose, 2h	Standard	Capillary, plasma	Enzymatic	17
86	Fruit flavored beverage with 0.22 g Filtered Molasses Concentrate added per 100 mL (3.6 g FMC/100 g carbohydrate)	Australia	2014*	58±3	15	Normal 10	50	820	Glucose, 2h	Standard	Capillary, plasma	Enzymatic	17

Food Number and Item	Country of food production	Year of test[1]	GI[2] ± SEM (Glu = 100)	GL	Subjects (type & number)	Avail carb (Test portion)	Test portion[3] (g)	Reference food & time period	Timepoints	Sample collection	Sample analysis method[4]	Ref.
87 Ice Green Tea, ready-to-drink brewed green tea (Pokka Corporation (S) Private Ltd, Singapore)	Singapore	2015*	50±5	13	Normal, 13	50	833.3	Glucose, 2h	Standard	Capillary, whole blood	HemoCue	18
88 Iced Green Tea, 7% lemon juice (Estathè verde, Ferrero, Italy)	Italy	2011	66±5	17	Normal, 9	50	575	Glucose, 2h	Standard	Capillary, whole blood	YSI	3

Food Number and Item	Country of food production	Year of test[1]	GI[2] ± SEM (Glu = 100)	GL	Subjects (type & number)	Avail carb (Test portion)	Test portion[3] (g)	Reference food & time period	Timepoints	Sample collection	Sample analysis method[4]	Ref.
89 Ice Lemon Tea, ready-to-drink black tea with lemon flavor (F&N Foods Private Ltd, Singapore)	Singapore	2015*	74±7	19	Normal, 14	50	500	Glucose, 2h	Standard	Capillary, whole blood	HemoCue	18
90 Iced lemon tea, decaffeinated, lemon juice powder 0.2% (7% fructose) (Estathè deteinato limone, Ferrero, Italy)	Italy	2011	38±3	10	Normal, 9	50	575	Glucose, 2h	Standard	Capillary, whole blood	YSI	3
91 Iced lemon tea, lemon juice powder 0.2%, (Estathè limone, Ferrero, Italy)	Italy	2011	72±6	18	Normal, 9	50	442	Glucose, 2h	Standard	Capillary, whole blood	YSI	3
92 Iced peach tea, 9.5% peach juice (Estathè pesca, Ferrero, Italy)	Italy	2011	71±7	18	Normal, 9	50	455	Glucose, 2h	Standard	Capillary, whole blood	YSI	3
93 Orange Delight Cocktail with pulp, President's Choice® Blue Menu™ (Loblaw Brands Limited, Canada)	Canada	2007	44±5	11	Normal, 10	50	781.3	Bread, 2h	Standard	Capillary, whole blood	YSI	UO[7]
94 Tropical blend fruit drink, V8 Splash® (Campbell's Soup Company, USA)	USA	2002	47±4	12	Normal, 10	50	455.4	Glucose, 2h	Standard	Capillary, whole blood	HemoCue	UO[5]
**Milk drinks**												
95 Choc Banana flavored drink, Aussie Bodies Start the Day UHT (Aussie Bodies Pty Ltd, Australia)	Australia	20062007	24±3	6	Normal, 10	50	847.5	Glucose, 2h	Standard	Capillary, whole blood	HemoCue	UO[10]
96 Chocolate flavored drink, Aussie Bodies Start the Day UHT (Aussie Bodies Pty Ltd, Australia)	Australia	2006-2007	26±4	7	Normal, 11	50	847.5	Glucose, 2h	Standard	Capillary, whole blood	HemoCue	UO[10]
97 Hot Chocolate mix made with hot water, Complete (Nestlé, Australia)	Australia	2001	51±3	13	Normal, 10	50	69.4	Glucose, 2h	Standard	Capillary, plasma	Enzymatic	UO[5]
98 Chocolate Liquid Breakfast, Devondale Fast Start (Murray Goulburn Co-Operative Company Limited, Australia)	Australia	2012	39±4	10	Normal, 9	50	468.7	Glucose, 2h	Standard	Capillary, plasma	Enzymatic	UO[5]

99	Coffee Liquid Breakfast, Devondale Fast Start (Murray Goulburn Co-Operative Company Limited, Australia)	Australia	2012	39±4	10	Normal, 9	50	449.2	Glucose, 2h	Standard	Capillary, plasma	Enzymatic	UO[5]
	Kellogg's® Coco Pops Chocolatey Liquid Breakfast 100 (Kellogg's Australia)	Australia	2012	35±4	9	Normal, 10	50	519.6	Glucose, 2h	Standard	Capillary, plasma	Enzymatic	UO[5]
101	Kellogg's® Nutri-Grain Breakfast Fuel (Kellogg's Australia)	Australia	2012	38±3	10	Normal, 10	50	514.6	Glucose, 2h	Standard	Capillary, plasma	Enzymatic	UO[5]
102	Milo™ (Nestlé, Australia) dissolved in water	Australia	1999	55±3	14	Normal, 10	50	73.0	Glucose, 2h	Standard	Capillary, plasma	Enzymatic	UO[5]
103	Milo™ (Nestlé, Auckland, New Zealand) dissolved in water	New Zealand	1999	52±5	13	Normal, 10	50	69.4	Glucose, 2h	Standard	Capillary, plasma	Enzymatic	UO[5]
104	Milk powder, Jerseyjoy Active Life (Australia Jersey Dairy Group Pty Ltd), prepared with water	Australia	2019	32±4	8	Normal, 10	50	96.2	Glucose, 2h	Standard	Capillary, plasma	Enzymatic	UO[5]
105	Nesquik™, chocolate (Nestlé, Australia), prepared with water	Australia	1998	53±5	13	Normal, 9	50	NS	Bread, 2h	Standard	Capillary, plasma	Enzymatic	UO[5]

Food Number and Item		Country of food production	Year of test[1]	GI[2] ± SEM (Glu = 100)	GL	Subjects (type & number)	Avail carb (Test portion)	Test portion[3] (g)	Reference food & time period	Timepoints	Sample collection	Sample analysis method[4]	Ref.
106	Nesquik™, strawberry (Nestlé, Australia), prepared with water	Australia	1998	64±6	16	Normal, 9	50	NS	Bread, 2h	Standard	Capillary, plasma	Enzymatic	UO[5]
107	Ovaltine®, chocolate (Twinings & Co, Australia), prepared with water	Australia	2016	50±4	13	Normal, 10	25	37.6	Glucose, 2h	Standard	Capillary, plasma	Enzymatic	UO[5]
108	Formulated Milk powder for Middle-Aged Adults, Probilove (Australia Jersey Dairy Group Pty Ltd), prepared with water	Australia	2019	32±4	8	Normal, 10	50	100.5	Glucose, 2h	Standard	Capillary, plasma	Enzymatic	UO[5]
109	Vanilla drink with fiber, Build-Up™ nutrient-fortified drink (Nestlé, Australia)	Australia	2001	41±4	10	Normal, 10	50	92.1	Glucose, 2h	Standard	Capillary, plasma	Enzymatic	UO[5]
110	Vanilla Liquid Breakfast, Devondale Fast Start (Murray Goulburn Co-Operative Company Limited, Australia)	Australia	2012	49±3	12	Normal, 10	50	456.8	Glucose, 2h	Standard	Capillary, plasma	Enzymatic	UO[5]
111	Yakult (Yakult Italia)	Italy	2011	51±4	13	Normal, 10	25	170	Glucose, 2h	Standard	Capillary, whole blood	YSI	3
112	Yakult, fermented milk drink (Yakult company, Taipei,	Taiwan	2010*	58±0.3	15	Normal, 10	50	NS	Bread, 2h	Standard	Capillary, plasma	Enzymatic	19

Food Number and Item		Country of food production	Year of test[1]	GI[2] ± SEM (Glu = 100)	GL	Subjects (type & number)	Avail carb (Test portion)	Test portion[3] (g)	Reference food & time period	Timepoints	Sample collection	Sample analysis method[4]	Ref.
	Taiwan)												
113	Yakult® Light, fermented milk drink with *Lactobacillus casei* (Yakult, Dandenong, Australia)	Australia	2005	36±6	9	Normal, 10	50	384.6	Glucose, 2h	Standard	Capillary, whole blood	HemoCue	UO[5]
	**Rice milk drink**												
114	Rice milk drink, low-fat, Australia's Own Natural™ (So Natural Foods, Taren Point, Australia)	Australia	2003	92±9	23	Normal, 10	50	390.6	Glucose, 2h	Standard	Capillary, whole blood	HemoCue	UO[5]
115	Rice milk, low-fat, calcium enriched, Vitasoy® (National Foods Ltd, Australia)	Australia	2005	79±8	20	Normal, 10	50	531.9	Glucose, 2h	Standard	Capillary, whole blood	HemoCue	UO[5]
	**Smoothies**												
116	Smoothie, apple, pear, banana (Frullato mela, pera e banana Storie di frutta, Mulino Bianco, Barilla, Italy)	Italy	2011	68±5	17	Normal, 9	50	336	Glucose, 2h	Standard	Capillary, whole blood	YSI	3
117	Smoothie, banana	Australia	2003	30±4	8	Normal, 10	50	505.1	Glucose, 2h	Standard	Capillary, whole blood	HemoCue	UO[5]
118	Smoothie, Banana & Honey, Devondale brand (Murray Goulburn Co-Operative Company Limited, Australia)	Australia	2014	28±3	7	Normal, 10	50	517.5	Glucose, 2h	Standard	Capillary, plasma	Enzymatic	UO[5]
119	Smoothie, banana and strawberry, V8 Splash® (Campbell's Soup Company, Camden, USA)	USA	2002	44±3	11	Normal, 10	50	473.0	Glucose, 2h	Standard	Capillary, whole blood	HemoCue	UO[5]
120	Smoothie, mango	Australia	2003	32±4	8	Normal, 10	50	476.2	Glucose, 2h	Standard	Capillary, whole blood	HemoCue	UO[5]
121	Smoothie, mango and passionfruit (Innocent Ltd., UK)	UK	2019*	36±5	9	Normal, 10	25	235.8	Glucose, 2h	Standard	Capillary, whole blood	HemoCue	20

Food Number and Item		Country of food production	Year of test[1]	GI[2] ± SEM (Glu = 100)	GL	Subjects (type & number)	Avail carb (Test portion)	Test portion[3] (g)	Reference food & time period	Timepoints	Sample collection	Sample analysis method[4]	Ref.
122	Smoothie, raspberry (Con Agra Inc, Omaha, USA)	USA	1999	33±9	8	Normal, 10	25	177.3	Bread, 2h	Standard	Capillary, plasma	Enzymatic	UO[5]
123	Smoothie, strawberry and banana (Innocent Ltd., UK)	UK	2019*	39±4	10	Normal, 10	25	213.7	Glucose, 2h	Standard	Capillary, whole blood	HemoCue	20
124	Smoothie, Tropical, Devondale brand (Murray Goulburn Co-Operative Company Limited, Australia)	Australia	2014	31±4	8	Normal, 10	50	588.1	Glucose, 2h	Standard	Capillary, plasma	Enzymatic	UO[5]

	Smoothie, mean of nine foods			38									
	**Soy drinks**												
125	Cocoa malt flavor, Up & Go™, soy milk, rice cereal liquid breakfast (Sanitarium Health Foods, Australia)	Australia	2001	43±5	11	Normal, 10	25	NS	Glucose, 2h	Standard	Capillary, plasma	Enzymatic	UO[5]
126	Chocolate Daydream™ shake, fructose (Revival Soy®, Physicians Pharmaceuticals, Inc, USA) (47.1 g powder + 305 mL water)	USA	2004	33±4	8	Normal, 10	25	47.1	Glucose, 2h	Standard	Capillary, whole blood	HemoCue	21
127	Chocolate flavored soy milk drink, So Good Bliss (Sanitarium Health Foods, Australia)	Australia	2008	46±3	12	Normal, 10	50	595.2	Glucose, 2h	Standard	Capillary, plasma	Enzymatic	UO[5]
128	Chocolate, Xpress, soy bean, cereal and legume extract drink with fructose (So Natural Foods, Australia)	Australia	2001	39±2	10	Normal, 10	25	438.6	Glucose, 2h	Standard	Capillary, whole blood	HemoCue	UO[5]
129	Original malt flavor, Up & Go™, soy milk, rice cereal liquid breakfast (Sanitarium Health Foods, Australia)	Australia	2001	46±5	12	Normal, 10	25	NS	Glucose, 2h	Standard	Capillary, plasma	Enzymatic	UO[5]
130	Smoothie drink, soy, banana, 1% fat (So Natural Foods, Australia)	Australia	2000	30±3	8	Normal, 10	25	342.5	Glucose, 2h	Standard	Capillary, plasma	Enzymatic	UO[5]
131	Smoothie drink, soy, chocolate hazelnut, 1% fat (So Natural Foods, Australia)	Australia	2000	34±3	9	Normal, 10	25	294.1	Glucose, 2h	Standard	Capillary, plasma	Enzymatic	UO[5]
132	Soy Beverage, Chocolate flavored, President's Choice® Blue Menu™ (Loblaw Brands Limited, Canada)	Canada	2004-2007	40±5	10	Normal, 10	50	446.4	Bread, 2h	Standard	Capillary, whole blood	YSI	UO[7]
133	Soy Beverage, Original flavored, President's Choice® Blue Menu™ (Loblaw Brands Limited, Canada)	Canada	2004-2007	15±4	4	Normal, 10	25	694.4	Bread, 2h	Standard	Capillary, whole blood	YSI	UO[7]
134	Soy Beverage, Vanilla flavored, President's Choice® Blue Menu™ (Loblaw Brands Limited, Canada)	Canada	2004-2007	28±3	7	Normal, 10	50	781.3	Bread, 2h	Standard	Capillary, whole blood	YSI	UO[7]
	**Sports drinks**												
135	Sport beverage based on whey protein powder with carbohydrate (CHO:PRO=4:1), orange flavor (Endurox R4, Pacific Health Laboratories, Parsippany, NJ, USA)	USA	2017*	67±6	17	Normal, 10	50	NS	Glucose, 2h	Standard	Capillary, whole blood	YSI	22
136	Sustagen® Sport Chocolate (Nestlé Health Science, Australia), prepared with water	Australia	2019	36±3	9	Normal, 10	50	76.6	Glucose, 2h	Standard	Capillary, plasma	Enzymatic	UO[5]

Food Number and Item		Country of food production	Year of test[1]	GI[2] ± SEM ([illegible]lu = 100)	GL	Subjects (type & number)	Avail carb (Test portion)	Test portion[3] (g)	Reference food & time period	Timepoints	Sample collection	Sample analysis method[4]	Ref.
137	Sustagen® Sport Vanilla (Nestlé Health Science, Australia), prepared with water	Australia	2019	37±3	9	Normal, 9	50	75.5	Glucose, 2h	Standard	Capillary, plasma	Enzymatic	UO[5]
138	Thorpedo® Advanced Hydration for Kids, all flavors (Thorpedo Foods, Australia)	Australia	2006-2007	11±2	3	Normal, 10	50	357.1	Glucose, 2h	Standard	Capillary, whole blood	HemoCue	UO[10]
139	Thorpedo® Ultra Low GI Energy Water, all flavors (Thorpedo Foods, Australia)	Australia	2006	16±4	4	Normal, 10	25	843.3	Glucose, 2h	Standard	Capillary, whole blood	HemoCue	UO[5]
	**Other beverages**												
140	Ancient Grains Quinoa milk drink with Chia (Freedom Foods, Australia)	Australia	2015	42±3	11	Normal, 10	50	561.1	Glucose, 2h	Standard	Capillary, plasma	Enzymatic	UO[5]
141	Coconut milk beverage, containing organic coconut milk and organic brown rice (Pure Harvest Coco Quench, Australia)	Australia	2020	68±6	17	Normal, 10	50	641.0	Glucose, 2h	Standard	Capillary, plasma	Enzymatic	UO[5]
142	Coconut Water, water of green coconut, Nudie™	Australia	2012	55±3	14	Normal, 9	25	500.0	Glucose, 2h	Standard	Capillary, plasma	Enzymatic	UO[5]
143	Goat Milk Drink, Symbiotics Low GI (NIG Nutritionals Pty Ltd, New Zealand), powder prepared with water	New Zealand	2019	25±2	6	Normal, 10	50	87.7	Glucose, 2h	Standard	Capillary, plasma	Enzymatic	UO[5]
144	Oat milk, calcium enriched, Vitasoy® (National Foods Ltd, Australia)	Australia	2009	69±6	17	Normal, 9	50	571.4	Glucose, 2h	Standard	Capillary, plasma	Enzymatic	UO[5]
	**BREADS**												
	Average available carbohydrate portion = 15 g, this value was used to determine the nominal GL for each item in this category.												
	**English Muffin**												
145	English Muffin™ bread (Natural Ovens, USA)	USA	1999	77±7	12	Normal, 10	50	141.6	Bread, 2h	Standard	Capillary, plasma	Enzymatic	UO[5]
146	English Muffin, Whole Grain Multigrain, President's Choice® Blue Menu™ (Loblaw Brands Limited, Canada)	Canada	2007	45±3	7	Normal, 10	50	142.5	Bread, 2h	Standard	Capillary, whole blood	YSI	UO[7]
	**Fruit breads**												
147	Fruit and Spice Bread, Vogel's (Goodman Fielder, New 13 Zealand)	New Zealand	2009*	57±5	9	Normal, 20	50	NS	Glucose, 2h	Standard	Capillary, whole blood	HemoCue	23
148	Fruit and Spice Bread, Vogel's (Goodman Fielder, Auckland, 13	New Zealand	2010*	68±4	10	Normal, 20	50	NS	Glucose, 2h	Standard	Capillary, whole blood	HemoCue	24

Food Number and Item		Country of food production	Year of test[1]	GI[2] ± SEM (Glu = 100)	GL	Subjects (type & number)	Avail carb (Test portion)	Test portion[3] (g)	Reference food & time period	Timepoints	Sample collection	Sample analysis method[4]	Ref.
	New Zealand), tested after a high-CHO evening meal												
149	Fruit and Spice Bread, Vogel's (Goodman Fielder, Auckland, New Zealand), tested after a high-fat evening meal[13]	New Zealand	2010*	59±4	9	Normal, 20	50	NS	Glucose, 2h	Standard	Capillary, whole blood	HemoCue	24
150	Fruit loaf, Bürgen® (Tip Top Bakeries, Chatswood, Australia)	Australia	1997	44±5	7	Normal, 10	50	NS	Bread, 2h	Standard	Capillary, plasma	Enzymatic	25
	Fruit and Muesli bread, Bürgen® (Tip Top Bakeries, 151 Australia)	Australia	2006-2007	53±4	8	Normal, 10	50	132.6	Glucose, 2h	Standard	Capillary, whole blood	HemoCue	UO[10]
152	Fruit and cinnamon bread (Finest, UK)	UK	2005	71±11	11	Normal, 10	50	94.9	Glucose, 2h	Standard	Capillary, whole blood	Glucometer[9]	26
	Fruit and Spice Loaf, thick sliced (Buttercup bakeries, 153 Moorebank, Australia)	Australia	2003	54±6	8	Normal, 10	50	95.1	Glucose, 2h	Standard	Capillary, plasma	Enzymatic	UO[5]
154	Fruit loaf, sliced	UK	2005	57±6	9	Normal, 10	50	91.9	Glucose, 2h	Standard	Capillary, whole blood	Glucometer[9]	26
155	Cinnamon, raisin, pecan bread, Happiness™ (Natural Ovens, Manitowoc, USA)	USA	1999	63±5	9	Normal, 10	50	133.3	Bread, 2h	Standard	Capillary, plasma	Enzymatic	UO[5]
156	Muesli bread, made from packet mix in bread making machine (Con Agra Inc., USA)	USA	1999	54±6	8	Normal, 10	50	122.9	Bread, 2h	Standard	Capillary, plasma	Enzymatic	UO[5]
157	Raisin Toast, TipTop™ (Tip Top Bakeries, Australia)	Australia	2016	53±3	8	Normal, 10	50	94.5	Glucose, 2h	Standard	Capillary, plasma	Enzymatic	UO[5]
158	Whole-wheat bread with dried fruit	China	2005	47±2	7	Normal, 10	50	NS	Glucose, 2h	Standard	Venous, NS	NS	12
	Fruit bread, mean of 12 foods			57									
	**Gluten-free breads**												
159	Multigrain bread, gluten-free (Country Life Bakery, Dandenong, Australia)	Australia	2000	79±13	12	Normal, 10	50	131.9	Glucose, 2h	Standard	Capillary, whole blood	HemoCue	UO[5]
160	White bread, gluten free (Country Life Bakery, Australia)	Australia	2006	40±5	6	Normal, 10	50	183.2	Glucose, 2h	Standard	Capillary, whole blood	HemoCue	UO[5]
161	White bread, gluten free, sliced (COOP Italia-Soc. Coop a.r.l., [8]	Italy	2015*	61±6	9	Normal, 10	50	124	Glucose, 2h	Standard	Capillary, whole blood	YSI	5

Food Number and Item		Country of food production	Year of test[1]	GI[2] ± SEM (Glu = 100)	GL	Subjects (type & number)	Avail carb (Test portion)	Test portion[3] (g)	Reference food & time period	Timepoints	Sample collection	Sample analysis method[4]	Ref.
	Italy)												
162	White roll bread, gluten free (Dr Schär AG/SPA, Italy)[8]	Italy	2015*	63±8	9	Normal, 10	50	112	Glucose, 2h	Standard	Capillary, whole blood	YSI	5
163	White sourdough bread, gluten free, sliced (Dr Schär AG/SPA, Italy)[8]	Italy	2015*	52±7	8	Normal, 10	50	132	Glucose, 2h	Standard	Capillary, whole blood	YSI	5
	Gluten-free breads, mean of five foods			59									
	**Hamburger/Hot dog buns/Rolls**												
164	Burger Buns, 100% Whole wheat Gigantico, President's ® Blue Menu™ (Loblaw Brands Limited, Canada) Choice	Canada	2007	62±6	9	Normal, 10	50	127.4	Bread, 2h	Standard	Capillary, whole blood	YSI	UO[7]
165	HermanBurger Protein Bun (Herman Brot Pty Ltd, Australia)	Australia	2018	31±4	5	Normal, 10	25	219.3	Glucose, 2h	Standard	Capillary, plasma	Enzymatic	UO[5]
166	Hot Dog Rolls, 100% Whole wheat Gigantico, President's ® Blue Menu™(Loblaw Brands Limited, Canada) Choice	Canada	2007	62±6	9	Normal, 10	50	125.0	Bread, 2h	Standard	Capillary, whole blood	YSI	UO[7]
167	Mixed Grain bread roll, Bürgen™(Tip Top Bakeries, Chatswood, Australia)	Australia	2008	52±4	8	Normal, 14	50	165.6	Glucose, 2h	Standard	Capillary, plasma	Enzymatic	UO[5]
168	Soft bread rolls, Pagnottelle classic, soft wheat flour, sugar (5.4%) (Mulino Bianco, Barilla, Italy)	Italy	2009	62±11	9	Normal, 9	50	98	Glucose, 2h	Standard	Capillary, whole blood	YSI	3
169	Soft bread rolls, Sfilatini al grano tenero (Mulino Bianco, Barilla, Italy)	Italy	2009	65±9	10	Normal, 9	50	99	Glucose, 2h	Standard	Capillary, whole blood	YSI	UO[14]
	**Mixed grain or seed wheat breads**												
170	Classic Seed Loaf, Helga's™ (Quality Bakers, Sydney, Australia)	Australia	2007	68±9	10	Normal, 10	50	120.4	Glucose, 2h	Standard	Capillary, plasma	Enzymatic	UO[5]
171	Hearty 7 Grain bread, Healthy Choice™ (Con Agra Inc., USA)	USA	2000	55±6	8	Normal, 10	50	130.9	Glucose, 2h	Standard	Capillary, plasma	Enzymatic	UO[5]

172	Hearty 100% Whole Grain bread, Healthy Choice™ (Con Agra Inc., USA)	USA	2000	62±6	9	Normal, 10	50	140.8	Glucose, 2h	Standard	Capillary, plasma	Enzymatic	UO[5]
173	Kibbled Konini bread (Country Fare, New Zealand)	New Zealand	2000	68±12	10	Normal, 14	50	NS	Glucose, 2h	Standard	Venous, plasma	Enzymatic	27
174	Kibbled wheat bread, Lawson's Settlers Grain (Goodman Fielder, Australia)	Australia	2008	65±5	10	Normal, 9	50	121.1	Glucose, 2h	Standard	Capillary, plasma	Enzymatic	UO[5]
175	Linola seed bread	China	2010*	90±11	14	Normal, 10	50	116.8	Glucose, 2h	Standard	Capillary, whole blood	HemoCue	28
176	Mixed Grain bread, Bürgen™ (Tip Top Bakeries, Chatswood, Australia)	Australia	1997	34±4	5	Normal, 10-12	50	NS	Bread, 2h	Standard	Capillary, plasma	Enzymatic	25
177	Mixed Grain, Bürgen™ (Tip Top Bakeries, Chatswood, Australia)	Australia	2007	52±4	8	Normal, 12	50	NS	Glucose, 2h	Standard	Capillary, whole blood	HemoCue	UO[15]
	Mixed grain sandwich bread, Molenberg™ (Goodman 178 Fielder, Auckland, New Zealand)	New Zealand	2000	75±10	11	Normal, 15	50	NS	Glucose, 2h	Standard	Venous, plasma	Enzymatic	27
179	Multigrain batch bread	UK	2005	62±8	9	Normal, 10	50	104.8	Glucose, 2h	Standard	Capillary, whole blood	Glucometer[9]	26
180	Multigrain bread (High 5 Silver Bird Group Berhad, Shah Alam, Selangor, Malaysia)	Malaysia	2009*	56±6	8	Normal, 11	25	57.9	Glucose, 2h	Standard	Capillary, whole blood	Enzymatic	29
181	Multi-Grain bread, 9-Grain (Tip Top Bakeries, Australia)	Australia	1997	43±5	6	Normal, 10-12	50	NS	Bread, 2h	Standard	Capillary, plasma	Enzymatic	25
182	Multigrain loaf, containing wheat flour and coconut flour	Philippines	2002	60±5	9	Normal, 10	50	NS	Bread, 2h	Standard	Capillary, serum	Enzymatic	2

**Food Number and Item**		**Country of food production**	**Year of test[1]**	**GI[2] ± SEM** (Glu = 100)	**GL**	**Subjects** (type & number)	**Avail carb** (Test portion)	**Test portion[3]** (g)	**Reference food & time period**	**Timepoints**	**Sample collection**	**Sample analysis method[4]**	**Ref.**
183	Multiseed bread	UK	2006	54±4	8	Normal, 10	50	119.6	Glucose, 2h	Standard	Capillary, whole blood	Glucometer[9]	30
	Oatbran & Honey bread, Bürgen™ (Tip Top Bakeries, 184 Australia)	Australia	2002	49±4	7	Normal, 10	50	149.7	Bread, 2h	Standard	Capillary, plasma	Enzymatic	UO[5]
185	Organic Heavy Wholegrain bread, Golden Hearth™ (Gold Coast Bakeries, QLD, Australia)	Australia	2004	53±7	8	Normal, 10	50	120.8	Glucose, 2h	Standard	Capillary, plasma	Enzymatic	UO[5]

Food Number and Item		Country of food production	Year of test[1]	GI[2] ± SEM (Glu = 100)	GL	Subjects (type & number)	Avail carb (Test portion)	Test portion[3] (g)	Reference food & time period	Timepoints	Sample collection	Sample analysis method[4]	Ref.
186	Pumpkin Seed bread, containing 6% pumpkin seeds, Bürgen™ (Tip Top Bakeries, Australia)	Australia	2007	49±4	7	Normal, 10	50	184.5	Glucose, 2h	Standard	Capillary, plasma	Enzymatic	UO[15]
187	Seeded bread	UK	2006	49±7	7	Normal, 10	50	130.9	Glucose, 2h	Standard	Capillary, whole blood	Glucometer[9]	30
188	Soy-Lin, kibbled soy (8%) and linseed (8%) loaf, Bürgen™ (Tip Top Bakeries, Australia)	Australia	1997	36±4	5	Normal, 10-12	50	NS	Bread, 2h	Standard	Capillary, plasma	Enzymatic	25
189	Soy & Linseed bread (made from packet mix cooked in bread maker) (Con Agra Inc., USA)	USA	1999	50±6	8	Normal, 10	50	165.0	Bread, 2h	Standard	Capillary, plasma	Enzymatic	UO[5]
190	Soy & Linseed bread (Coles Supermarkets Pty Ltd, Australia)	Australia	2015	56±7	8	Normal, 10	50	130.5	Glucose, 2h	Standard	Capillary, plasma	Enzymatic	UO[5]
191	Sprouted wheat bread, Sprouted 3 Grains bread (Stonemill Bread, Toronto, Canada)	Canada	2007	55±6	8	Normal, 10	50	150.6	Bread, 2h	Standard	Capillary, whole blood	YSI	UO[7]
192	Whole grain bread, Hunger Filler™ (Natural Ovens, USA)	USA	1999	59±8	9	Normal, 10	50	154.3	Bread, 2h	Standard	Capillary, plasma	Enzymatic	UO[5]
193	Whole grain bread, Nutty Natural™ (Natural Ovens, USA)	USA	1999	59±7	9	Normal, 10	50	154.8	Bread, 2h	Standard	Capillary, plasma	Enzymatic	UO[5]
194	Whole grain bread, Stay Trim™ (Natural Ovens, USA)	USA	1999	70±10	11	Normal, 10	50	172.4	Bread, 2h	Standard	Capillary, plasma	Enzymatic	UO[5]
195	Wholemeal Country Grain bread, Bakers Delight™ (Bakers Delight Holdings, Australia)	Australia	2005	53±9	8	Normal, 10	50	126.6	Glucose, 2h	Standard	Capillary, whole blood	HemoCue	UO[5]
196	Wholemeal & Seeds bread, Bürgen™ (Tip Top Bakeries, Australia)	Australia	2007	39±8	6	Normal, 10	50	194.6	Glucose, 2h	Standard	Capillary, plasma	Enzymatic	UO[15]
	Mixed grain or seed bread, mean of 27 foods			56									
	**Mixed grain or seed wheat bread, lower carbohydrate**												
197	Lower Carb Bread (Herman Brot Pty Ltd, Australia)	Australia	2013	24±3	4	Normal, 10	25	337.8	Glucose, 2h	Standard	Capillary, plasma	Enzymatic	UO[5]
198	Lower Carb 5 Seeds Bread, Helga's™ (Quality Bakers, Sydney, Australia)	Australia	2018	53±5	8	Normal, 10	50	172.4	Glucose, 2h	Standard	Capillary, plasma	Enzymatic	UO[5]

	Lower Carb Soy & Toasted Sesame Bread, Helga's™ 199 (Quality Bakers, Sydney, Australia)	Australia	2018	51±4	8	Normal, 10	50	176.0	Glucose, 2h	Standard	Capillary, plasma	Enzymatic	UO[5]
200	Lower Carb Wholemeal & Seeds Bread, Helga's™ (Quality Bakers, Sydney, Australia)	Australia	2018	51±5	8	Normal, 10	50	173.6	Glucose, 2h	Standard	Capillary, plasma	Enzymatic	UO[5]
	Mixed grain or seed wheat bread, lower carbohydrate mean of four foods			45									
	**Oat breads**												
201	Bread, short oat (50% wholemeal wheat, 20% rye, 10% oatmeal, 20% white wheat, yeast (1.3% net flour weight), no rising time, 30 min proving time, 220°C baking temperature, 30 min bake[13]	New Zealand	2010*	77±6	12	Normal, 10	50	111	Glucose, 2h	Standard	Capillary, plasma	Enzymatic	31
202	Bread, wholemeal with oatmeal (High 5 Silver Bird Group Berhad, Shah Alam, Selangor, Malaysia)	Malaysia	2009*	67±7	10	Normal, 12	25	56.3	Glucose, 2h	Standard	Capillary, whole blood	Enzymatic	29
203	Degraded oat bran concentrate bread (50% oat bran concentrate + 50% wheat, 5 h proving time)	Norway	2019*	64±5	10	Normal, 14	25	96.4	Glucose, 2h	Standard	Capillary, plasma	YSI	32
204	Optimal oat bran concentrate bread (50% oat bran concentrate + 50% wheat, separate proving of wheat dough part)	Norway	2019*	57±4	9	Normal, 14	25	97	Glucose, 2h	Standard	Capillary, plasma	YSI	32
205	Low oat bran concentrate bread (25% oat bran concentrate + 75% wheat, separate proving of wheat dough part)	Norway	2019*	65±4	10	Normal, 14	25	68.6	Glucose, 2h	Standard	Capillary, plasma	YSI	32
206	Oatmeal batch bread	UK	2005	62±8	9	Normal, 10	50	99.8	Glucose, 2h	Standard	Capillary, whole blood	Glucometer[9]	26
	Oat breads, mean of six foods			65									
	**Pretzels**												
207	Soft pretzel, soy[11]	USA	2011*	39±6	6	Normal, 12	50	72.9	Glucose, 2h	Standard	Venous, whole blood	YSI	33
208	Soft pretzel, wheat[11]	USA	2011*	66±4	10	Normal, 12	50	62	Glucose, 2h	Standard	Venous, whole blood	YSI	33
209	Soft pretzel (Ditsch, Mainz, Germany)	Germany	2014	80±6	12	Normal, 11	50	100.2	Glucose, 2h	Standard	Capillary, plasma	Enzymatic	34
	**Rice breads**												
210	Rice bread, high-amylose Doongara rice (Pav's Allergy Bakery, Australia)	Australia	1996	61±9	9	Normal, 12	50	111.1	Bread, 2h	Standard	Capillary, plasma	Enzymatic	UO[5]

211	Rice bread, low-amylose Calrose rice (Pav's Allergy Bakery, Ingleburn, Australia)	Australia	1996	72±9	11	Normal, 12	50	140.0	Bread, 2h	Standard	Capillary, plasma	Enzymatic	UO[5]

Food Number and Item		Country of food production	Year of test[1]	GI[2] ± SEM (Glu = 100)	GL	Subjects (type & number)	Avail carb (Test portion)	Test portion[3] (g)	Reference food & time period	Timepoints	Sample collection	Sample analysis method[4]	Ref.
	**Rye breads**												
212 min	Bread, long rye (50% wholemeal wheat, 30% rye, 20% white wheat, yeast (13% net flour weight), 20 min rising time, 30 proving time, 220°C baking temperature, 30 min bake time[13]	New Zealand	2010*	76±15	11	Normal, 10	50	131	Glucose, 2h	Standard	Capillary, plasma	Enzymatic	31
213	Bread, short rye (50% wholemeal wheat, 30% rye, 20% white wheat, yeast (1.3% net flour weight), no rising time, 30 min proving time, 220°C baking temperature, 30 min bake time[13]	New Zealand	2010*	82±11	12	Normal, 10	50	119	Glucose, 2h	Standard	Capillary, plasma	Enzymatic	31
214	Rye, Bürgen™ (Tip Top Bakeries, Australia)	Australia	2002	51±3	8	Normal, 10	50	155.8	Glucose, 2h	Standard	Capillary, plasma	Enzymatic	UO[5]
215	Rye, Country Grain Organic (Country Life Bakery, Australia)	Australia	2006-2007	53±4	8	Normal, 12	50	NS	Glucose, 2h	Standard	Capillary, whole blood	HemoCue	UO[10]
216	Pumpernickel Rye bread (Van der Meulen BV, Netherlands)	Netherlands	2015	49±4	7	Normal, 10	50	135.9	Glucose, 2h	Standard	Capillary, plasma	Enzymatic	UO[5]
217	Rye Hi-Soy with Linseed (Country Life Bakery, Australia)	Australia	2006-2007	55±4	8	Normal, 13	50	NS	Glucose, 2h	Standard	Capillary, whole blood	HemoCue	UO[10]
218	Rye wheat sourdough bread (Paderborner, Lieken Urkorn, Dortmund, Germany)	Germany	2014	62±4	9	Normal, 11	50	119	Glucose, 2h	Standard	Capillary, plasma	Enzymatic	34
219	Sourdough rye bread	Australia	2004	48±5	7	Normal, 10	50	116.7	Glucose, 2h	Standard	Capillary, whole blood	HemoCue	UO[5]
220	Sourdough bread, made with rye flour, wholemeal rye flour, spelt flour and intact barley kernels and white bean kernels[11]	Sweden	2020*	67±5	10	Normal, 12	50	143	Glucose, 2h	Standard	Capillary, whole blood	HemoCue	35
221	Sourdough bread, made with rye flour, wholemeal rye flour, spelt flour with intact barley kernels and mung bean kernels[11]	Sweden	2020*	65±4	10	Normal, 12	50	146	Glucose, 2h	Standard	Capillary, whole blood	HemoCue	35
222	Sprouted rye bread	Australia	2008	61±5	9	Normal, 10	50	145.3	Glucose, 2h	Standard	Capillary, plasma	Enzymatic	UO[5]

Food Number and Item		Country of food production	Year of test[1]	GI[2] ± SEM (Glu = 100)	GL	Subjects (type & number)	Avail carb (Test portion)	Test portion[3] (g)	Reference food & time period	Timepoints	Sample collection	Sample analysis method[4]	Ref.
223	Rye, made with 30% wholemeal wheat, 20% kibbled rye, 30% wheat flour, and baker's yeast[13]	New Zealand	2010*	60±3	9	Normal, 10	50	154	Glucose, 2h	Standard	Capillary, plasma	Enzymatic	31
224	Wholemeal rye bread with intact grains and sunflower seeds (Kraftkerni, Lieken Urkorn, Dortmund, Germany)	Germany	2014	55±4	8	Normal, 12	50	151.5	Glucose, 2h	Standard	Capillary, plasma	Enzymatic	34
	Rye breads, mean of 13 foods			60									
	**Sourdough wheat breads**												
225	Sourdough Cob Bread (Fine Breads of Australia, NSW, Australia)	Australia	2008	77±3	12	Normal, 10	50	117.7	Glucose, 2h	Standard	Capillary, plasma	Enzymatic	UO[5]
226	Sourdough wheat bread	Australia	2003	54±5	8	Normal, 10	50	107.1	Glucose, 2h	Standard	Capillary, plasma	Enzymatic	UO[5]
227	Sourdough bread (50% wholemeal wheat, 50% white wheat, sourdough starter (70% wet weight), 80 min rising time, 60 min proving time, 200°C baking temperature, 60 min baking time[13]	New Zealand	2010*	82±13	12	Normal, 10	50	116	Glucose, 2h	Standard	Capillary, plasma	Enzymatic	31
228	Sourdough bread + oats (50% wholemeal wheat, 50% white wheat, 10% rolled oats; sourdough starter (70% wet weight), 80 min rising time, 60 min proving time, 200°C baking temperature, 60 min baking time[13]	New Zealand	2010*	71±10	11	Normal, 10	50	116	Glucose, 2h	Standard	Capillary, plasma	Enzymatic	31
229	Sourdough Vienna bread, Bakers Delight™ (Bakers Delight Holdings, Australia)	Australia	2005	66±6	10	Normal, 10	50	96.3	Glucose, 2h	Standard	Capillary, whole blood	HemoCue	UO[5]
	Sourdough wheat breads, mean of five foods			70									
	**Spelt wheat bread**												
230	Multigrain spelt bread® (Pav's bakery, Australia)	Australia	1996	54±10	8	Normal, 12	50	122.0	Bread, 2h	Standard	Capillary, plasma	Enzymatic	UO[5]
231	Sprouted Spelt bread	Australia	2008	62±6	9	Normal, 10	50	124.1	Glucose, 2h	Standard	Capillary, plasma	Enzymatic	UO[5]
232	White spelt wheat bread	Belgium	2005	66±6	10	Normal, 10	50	95	Bread, 2h	Standard	Capillary, whole blood	Glucometer[9]	36

233	Wholemeal spelt wheat bread (local bakery)	Germany	2014	63±4	9	Normal, 12	50	136.2	Glucose, 2h	Standard	Capillary, plasma	Enzymatic	34
	Spelt bread, mean of four foods			61									
	**White wheat flour bread**												
234	Wheat bread, sliced (Pan Bauletto Bianco)[6]	Italy	2009	59±7	9	Normal, 10	50	93.7	Glucose, 2h	Standard	Capillary, whole blood	YSI	3
235	White bread	UK	2006	59±11	9	Normal, 10	50	112.9	Glucose, 2h	Standard	Capillary, whole blood	Glucometer[9]	30
236	White bread (Giant Eagle King Size Enriched bread, Pittsburgh, PA, USA)[11]	USA	2011*	60±6	9	Normal, 12	50	62	Glucose, 2h	Standard	Venous, whole blood	YSI	33
237	White bread (Pepperidge Farm Original White Bread, USA)[11]	USA	2016*	62±2	9	Normal, 63	50	96.3	Glucose, 2h	Standard	Venous, plasma/serum	Enzymatic	37
238	White bread[11]	France	2019*	63±7	9	Normal, 13	50	63.8	Glucose, 2h	Standard	Capillary, plasma	Enzymatic	7
239	White bread[11]	France	2019*	65±4	10	Normal, 15	50	63.8	Glucose, 2h	Standard	Capillary, whole blood	YSI	7

Food Number and Item		Country of food production	Year of test[1]	GI[2] ± SEM (Glu = 100)	GL	Subjects (type & number)	Avail carb (Test portion)	Test portion[3] (g)	Reference food & time period	Timepoints	Sample collection	Sample analysis method[4]	Ref.
240	White bread	Canada	2009*	66±4	10	Normal, Caucasian, 40	50	109	Glucose, 2h	Standard	Capillary, whole blood	YSI	38
241	White bread	Canada	2009*	68±3	10	Normal, lean, 37	50	109	Glucose, 2h	Standard	Capillary, whole blood	YSI	38
242	White bread	Canada	2009*	69±3	10	Normal, 37	50	109	Glucose, 2h	Standard	Capillary, whole blood	YSI	38
243	White bread, WonderWhite™ (Goodman Fielder Ltd, NSW, Australia)	Australia	2010	70±3	11	Normal, 10	50	119	Glucose, 2h	Standard	Capillary, plasma	Enzymatic	39
244	White bread, WonderWhite™ (Goodman Fielder Ltd, NSW, Australia), tested by subjects with a low AMY1 copy number	Australia	2014	70±3	11	Normal, 19	50	119.9	Glucose, 2h	Standard	Capillary, plasma	Enzymatic	40

Food Number and Item		Country of food production	Year of test[1]	GI[2] ± SEM (Glu = 100)	GL	Subjects (type & number)	Avail carb (Test portion)	Test portion[3] (g)	Reference food & time period	Timepoints	Sample collection	Sample analysis method[4]	Ref.
245	White bread, Sunblest™ (Tip Top Bakeries, Australia)	Australia	1999	70±5	11	Normal, 10	50	105.7	Bread, 2h	Standard	Capillary, plasma	Enzymatic	UO[5]
246	White bread[11]	Taiwan	2012*	71±0.1	11	Normal, 10	50	121.7	Glucose, 2h	Standard	Capillary, plasma	YSI	41
247	White bread	Multiple countries	2001	71±3	11	Normal, 47	50	NS	Glucose, 2h	Standard	Capillary, NS	YSI / Enzymatic	42
248	White bread (Somerfield Plc, Oxford, UK)	UK	2007*	71±5	11	Normal, 15	50	NS	Glucose, 2h	Standard	Capillary, whole blood	Glucometer[9]	43
	White bread (Original White Bread, Pepperidge Farm, 249 Norwalk, CT, USA)	USA	2006	71±6	11	Normal, 14	50	96	Glucose, 2h	Standard	Venous, serum	Enzymatic	44
250	Wheat bread, bite-sized, soft wheat flour (Bocconcini, Panem, Italy)	Italy	2010	71±6	11	Normal, 10	50	89	Glucose, 2h	Standard	Capillary, whole blood	YSI	3
251	White bread	Canada	2009*	72±3	11	Normal, >40 y, 38	50	109	Glucose, 2h	Standard	Capillary, whole blood	YSI	38
252	White bread	Canada	2009*	72±4	11	Normal, ≤40 y, 39	50	109	Glucose, 2h	Standard	Capillary, whole blood	YSI	38
253	White bread	Australia	2014*	74±3	11	Normal 10	50	121	Glucose, 2h	Standard	Capillary, plasma	Enzymatic	17
254	White bread	Canada	2009*	75±4	11	Normal, 40	50	109	Glucose, 2h	Standard	Capillary, whole blood	YSI	38
255	White bread (Nishin Shokuhin, Japan)[16]	Japan	2003	75±10	11	Normal, 10	50	116	Rice[17], 2h	Standard	Capillary, whole blood	Glucometer[9]	45
	White bread (Vodova veka Penam, Olomouc, Czech 256 Republic)	Czech Republic	2003	75±10	11	Normal, 11	50	85	Glucose, 2h	Standard	Capillary, whole blood	Glucometer[9]	46
257	White bread	Canada	2009*	76±4	11	Normal, overweight, 40	50	109	Glucose, 2h	Standard	Capillary, whole blood	YSI	38
258	White bread[11]	France	2019*	76±6	11	Normal, 15	50	63.8	Glucose, 2h	Standard	Capillary, plasma	Enzymatic	7

Food Number and Item		Country of food production	Year of test[1]	GI[2] ± SEM (Glu = 100)	GL	Subjects (type & number)	Avail carb (Test portion)	Test portion[3] (g)	Reference food & time period	Timepoints	Sample collection	Sample analysis method[4]	Ref.
259	White bread, containing 9% sourdough, baked for 30 min at[13] 210°C	Spain	2013	76±8	11	Normal, 22	50	95	Glucose, 2h	Standard	Venous, plasma/serum	Enzymatic	47
260	White bread	Multiple countries	2001	76±13	11	Normal, 21	50	NS	Glucose, 2h	Standard	Venous, NS	YSI / Enzymatic	42
261	Wheat bread, sliced (Harry's)	Italy	2005-2015	76±14	11	Normal,	50	96.2	Glucose, 2h	Standard	Capillary, whole blood	YSI	4
262	White bread	Canada	2009*	78±4	12	Normal, Non-Caucasian, 37	50	109	Glucose, 2h	Standard	Capillary, whole blood	YSI	38
263	White bread, WonderWhite™ (Goodman Fielder Ltd, NSW, Australia), tested by subjects with a high AMY1 copy number	Australia	2014	81±3	12	Normal, 19	50	119.9	Glucose, 2h	Standard	Capillary, plasma	Enzymatic	40
264	White bread (Gardenia Bakeries (KL) Sdn Bhd Shah Alam, Selangor)	Malaysia	2009*	83±7	12	Normal, 12	25	51.9	Glucose, 2h	Standard	Capillary, whole blood	Enzymatic	29
265	White bread (Gardenia brand, NTUC Fairprice, Singapore)	Singapore	2015*	83±9	12	Normal, 11	50	91.4	Glucose, 2h	Standard	Capillary, whole blood	HemoCue	48
266	Wheat bread	Norway	2019*	84±7	13	Normal, 14	25	51	Glucose, 2h	Standard	Capillary, plasma	YSI	32
267	White bread (lab made)	China	2020	87±11	13	Normal, 16	50	65.2	Glucose, 2h	Standard	Capillary, plasma	Enzymatic	UO[18]
268	White bread	China	2005	88±3	13	Normal, 10	50	NS	Glucose, 2h	Standard	Venous, NS	NS	12
	White wheat flour bread, mean of 35 foods			73									
	**White wheat flour bread, other**												
269	Baguette, Whole Grain, President's Choice® Blue Menu™ (Loblaw Brands Limited, Canada)	Canada	2007	73±6	11	Normal, 10	50	119.0	Bread, 2h	Standard	Capillary, whole blood	YSI	UO[7]
270	Bread, bakery made from an industrial mix for bread preparation (Pan Gustavo, Novaterra Zeelandia, Italy)	Italy	2014	72±8	11	Normal, 10	25	100	Glucose, 2h	Standard	Capillary, whole blood	YSI	3

271	Bread, bakery made from an industrial mix for bread preparation (Pan Semola, Novaterra Zeelandia, Italy)	Italy	2014	87±11	13	Normal, 10	25	51	Glucose, 2h	Standard	Capillary, whole blood	YSI	3
272	Bread, sliced, soft wheat flour, low GI and high fiber (Pane a fette a basso indice glicemico e ad alto contenuto di fibre, Roberto Industria Alimentare, Italy)	Italy	2011	41±2	6	Normal, 9	25	65	Glucose, 2h	Standard	Capillary, whole blood	YSI	3
273	Pan Bauletto 5 cereali e soia (Mulino Bianco, Barilla, Italy)	Italy	2009	60±9	9	Normal, 9	50	115	Glucose, 2h	Standard	Capillary, whole blood	YSI	3
274	Bread, sliced, durum wheat flour (Pan Bauletto al grano duro, Mulino Bianco, Barilla, Italy)	Italy	2009	68±7	10	Normal, 9	50	104	Glucose, 2h	Standard	Capillary, whole blood	YSI	3
275	White bread, Alfacar[13]	Spain	2013	68±8	10	Normal, 22	50	94	Glucose, 2h	Standard	Venous, plasma/serum	Enzymatic	47
276	White bread, candeal-flour[13]	Spain	2013	86±8	13	Normal, 22	50	94	Glucose, 2h	Standard	Venous, plasma/serum	Enzymatic	47
277	White sandwich bread, low GI, high fiber, Bakers Delight™ Hi Fibre Lo GI (Bakers Delight Holdings, Australia)	Australia	2006	52±7	8	Normal, 10	50	98.8	Glucose, 2h	Standard	Capillary, whole blood	HemoCue	UO[5]
278	White sandwich bread, low GI, high fiber, Tip Top™ EnerGI (Tip Top Bakeries, George Weston Ltd, Australia)	Australia	2007	58±3	9	Normal, 20	50	111.6	Glucose, 2h	Standard	Capillary, plasma	Enzymatic	UO[5,10]
279	White bread, low GI, high fiber, Tip Top™ EnerGI (Tip Top Bakeries, George Weston Ltd, Australia)	Australia	2007	54±4	8	Normal, 11	50	111.6	Glucose, 2h	Standard	Capillary, plasma	Enzymatic	UO[15]
280	White sandwich bread, low GI, high fiber, Vogel's Wonder White 'Low GI' (Quality Bakers, Australia)	Australia	2006-2007	54±3	8	Normal, 10	50	135.1	Glucose, 2h	Standard	Capillary, whole blood	HemoCue	UO[10]
281	White sandwich bread, low GI, high fiber, Vogel's Wonder White 'Low GI' (Quality Bakers, Australia)	Australia	2007	59±3	9	Normal, 20	50	135.1	Glucose, 2h	Standard	Capillary, plasma	Enzymatic	UO[5]
282	White sandwich bread, Low GI, high fiber, Low GI Wonder Active (Quality Bakers, Sydney, Australia)	Australia	2019	52±4	8	Normal, 10	50	131.9	Glucose, 2h	Standard	Capillary, plasma	Enzymatic	UO[5]
283	White bread, toasted (Hovis, UK)	UK	2005	50±7	8	Normal, 10	50	101.6	Glucose, 2h	Standard	Capillary, whole blood	Glucometer[9]	49

Food Number and Item		Country of food production	Year of test[1]	GI[2] ± SEM (Glu = 100)	GL	Subjects (type & number)	Avail carb (Test portion)	Test portion[3] (g)	Reference food & time period	Timepoints	Sample collection	Sample analysis method[4]	Ref.
284	Enriched white bread, Wonder™ (Interstate Brands Companies, Kansas City, USA)	USA	1999	71±9	11	Normal, 10	50	111.1	Bread, 2h	Standard	Capillary, plasma	Enzymatic	UO[5]
285	Enriched white bread, Wonder™ (Interstate Brands Companies, Kansas City, USA)	USA	2000	72±4	11	Normal, 10	50	111.1	Glucose, 2h	Standard	Capillary, plasma	Enzymatic	UO[5]
286	Enriched white bread, Wonder™ (Interstate Brands Companies, Kansas City, USA)	USA	2000	77±3	12	Normal, 10	50	108	Glucose, 2h	Standard	Capillary, plasma	Enzymatic	UO[5]
	**White bread with added ingredients**												
287	Bread, sliced, soft wheat flour, olive oil (2.5%) (Pane Bianco, Roberto Industria Alimentare, Italy)	Italy	2011	63±4	9	Normal, 10	25	55	Glucose, 2h	Standard	Capillary, whole blood	YSI	3
288	Bread, sliced, soft wheat flour, extra virgin olive oil (Pane Bianco all'olio d'oliva, Coop, Italy)	Italy	2011	73±7	11	Normal, 10	25	50	Glucose, 2h	Standard	Capillary, whole blood	YSI	3
289	White bread (100% wheat flour) + 2% pectin (George Weston Foods Ltd, Australia)	Australia	1998	86±8	13	Normal, 10	50	NS	Bread, 2h	Standard	Capillary, plasma	Enzymatic	UO[5]
290	White bread (100% wheat flour) + 5% fruit fiber (George Weston Foods Ltd, Australia)	Australia	1998	77±9	12	Normal, 10	50	NS	Bread, 2h	Standard	Capillary, plasma	Enzymatic	UO[5]
291	White bread (80% wheat flour + 20% chickpea flour) (George Weston Foods Ltd, Australia)	Australia	1998	80±9	12	Normal, 10	50	NS	Bread, 2h	Standard	Capillary, plasma	Enzymatic	UO[5]
292	White bread (90% wheat flour + 10% modified corn starch) (George Weston Foods Ltd, Australia)	Australia	1998	79±5	12	Normal, 10	50	NS	Bread, 2h	Standard	Capillary, plasma	Enzymatic	UO[5]
293	White bread (90% wheat flour + 10% tapioca starch) (George Weston Foods Ltd, Australia)	Australia	1998	78±11	12	Normal, 10	50	NS	Bread, 2h	Standard	Capillary, plasma	Enzymatic	UO[5]
	White bread (95% wheat flour + 5% oat bran) (George 294 Weston Foods Ltd, Australia)	Australia	1998	75±6	11	Normal, 10	50	NS	Bread, 2h	Standard	Capillary, plasma	Enzymatic	UO[5]
295	White bread, containing 2% guar gum (George Weston Foods Ltd, Australia)	Australia	1998	67±6	10	Normal, 10	50	NS	Bread, 2h	Standard	Capillary, plasma	Enzymatic	UO[5]

Food Number and Item		Country of food production	Year of test[1]	GI[2] ± SEM (Glu = 100)	GL	Subjects (type & number)	Avail carb (Test portion)	Test portion[3] (g)	Reference food & time period	Timepoints	Sample collection	Sample analysis method[4]	Ref.
296	White bread, containing 2% vinegar and 2.5% sourdough (Noble Rise Crunchy Toast, Australia)	Australia	1998	80±4	12	Normal, 10	50	NS	Bread, 2h	Standard	Capillary, plasma	Enzymatic	UO[5]
297	Bread, fibre-enriched flour (Pan Bauletto Fior di Fibra, Mulino Bianco, Barilla, Italy)	Italy	2009	53±5	8	Normal, 10	50	120	Glucose, 2h	Standard	Capillary, whole blood	YSI	3
298	White bread, fiber-enriched, Fiber White™ (Nature's Fresh, Auckland, New Zealand)	New Zealand	2000	77±10	12	Normal, 14	50	NS	Glucose, 2h	Standard	Venous, plasma	Enzymatic	27
299	White bread with 2.5 g Filtered Molasses Concentrate added per 100 g (6.04 g FMC/100 g carbohydrate)	Australia	2014*	59±6	9	Normal 10	50	121	Glucose, 2h	Standard	Capillary, plasma	Enzymatic	17
300	Wheat bread enriched with ß-glucan (6 g per serving)	Greece	2020*	48±10	7	Normal, 10	50	166	Glucose, 2h	Standard	Venous, plasma/serum	YSI	50
301	White bread, containing 0.8% Reducose®, lab made	China	2020	42±4	6	Normal, 10	50	65.2	Glucose, 2h	Standard	Capillary, plasma	Enzymatic	UO[18]
302	Wheat bread enriched with resistant starch (high amylose waxy maize starch; 8.8 g/serving)	Greece	2020*	40±8	6	Normal, 10	50	166	Glucose, 2h	Standard	Venous, plasma/serum	YSI	50
303	White bread (10% of white bread flour was substituted by resistant starch from tapioca)[11]	Taiwan	2012*	70±0.1	11	Normal, 10	50	144.5	Glucose, 2h	Standard	Capillary, plasma	YSI	41
304	White bread (30% of white bread flour was substituted by resistant starch from tapioca)[11]	Taiwan	2012*	68±0.2	10	Normal, 10	50	204.9	Glucose, 2h	Standard	Capillary, plasma	YSI	41
305	White bread (60% of white bread flour was substituted by resistant starch from tapioca)[11]	Taiwan	2012*	51±0.1	8	Normal, 10	50	221.2	Glucose, 2h	Standard	Capillary, plasma	YSI	41
	**White bread, with different proving times and bread volumes**												
306	White bread, prepared with a 10 min prove and a second 2 min proving (low loaf volume)	UK	2006	38±4	6	Normal, 10	50	NS	Glucose, 2h	Standard	Capillary, whole blood	Glucometer[9]	51
307	White bread, prepared with a 30 min prove and a second 12 min proving (moderate loaf volume)	UK	2006	72±7	11	Normal, 10	50	NS	Glucose, 2h	Standard	Capillary, whole blood	Glucometer[9]	51

Food Number and Item	Country of food production	Year of test[1]	GI[2] ± SEM (Glu = 100)	GL	Subjects (type & number)	Avail carb (Test portion)	Test portion[3] (g)	Reference food & time period	Timepoints	Sample collection	Sample analysis method[4]	Ref.
308 White bread, prepared with a 60 min prove and a second 30 min proving (moderate loaf volume)	UK	2006	86±9	13	Normal, 10	50	NS	Glucose, 2h	Standard	Capillary, whole blood	Glucometer[9]	51
309 White bread, prepared with a 40 min prove, a second 25 min proving and a third 50 min proving (large loaf volume)	UK	2006	100±7	15	Normal, 10	50	NS	Glucose, 2h	Standard	Capillary, whole blood	Glucometer[9]	51
**White bread with different processing conditions**												
310 Western baked bread, made with high protein flour (15 min resting time, 70 min proving time, 11 min baking at 210°C)	Singapore	2014*	71±5	11	Normal, 13	50	102	Glucose, 2h	Standard	Capillary, whole blood	HemoCue	52
311 White bread, pre-cooked frozen, baked 16 min at 180°C, cooled at 30°C for 18 min; frozen at -30°C for 30 min; preserved at -18°C; 2nd baking at 230°C for 12 min[13]	Spain	2013	78±8	12	Normal, 22	50	100	Glucose, 2h	Standard	Venous, plasma/serum	Enzymatic	47
312 Modified steamed bread made with conventional baked bread recipe, using high protein flour (15 min resting time, 40 min proving time, 10 min steaming at 100°C)	Singapore	2014*	65±4	10	Normal, 13	50	120	Glucose, 2h	Standard	Capillary, whole blood	HemoCue	52
313 Oriental steamed bread, made with medium protein flour (15 min resting time, 40 min proving time, 10 min steaming at 100°C)	Singapore	2014*	68±5	10	Normal, 13	50	105	Glucose, 2h	Standard	Capillary, whole blood	HemoCue	52
314 Modified baked bread made with steamed bread recipe, made with medium protein flour (15 min resting time, 70 min proving time, 11 min baking at 210°C)	Singapore	2014*	75±4	11	Normal, 13	50	87	Glucose, 2h	Standard	Capillary, whole blood	HemoCue	52
**Wholemeal (whole wheat) wheat flour bread**												
315 Wholemeal wheat flour bread, sliced (Pan Bauletto Integrale, Italy)[19]	Italy	20052015	59±5	9	Normal, 10	50	126.9	Glucose, 2h	Standard	Capillary, whole blood	YSI	3
316 Wholemeal flour bread	China	2005	69±3	10	Normal, 10	50	NS	Glucose, 2h	Standard	Venous, NS	NS	12
317 Wholemeal flour bread	Australia	2012	75±5	11	Normal, 10	50	125.0	Glucose, 2h	Standard	Capillary, plasma	Enzymatic	UO[5]
318 Wholemeal flour bread, short wholemeal (50% whole meal wheat, 50% white wheat, Baker's yeast (1.3% net flour weight), no rising time, 30 min proving time, 220°C baking temperature, 30 min baking time[13]	New Zealand	2010*	78±5	12	Normal, 10	50	111	Glucose, 2h	Standard	Capillary, plasma	Enzymatic	31

Food Number	and Item	Country of food production	Year of test[1]	GI[2] ± SEM (Glu = 100)	GL	Subjects (type & number)	Avail carb (Test portion)	Test portion[3] (g)	Reference food & time period	Timepoints	Sample collection	Sample analysis method[4]	Ref.
319	Wholemeal bread, long whole meal (50% whole meal wheat, 50% white wheat, Baker's yeast (13% net flour weight), 20 min rising time, 30 min proving time, 220°C baking temperature, 30 min baking time[13]	New Zealand	2010*	80±7	12	Normal, 10	50	115	Glucose, 2h	Standard	Capillary, plasma	Enzymatic	31
320	Wholemeal bread (Gardenia Bakeries (KL) Sdn Bhd Shah Alam, Selangor, Malaysia)	Malaysia	2009*	85±6	13	Normal, 11	25	80	Glucose, 2h	Standard	Capillary, whole blood	Enzymatic	29
321	Wholemeal bread, yeast (50% wholemeal wheat, 50% white wheat, Baker's yeast (15% dry weight), 60 min rising time, 40 min proving time, 200°C baking temperature, 40-50 min baking time[13]	New Zealand	2010*	88±8	13	Normal, 10	50	119	Glucose, 2h	Standard	Capillary, plasma	Enzymatic	31
322	Wholemeal bread, smooth milled, Ploughman's™ (Quality Bakers, Australia)	Australia	1996	64±10	10	Normal, 12	50	117.1	Bread, 2h	Standard	Capillary, plasma	Enzymatic	UO[5]
	Wholemeal wheat flour bread, mean of eight foods			75									
323	Wholemeal stoneground flour sourdough bread (Bill's Organic Bread, Cardiff, Australia)	Australia	2003	59±8	9	Normal, 10	50	135.9	Glucose, 2h	Standard	Capillary, whole blood	HemoCue	UO[5]
324	Wholemeal sliced bread, High fibre Performax™ (Country Life Bakery, Australia)	Australia	1999	38±3	6	Normal, 10	50	NS	Bread, 2h	Standard	Capillary, plasma	Enzymatic	UO[5]
325	Wholemeal bread (wholemeal wheat flour, water, yeast, salt, and 40% sourdough), baked at 200°C for 40 min[13]	Spain	2013	61±8	9	Normal, 22	50	134	Glucose, 2h	Standard	Venous, plasma/serum	Enzymatic	47
326	Wholemeal bread sticks, crunchy	Crete	2006	67±5	10	Normal, 10	50	84.7	Glucose, 2h	Standard	Capillary, plasma	Enzymatic	UO[5]
	**Wrap breads**												
327	Corn tortilla, made from white corn, Diego's brand (San Diego Tortilla Factory Pty Ltd, Andrews, QLD, Australia)	Australia	2007	49±6	7	Normal, 9	50	121.1	Glucose, 2h	Standard	Capillary, plasma	Enzymatic	UO[5]
328	Corn tortilla, made from white corn, gluten-free, Mission® (Mission Foods Australia)	Australia	2010	52±4	8	Normal, 9	50	121.1	Glucose, 2h	Standard	Capillary, plasma	Enzymatic	UO[5]
329	Protein Wraps (Herman Brot Pty Ltd, Australia)	Australia	2019	27±3	4	Normal, 10	25	176.1	Glucose, 2h	Standard	Capillary, plasma	Enzymatic	UO[5]

Food Number and Item	Country of food production	Year of test[1]	GI[2] ± SEM (Glu = 100)	GL	Subjects (type & number)	Avail carb (Test portion)	Test portion[3] (g)	Reference food & time period	Timepoints	Sample collection	Sample analysis method[4]	Ref.
330 Mixed Grain wraps, Helga's (Quality Bakers Australia Pty Ltd, Australia)	Australia	2015	55±6	8	Normal, 10	50	105.3	Glucose, 2h	Standard	Capillary, plasma	Enzymatic	UO[5]
331 Oat Light Wrap bread (Mountain Bread, VIC, Australia)	Australia	2008	62±6	9	Normal, 10	50	94.0	Glucose, 2h	Standard	Capillary, plasma	Enzymatic	UO[5]
332 Whole Grain Tortilla, Old El Paso™ (General Mills Australia Pty Ltd)	Australia	2015	50±5	8	Normal, 10	50	106.2	Glucose, 2h	Standard	Capillary, plasma	Enzymatic	UO[5]
333 Wholemeal and white flour wrap with chia seeds, Mission brand (Gruma Oceania Pty Ltd, Australia)	Australia	2015	50±6	8	Normal, 10	50	127.6	Glucose, 2h	Standard	Capillary, plasma	Enzymatic	UO[5]
334 Wholemeal and white flour wrap with red quinoa, Mission brand (Gruma Oceania Pty Ltd, Australia)	Australia	2015	59±5	9	Normal, 10	50	122.2	Glucose, 2h	Standard	Capillary, plasma	Enzymatic	UO[5]
**Other Special wheat breads**												
Chia White bread, Bakers Delight™ (Bakers Delight 335 Holdings, Australia)	Australia	2010	63±3	9	Normal, 10	50	117.1	Glucose, 2h	Standard	Capillary, plasma	Enzymatic	UO[5]
Bread, increased GI variant (sugar-to-flour ratio: 0.24) 336 (Bakery School, Herk-de-Stad, Belgium)	Belgium	2010*	69±15	10	Normal, 10	25	55.6	Glucose, 2h	Standard	Venous, plasma/serum	Enzymatic	1
337 Bread, decreased GI variant (sugar-to-flour ratio: 0.0) (Bakery School, Herk-de-Stad, Belgium)	Belgium	2010*	40±4	6	Normal, 10	25	58.1	Glucose, 2h	Standard	Venous, plasma/serum	Enzymatic	1
338 Bread, sliced, soft wheat flour, flaxseeds (4.9%), soy grain (4.3%) (Cuor di Lino, Mulino Bianco, Barilla, Italy)	Italy	2012	58±10	9	Normal, 9	50	127	Glucose, 2h	Standard	Capillary, whole blood	YSI	3
339 Bread, desem (50% whole meal wheat, 50% white wheat), baker's yeast (15% dry weight), desem (15% wet weight), 60 min rising time, 40 min proving time, 200°C baking temperature, 40-50 min baking time[13]	New Zealand	2010*	92±10	14	Normal, 10	50	119	Glucose, 2h	Standard	Capillary, plasma	Enzymatic	31
340 Crusty malted wheat bread (Finest, UK)	UK	2005	52±8	8	Normal, 10	50	113.1	Glucose, 2h	Standard	Capillary, whole blood	Glucometer[9]	26
341 Dried wheat bread rusk	Crete	2006	63±6	9	Normal, 10	50	71.4	Glucose, 2h	Standard	Capillary, plasma	Enzymatic	UO[5]

342	Focaccia, soft wheat flour, with extra virgin olive oil (8.7%) (Focaccelle con olio di oliva extravergine, Mulino Bianco, Barilla)	Italy	2015	63±8	9	Normal, 9	25	62	Glucose, 2h	Standard	Capillary, whole blood	YSI	3
343	Lebanese bread, white (Seda Bakery, Sydney, Australia)	Australia	2002	75±9	11	Normal, 10	50	NS	Glucose, 2h	Standard	Capillary, whole blood	HemoCue	UO[5]
344	Malt loaf, organic	UK	2005	59±9	9	Normal, 10	50	81.7	Glucose, 2h	Standard	Capillary, whole blood	Glucometer[9]	26
345	Naan bread (True Foods Pty Ltd, VIC, Australia), heated in microwave for 25 sec	Australia	2013	71±5	11	Normal, 10	50	117.9	Glucose, 2h	Standard	Capillary, plasma	Enzymatic	UO[5]
346	Pain au lait (Pasquier, France)	France	2002	63±10	9	Normal, 11-14	50	94	Glucose, 2h	Standard	Capillary, plasma	Enzymatic	53
347	Pita bread, white, mini	UK	2005	68±5	10	Normal, 10	50	97.7	Glucose, 2h	Standard	Capillary, whole blood	Glucometer[9]	26
348	Pita bread, wholemeal	UK	2005	56±13	8	Normal, 10	50	107.8	Glucose, 2h	Standard	Capillary, whole blood	Glucometer[9]	26
349	Pumpkin Cob Gourmet Bread (Fine Breads of Australia, NSW, Australia)	Australia	2008	85±12	13	Normal, 10	50	121.7	Glucose, 2h	Standard	Capillary, plasma	Enzymatic	UO[5]
350	Roti (unleavened flatbread), whole wheat flour	India	2010*	45±4	7	Normal, 18	50	74.6	Glucose, 2h	Standard	Capillary, whole blood	Glucometer[9]	54
351	Roti (unleavened flatbread), atta mix containing roasted Bengal flour, psyllium husk powder and fenugreek powder	India	2010*	27±2	4	Normal, 18	50	66.7	Glucose, 2h	Standard	Capillary, whole blood	Glucometer	54
352	Sprouted kamut bread	Australia	2008	67±5	10	Normal, 10	50	128.9	Glucose, 2h	Standard	Capillary, plasma	Enzymatic	UO[5]
353	Steamed wheat bread	China	2005	88±7	13	Normal, 9	50	NS	Glucose, 2h	Standard	Venous, NS	NS	12
	**Other special breads**												
354	Barley bread, containing 40% barley flour and 60% wheat flour, separate proving of wheat dough	Norway	2019*	72±6	11	Normal, 14	25	56.7	Glucose, 2h	Standard	Capillary, plasma	YSI	32
355	Lompe (made from potato, precooked and cooled, and spelt flour) (Speltlompe) (Buer AS, Askim, Norway)	Norway	2019*	63±6	9	Normal, 14	25	66.8	Glucose, 2h	Standard	Capillary, plasma	YSI	32
356	Millet steamed bread (75% foxtail-millet flour and 25% extrusion flour (w/w, dry basis)	China	2016*	90±9	14	Normal, 10	50	100	Glucose, 2h	Standard	Venous, plasma/serum	Enzymatic	11
357	Potato mixed flour bread (lab made)	China	2020	55±3	8	Normal, 12	50	107.1	Glucose, 2h	Standard	Capillary, plasma	Enzymatic	UO[18]

358	Soy flour bread	Australia	2005	40±6	6	Normal, 10	50	197.6	Glucose, 2h	Standard	Capillary, whole blood	HemoCue	UO[5]

Food Number and Item		Country of food production	Year of test[1]	GI[2] ± SEM (Glu = 100)	GL	Subjects number)(type &	Avail carb (Test portion)	Test portion[3] (g)	Reference food & time period	Timepoints	Sample collection	Sample analysis method[4]	Ref.
	**BREAKFAST CEREALS**												
	Average available carbohydrate portion = 20 g, this value was used to determine the nominal GL for each item in this category.												
	**Bran Flakes**												
359	Branflakes (Healthy Living, UK)	UK	2005	50±7	10	Normal, 10	50	74.5	Glucose, 2h	Standard	Capillary, whole blood	Glucometer[9]	26
360	Bran Flakes, President's Choice® Blue Menu™(Loblaw Brands Limited, Canada)	Canada	2004-2007	65±8	13	Normal, 10	50	79.0	Bread, 2h	Standard	Capillary, whole blood	YSI	UO[7]
361	Bran Flakes™ (Kellogg's, Australia)	Australia	2000	74±6	15	Normal, 12	50	83.0	Bread, 2h	Standard	Capillary, whole blood	HemoCue	UO[5]
	Bran flakes, mean of three foods			63									
	**Chocapic™ (Nestlé, France)**												
362	Chocapic™, wheat-based flaked cereal	France	2003	70±10	14	Normal, 11	50	62.6	Glucose, 2h	Standard	Capillary, plasma	Enzymatic	UO[5]
363	Chocapic™, wheat-based flaked cereal	France	2003	74±9	15	Normal, 12	50	62.6	Glucose, 2h	Standard	Capillary, plasma	Enzymatic	UO[5]
364	Chocapic™, wheat-based flaked cereal	France	2002	84±9	17	Normal, 11-14	50	60	Glucose, 2h	Standard	Capillary, plasma	Enzymatic	53
	Chocapic, mean of three foods			76									
	**Cornflakes™**												
365	Cornflakes (Kellogg's, Australia)[13], tested in younger adults (19-32 y)	Australia	2014*	64±4	13	Normal, 20	50	NS	Glucose, 2h	Standard	Capillary, whole blood	HemoCue	55
366	Cornflakes[11]	France	2019*	66±5	13	Normal, 13	50	53.5	Glucose, 2h	Standard	Capillary, plasma	Enzymatic	7
367	Cornflakes	China	2005	74±3	15	Normal, 9	50	NS	Glucose, 2h	Standard	Venous, NS	NS	12

Food Number and Item		Country of food production	Year of test[1]	GI[2] ± SEM (Glu = 100)	GL	Subjects (type & number)	Avail carb (Test portion)	Test portion[3] (g)	Reference food & time period	Timepoints	Sample collection	Sample analysis method[4]	Ref.
368	Cornflakes[11]	France	2019*	77±6	15	Normal, 15	50	53.5	Glucose, 2h	Standard	Capillary, plasma	Enzymatic	7
369	Cornflakes[11]	France	2019*	77±6	15	Normal, 15	50	53.5	Glucose, 2h	Standard	Capillary, whole blood	YSI	7
370	Cornflakes	China	2005	79±4	16	Normal, 9	50	NS	Glucose, 2h	Standard	Venous, NS	NS	12
371	Cornflakes (Kellogg's, Australia)[13], tested in older adults (56-86 y)	Australia	2014*	81±3	16	Normal, 60	50	NS	Glucose, 2h	Standard	Capillary, whole blood	HemoCue	55
372	Cornflakes (Kellogg's, Australia)	Australia	2010	82±4	16	Normal, 10	50	59.8	Glucose, 2h	Standard	Capillary, plasma	Enzymatic	39
373	Cornflakes (Kellogg's)	Thailand	2019	93±8	19	Normal, 10	50	60	Glucose, 2h	Standard	Capillary, plasma	Enzymatic	UO[18]
374	Cornflakes (Kellogg's)	Thailand	2020	105±10	21	Normal, 12	50	60	Glucose, 2h	Standard	Capillary, plasma	Enzymatic	UO[18]
	Cornflakes, mean of 10 foods			*80*									
375	Cornflakes, Crunchy Nut™ (Kellogg’s, Australia)	Australia	1999	72±4	14	Normal, 10	50	62.8	Glucose, 2h	Standard	Capillary, plasma	Enzymatic	UO[5]
	**Fruit and Fibre cereals**												
376	Fruit and Fibre	UK	2005	67±7	13	Normal, 10	50	72.4	Glucose, 2h	Standard	Capillary, whole blood	Glucometer[9]	26
377	Fruit and Fibre (Value, UK)	UK	2005	68±7	14	Normal, 10	50	76.1	Glucose, 2h	Standard	Capillary, whole blood	Glucometer[9]	26
	**Granola**												
378	Granola, blueberry	Canada	2010*	64±6	13	Normal, 10	50	92	Bread, 2h	Standard	Capillary, whole blood	YSI	56
379	Granola Clusters, Original, low fat, President’s Choice® Blue Menu™ Loblaw Brands Limited, Canada)	Canada	2004-2007	63±4	13	Normal, 10	50	68.2	Bread, 2h	Standard	Capillary, whole blood	YSI	UO[7]
380	Granola Clusters, Raisin & Almond, low fat, President’s ® Blue Menu™ Loblaw Brands Limited, Canada) Choice	Canada	2004-2007	70±7	14	Normal, 10	50	68.2	Bread, 2h	Standard	Capillary, whole blood	YSI	UO[7]

Food Number and Item		Country of food production	Year of test[1]	GI[2] ± SEM (Glu = 100)	GL	Subjects (type & number)	Avail carb (Test portion)	Test portion[3] (g)	Reference food & time period	Timepoints	Sample collection	Sample analysis method[4]	Ref.
381	Granola, whole-grain oat protein	USA	2018*	51±4	10	Normal, 19	50	87	Glucose, 2h	Standard	Capillary, whole blood	HemoCue	57
382	Low GI Granola Golden Almond Crunch (Sanitarium Health and Food Company, New Zealand)	New Zealand	2019	49±7	10	Normal, 11	50	102	Glucose, 2h	Standard	Capillary, whole blood	HemoCue	UO[20]
383	Low GI Granola Strawberry & Coconut (Sanitarium health and Food Company)	New Zealand	2019	52±6	10	Normal, 11	50	96.2	Glucose, 2h	Standard	Capillary, whole blood	HemoCue	UO[20]
384	Soy Crunch Multi-Grain Cereal, President's Choice® Blue Menu™ (Loblaw Brands Limited, Canada)	Canada	2007	47±4	9	Normal, 10	50		Bread, 2h	Standard	Capillary, whole blood	YSI	UO[7]
385	Soytana™, Vogel's, soy and linseed bran crunch with sultanas, 20.1 g fiber/100 g (Specialty Cereals, Mt Ku-ringgai, NSW, Australia)	Australia	2001	49±3	10	Normal, 10	50	81.1	Glucose, 2h	Standard	Capillary, whole blood	HemoCue	UO[5]
386	Vogel's Cluster Crunch Classic (Specialty Cereals, Australia)	Australia	2006	50±6	10	Normal, 10	50	76.7	Glucose, 2h	Standard	Capillary, whole blood	HemoCue	UO[5]
387	Vogel's Cluster Crunch, Honey Hazelnut (Specialty Cereals, Australia)	Australia	2006	43±3	9	Normal, 10	50	79.5	Glucose, 2h	Standard	Capillary, whole blood	HemoCue	UO[5]
388	Wild Oats Cluster Crunch Hazelnut Chocolate (Specialty Cereals, Australia)	Australia	2006	43±6	9	Normal, 10	50	79.1	Glucose, 2h	Standard	Capillary, whole blood	HemoCue	UO[5]
	Granola, mean of 11 foods			53									
	**Malted cereals**												
389	Malted wholewheat cereal	UK	2008*	56±5	11	Normal Caucasian, 10	50	NS	Glucose, 2h	Standard	Capillary, whole blood	Glucometer[9]	58
390	Malted wholewheat cereal	UK	2008*	60±7	12	Normal Asian, 10	50	NS	Glucose, 2h	Standard	Capillary, whole blood	Glucometer[9]	58
391	Malted wheat cereal	UK	2008*	60±5	12	Normal Caucasian, 10	50	NS	Glucose, 2h	Standard	Capillary, whole blood	Glucometer[9]	58
392	Malted wheat cereal	UK	2008*	68±5	14	Normal Asian, 10	50	NS	Glucose, 2h	Standard	Capillary, whole blood	Glucometer[9]	58

	Malted wheat cereal, mean of four foods			61									
	**Muesli**												
393	Apple & Cinnamon Muesli, Nature’s Source (Bokomo, Cape Town, South Africa)	South Africa	2008	59±6	12	Normal, 10	50	79.4	Glucose, 2h	Standard	Capillary, plasma	Enzymatic	UO[5]
394	Apple & Cranberry Muesli, Balance (Simply Cereal Pty Ltd, Australia)	Australia	2008	67±3	13	Normal, 10	50	72.1	Glucose, 2h	Standard	Capillary, plasma	Enzymatic	UO[5]
395	Bircher Muesli, Apple & Raisin (Aussie Bodies Pty Ltd, VIC, Australia)	Australia	2008	66±11	13	Normal, 10	50	92.6	Glucose, 2h	Standard	Capillary, plasma	Enzymatic	UO[5]
396	Bircher Muesli, Carman’s Natural (Carman’s Fine Foods, Australia)	Australia	2014	48±5	10	Normal, 10	50	101.2	Glucose, 2h	Standard	Capillary, plasma	Enzymatic	UO[5]
397	Fibre-Rich Muesli, Vogel's Café Style (Specialty Cereals, Mt Ku-ring-gai, NSW, Australia)	Australia	2006	48±6	10	Normal, 10	50	78.4	Glucose, 2h	Standard	Capillary, plasma	Enzymatic	UO[5]
398	Fruit Free Muesli, Carman’s Original (Carman’s Fine Foods, Australia)	Australia	2014	45±5	9	Normal, 10	50	107.1	Glucose, 2h	Standard	Capillary, plasma	Enzymatic	UO[5]
399	Fruit & Muesli, Bürgen® (George Weston Foods, Australia)	Australia	2007	51±4	10	Normal, 10	50	NS	Glucose, 2h	Standard	Capillary, whole blood	HemoCue	UO[15]
400	Fruit & Nut Muesli, Carman’s Classic (Carman’s Fine Foods, Australia)	Australia	2014	50±5	10	Normal, 10	50	92.6	Glucose, 2h	Standard	Capillary, plasma	Enzymatic	UO[5]

Food Number and Item		Country of food production	Year of test[1]	GI[2] ± SEM (Glu = 100)	GL	Subjects (type & number)	Avail carb (Test portion)	Test portion[3] (g)	Reference food & time period	Timepoints	Sample collection	Sample analysis method[4]	Ref.
	Liver Cleansing Muesli (Food For Health Life Food, 401 Australia)	Australia	2008	31±4	6	Normal, 10	50	116.3	Glucose, 2h	Standard	Capillary, plasma	Enzymatic	UO[5]
402	Muesli (Value, UK)	UK	2005	64±9	13	Normal, 10	50	79.9	Glucose, 2h	Standard	Capillary, whole blood	Glucometer[9]	26
403	Muesli (Healthy Eating, UK)	UK	2005	86±10	17	Normal, 10	50	70.3	Glucose, 2h	Standard	Capillary, whole blood	Glucometer[9]	26
404	Muesli (Cereali croccanti classic, Italy)[6]	Italy	2009	66±6	13	Normal, 10	50	75.5	Glucose, 2h	Standard	Capillary, whole blood	YSI	3

Food Number and Item		Country of food production	Year of test[1]	GI[2] ± SEM (Glu = 100)	GL	Subjects (type & number)	Avail carb (Test portion)	Test portion[3] (g)	Reference food & time period	Timepoints	Sample collection	Sample analysis method[4]	Ref.
405	Muesli, fruit	UK	2005	67±7	13	Normal, 10	50	74.4	Glucose, 2h	Standard	Capillary, whole blood	Glucometer[9]	26
406	Muesli, with concentrated fruit juices (Cereali croccanti classic, Italy)[6]	Italy	2009	62±8	12	Normal, 10	50	69.9	Glucose, 2h	Standard	Capillary, whole blood	YSI	3
407	Muesli Gluten Free, Carman's Deluxe (Carman's Fine Foods, Australia)	Australia	2014	49±3	10	Normal, 10	50	79.0	Glucose, 2h	Standard	Capillary, plasma	Enzymatic	UO[5]
408	Muesli, gluten-free with psyllium (Freedom Foods, Australia)	Australia	2007	50±7	10	Normal, 9	50	150.2	Glucose, 2h	Standard	Capillary, whole blood	HemoCue	UO[10]
409	Muesli, Light, mixed berry & apple flavor, Special K brand (Kellogg's, Australia)	Australia	2007	64±7	13	Normal, 10	50	74.5	Glucose, 2h	Standard	Capillary, whole blood	HemoCue	UO[5]
410	Muesli, made from steamed rolled oats with dried fruit and nuts, Alpen original (Weetabix, UK)	UK	2002	55±10	11	Normal, 11-14	50	80	Glucose, 2h	Standard	Capillary, plasma	Enzymatic	53
411	Muesli, Morning Sun Natural Apricot & Almond (Nestlé, Australia)	Australia	2006	49±5	10	Normal, 10	25	56.6	Glucose, 2h	Standard	Capillary, whole blood	HemoCue	UO[5]
412	Muesli, Natural (Sanitarium, Australia)	Australia	2001	40±6	8	Normal, 10	50	79.1	Glucose, 2h	Standard	Capillary, plasma	Enzymatic	UO[5]
413	Muesli, Natural (Sanitarium, New Zealand)	New Zealand	2000	57±9	11	Normal, 10	50	NS	Glucose, 2h	Standard	Venous, plasma	Enzymatic	27
414	Muesli, Natural Style Original Swiss Formula (Uncle Toby's, Australia)	Australia	2007	62±6	12	Normal, 9	50	83.8	Glucose, 2h	Standard	Capillary, whole blood	HemoCue	UO[5]
415	Muesli, Naytura Fruit and Nut (Woolworths, Australia)	Australia	2007	48±6	10	Normal, 10	50	108.0	Glucose, 2h	Standard	Capillary, whole blood	HemoCue	UO[5]
416	Muesli, Swiss Bircher (Woolworths Select, Australia)	Australia	2008	52±5	10	Normal, 10	50	85.0	Glucose, 2h	Standard	Capillary, plasma	Enzymatic	UO[5]
417	Muesli, toasted, with nuts	Australia	2006	65±5	13	Normal, 10	50	NS	Glucose, 2h	Standard	Capillary, plasma	Enzymatic	UO[5]
418	Muesli, wholewheat	UK	2005	56±6	11	Normal, 10	50	84.6	Glucose, 2h	Standard	Capillary, whole blood	Glucometer[9]	26

419	Muesli, yeast & wheat free (Freedom Foods, Australia)	Australia	2007	45±5	9	Normal, 10	50	150.2	Glucose, 2h	Standard	Capillary, whole blood	HemoCue	UO[10]
420	Rye Muesli, Bürgen® (George Weston Foods, Australia)	Australia	2007	41±5	8	Normal, 10	50	NS	Glucose, 2h	Standard	Capillary, whole blood	HemoCue	UO15
421	Soy-Lin™ Muesli, Bürgen® (George Weston Foods, Australia)	Australia	2006-2007	51±3	10	Normal, 10	50	NS	Glucose, 2h	Standard	Capillary, whole blood	HemoCue	UO[10]
422	Whole-grain oat muesli	USA	2018*	55±4	11	Normal, 19	50	84	Glucose, 2h	Standard	Capillary, whole blood	HemoCue	57
	Muesli, mean of 30 foods			55									
	**Muesli, high protein, low carbohydrate**												
423	Chocolate Protein Muesli (Herman Brot Pty Ltd, Australia)	Australia	2016	35±4	7	Normal, 10	25	120.8	Glucose, 2h	Standard	Capillary, plasma	Enzymatic	UO[5]
424	Peanut Candy Protein Muesli (Herman Brot Pty Ltd, Australia)	Australia	2016	30±3	6	Normal, 10	25	147.1	Glucose, 2h	Standard	Capillary, plasma	Enzymatic	UO[5]
425	Red Fruits Protein Muesli (Herman Brot Pty Ltd, Australia)	Australia	2016	32±4	6	Normal, 10	25	134.4	Glucose, 2h	Standard	Capillary, plasma	Enzymatic	UO[5]
	**Porridge / oatmeal**												
	**Instant oat porridge**												
426	Instant oats, cooked in microwave for 2.5 min (Quaker Quick Oats; Peterborough, Ontario)	Canada	2010	76±4	15	Normal, 10	50	88.2	Glucose, 2h	Standard	Capillary, plasma	Enzymatic	39
427	Instant oat porridge, cooked in microwave with water (Uncle Toby's, Australia)	Australia	2003	82±10	16	Normal, 10	50	88.2	Glucose, 2h	Standard	Capillary, whole blood	HemoCue	UO[5]
428	Instant oat cereal porridge, prepared with 348 g water	UK	2006	83±10	17	Normal, 10	50	87.1	Glucose, 2h	Standard	Capillary, whole blood	Glucometer[9]	30
429	Instant oat porridge, prepared with water, cooked	Australia	2007	87±9	17	Normal, 10	50	88.2	Glucose, 2h	Standard	Capillary, plasma	Enzymatic	UO[5]
	Instant oat porridge, mean of four foods			82									
	**Porridge, made from rolled oats**												
430	Porridge oats (Freedom Foods, Australia)	Australia	2007	50±4	10	Normal, 12	50	80.9	Glucose, 2h	Standard	Capillary, whole blood	HemoCue	UO[10]
431	Porridge oats, traditional (Lowan Whole Foods, Australia)	Australia	2004	51±8	10	Normal, 10	50	81.0	Bread, 2h	Standard	Capillary, whole blood	HemoCue	UO[5]

432	Porridge oats	China	2005	55±2	11	Normal, 9	50	NS	Glucose, 2h	Standard	Venous, NS	NS	12

Food Number and Item		Country of food production	Year of test[1]	GI[2] ± SEM (Glu = 100)	GL	Subjects (type & number)	Avail carb (Test portion)	Test portion[3] (g)	Reference food & time period	Timepoints	Sample collection	Sample analysis method[4]	Ref.
433	Porridge, Traditional Rolled Oats (Woolworths Select brand, Australia)	Australia	2008	57±4	11	Normal, 10	50	81.4	Glucose, 2h	Standard	Capillary, plasma	Enzymatic	UO[5]
434	Porridge (Hubbards, New Zealand)	New Zealand	2000	58±9	12	Normal, 10	50	NS	Glucose, 2h	Standard	Venous, plasma	Enzymatic	27
435	Porridge, Scottish, made with water, cooked in microwave	UK	2005	63±7	13	Normal, 10	50	80.6	Glucose, 2h	Standard	Capillary, whole blood	Glucometer[9]	26
436	Oat porridge made from 0.5-0.6 mm thick rolled oat flakes, cooked for 10 min (Elovena, Raisio Group Ltd, Raisio, Finland)	Finland	2005	76±7	15	Normal, 12	50	495	Glucose, 3h[21]	0,15,30,45,60,90,120,180	Capillary, whole blood	HemoCue	59
437	Multigrain porridge, containing rolled oats, wheat, triticale, rye, barley and rice, cooked with water (The Monster Muesli Company, Beecroft, Australia)	Australia	2003	55±2	11	Normal, 9	50	143.8	Glucose, 2h	Standard	Capillary, whole blood	HemoCue	UO[5]
	Porridge made from rolled oats, mean of eight foods			58									
	**Porridge, made from steel-cut oats**												
438	Porridge, made from steel-cut oats, cooked in water	Australia	2003	48±5	10	Normal, 10	50	81.2	Glucose, 2h	Standard	Capillary, plasma	Enzymatic	UO[5]
439	Porridge, made from steel-cut oats, Moulamein™, cooked in water	Australia	2016	51±5	10	Normal, 9	50	87.9	Glucose, 2h	Standard	Capillary, plasma	Enzymatic	UO[5]
440	Porridge, made from steel-cut oats, cooked in water	USA	2003	52±4	10	Normal, 9	50	90.9	Glucose, 2h	Standard	Capillary, whole blood	HemoCue	UO[5]
441	Porridge, made from steel-cut oats, cooked in water (Meebags, Sunny International Trading Pty Ltd)	Australia	2019	53±3	11	Normal, 9	50	89.6	Glucose, 2h	Standard	Capillary, plasma	Enzymatic	UO[5]
442	Porridge, made from old-Fashioned Steel Cut Oats, President's Choice® Blue Menu™(Loblaw Brands Limited, Canada)	Canada	2007	57±8	11	Normal, 10	50	80.0	Bread, 2h	Standard	Capillary, whole blood	YSI	UO[7]
	Porridge made from steel-cut oats, mean of five foods			52									

	**Porridge, made from rolled oats, flavored**											
443	Almond & Cinnamon, Uncle Toby's® Oats Super Blends Protein, prepared with water	Australia	2019	57±5	11	Normal, 10	50	105.3	Glucose, 2h	Standard	Capillary, plasma	Enzymatic	UO[5]
444	Cranberry & Chia, Uncle Toby's® Oats Super Blends Protein, prepared with water	Australia	2019	52±5	10	Normal, 10	50	94.3	Glucose, 2h	Standard	Capillary, plasma	Enzymatic	UO[5]
445	Multi grain instant oatmeal, Regular and Cinnamon & Spice, President's Choice® Blue Menu™ Loblaw Brands Limited, Canada)	Canada	2007	55±7	11	Normal, 10	50	86.5	Bread, 2h	Standard	Capillary, whole blood	YSI	UO[7]

Food Number and Item		Country of food production	Year of test[1]	GI[2] ± SEM (Glu = 100)	GL	Subjects (type & number)	Avail carb (Test portion)	Test portion[3] (g)	Reference food & time period	Timepoints	Sample collection	Sample analysis method[4]	Ref.
	**Rice Bubbles**												
446	Rice Bubbles™ (Kellogg's, Australia)	Australia	2000	85±3	17	Normal, 10	50	58.5	Glucose, 2h	Standard	Capillary, plasma	Enzymatic	UO[5]
447	Rice Bubbles™ (Kellogg's, Australia)	Australia	2007	92±8	18	Normal, 9	50	58.4	Glucose, 2h	Standard	Capillary, plasma	Enzymatic	UO[5]
	**Special K™** - *formulation of this cereal varies in different countries*												
448	Special K™ (Kellogg's, USA)	USA	2000	69±5	14	Normal, 10	50	NS	Bread, 2h	Standard	Capillary, plasma	Enzymatic	UO[5]
449	Special K™, made from rice (Kellogg's, France)	France	2002	84±12	17	Normal, 11-14	50	64	Glucose, 2h	Standard	Capillary, plasma	Enzymatic	53
	**Sustain**												
450	Sustain™, Original (Kellogg's, Australia)	Australia	2007	55±5	11	Normal, 10	50	67.4	Glucose, 2h	Standard	Capillary, plasma	Enzymatic	UO[5]
451	Sustain™ (Kellogg's)[13], tested in younger adults (19-32 y)	Australia	2014*	56±4	11	Normal, 20	50	NS	Glucose, 2h	Standard	Capillary, whole blood	HemoCue	55
452	Sustain™ (Kellogg's)[13], tested in older adults (56–86 y)	Australia	2014*	66±3	13	Normal, 60	50	NS	Glucose, 2h	Standard	Capillary, whole blood	HemoCue	55
	Sustain, mean of three studies			59									
	**Wheat biscuits (plain flaked wheat)**												
453	Vita-Brits™ (Uncle Toby's, Australia)	Australia	2001	68±6	14	Normal, 10	50	75.6	Glucose, 2h	Standard	Capillary, whole blood	HemoCue	UO[5]

454	Weet-Bix™ (Sanitarium, Australia)	Australia	2004	69±4	14	Normal, 10	50	78.2	Glucose, 2h	Standard	Capillary, whole blood	HemoCue	UO[5]
455	Weet-Bix™ (Sanitarium, Australia)	Australia	2001	69±4	14	Normal, 12	50	75.1	Bread, 2h	Standard	Capillary, whole blood	HemoCue	UO[5]
456	Whole wheat Goldies™ (Kellogg's, Australia)	Australia	2001	70±4	14	Normal, 10	50	74.5	Glucose, 2h	Standard	Capillary, whole blood	HemoCue	UO[5]
457	Wheat based cereal biscuits	UK	2006	72±10	14	Normal, 10	50	74.5	Glucose, 2h	Standard	Capillary, whole blood	Glucometer[9]	30
458	Wheat flake cereal biscuits	Australia	2014*	76±5	15	Normal 10	50	75	Glucose, 2h	Standard	Capillary, plasma	Enzymatic	17
	Wheat biscuits plain, mean of six foods			71									

Food Number and Item		Country of food production	Year of test[1]	GI[2] ± SEM (Glu = 100)	GL	Subjects (type & number)	Avail carb (Test portion)	Test portion[3] (g)	Reference food & time period	Timepoints	Sample collection	Sample analysis method[4]	Ref.
	**Wheat biscuits (flaked wheat) with additional ingredients**												
459	Good Start™, muesli wheat biscuits (Sanitarium, Australia)	Australia	2001	68±4	14	Normal, 10	50	75.8	Glucose, 2h	Standard	Capillary, whole blood	HemoCue	UO[5]
460	Hi-Bran Weet-Bix™, wheat biscuits with extra wheat bran (Sanitarium, Australia)	Australia	2001	61±4	12	Normal, 10	50	147.4	Glucose, 2h	Standard	Capillary, whole blood	HemoCue	UO[5]
461	Hi-Bran Weet-Bix™ with soy and linseed (Sanitarium, Australia)	Australia	2001	57±3	11	Normal, 10	50	92.3	Glucose, 2h	Standard	Capillary, whole blood	HemoCue	UO[5]
462	Honey Goldies™ (Kellogg's Australia)	Australia	2001	72±3	14	Normal, 10	50	71.3	Glucose, 2h	Standard	Capillary, whole blood	HemoCue	UO[5]
463	Lite-Bix™, plain, no added sugar (Sanitarium, Australia)	Australia	2001	70±3	14	Normal, 10	50	92.9	Glucose, 2h	Standard	Capillary, whole blood	HemoCue	UO[5]
464	Oat bran Weet-Bix™ (Sanitarium, Australia)	Australia	2001	57±4	11	Normal, 10	50	91.4	Glucose, 2h	Standard	Capillary, whole blood	HemoCue	UO[5]
465	Sultana Goldies™ (Kellogg's Australia)	Australia	2001	65±6	13	Normal, 10	50	73.1	Glucose, 2h	Standard	Capillary, whole blood	HemoCue	UO[5]
466	Wheat flake cereal biscuits with 1.4 g Filtered Molasses Concentrate added per 100 g (2.08 g FMC/100 g carbohydrate)	Australia	2014*	72±5	14	Normal 10	50	75	Glucose, 2h	Standard	Capillary, plasma	Enzymatic	17

Food Number and Item		Country of food production	Year of test[1]	GI[2] ± SEM (Glu = 100)	GL	Subjects (type & number)	Avail carb (Test portion)	Test portion[3] (g)	Reference food & time period	Timepoints	Sample collection	Sample analysis method[4]	Ref.
467	Wheat flake cereal biscuits with 2.0 g Filtered Molasses Concentrate added per 100g (2.98 g FMC/100 g carbohydrate)	Australia	2014*	70±4	14	Normal 10	50	75	Glucose, 2h	Standard	Capillary, plasma	Enzymatic	17
468	Wheat flakes	Australia	2019	73±7	15	Normal 10	50	85.6	Glucose, 2h	Standard	Capillary, plasma	Enzymatic	UO[5]
	**Other breakfast cereals**												
469	All-Bran Fruit 'n Oats™ (Kellogg's, Australia)	Australia	1997	41±9	8	Normal, 10-12	50	NS	Bread, 2h	Standard	Capillary, plasma	Enzymatic	25
470	All-Bran Soy 'n Fiber™ (Kellogg's, Australia)	Australia	1999	33±3	7	Normal, 10	50	88.0	Bread, 2h	Standard	Capillary, plasma	Enzymatic	UO[5]
471	All-Bran Wheat Flakes™ (Kellogg's, Australia)	Australia	2007	60±8	12	Normal, 9	50	72.6	Glucose, 2h	Standard	Capillary, plasma	Enzymatic	UO[5]
472	Bran cereal, high fiber	UK	2005	43±10	9	Normal, 10	50	129.9	Glucose, 2h	Standard	Capillary, whole blood	Glucometer[9]	26
473	Coco Pops™ (Kellogg's, Australia)	Australia	2000	77±3	15	Normal, 10	50	57.1	Glucose, 2h	Standard	Capillary, plasma	Enzymatic	UO[5]
474	Corn Pops™ (Kellogg's, Australia)	Australia	2000	80±4	16	Normal, 10	50	55.6	Glucose, 2h	Standard	Capillary, plasma	Enzymatic	UO[5]
475	Crunchola Apple & Cinnamon Oat & Fruit (Norganic Foods Pty Ltd, Australia)	Australia	2008	70±6	14	Normal, 10	50	69.1	Glucose, 2h	Standard	Capillary, plasma	Enzymatic	UO[5]
476	Energy Mix™, wheat-based flaked cereal (Quaker, France)	France	2002	80±7	16	Normal, 11-14	50	58	Glucose, 2h	Standard	Capillary, plasma	Enzymatic	53
477	Fibre First Multi-Bran Cereal, President's Choice® Blue Menu™ (Loblaw Brands Limited, Canada)	Canada	2004-2007	56±10	11	Normal, 10	50	150.2	Bread, 2h	Standard	Capillary, whole blood	YSI	UO[7]
478	Froot Loops™ (Kellogg's, Australia)	Australia	1999	69±9	14	Normal, 10	50	58.6	Bread, 2h	Standard	Capillary, plasma	Enzymatic	UO[5]
479	Frosties™, sugar-coated cornflakes (Kellogg's, Australia)	Australia	1997	55±7	11	Normal, 12	50	57.8	Bread, 2h	Standard	Capillary, plasma	Enzymatic	UO[5]
480	Fruity-Bix™, berry (Sanitarium, New Zealand)	New Zealand	2000	113±10	23	Normal, 10	50	NS	Glucose, 2h	Standard	Venous, plasma	Enzymatic	27

Food Number and Item		Country of food production	Year of test[1]	GI[2] ± SEM (Glu = 100)	GL	Subjects (type & number)	Avail carb (Test portion)	Test portion[3] (g)	Reference food & time period	Timepoints	Sample collection	Sample analysis method[4]	Ref.
481	Golden Wheats™ (Kellogg's, Australia)	Australia	1997	71±8	14	Normal, 10	50	65.2	Bread, 2h	Standard	Capillary, plasma	Enzymatic	UO[5]
482	Goodness Superfoods Fibre Boost Sprinkles (Popina Pty Ltd, Australia)	Australia	2009	34±4	7	Normal, 10	25	40.0	Glucose, 2h	Standard	Capillary, plasma	Enzymatic	UO[5]
483	Grapenuts™ (Kraft Foods Inc, Port Chester, USA)	USA	1997	75±6	15	Normal, 10	50	72.5	Bread, 2h	Standard	Capillary, plasma	Enzymatic	UO[5]
484	Guardian™ (Kellogg's, Australia)	Australia	1997	37±9	7	Normal, 10-12	50	NS	Bread, 2h	Standard	Capillary, plasma	Enzymatic	25
485	Guardian™ (Kellogg's, Australia)	Australia	2019	43±4	9	Normal, 11	50	38.3	Glucose, 2h	Standard	Capillary, plasma	Enzymatic	UO[5]
486	Healthwise™ for bowel health (Uncle Toby's, Wahgunyah, Australia)	Australia	2006	66±9	13	Normal, 10	50	72.7	Glucose, 2h	Standard	Capillary, whole blood	HemoCue	UO[5]
487	High-fiber cereal	UK	2006	52±6	10	Normal, 10	50	87.1	Glucose, 2h	Standard	Capillary, whole blood	Glucometer[9]	30
488	Hi-Lite™, containing 55% rolled barley grains (Freedom Foods, Cheltenham, Australia)	Australia	2006-2007	54±5	11	Normal, 9	50	82.1	Glucose, 2h	Standard	Capillary, whole blood	HemoCue	UO[10]
489	Honey Rice Bubbles™ (Kellogg's, Australia)	Australia	2000	77±4	15	Normal, 10	50	56.4	Glucose, 2h	Standard	Capillary, plasma	Enzymatic	UO[5]
490	Honey Smacks™ (Kellogg's, Australia)	Australia	1997	71±10	14	Normal, 10-12	50	NS	Bread, 2h	Standard	Capillary, plasma	Enzymatic	25
491	Hot cereal, Sustagrain, apple & cinnamon (Con Agra Inc, USA)	USA	1999	37±6	7	Normal, 10	50	96.3	Bread, 2h	Standard	Capillary, plasma	Enzymatic	UO[5]
492	Hot cereal, Sustagrain, unflavored (Con Agra Inc., USA)	USA	1999	25±5	5	Normal, 10	50	135.5	Bread, 2h	Standard	Capillary, plasma	Enzymatic	UO[5]
493	Just Right™ (Kellogg's, Australia)	Australia	1997	60±15	12	Normal, 10-12	50	NS	Bread, 2h	Standard	Capillary, plasma	Enzymatic	25
494	Just Right Just Grains™ (Kellogg's, Australia)	Australia	1999	62±11	12	Normal, 10	50	64.3	Bread, 2h	Standard	Capillary, plasma	Enzymatic	UO[5]

495	Komplete™ (Kellogg's, Australia)	Australia	1997	48±5	10	Normal, 10	50	NS	Bread, 2h	Standard	Capillary, plasma	Enzymatic	UO[5]
496	Milo® Protein Clusters Cereal (Nestlé, Australia)	Australia	2015	47±4	9	Normal, 10	50	80.9	Glucose, 2h	Standard	Capillary, plasma	Enzymatic	UO[5]
497	Mini Wheats™, blackcurrant (Kellogg's, Australia)	Australia	1997	72±10	14	Normal, 10-12	50	NS	Bread, 2h	Standard	Capillary, plasma	Enzymatic	25
498	Oat 'n Honey Bake™ (Kellogg's, Australia)	Australia	1999	77±11	15	Normal, 10	50	NS	Bread, 2h	Standard	Capillary, whole blood	HemoCue	UO[5]
499	Oats, rolled, uncooked (Lowan's Whole Foods, Box Hill, Australia)	Australia	2003	59±4	12	Normal, 10	50	80.9	Glucose, 2h	Standard	Capillary, whole blood	HemoCue	UO[5]
500	Raisin Bran™ (Kellogg's, USA)	USA	2000	61±5	12	Normal, 10	50	NS	Bread, 2h	Standard	Capillary, whole blood	HemoCue	UO[5]
501	Soy Tasty™ (flaked grains, soy nuts, dried fruit) (Sanitarium, Australia)	Australia	2001	60±5	12	Normal, 10	50	77.6	Glucose, 2h	Standard	Capillary, whole blood	HemoCue	UO[5]
502	Sultana Bran™ (Kellogg's, Australia)	Australia	2007	64±3	13	Normal, 9	50	78.6	Glucose, 2h	Standard	Capillary, plasma	Enzymatic	UO[5]
503	Ultra-bran™, Vogel's, soy and linseed extruded wheat bran cereal, 30.2 g fiber/100 g (Specialty Cereals, Australia)	Australia	2001	41±4	8	Normal, 10	50	104.6	Glucose, 2h	Standard	Capillary, whole blood	HemoCue	UO[5]
504	Wheat flakes	Australia	2019	73±7	15	Normal, 10	50	85.6	Glucose, 2h	Standard	Capillary, plasma	Enzymatic	UO[5]
505	Whole-grain protein cereal	USA	2018*	49±3	10	Normal, 19	50	99	Glucose, 2h	Standard	Capillary, whole blood	HemoCue	57
	**Breakfast cereal bars and cookies**												
	**Breakfast cereal bars**												
506	Apricot Breakfast Bar, chewy (Norganic Foods Pty Ltd, Australia)	Australia	2008	85±10	17	Normal, 10	50	74.7	Glucose, 2h	Standard	Capillary, plasma	Enzymatic	UO[5]
507	Cereal bar, cranberry flavor	UK	2006	42±5	8	Normal, 10	50	103.1	Glucose, 2h	Standard	Capillary, whole blood	Glucometer[9]	30
508	Cereal bar, hazelnut flavor	UK	2006	33±6	7	Normal, 10	50	103.7	Glucose, 2h	Standard	Capillary, whole blood	Glucometer[9]	30

Food Number and Item		Country of food production	Year of test[1]	GI[2] ± SEM (Glu = 100)	GL	Subjects (type & number)	Avail carb (Test portion)	Test portion[3] (g)	Reference food & time period	Timepoints	Sample collection	Sample analysis method[4]	Ref.
509	Cereal bar, orange flavor	UK	2006	33±3	7	Normal, 10	50	105.5	Glucose, 2h	Standard	Capillary, whole blood	Glucometer[9]	30
510	Crunchy Nut Cornflakes™ bar (Kellogg's, Australia)	Australia	1999	72±6	14	Normal, 10	50	58.3	Bread, 2h	Standard	Capillary, plasma	Enzymatic	UO[5]
511	Fruity-Bix™ bar, wheat biscuit cereal with dried fruit and nuts with yoghurt coating (Sanitarium, Australia)	Australia	2001	56±4	11	Normal, 10	50	79.0	Glucose, 2h	Standard	Capillary, plasma	Enzymatic	UO[5]
512	Fruity-Bix™ bar, wild berry, wheat biscuit cereal and dried fruit covered with yoghurt coating (Sanitarium, Australia)	Australia	2001	51±4	10	Normal, 10	50	82.4	Glucose, 2h	Standard	Capillary, plasma	Enzymatic	UO[5]
513	Hi-Lite™ breakfast bar (Freedom Foods, Australia)	Australia	2007	53±3	11	Normal, 10	50	71.6	Glucose, 2h	Standard	Capillary, whole blood	HemoCue	UO[10]
514	K-Time Just Right™ bar (Kellogg's, Australia)	Australia	2000	72±4	14	Normal, 10	50	64.3	Glucose, 2h	Standard	Capillary, plasma	Enzymatic	UO[5]
515	K-Time Strawberry Crunch™ bar (Kellogg's, Australia)	Australia	2000	77±5	15	Normal, 10	50	61.7	Glucose, 2h	Standard	Capillary, plasma	Enzymatic	UO[5]
516	Muesli breakfast bar, gluten-free (Freedom Foods, Australia)	Australia	2007	50±6	10	Normal, 9	50	87.9	Glucose, 2h	Standard	Capillary, whole blood	HemoCue	UO[10]
517	Rice Bubble Treat™ bar (Kellogg's, Australia)	Australia	1999	63±11	13	Normal, 10	50	63.2	Bread, 2h	Standard	Capillary, plasma	Enzymatic	UO[5]
518	Sustain™ bar (Kellogg's, Australia)	Australia	1999	57±10	11	Normal, 10	50	60.8	Bread, 2h	Standard	Capillary, plasma	Enzymatic	UO[5]
519	Uncle Toby's® Milk and Oats, Chocolate cereal bar	Australia	2019	48±3	10	Normal, 10	50	93.8	Glucose, 2h	Standard	Capillary, plasma	Enzymatic	UO[5]
520	Uncle Toby's® Milk and Oats, Strawberry cereal bar	Australia	2018	50±4	10	Normal, 10	50	94.2	Glucose, 2h	Standard	Capillary, plasma	Enzymatic	UO[5]
521	Uncle Toby's® Milk and Oats, Vanilla cereal bar	Australia	2019	46±4	9	Normal, 9	50	93.6	Glucose, 2h	Standard	Capillary, plasma	Enzymatic	UO[5]
	Breakfast cereal bars, mean of 16 foods			56									

	**Breakfast cereal cookies**												
522	Uncle Toby's® Breakfast Bakes, Apple and Cinnamon	Australia	2019	45±5	9	Normal, 9	50	95.8	Glucose, 2h	Standard	Capillary, plasma	Enzymatic	UO[5]
523	Uncle Toby's® Breakfast Bakes, Classic Peanut Butter	Australia	2019	47±5	9	Normal, 10	50	102.7	Glucose, 2h	Standard	Capillary, plasma	Enzymatic	UO[5]
524	Uncle Toby's® Breakfast Bakes, Honey Almond	Australia	2019	50±4	10	Normal, 9	50	101.2	Glucose, 2h	Standard	Capillary, plasma	Enzymatic	UO[5]

Food Number and Item		Country of food production	Year of test[1]	GI[2] ± SEM (Glu = 100)	GL	Subjects number)(type &	Avail carb (Test portion)	Test portion[3] (g)	Reference food & time period	Timepoints	Sample collection	Sample analysis method[4]	Ref.
525	Uncle Toby's® Breakfast Bakes, Vanilla & Roasted Cashew	Australia	2019	44±3	9	Normal, 9	50	101.8	Glucose, 2h	Standard	Capillary, plasma	Enzymatic	UO[5]
	Breakfast cereal cookies, mean of four foods			47									
	**CEREAL GRAINS**												
	Average available carbohydrate portion = 45 g, this value was used to determine the nominal GL for each item in this category.												
	**Adlay/Chinese pearl barley (Coix lachryma-Jobi)**												
526	Adlay (Coix lachryma-jobi L)[22]	Taiwan	2010*	39±0.3	18	Normal, 10	50	64	Bread, 2h	Standard	Capillary, whole blood	Enzymatic	60
527	Adlay, Chinese pearl barley (Coix lachryma-Jobi L.), boiled in water for 28 min	Australia	2005	78±6	35	Normal, 10	50	81.7	Glucose, 2h	Standard	Capillary, plasma	Enzymatic	UO[5]
	**Barley**												
528	Barley, Celebrity cultivar (six-rowed, hulled, normal barley; high amylose, medium ß-glucan), whole grain (only the husk was removed), boiled for 25 min	Canada	2012*	21±4	9	Normal, 10	50	92.2	Bread, 2h	Standard	Capillary, whole blood	YSI	61
529	Barley, Celebrity cultivar (six-rowed, hulled, normal barley; high amylose, medium ß-glucan), pot pearled (all of the bran and most of the germ removed), boiled for 30 min	Canada	2012*	22±3	10	Normal, 10	50	83.7	Bread, 2h	Standard	Capillary, whole blood	YSI	61
530	Barley, CDC Fibar cultivar (two-rowed, hull-less, waxy barley; low amylose, high ß-glucan) whole grain (only the husk was removed), boiled for 25 min	Canada	2012*	22±4	10	Normal, 10	50	102.2	Bread, 2h	Standard	Capillary, whole blood	YSI	61

Food Number and Item		Country of food production	Year of test[1]	GI[2] ± SEM (Glu = 100)	GL	Subjects (type & number)	Avail carb (Test portion)	Test portion[3] (g)	Reference food & time period	Timepoints	Sample collection	Sample analysis method[4]	Ref.
531	Barley, GB cultivar (two-rowed, hulled, normal barley), whole grain (only the husk was removed), boiled for 25 min	Canada	2012*	24±5	11	Normal, 10	50	89.5	Bread, 2h	Standard	Capillary, whole blood	YSI	61
532	Barley, pot, boiled in salted water 20 min (Goudas Food Products, Concord, Canada)	Canada	1999	25±2	11	Normal, 10	50	79.6	Glucose, 2h	Standard	Capillary, plasma	Enzymatic	UO[5]
533	Barley, Celebrity cultivar (six-rowed, hulled, normal barley; high amylose, medium ß-glucan), commercial pearled (some bran and germ were also removed), boiled for 25 min	Canada	2012*	25±3	11	Normal, 10	50	91.6	Bread, 2h	Standard	Capillary, whole blood	YSI	61
534	Barley, Celebrity cultivar (six-rowed, hulled, normal barley; high amylose, medium ß-glucan) whole grain (only the husk was removed), boiled for 25 min	Canada	2012*	25±4	11	Normal, 10	50	92.2	Bread, 2h	Standard	Capillary, whole blood	YSI	61
535	Barley, Rattan cultivar (two-rowed, hull-less, waxy barley), whole grain (only the husk was removed), boiled for 25 min	Canada	2012*	26±6	12	Normal, 10	50	95.3	Bread, 2h	Standard	Capillary, whole blood	YSI	61
536	Barley, Kawathra cultivar (six-rowed, hulled, normal barley), whole grain (only the husk was removed), boiled for 25 min	Canada	2012*	28±4	13	Normal, 10	50	85.1	Bread, 2h	Standard	Capillary, whole blood	YSI	61
537	Barley, Chief cultivar (two-rowed, hulled, normal barley), whole grain (only the husk was removed), boiled for 25 min	Canada	2012*	29±4	13	Normal, 10	50	83.6	Bread, 2h	Standard	Capillary, whole blood	YSI	61
538	Barley, AC Alberta cultivar (two-rowed, hull-less, normal barley), whole grain (only the husk was removed), boiled for 25 min	Canada	2012*	29±7	13	Normal, 10	50	91.2	Bread, 2h	Standard	Capillary, whole blood	YSI	61
539	Barley, CDC Fibar cultivar (two-rowed, hull-less, waxy barley; low amylose, high ß-glucan) white pearled (all of the bran and most of the germ and crease removed), boiled for 30 min	Canada	2012*	30±3	14	Normal, 10	50	86.9	Bread, 2h	Standard	Capillary, whole blood	YSI	61
540	Barley, AC Parkhill cultivar (two-rowed, hulled, normal barley; high amylose, low ß-glucan) whole grain (only the husk was removed), boiled for 25 min	Canada	2012*	30±5	14	Normal, 10	50	87.6	Bread, 2h	Standard	Capillary, whole blood	YSI	61
541	Barley, Celebrity cultivar (six-rowed, hulled, normal barley; high amylose, medium ß-glucan), white pearled (all of the bran and most of the germ and crease removed), boiled for 30 min	Canada	2012*	32±6	14	Normal, 10	50	80.2	Bread, 2h	Standard	Capillary, whole blood	YSI	61

Food Number and Item		Country of food production	Year of test[1]	GI[2] ± SEM (Glu = 100)	GL	Subjects (type & number)	Avail carb (Test portion)	Test portion[3] (g)	Reference food & time period	Timepoints	Sample collection	Sample analysis method[4]	Ref.
542	Barley, Celebrity cultivar (six-rowed, hulled, normal barley; high amylose, medium ß-glucan) white pearled (all of the bran and most of the germ and crease removed), boiled for 30 min	Canada	2012*	33±3	15	Normal, 10	50	80.2	Bread, 2h	Standard	Capillary, whole blood	YSI	61
543	Barley, pot, boiled (Goudas Food Products, Canada)	Canada	2001	35±4	16	Normal, 47	50	NS	Glucose, 2h	Standard	Capillary, NS	YSI or Enzymatic	42
544	Barley, pot, boiled (Goudas Food Products, Canada)	Canada	2001	33±8	15	Normal, 21	50	NS	Glucose, 2h	Standard	Venous, NS	Enzymatic	42
545	Barley, AC Klinck cultivar (six-rowed, hulled, normal barley), whole grain (only the husk was removed), boiled for 25 min	Canada	2012*	36±8	16	Normal, 10	50	85.8	Bread, 2h	Standard	Capillary, whole blood	YSI	61
546	Barley, AC Parkhill cultivar (two-rowed, hulled, normal barley; high amylose, low ß-glucan) white pearled (all of the bran and most of the germ and crease removed), boiled for 30 min	Canada	2012*	41±5	18	Normal, 10	50	76.1	Bread, 2h	Standard	Capillary, whole blood	YSI	61
547	Barley, pearled (Orzo, Barilla, Italy)	Italy	2009	58±8	26	Normal, 9	50	76	Glucose, 2h	Standard	Capillary, whole blood	YSI	3
	Barley, mean of 20 foods			30									
548	Barley tempe (prepared by Lantmännen R&D from whole barley grains (c.v. Karmosé) with high ß-glucan (6%) and high amylose content (~40%) from Svalöf Weibull AB, Sweden)	Sweden	2007	30±5	14	Normal, 13	25	95	Glucose, 2h	Standard	Capillary, whole blood	HemoCue	62
549	Highland Barley Mixed Flour (20% Highland Barley flour, 80% wheat flour) (Xinwang (Tibet XinWang Biology & Technology, China)	China	2019	63±5	28	Normal, 12	50	71.4	Glucose, 2h	Standard	Capillary, plasma	Enzymatic	UO[18]
550	Porridge made from pearl barley (15.6 g fiber/100 g) (Waitrose Limited, Bracknell, UK)	UK	2012*	44±7	20	Normal, 10	25	NS	Glucose, 2h	Standard	Capillary, whole blood	HemoCue	63
551	Porridge made from pearl barley (15.6 g fiber/100 g) (Waitrose Limited, Bracknell, UK)	UK	2012*	39±10	18	Normal, 10	50	NS	Glucose, 2h	Standard	Capillary, whole blood	HemoCue	63
552	Porridge made from organic barley grain (9.6 g fiber/100 g) (Suma Whole Foods, UK)	UK	2012*	50±5	23	Normal, 10	25	NS	Glucose, 2h	Standard	Capillary, whole blood	HemoCue	63

Food Number and Item		Country of food production	Year of test[1]	GI[2] ± SEM (Glu = 100)	GL	Subjects (type & number)	Avail carb (Test portion)	Test portion[3] (g)	Reference food & time period	Timepoints	Sample collection	Sample analysis method[4]	Ref.
553	Porridge made from organic barley grain (9.6 g fiber/100 g) (Suma Whole Foods, UK)	UK	2012*	43±8	19	Normal, 10	50	NS	Glucose, 2h	Standard	Capillary, whole blood	HemoCue	63
	Porridge made from barley, mean of four studies			44									
	**Corn/Maize**												
554	Corn granules	China	2005	52±3	23	Normal, 10	50	NS	Glucose, 2h	Standard	Venous, NS	NS	12
555	Cornmeal porridge	China	2005	68±3	31	Normal, 10	50	NS	Glucose, 2h	Standard	Venous, NS	NS	12
556	100% waxy corn starch (Amioca™ powder, Ingredion), prepared as a gel with 224.2 mL water, heated in microwave for 90 sec	Australia	2016	92±6	41	Normal, 10	25	28.7	Glucose, 2h	Standard	Capillary, plasma	Enzymatic	UO[5]
557	100% waxy corn starch (Ultra-Sperse™powder, Ingredion), prepared as a gel with 224.2 mL water, heated in microwave for 90 sec	Australia	2016	101±7	45	Normal, 10	25	28.7	Glucose, 2h	Standard	Capillary, plasma	Enzymatic	UO[5]
	**Sweet corn**												
558	Sweet corn, cooked in microwave for 1.5 min	Australia	2015	51±4	23	Normal, 10	25	185.2	Glucose, 2h	Standard	Capillary, plasma	Enzymatic	UO[5]
559	Sweet corn, cooked	China	2005	55±2	25	Normal, 10	50	NS	Glucose, 2h	Standard	Venous, NS	NS	12
	**Couscous**												
560	Couscous, rehydrated with hot water (San Remo, Australia)	Australia	2006	65±7	29	Normal, 9	50	70.4	Glucose, 2h	Standard	Capillary, plasma	Enzymatic	UO[5]
561	Pearl couscous (Israeli couscous) (Blu™ Gourmet brand, Benedikts Imports, Australia), rehydrated with hot water	Australia	2008	52±5	23	Normal, 9	50	63.6	Glucose, 2h	Standard	Capillary, plasma	Enzymatic	UO[5]
562	Pearl couscous (Israeli couscous), whole wheat (Blu™ brand, Benedikts Imports, Australia), rehydrated with hot water	Australia	2013	53±4	24	Normal, 9	50	80.6	Glucose, 2h	Standard	Capillary, plasma	Enzymatic	UO[5]

Food Number and Item		Country of food production	Year of test[1]	GI[2] ± SEM (Glu = 100)	GL	Subjects (type & number)	Avail carb (Test portion)	Test portion[3] (g)	Reference food & time period	Timepoints	Sample collection	Sample analysis method[4]	Ref.
	**Millet**												
563	Foxtail millet, cooked (millet:water ratio 1:1.5)	China	2016*	64±9	29	Normal, 10	50	169	Glucose, 2h	Standard	Venous, plasma/serum	Enzymatic	11

564	Unpolished little millet, plain cooked (Earth 360, Kadiri, Andhra Pradesh, India)	India	201718	89±6	40	Normal, 15	50	182.5	Glucose, 2h	Standard	Capillary, whole blood	HemoCue	64
565	Unpolished foxtail millet, plain cooked (Earth 360, Kadiri, Andhra Pradesh, India)	India	201718	89±9	40	Normal, 15	50	170.1	Glucose, 2h	Standard	Capillary, whole blood	HemoCue	64
566	Foxtail millet porridge (millet:water ratio 1:9)	China	2016*	94±11	42	Normal, 10	50	550	Glucose, 2h	Standard	Venous, plasma/serum	Enzymatic	11
	**Oat**												
567	Oat tempe (prepared by Lantmännen R&D from whole oat grains (c.v. Betania) with high ß-glucan content (6%) from Svalöf Weibull AB, Sweden)	Sweden	2007	63±6	28	Normal, 13	25	111	Glucose, 2h	Standard	Capillary, whole blood	HemoCue	62
	**Quinoa**												
568	Quinoa, white, cooked in boiling water for 15 min (Nutritious Foods, Australia)	Australia	2017	50±6	23	Normal, 10	50	74.4	Glucose, 2h	Standard	Capillary, plasma	Enzymatic	UO[5]
569	Quinoa, retort product, Coles Simply Gluten Free Quinoa Cups, microwaved for 70 sec (Coles Supermarkets, Australia)	Australia	2013	53±3	24	Normal, 10	50	136.6	Glucose, 2h	Standard	Capillary, plasma	Enzymatic	UO[5]
570	Quinoa, cooked, refrigerated, reheated in microwave for 1.5 min (Nature First Organic, Australia)	Australia	2003	53±5	24	Normal, 10	50	73.5	Glucose, 2h	Standard	Capillary, whole blood	HemoCue	UO[5]
571	Quinoa, red, cooked in boiling water for 15 min (Nutritious Foods, Australia)	Australia	2017	54±4	24	Normal, 10	50	74.4	Glucose, 2h	Standard	Capillary, plasma	Enzymatic	UO[5]
	Quinoa, mean of four foods			53									
572	Quinoa, retort product, Coles Simply Gluten Free Mexican Style Quinoa and Brown Rice Cups, microwaved for 70 sec (Coles Supermarkets, Australia)	Australia	2013	49±3	22	Normal, 10	50	167.8	Glucose, 2h	Standard	Capillary, plasma	Enzymatic	UO[5]
	**Rice**												
573	Rice flour, 10% resistant starch, prepared with boiled water (Youtang Shanghai Academy of Agricultural Sciences, China)	China	2020	56±4	25	Normal, 12	25	32.5	Glucose, 2h	Standard	Capillary, plasma	Enzymatic	UO[18]
	**Basmati, white rice, boiled**												
574	Basmati, white, polished, cooked 10 min	UK	2009*	50±6	23	Normal, 10	50	64.8	Glucose, 2h	Standard	Capillary, whole blood	HemoCue	65
575	Basmati rice (Dreamrice, Singapore)	Singapore	2015*	56±3	25	Normal, Chinese, 25	50	66.5	Glucose, 2h	Standard	Capillary, whole blood	HemoCue	66

Food Number and Item		Country of food production	Year of test[1]	GI[2] ± SEM (Glu = 100)	GL	Subjects (type & number)	Avail carb (Test portion)	Test portion[3] (g)	Reference food & time period	Timepoints	Sample collection	Sample analysis method[4]	Ref.
576	Basmati rice (SunRice; Ricegrowers Ltd.; Leeton, NSW, Australia)[13]	Australia	2013*	57±4	26	Normal European, 31	50	NS	Glucose, 2h	Standard	Capillary, whole blood	HemoCue	67
577	Basmati, white, boiled (Mahatma brand, Australia)	Australia	1995	58±8	26	Normal, 9	50	63.0	Bread, 2h	Standard	Capillary, plasma	Enzymatic	UO[5]
578	Basmati, white, boiled (SunRice brand, Rice Growers Co-Op., Australia)	Pakistan	2003	59±6	27	Normal, 10	50	63.3	Glucose, 2h	Standard	Capillary, plasma	Enzymatic	UO[5]
579	Basmati rice (Dreamrice, Singapore)	Singapore	2015*	60±4	27	Normal, AsianIndian, 25	50	66.5	Glucose, 2h	Standard	Capillary, whole blood	HemoCue	66
580	Basmati rice (Dreamrice, Singapore)	Singapore	2015*	63±3	28	Normal, Malay, 25	50	66.5	Glucose, 2h	Standard	Capillary, whole blood	HemoCue	66
581	Basmati, white, boiled (SunRice brand, Rice Growers Co-Op., Australia)	Pakistan	2005	65±7	29	Normal, 10	50	69.0	Glucose, 2h	Standard	Capillary, plasma	Enzymatic	UO[5]
582	Basmati rice (Laila Basmati Rice, Surya Foods, Harwich, UK)	UK	2015*	66±7	30	Normal, 10	50	NS	Glucose, 2h	Standard	Capillary, whole blood	HemoCue	68
583	Basmati rice (SunRice; Ricegrowers Ltd.; Leeton, NSW)[13]	Australia	2013*	67±5	30	Normal Chinese, 32	50	NS	Glucose, 2h	Standard	Capillary, whole blood	HemoCue	67
	Basmati rice, white, mean of 10 studies			60									
584	Basmati, parboiled long grain rice, Maharani brand (Chaman Lal Setia Exports Ltd, India)	India	2012	52±6	23	Normal, 9	50	63.9	Glucose, 2h	Standard	Capillary, plasma	Enzymatic	UO[5]
585	Basmati, brown (unpolished milled basmati rice), cooked for 25 min	UK	2009*	75±8	34	Normal, 10	50	71.9	Glucose, 2h	Standard	Capillary, whole blood	HemoCue	65
586	Basmati, white and brown (mixture of 60% white and 40% brown basmati rice), cooked for 25 min	UK	2009*	59±9	27	Normal, 10	50	67	Glucose, 2h	Standard	Capillary, whole blood	HemoCue	65
587	Basmati with wild rice (mixture of 83% easy-cook basmati and 17% North American wild rice (*Zizania palustris L.*), cooked 20 min	UK	2009*	63±8	28	Normal, 10	50	65	Glucose, 2h	Standard	Capillary, whole blood	HemoCue	65

**Basmati, white rice, quick cooking**												
588 Precooked basmati rice in pouch, white, reheated in microwave, Uncle Ben's Express® (Masterfoods, UK)	UK	2001	57±4	26	Normal, 10	50	151.5	Glucose, 2h	Standard	Capillary, plasma	Enzymatic	UO[5]
589 Quick cooking white basmati, cooked 10 min, Uncle Ben's® Superior (Masterfoods, Belgium)	Belgium	2000	60±5	27	Normal, 10	50	67.5	Glucose, 2h	Standard	Capillary, plasma	Enzymatic	UO[5]
590 Basmati, white, express rice, microwaved for 2 min (Uncle Ben's®, Masterfoods, Australia)	Australia	2011	63±6	28	Normal, 9	50	166.7	Glucose, 2h	Standard	Capillary, plasma	Enzymatic	UO[5]

Food Number and Item	Country of food production	Year of test[1]	GI[2] ± SEM (Glu = 100)	GL	Subjects (type & number)	Avail carb (Test portion)	Test portion[3] (g)	Reference food & time period	Timepoints	Sample collection	Sample analysis method[4]	Ref.
591 Basmati, easy-cook (heat-treated white, polished basmati rice), cooked 15 min	UK	2009*	80±8	36	Normal, 10	50	64.1	Glucose, 2h	Standard	Capillary, whole blood	HemoCue	65
Basmati, quick cooking, mean of four foods			65									
**Doongara, white rice (SunRice CleverRice™ brand, Rice Growers Co-Op., Australia)**												
592 Doongara, white, cooked in rice cooker	Australia	2007	48±4	22	Normal, 9	50	64.1	Glucose, 2h	Standard	Capillary, plasma	Enzymatic	UO[5]
593 Doongara, white rice (SunRice CleverRice, Rice Growers Coop, New Zealand)[13]	New Zealand	2011*	48±4	22	Normal, 30	50	NS	Glucose, 2h	Standard	Capillary, whole blood	HemoCue	69
594 Doongara, white	Australia	2015	53±4	24	Normal, 10	50	66.0	Glucose, 2h	Standard	Capillary, plasma	Enzymatic	UO[5]
595 Doongara, white	Australia	2005	54±6	24	Normal, 10	50	65.8	Glucose, 2h	Standard	Capillary, plasma	Enzymatic	UO[5]
596 Doongara, white	Australia	1995	54±7	24	Normal, 9	50	185	Bread, 2h	0,15,30,45,60,75,90,105, 120	Capillary, plasma	Enzymatic	70
597 Doongara rice, cooked in rice cooker[13]	Australia	2013*	55±4	25	Normal European, 31	50	NS	Glucose, 2h	Standard	Capillary, whole blood	HemoCue	67
598 Doongara rice, cooked in rice cooker[13]	Australia	2013*	67±4	30	Normal Chinese, 32	50	NS	Glucose, 2h	Standard	Capillary, whole blood	HemoCue	67

Food Number and Item		Country of food production	Year of test[1]	GI[2] ± SEM (Glu = 100)	GL	Subjects (type & number)	Avail carb (Test portion)	Test portion[3] (g)	Reference food & time period	Timepoints	Sample collection	Sample analysis method[4]	Ref.
	Doongara rice, mean of seven studies			54									
	**Jasmine, white rice**												
599	Jasmine rice, white Thai (Double FP brand, NTUC Fairprice Co-operative Ltd., Singapore), chewed 15 times	Singapore	2014*	68±3	31	Normal, 15	50	NS	Glucose, 2h	Standard	Capillary, whole blood	Enzymatic	71
600	Jasmine rice, cooked in rice cooker (SunRice; Ricegrowers Ltd., NSW, Australia)[13]	Australia	2013*	68±4	31	Normal European, 31	50	NS	Glucose, 2h	Standard	Capillary, whole blood	HemoCue	67
601	Jasmine rice, steamed for 30 min, 31.6 g rice cooked with 35 mL water (COFCO China)	Thailand	2016	76±5	34	Normal, 10	25	31.6	Glucose, 2h	Standard	Capillary, whole blood	Enzymatic	UO[18]
602	Jasmine Fragrant rice, white (SunRice brand, Ricegrowers Limited, Australia)	Australia	2008	79±5	36	Normal, 10	50	63.0	Glucose, 2h	Standard	Capillary, plasma	Enzymatic	UO[5]
603	Jasmine Fragrant rice (SunRice, Ricegrowers Limited, NSW)	Australia	2011	80±3	36	Normal, 10	50	63.0	Glucose, 2h	Standard	Capillary, plasma	Enzymatic	UO[5]
604	Jasmine rice, cooked in rice cooker (SunRice; Ricegrowers Ltd., NSW, Australia)[13]	Australia	2013*	80±4	36	Normal Chinese, 32	50	NS	Glucose, 2h	Standard	Capillary, whole blood	HemoCue	67
605	Jasmine long grain rice	Canada	2010*	82±8	37	Normal, 10	50	62.5	Bread, 2h	Standard	Capillary, whole blood	YSI	56
606	Jasmine white rice (Uncle Ben's Jasmine Rice, Mars Canada, Bolton, Ontario, Canada)	Canada	2010	84±4	38	Normal, 10	50	60.0	Glucose, 2h	Standard	Capillary, plasma	Enzymatic	39
607	Jasmine rice, white Thai (Double FP brand, NTUC Fairprice Co-operative Ltd., Singapore); chewed 30 times	Singapore	2014*	88±10	40	Normal, 14	50	NS	Glucose, 2h	Standard	Capillary, whole blood	Enzymatic	71
608	Jasmine Fragrant rice, white (SunRice brand, Ricegrowers Limited, Australia)	Australia	2006	89±4	40	Normal, 10	50	63.0	Glucose, 2h	Standard	Capillary, plasma	Enzymatic	UO[5]
609	Jasmine rice (Double FP Thai Hom Mali premium quality fragrant rice, Thailand)[11]	Thailand	2015*	90±5	41	Normal, AsianIndian, 25	50	63.6	Glucose, 2h	Standard	Capillary, whole blood	HemoCue	66

	Food Number and Item	Country of food production	Year of test[1]	GI[2] ± SEM (Glu = 100)	GL	Subjects (type & number)	Avail carb (Test portion)	Test portion[3] (g)	Reference food & time period	Timepoints	Sample collection	Sample analysis method[4]	Ref.
610	Jasmine rice (Double FP Thai Hom Mali premium quality fragrant rice, Thailand)[11]	Thailand	2015*	91±4	41	Normal, Chinese, 25	50	63.6	Glucose, 2h	Standard	Capillary, whole blood	HemoCue	66
611	Jasmine rice (Double FP Thai Hom Mali premium quality fragrant rice, Thailand)[11]	Thailand	2015*	92±4	41	Normal, Malay, 25	50	63.6	Glucose, 2h	Standard	Capillary, whole blood	HemoCue	66
612	Rice, white, Thai Hom Mali fragrant rice, cooked in rice cooker (NTUC Fairprice, Singapore)	Singapore	2013	96±6	43	Normal, 12	50	63.2	Glucose, 3h[21]	0,15,30,45,60,90,120,150,180	Capillary, whole blood	HemoCue	72
613	Jasmine rice, Jazzmen, cooked in rice cooker (Jazzmen Rice, Inc., USA)[11]	USA	2014*	106±13	48	Normal, 12	50	68.8	Glucose, 2h	Standard	Capillary, whole blood	HemoCue	73
614	Jasmine rice, white, cooked in rice cooker (Golden World Foods, Bangkok, Thailand)	Thailand	2000	109±10	49	Normal, 12	50	63.2	Glucose, 2h	Standard	Capillary, plasma	Enzymatic	74
615	Jasmine rice, Mahatma, cooked in rice cooker (Riviana Foods Inc., Thailand)[11]	Thailand	2014*	115±7	52	Normal, 12	50	62.5	Glucose, 2h	Standard	Capillary, whole blood	HemoCue	73
616	Jasmine rice, Reindeer, cooked in rice cooker (Oriental Jasmine Rice Co., Thailand)[11]	Thailand	2014*	116±11	52	Normal, 12	50	62.5	Glucose, 2h	Standard	Capillary, whole blood	HemoCue	73
	Jasmine rice, white, mean of 18 studies			89									
	**Rice, brown**												
617	Brown Doongara rice (SunRice; Ricegrowers Ltd., NSW, Australia)	Australia	2016	51±5	23	Normal, 10	50	69.9	Glucose, 2h	Standard	Capillary, plasma	Enzymatic	UO[5]
618	Brown Doongara rice (SunRice; Ricegrowers Ltd., NSW, Australia)	Australia	2006	54±4	24	Normal, 10	50	68.7	Glucose, 2h	Standard	Capillary, plasma	Enzymatic	UO[5]
619	Brown rice, boiled in excess water for 25 min (SunRice brand, Rice Growers Co-op, Australia)	Australia	2005	72±6	32	Normal, 9	50	69.8	Glucose, 2h	Standard	Capillary, plasma	Enzymatic	UO[5]
620	Brown rice	China	2005	87±2	39	Normal, 10	50	NS	Glucose, 2h	Standard	Venous, NS	NS	12

621	Brown rice (SunRice, Ricegrowers Ltd., NSW, Australia)[13]	Australia	2013*	65±4	29	Normal European, 31	50	NS	Glucose, 2h	Standard	Capillary, whole blood	HemoCue	67
622	Brown rice (SunRice, Ricegrowers Ltd., NSW, Australia)[13]	Australia	2013*	78±5	35	Normal Chinese, 32	50	NS	Glucose, 2h	Standard	Capillary, whole blood	HemoCue	67
623	Brown rice, variety Tai Ken #9; *Oryza sativa* L. japonica (Union Rice Company; Taipei), soaked overnight and cooked in a rice cooker[22]	Taiwan	2010*	58±0.2	26	Normal, 10	50	84	Bread, 2h	Standard	Capillary, whole blood	Enzymatic	60
624	Brown rice (0% polished), prepared from the Indian rice variety "Bapatla" (BPT-5204), pressure cooked for 8 min	India	2017*	58±7	26	Normal, 12	50	67	Glucose, 2h	Standard	Capillary, whole blood	HemoCue	75
	**Rice, brown, instant**												
625	Brown Rice, Uncle Ben's® Ready Whole Grain (pouch) (Effem Foods, USA)	USA	2006	48±5	22	Normal, 10	50	179.5	Bread, 2h	Standard	Capillary, whole blood	YSI	UO[5]
626	Brown & Wild, Uncle Ben's® Ready Whole Grain Medley™ (pouch) (Effem Foods, USA)	USA	2006	45±5	20	Normal, 10	50	192.1	Bread, 2h	Standard	Capillary, whole blood	YSI	UO[5]
627	Chicken Flavored Brown Rice, Uncle Ben's® Ready Whole Grain (pouch) (Effem Foods, USA)	USA	2005	46±4	21	Normal, 10	50	192.3	Bread, 2h	Standard	Capillary, whole blood	YSI	UO[5]
628	Medium Grain Brown Rice in 90 sec, microwaved on high (SunRice brand, Rice Growers Co-Op., Australia)	Australia	2005	59±8	27	Normal, 10	50	147.1	Glucose, 2h	Standard	Capillary, plasma	Enzymatic	UO[5]
	Rice Instant, brown, mean of four foods			50									
	**Rice, Long grain, white, boiled**												
629	Long grain, white (Uncle Ben’s, Auckland, New Zealand)	New Zealand	2000	56±7	25	Normal, 14	50	NS	Glucose, 2h	Standard	Venous, plasma	Enzymatic	27
630	Premium long grain, white (SunRice brand, Rice Growers Co-Op., Australia)	Australia	2005	59±7	27	Normal, 10	50	66.2	Glucose, 2h	Standard	Capillary, plasma	Enzymatic	UO[5]
631	Long grain, white, boiled 7 min (Star brand, Gouda Food Products, Canada)	Canada	1999	64±3	29	Normal, 10	50	64.8	Glucose, 2h	Standard	Capillary, plasma	Enzymatic	UO[5]
632	Long grain, boiled (Star Brand, Goudas Food Products, Canada)	Canada	2001	69±3	31	Normal, 47	50	NS	Glucose, 2h	Standard	Capillary, NS	YSI or Enzymatic	42
633	Long grain, boiled (Star Brand, Goudas Food Products, Canada)	Canada	2001	76±13	34	Normal, 21	50	NS	Glucose, 2h	Standard	Venous, NS	Enzymatic	42
634	Long-grain rice (Indica-type long-grain rice, polished), cooked 15 min	UK	2009*	47±6	21	Normal, 10	50	64.6	Glucose, 2h	Standard	Capillary, whole blood	HemoCue	65

Rice long grain, mean of six foods		62

	Food Number and Item	Country of food production	Year of test[1]	GI[2] ± SEM (Glu = 100)	GL	Subjects (type & number)	Avail carb (Test portion)	Test portion[3] (g)	Reference food & time period	Timepoints	Sample collection	Sample analysis method[4]	Ref.
	**Rice, long grain, white, quick-cooking varieties**												
635	Long grain, easy-cook (heat-treated white polished long-grain rice), cooked 15 min	UK	2009*	47±8	21	Normal, 10	50	68.3	Glucose, 2h	Standard	Capillary, whole blood	HemoCue	65
	**Rice, white, instant**												
636	DGI SunRice Low GI Rice, heated in microwave (Imported by Methuselah Shanghai Medical Technology from SunRice Australia)	Australia	2018	49±4	22	Normal, 10	50	148.4	Glucose, 2h	Standard	Capillary, plasma	Enzymatic	UO[5]
637	Instant Doongara, white, cooked 5 min (Rice Growers Co-op, Australia)	Australia	1995	94±7	42	Normal, 9	50	178	Bread, 2h	0,15,30,45,60,75,90,105, 120	Capillary, plasma	Enzymatic	70
638	Instant rice, white, cooked 3 min	China	2005	46±3	21	Normal, 10	50	NS	Glucose, 2h	Standard	Venous, NS	NS	12
639	Instant rice, white, cooked 6 min	China	2005	87±2	39	Normal, 10	50	NS	Glucose, 2h	Standard	Venous, NS	NS	12
640	Long grain, white, pre-cooked, microwaved 2 min (Express Rice, plain, Uncle Ben's, Masterfoods, UK)	UK	2002	52±5	23	Normal, 10	50	164.5	Glucose, 2h	Standard	Capillary, plasma	Enzymatic	UO[5]
641	Long grain, white, cooked in microwave for 90 sec (Uncle ®, Masterfoods, Australia) Ben's	Australia	2011	59±6	27	Normal, 9	50	162.3	Glucose, 2h	Standard	Capillary, plasma	Enzymatic	UO[5]
642	Long Grain White Rice in 90 Seconds, microwaved (SunRice brand, Rice Growers Co-Op., Australia)	Australia	2005	76±7	34	Normal, 10	50	139.8	Glucose, 2h	Standard	Capillary, plasma	Enzymatic	UO[5]
643	Long Grain White Rice, steamed rice, heated in microwave ® brand, Rice Growers Co-Op., Australia) for 40 sec (SunRice	Australia	2020	45±4	20	Normal, 10	50	148.4	Glucose, 2h	Standard	Capillary, plasma	Enzymatic	UO[5]
	Long Grain & Wild, Uncle Ben's® Ready Rice (pouch) 644 (Effem Foods, USA)	USA	2003	49±3	22	Normal, 10	50	177.9	Glucose, 2h	Standard	Capillary, plasma	Enzymatic	UO[5]
	Original Long Grain, Uncle Ben's® Ready Rice pouch) 645 (Effem Foods, USA)	USA	2003	48±5	22	Normal, 10	50	162.8	Glucose, 2h	Standard	Capillary, plasma	Enzymatic	UO[5]

Food Number and Item	Country of food production	Year of test[1]	GI[2] ± SEM (Glu = 100)	GL	Subjects (type & number)	Avail carb (Test portion)	Test portion[3] (g)	Reference food & time period	Timepoints	Sample collection	Sample analysis method[4]	Ref.
Rice, parboiled, pre-cooked, ready to eat (Riso Blond 646 Espresso (Riso Gallo, Italy)	Italy	2009	67±8	30	Normal, 10	50	116	Glucose, 2h	Standard	Capillary, whole blood	YSI	3
647 Steamed Low GI White Rice (SunRice® brand, Rice Growers Co-Op., Australia), heated in microwave	Australia	2013	52±5	23	Normal, 10	50	168.9	Glucose, 2h	Standard	Capillary, plasma	Enzymatic	UO[5]
Instant white rice, mean of 12 foods			60									
**Rice, boiled, white, other varieties**												
648 Arborio, risotto rice, boiled (SunRice brand, Rice Growers Co-Op., Australia)	Australia	2005	69±7	31	Normal, 10	50	NS	Glucose 2 h	Standard	Capillary, plasma	Enzymatic	UO[5]
649 Medium grain white rice, boiled 12 min (Sun Rice brand, Rice Growers Co-Op., Australia)	Australia	2005	75±8	34	Normal, 10	50	63.5	Glucose, 2h	Standard	Capillary, plasma	Enzymatic	UO[5]
650 Moolgiri white rice (Tajmahal Agro Industries, India)	India	2005	54±5	24	Normal, 10	50	NS	Glucose, 2h	Standard	Capillary, whole blood	YSI	UO[5]
651 White rice, NS	China	2005	83±1	37	Normal, 12	50	NS	Glucose, 2h	Standard	Venous, NS	NS	12
652 White rice, fully polished, prepared from the Indian rice variety "Bapatla" (BPT-5204), pressure cooked for 18 min	India	2017*	80±7	36	Normal, 12	50	76	Glucose, 2h	Standard	Capillary, whole blood	HemoCue	75
653 White rice, Carnaroli, regular (Scotti, Italy)	Italy	2009	64±11	29	Normal, 10	50	63	Glucose, 2h	Standard	Capillary, whole blood	YSI	3
654 White rice, Ponni, cooked in rice cooker for 33 min	India	2012*	70±4	32	Normal, 23	50	236	Glucose, 2h	Standard	Capillary, whole blood	HemoCue	76
655 White rice, Ponni, refined, harvested 2013, pressure-cooked for 30 min	India	2013	81±7	36	Normal, 25	50	63.6	Glucose, 2h	Standard	Capillary, whole blood	HemoCue	77
656 White rice, Ponni, refined, harvested 2014, pressure-cooked for 30 min	India	2014	77±8	35	Normal, 15	50	65	Glucose, 2h	Standard	Capillary, whole blood	HemoCue	77
White rice, Ponni variety, mean of three studies			76									
657 White rice, Sona Masuri variety, cooked in rice cooker for 35 min	India	2012*	72±5	32	Normal, 23	50	235	Glucose, 2h	Standard	Capillary, whole blood	HemoCue	76

658	White rice, Surti Kolam variety, cooked in rice cooker for 32 min	India	2012*	77±4	35	Normal, 23	50	259	Glucose, 2h	Standard	Capillary, whole blood	HemoCue	76
	**Rice porridge/Congee**												
659	Rice porridge, made with Jasmine rice, steamed for 40 min, 31.6 g rice cooked with 250 mL water (COFCO China)	Thailand	2016	80±7	36	Normal, 10	25	250	Glucose, 2h	Standard	Capillary, whole blood	Enzymatic	UO[18]
660	Rice porridge, NS	China	2005	66±5	30	Normal, 9	50	NS	Glucose, 2h	Standard	Venous, NS	NS	12
661	Rice porridge, NS	China	2005	69±6	31	Normal, 10	50	NS	Glucose, 2h	Standard	Venous, NS	NS	12
662	Rice porridge, NS	Australia	2005	88±5	40	Normal, 9	50	469.5	Glucose, 2h	Standard	Capillary, plasma	Enzymatic	UO[5]
	Rice porridge/congee, mean of four foods			76									
663	Rice porridge, made from rice bran	China	2005	19±1	9	Normal, 9	50	NS	Glucose, 2h	Standard	Venous, NS	NS	12
664	Rice porridge, made from black rice	China	2005	42±3	19	Normal, 9	50	NS	Glucose, 2h	Standard	Venous, NS	NS	12
665	Rice porridge, made from sticky rice	China	2005	65±7	29	Normal, 9	50	NS	Glucose, 2h	Standard	Venous, NS	NS	12

Food Number and Item		Country of food production	Year of test[1]	GI[2] ± SEM (Glu = 100)	GL	Subjects (type & number)	Avail carb (Test portion)	Test portion[3] (g)	Reference food & time period	Timepoints	Sample collection	Sample analysis method[4]	Ref.
	**Rice, specialty rice**												
	Broken rice, white, cooked in rice cooker (Lion foods, 666 Bangkok, Thailand)	Thailand	2000	86±8	39	Normal, 11	50	63.2	Glucose, 2h	Standard	Capillary, plasma	Enzymatic	74
667	Glutinous rice, white, cooked in rice cooker (Bangsue Chia Meng Rice Co., Bangkok, Thailand)	Thailand	2000	94±6	42	Normal, 11	50	65.5	Glucose, 2h	Standard	Capillary, plasma	Enzymatic	74
668	Glutinous rice, white, cooked in rice cooker (Bangsue Chia Meng Rice Mill, Bangkok, Thailand)	Thailand	2001	98±7	44	Normal, 12	50	65.5	Glucose, 2h	Standard	Capillary, plasma	Enzymatic	UO[5]
669	Guilin rice vermicelli (Chinese rice vermicelli), cooked 8 min	UK	2009*	37±8	17	Normal, 10	50	63.5	Glucose, 2h	Standard	Capillary, whole blood	HemoCue	65
670	Hassawi rice (Al-Hassa, Saudi Arabia)	Saudi Arabia	2011*	59±5	27	Normal, 10	25	95.6	Glucose, 2h	Standard	Capillary, plasma	YSI	78

Food Number and Item		Country of food production	Year of test[1]	GI[2] ± SEM (Glu = 100)	GL	Subjects (type & number)	Avail carb (Test portion)	Test portion[3] (g)	Reference food & time period	Timepoints	Sample collection	Sample analysis method[4]	Ref.
671	High-fiber white rice, harvested 2013 (Kharif), pressure-cooked for 32 min	India	2013	59±4	27	Normal, 25	50	67	Glucose, 2h	Standard	Capillary, whole blood	HemoCue	77
672	High-fiber white rice, harvested 2014 (Rabi), pressure-cooked for 32 min	India	2013	64±4	29	Normal, 25	50	100	Glucose, 2h	Standard	Capillary, whole blood	HemoCue	77
	Japanese Style Sushi rice, white (SunRice brand, Rice 673 Growers Co-Op., Australia)	Australia	2005	85±10	38	Normal, 10	50	66.2	Glucose, 2h	Standard	Capillary, plasma	Enzymatic	UO[5]
674	Jiangxi rice vermicelli (Chinese rice vermicelli), cooked for 8 min	UK	2009*	40±6	18	Normal, 10	50	63.5	Glucose, 2h	Standard	Capillary, whole blood	HemoCue	65
675	Koshihikari rice, white (SunRice brand, Rice Growers Co-Op., Australia	Australia	2005	73±7	33	Normal, 10	50	64.9	Glucose, 2h	Standard	Capillary, plasma	Enzymatic	UO[5]
	Mighty Rice, short grain white rice, boiled for 16 min 676 (VitaRice, Mauritius)	Mauritius	2010	48±5	22	Normal, 10	50	73.5	Glucose, 2h	Standard	Capillary, plasma	Enzymatic	UO[5]
677	Red Raw rice, Sri Lankan, cooked in rice cooker	Sri Lanka	2006	59±4	27	Normal, 10	50	63.9	Glucose, 2h	Standard	Capillary, plasma	Enzymatic	UO[5]
678	Red rice, Thai, unpolished, cooked 25 min	UK	2009*	76±8	34	Normal, 10	50	67.4	Glucose, 2h	Standard	Capillary, whole blood	HemoCue	65
679	Roasted Chicken Flavored, Uncle Ben's® Ready Rice (pouch) (Effem Foods, USA)	USA	2003	51±4	23	Normal, 10	50	176.7	Glucose, 2h	Standard	Capillary, plasma	Enzymatic	UO[5]
	Santa Fe, Uncle Ben's® Ready Whole Grain Medley™ 680 (pouch) (Effem Foods, USA)	USA	2005	48±6	22	Normal, 10	50	205.1	Bread, 2h	Standard	Capillary, whole blood	YSI	UO[5]
681	Spanish Style, Uncle Ben's® Ready Rice (pouch) (Effem Foods, USA)	USA	2003	51±4	23	Normal, 10	50	170.7	Glucose, 2h	Standard	Capillary, plasma	Enzymatic	UO[5]
682	Sticky rice	China	2005	87±2	39	Normal, 10	50	NS	Glucose, 2h	Standard	Venous, NS	NS	12
683	Sticky rice	China	2005	88±2	40	Normal, 9	50	NS	Glucose, 2h	Standard	Venous, NS	NS	12
684	Sticky rice, high-amylose	China	2005	50±2	23	Normal, 10	50	NS	Glucose, 2h	Standard	Venous, NS	NS	12
685	Sticky rice, Thai glutinous rice, cooked for 10 min	UK	2009*	92±8	41	Normal, 10	50	64.7	Glucose, 2h	Standard	Capillary, whole blood	HemoCue	65

686	Rice, under milled, minimally polished 2.3%, prepared from the Indian rice variety "Bapatla" (BPT-5204), pressure cooked for 10 min	India	2017*	73±5	33	Normal, 12	50	71	Glucose, 2h	Standard	Capillary, whole blood	HemoCue	75
	Vegetable Harvest, Uncle Ben's® Ready Whole Grain 687 Medley™ (pouch) (Effem Foods, USA)	USA	2005	48±5	22	Normal, 10	50	206.8	Bread, 2h	Standard	Capillary, whole blood	YSI	UO[5]
	**Rice, Parboiled**												
688	Parboiled brown rice, Uncle Ben’s® Natural Wholegrain Instant brown rice, cooked for 20 min	India	2019*	88±7	40	Normal, 15	50	76.9	Glucose, 2h	Standard	Capillary, whole blood	HemoCue	79
689	Parboiled rice, cooked 20 min, Uncle Ben's Natur-reis® (Masterfoods, Belgium)	Belgium	2000	64±7	29	Normal, 10	50	71.8	Glucose, 2h	Standard	Capillary, plasma	Enzymatic	UO[5]
690	Parboiled rice, long grain white Uncle Ben's®	Belgium	2011*	54±7	24	Normal, 10	25	45.9	Glucose, 2h	Standard	Capillary, plasma	YSI	78
691	Parboiled, long-grain rice (Harlem Foods AS, Oslo) [11]	Norway	2019*	57±5	26	Normal, 13	50	179.9	Glucose, 2h	Standard	Capillary, plasma	YSI	80
692	Parboiled, long grain, 10 min cooking time (Uncle Ben's, Masterfoods, Belgium)	Belgium	2000	68±6	31	Normal, 10	50	65.8	Glucose, 2h	Standard	Capillary, plasma	Enzymatic	UO[5]
693	Parboiled, long grain, 20 min cooking time (Uncle Ben's, Masterfoods, Belgium)	Belgium	2000	75±7	34	Normal, 10	50	66.1	Glucose, 2h	Standard	Capillary, plasma	Enzymatic	UO[5]
694	Rice, parboiled, NS	Canada	2010*	51±4	23	Normal, 10	50	63	Bread, 2h	Standard	Capillary, whole blood	YSI	81
695	Parboiled rice, NS (Riso Blond Risotti, Riso Gallo, Italy)	Italy	2009	53±6	24	Normal, 10	50	59	Glucose, 2h	Standard	Capillary, whole blood	YSI	3
696	Parboiled rice, NS (Uncle Ben’s, Masterfoods Australia New Zealand, NSW, Australia)[13]	Australia	2013*	57±3	26	Normal European, 31	50	NS	Glucose, 2h	Standard	Capillary, whole blood	HemoCue	67
697	Parboiled rice, NS (Riso Blond Veloce e Versatile, Riso Gallo, Italy)	Italy	2009	61±11	27	Normal, 10	50	64	Glucose, 2h	Standard	Capillary, whole blood	YSI	3
698	Parboiled rice, NS (Uncle Ben's, Masterfoods Australia New Zealand, NSW, Australia)[13]	Australia	2013*	72±5	32	Normal Chinese, 32	50	NS	Glucose, 2h	Standard	Capillary, whole blood	HemoCue	67
699	Parboiled rice, white, cooked 5 min, Uncle Ben's Snabbris® (Masterfoods, Belgium)	Belgium	2000	74±5	33	Normal, 10	50	62.1	Glucose, 2h	Standard	Capillary, plasma	Enzymatic	UO[5]

Food Number and Item		Country of food production	Year of test[1]	GI[2] ± SEM (Glu = 100)	GL	Subjects (type & number)	Avail carb (Test portion)	Test portion[3] (g)	Reference food & time period	Timepoints	Sample collection	Sample analysis method[4]	Ref.
700	Rice, whole-meal, parboiled (Riso Blond Integrale, Riso Gallo, Italy)	Italy	2009	56±6	25	Normal, 10	50	66	Glucose, 2h	Standard	Capillary, whole blood	YSI	3
	Parboiled rice, mean of 10 studies			64									
	**Wheat**												
	**Wheat, precooked kernels**												
701	Durum wheat, precooked, cooked 20 min (Ebly, Chateaudun, France)	France	2000	52±4	23	Normal, 10	50	75.3	Glucose, 2h	Standard	Capillary, plasma	Enzymatic	UO[5]
702	Durum wheat, precooked, cooked 10 min (Ebly, France)	France	2000	50±5	23	Normal, 10	50	75.3	Glucose, 2h	Standard	Capillary, plasma	Enzymatic	UO[5]
703	Durum wheat, precooked in pouch, reheated in microwave, Ebly Express (Ebly, France)	France	2000	40±5	18	Normal, 10	50	159.2	Glucose, 2h	Standard	Capillary, plasma	Enzymatic	UO[5]
704	Wheat, pearled (Grano, Barilla, Italy)	Italy	2009	72±8	32	Normal, 9	50	77	Glucose, 2h	Standard	Capillary, whole blood	YSI	3
705	Whole wheat powder	China	2005	42±2	19	Normal, 10	50	NS	Glucose, 2h	Standard	Venous, NS	NS	12
706	Wheat flour, raw (2005 from Griffith NSW, Chara, Row10, Plot 6:181), consumed suspended in 250 mL water	Australia	2009	22±3	10	Normal, 10	50	NS	Glucose, 2h	Standard	Capillary, plasma	Enzymatic	10
707	Wheat flour, raw, consumed suspended in 250 mL water (Coles Smart Buy Plain Flour, Coles Supermarkets Pty Ltd)	Australia	2009	20±2	9	Normal, 10	50	68.9	Glucose, 2h	Standard	Capillary, plasma	Enzymatic	10
	**Other cereals**												
708	Beta Flour (oat, flaxseed, buckwheat, pearl barley, soybean and wheat flours), tested prepared with water, steamed (Inner Mongolia Golden Flax Biotech Pty Ltd, China)	China	2019	27±3	12	Normal, 10	50	87.1	Glucose, 2h	Standard	Capillary, plasma	Enzymatic	UO[5]
709	Mixed cereals powder, NS	China	2005	58±1	26	Normal, 9	50	NS	Glucose, 2h	Standard	Venous, NS	NS	12
710	3 Cereals (rice, oat, kamut) (Riso Gallo, Italy)	Italy	2009	41±6	18	Normal, 10	50	67	Glucose, 2h	Standard	Capillary, whole blood	YSI	3
711	3 Cereals (rice, spelt, barley) (Riso Gallo, Italy)	Italy	2009	44±8	20	Normal, 10	50	65	Glucose, 2h	Standard	Capillary, whole blood	YSI	3
712	Cereal powder, various cereals NS	China	2005	58±1	26	Normal, 9	50	NS	Glucose, 2h	Standard	Venous, NS	NS	12

713	Teff, brown (Macro brand, Woolworths Australia Pty Ltd), boiled in water for 15 min	Australia	2016	57±5	26	Normal, 10	50	76.9	Glucose, 2h	Standard	Capillary, plasma	Enzymatic	UO[5]
714	Spelt, pearled (Farro, Barilla, Italy)	Italy	2009	63±10	28	Normal, 9	50	75	Glucose, 2h	Standard	Capillary, whole blood	YSI	3

Food Number and Item		Country of food production	Year of test[1]	GI[2] ± SEM (Glu = 100)	GL	Subjects number)(type &	Avail carb (Test portion)	Test portion[3] (g)	Reference food & time period	Timepoints	Sample collection	Sample analysis method[4]	Ref.
	**COOKIES**												
	Average available carbohydrate portion = 20 g, this value was used to determine the nominal GL for each item in this category.												
715	Apricot Temptation fruit cookies, 97% fat-free (Freedom Foods, Australia)	Australia	2007	47±4	9	Normal, 10	50	78.7	Glucose, 2h	Standard	Capillary, whole blood	HemoCue	UO[10]
716	Barquette Abricot (LU, Ris, Orangis, France)	France	2002	71±6	14	Normal, 11-14	50	65	Glucose, 2h	Standard	Capillary, plasma	Enzymatic	53
717	Bebe Dobre Rano Chocolate (LU, Czech Republic)	Czech Republic	2002	46±4	9	Normal, 12	50	72.2	Glucose, 2h	Standard	Capillary, plasma	Enzymatic	UO[5]
718	Bebe Dobre Rano Chocolate (LU, Czech Republic)	Czech Republic	2006	46±3	9	Normal, 12	50	72.0	Glucose, 2h	Standard	Capillary, plasma	Enzymatic	UO[5]
719	Bebe Dobre Rano Chocolate (Opavia/LU, Czech Republic)	Czech Republic	1996-2000	57±9	11	Normal, 11	50	NS	Glucose, 2h	Standard	Capillary, plasma	Enzymatic	UO[23, 24]
	Bebe Dobre Rano Chocolate, mean of three studies			50									
720	Bebe Dobre Rano Coconut (LU, Czech Republic)	Czech Republic	2002	48±3	10	Normal, 12	50	69.0	Glucose, 2h	Standard	Capillary, plasma	Enzymatic	UO[5]
721	Bebe Dobre Rano Four Cereals (LU, Czech Republic)	Czech Republic	2007	51±4	10	Normal, 12	50	71.4	Glucose, 2h	Standard	Capillary, plasma	Enzymatic	UO[5]
722	Bebe Dobre Rano Honey and Hazelnuts (Opavia/LU, Czech Republic)	Czech Republic	1996-2000	51±9	10	Normal, 11	50	NS	Glucose, 2h	Standard	Capillary, plasma	Enzymatic	UO[23, 24]
723	Bebe Dobre Rano Nuts and Honey (LU, Czech Republic)	Czech Republic	2002	41±5	8	Normal, 12	50	70.4	Glucose, 2h	Standard	Capillary, plasma	Enzymatic	UO[5]
724	Bebe Dobre Rano Nuts and Honey (LU, Czech Republic)	Czech Republic	2006	45±4	9	Normal, 12	50	71.5	Glucose, 2h	Standard	Capillary, plasma	Enzymatic	UO[5]

Food Number and Item		Country of food production	Year of test[1]	GI[2] ± SEM (Glu = 100)	GL	Subjects (type & number)	Avail carb (Test portion)	Test portion[3] (g)	Reference food & time period	Timepoints	Sample collection	Sample analysis method[4]	Ref.
725	Bebe Dobre Rano with Milk (LU, Czech Republic)	Czech Republic	2003	50±4	10	Normal, 12	50	70.4	Glucose, 2h	Standard	Capillary, plasma	Enzymatic	UO[5]
726	Bebe Jemne Susenky (Opavia/LU, Czech Republic)	Czech Republic	1996-2000	67±11	13	Normal, 11	50	NS	Glucose, 2h	Standard	Capillary, plasma	Enzymatic	UO[23, 24]
727	Better Choice Multicereals (LU, Denmark)	Denmark	2004	51±4	10	Normal, 12	50	NS	Glucose, 2h	Standard	Capillary, plasma	Enzymatic	UO[5]
728	Better Choice Wholegrain (LU Denmark)	Denmark	2004	46±3	9	Normal, 11	50	NS	Glucose, 2h	Standard	Capillary, plasma	Enzymatic	UO[5]
729	Biscuit, Danone, NS	China	2005	39±4	8	Normal, 9	50	NS	Glucose, 2h	Standard	Venous, NS	NS	12
730	Biscuit, Danone, NS	China	2005	47±4	9	Normal, 9	50	NS	Glucose, 2h	Standard	Venous, NS	NS	12
731	Biscuit, NS	China	2005	72±5	14	Normal, 9	50	NS	Glucose, 2h	Standard	Venous, NS	NS	12
732	Biscuit, thin, NS	China	2005	81±2	16	Normal, 9	50	NS	Glucose, 2h	Standard	Venous, NS	NS	12
733	Biscuits, rolled oat, wholemeal wheat flour, wheat fibre (Gran Cereale Classico, Italy)[6]	Italy	2005	49±4	10	Normal, 10	50	85.9	Glucose, 2h	Standard	Capillary, whole blood	YSI	3
734	Biscuits, wheat flour, butter, cream (Macine, Italy)[6]	Italy	2006	52±7	10	Normal, 10	50	75.6	Glucose, 2h	Standard	Capillary, whole blood	YSI	3
735	Biscuits, wheat flour, oat flour, wholemeal buckwheat flour (6.2%) (Molinetti, Italy)[6]	Italy	2006	58±7	12	Normal, 10	50	77.3	Glucose, 2h	Standard	Capillary, whole blood	YSI	3
736	Biscuit, wholegrain	USA	2018*	54±5	11	Normal, 19	50	82	Glucose, 2h	Standard	Capillary, whole blood	HemoCue	57
737	Biscuits, wholegrain, filled with peanut butter	USA	2018*	44±3	9	Normal, 19	50	102	Glucose, 2h	Standard	Capillary, whole blood	HemoCue	57
738	Breakfast biscuit (Frollino), gluten free (Dr Schär AG/SPA, Italy)[25]	Italy	2015*	37±7	7	Normal, 10	50	66	Glucose, 2h	Standard	Capillary, whole blood	YSI	5
739	Chocolate chip cookies, containing wheat flour and coconut flour	Philippines	2002	44±3	9	Normal, 10	50	NS	Bread, 2h	Standard	Capillary, serum	Enzymatic	2

Food Number and Item		Country of food production	Year of test[1]	GI[2] ± SEM (Glu = 100)	GL	Subjects (type & number)	Avail carb (Test portion)	Test portion[3] (g)	Reference food & time period	Timepoints	Sample collection	Sample analysis method[4]	Ref.
740	Cookie, fiber enriched (Bahlsen GmbH&Co.KG, Hannover, Germany)[11]	Germany	2016*	42±4	8	Normal, 26	50	84.5	Bread, 2h	Standard	Venous, whole blood	Enzymatic	82
741	Cookie, decreased GI variant (sugar-to-flour ratio: 0.45)	Belgium	2010*	37±10	7	Normal, 10	25	62.5	Glucose, 2h	Standard	Venous, plasma/serum	Enzymatic	1
742	Digestives	UK	2006	39±5	8	Normal, 10	50	79.7	Glucose, 2h	Standard	Capillary, whole blood	Glucometer[9]	30
743	Evergreen met Krenten (LU, Netherlands)	Netherlands	1996-2000	66±12	13	Normal, 12	50	NS	Glucose, 2h	Standard	Capillary, plasma	Enzymatic	UO[23]
744	Fior di Latte (Mulino Bianco, Barilla, Italy)	Italy	2012	47±4	9	Normal, 9	50	70	Glucose, 2h	Standard	Capillary, whole blood	YSI	3
745	Gemme Integrali (Mulino Bianco, Barilla, Italy)	Italy	2012	46±6	9	Normal, 9	50	80	Glucose, 2h	Standard	Capillary, whole blood	YSI	3
746	Gingerbread cookies	Australia	2016	66±4	13	Normal, 9	50	70.1	Glucose, 2h	Standard	Capillary, plasma	Enzymatic	UO[5]
747	Golden Flaxseed biscuits (SCIQ Biotech Pty Ltd, Australia)	Australia	2020	36±5	7	Normal, 10	50	106.2	Glucose, 2h	Standard	Capillary, plasma	Enzymatic	UO[5]
748	Gran Cereale Cioccolato (Grancereale, Barilla, Italy)	Italy	2010	52±10	10	Normal, 9	50	81	Glucose, 2h	Standard	Capillary, whole blood	YSI	3
749	Gran Cereale Frutta (Grancereale, Barilla, Italy)	Italy	2010	51±7	10	Normal, 9	50	87	Glucose, 2h	Standard	Capillary, whole blood	YSI	3
750	Gran Cereale Classico (Grancereale, Barilla, Italy)	Italy	2005	49±4	10	Normal, 10	50	86	Glucose, 2h	Standard	Capillary, whole blood	YSI	3
751	Gran'Dia Banana, Oats and Honey (LU, Brazil)	Brazil	2002	28±5	6	Normal, 11-14	50	65	Glucose, 2h	Standard	Capillary, plasma	Enzymatic	53
752	Gran'Dia Chocolate with five cereals (Danone, Brazil)	Brazil	2002	39±8	8	Normal, 11-14	50	69	Glucose, 2h	Standard	Capillary, plasma	Enzymatic	53
753	Grany en-cas Abricot (LU, France)	France	1996-2000	55±6	11	Normal, 12	50	NS	Glucose, 2h	Standard	Capillary, plasma	Enzymatic	UO[10]
754	Grany en-cas Fruits des bois (LU, France)	France	1996-2000	50±5	10	Normal, 14	50	NS	Glucose, 2h	Standard	Capillary, plasma	Enzymatic	UO[23]

Food Number and Item		Country of food production	Year of test[1]	GI[2] ± SEM (Glu = 100)	GL	Subjects (type & number)	Avail carb (Test portion)	Test portion[3] (g)	Reference food & time period	Timepoints	Sample collection	Sample analysis method[4]	Ref.
755	Grany Rush Apricot (LU, Netherlands)	Netherlands	2000	62±3	12	Normal, 12	50	NS	Glucose, 2h	Standard	Capillary, plasma	Enzymatic	UO[5]
	Grain biscuit, Mixed Berry flavor (belVita, Mondelēz, 756 Shanghai, China)	China	2019	55±6	11	Normal, 12	50	87.5	Glucose, 2h	Standard	Capillary, plasma	Enzymatic	UO[18]
757	Grain biscuits, Milk & Cereal flavor (belVita, Mondelēz, Shanghai, China)	China	2020	53±4	11	Normal, 12	50	78.1	Glucose, 2h	Standard	Capillary, plasma	Enzymatic	UO[18]
758	Györi Édes Jó reggelt! Müzlivel és gyümölccsel / Fruits and Muesli (LU Hungary)	Hungary	2002	45±5	9	Normal, 11	50	69.3	Glucose, 2h	Standard	Capillary, plasma	Enzymatic	UO[5]
759	Györi Édes Jó reggelt! Müzlivel és gyümölccsel / Fruits and Muesli (LU Hungary)	Hungary	2003	49±5	10	Normal, 11	50	69.3	Glucose, 2h	Standard	Capillary, plasma	Enzymatic	UO[5]
760	Györi Édes Jó reggelt! Müzlivel és gyümölccsel / Fruits and Muesli (LU Hungary)	Hungary	2004	47±6	9	Normal, 12	50	69.3	Glucose, 2h	Standard	Capillary, plasma	Enzymatic	UO[5]
	Györi Édes Jó reggelt! Müzlivel és gyümölccsel, mean of three studies			47									
761	Jo Reggelt Fruits and Figs (LU, Hungary)	Hungary	2004	41±6	8	Normal, 11	50	NS	Glucose, 2h	Standard	Capillary, plasma	Enzymatic	UO[5]
762	Jubilee Nuts and Honey (LU, Russia)	Russia	2002	44±3	9	Normal, 11	50	72.1	Glucose, 2h	Standard	Capillary, plasma	Enzymatic	UO[5]
763	Jubilee Nuts and Honey (LU, Russia)	Russia	2003	45±4	9	Normal, 12	50	74.2	Glucose, 2h	Standard	Capillary, plasma	Enzymatic	UO[5]
764	Jyväshyvä Paussi Mysli and Fruits (LU Finland)	Finland	2002	45±5	9	Normal, 11	50	69.3	Glucose, 2h	Standard	Capillary, plasma	Enzymatic	UO[5]
765	Jyväshyvä Paussi Mysli and Fruits (LU Finland)	Finland	2003	49±5	10	Normal, 11	50	69.3	Glucose, 2h	Standard	Capillary, plasma	Enzymatic	UO[5]
766	Jyväshyvä Paussi Mysli and Fruits (LU Finland)	Finland	2004	47±6	9	Normal, 12	50	69.3	Glucose, 2h	Standard	Capillary, plasma	Enzymatic	UO[5]
	Jyväshyvä Paussi Mysli and Fruits, mean of three studies			47									
767	Jyväshyvä Paussi Vanilla (LU Finland)	Finland	2004	55±7	11	Normal, 11	50	NS	Glucose, 2h	Standard	Capillary, plasma	Enzymatic	UO[5]

768	Jyväshyvä Paussi Vanilla (LU Finland)	Finland	2006	39±4	8	Normal, 12	50	NS	Glucose, 2h	Standard	Capillary, plasma	Enzymatic	UO[5]
769	Jyväshyvä Paussi Wholegrain (LU Finland)	Finland	2004	46±3	9	Normal, 11	50	NS	Glucose, 2h	Standard	Capillary, plasma	Enzymatic	UO[5]
770	Kingston, butternut cookies with chocolate filling (Arnott's Pty Ltd, Australia)	Australia	2013	48±6	10	Normal, 10	50	81.3	Glucose, 2h	Standard	Capillary, plasma	Enzymatic	UO[5]
771	Leda Luxury gluten-free chocolate coated cookies (Leda Nutrition, QLD, Australia)	Australia	2004	35±7	7	Normal, 10	50	111.1	Glucose, 2h	Standard	Capillary, plasma	Enzymatic	UO[5]
772	LU P'tit Déjeuner Chocolat (LU, France)	France	2002	42±5	8	Normal, 11-14	50	73	Glucose, 2h	Standard	Capillary, plasma	Enzymatic	53
773	LU P'tit Déjeuner Miel et Pépites Chocolat (LU, France)	France	2002	45±5	9	Normal, 11-14	50	71	Glucose, 2h	Standard	Capillary, plasma	Enzymatic	53
774	LU P'tit Déjeuner Miel et Pépites Chocolat (LU, France)	France	1996-2000	49±8	10	Normal, 11	50	NS	Glucose, 2h	Standard	Capillary, plasma	Enzymatic	UO[23, 24]
775	LU P'tit Déjeuner Miel et Pépites Chocolat (LU, France)	France	2000	52±3	10	Normal, 12	50	76.1	Glucose, 2h	Standard	Capillary, plasma	Enzymatic	UO[5]
	LU P'tit Déjeuner Miel et Pépites Chocolat, mean of three studies			49									
776	LU Petit Dejeuner, Chocolate, low in sugar (LU France)	France	2004	51±7	10	Normal, 11	50	69.1	Glucose, 2h	Standard	Capillary, plasma	Enzymatic	UO[5]
777	LU Petit Dejeuner Chocolate & Cereals (LU, France)	France	2003	46±6	9	Normal, 11	50	70.7	Glucose, 2h	Standard	Capillary, plasma	Enzymatic	UO[5]
778	LU Petit Dejeuner Chocolate & Cereals (LU, France)	France	2006	58±6	12	Normal, 12	50	71.4	Glucose, 2h	Standard	Capillary, plasma	Enzymatic	UO[5]
779	LU Petit Dejeuner Cereals & Chocolate Chips, low in sugar (LU France, LU Belgium, LU Czech Republic)	NS	2004	37±5	7	Normal, 11	50	NS	Glucose, 2h	Standard	Capillary, plasma	Enzymatic	UO[5]
780	LU Petit Dejeuner Coconut, nuts and chocolate (LU France)	France	2005	55±6	11	Normal, 12	50	71.8	Glucose, 2h	Standard	Capillary, plasma	Enzymatic	UO[5]
781	LU Petit Dejeuner Coconut, nuts and chocolate (LU France)	France	2006	51±7	10	Normal, 12	50	74.1	Glucose, 2h	Standard	Capillary, plasma	Enzymatic	UO[5]
782	LU Petit Dejeuner Fruits and Muesli (LU France)	France	2002	45±5	9	Normal, 11	50	69.3	Glucose, 2h	Standard	Capillary, plasma	Enzymatic	UO[5]

Food Number and Item		Country of food production	Year of test[1]	GI[2] ± SEM (Glu = 100)	GL	Subjects (type & number)	Avail carb (Test portion)	Test portion[3] (g)	Reference food & time period	Timepoints	Sample collection	Sample analysis method[4]	Ref.
783	LU Petit Dejeuner Fruits and Muesli (LU France)	France	2003	49±5	10	Normal, 11	50	69.3	Glucose, 2h	Standard	Capillary, plasma	Enzymatic	UO[5]
784	LU Petit Dejeuner Fruits and Muesli (LU France)	France	2004	47±6	9	Normal, 12	50	69.3	Glucose, 2h	Standard	Capillary, plasma	Enzymatic	UO[5]
	LU Petit Dejeuner Fruits and Muesli, mean of three studies			47									
785	LU Petit Dejeuner Honey & Chocolate chips (LU France)	France	2003	47±6	9	Normal, 11	50	70.7	Glucose, 2h	Standard	Capillary, plasma	Enzymatic	UO[5]
786	LU Petit Dejeuner Honey & Chocolate chips (LU France)	France	2006	46±4	9	Normal, 11	50	71.6	Glucose, 2h	Standard	Capillary, plasma	Enzymatic	UO[5]
787	LU Petit Dejeuner Milk and Cereals (LU France, LU Belgium)	NS	2004	55±7	11	Normal, 11	50	72.7	Glucose, 2h	Standard	Capillary, plasma	Enzymatic	UO[5]
788	LU Petit Dejeuner Milk and Cereals (LU France, LU Belgium)	NS	2006	39±4	8	Normal, 12	50	74.1	Glucose, 2h	Standard	Capillary, plasma	Enzymatic	UO[5]
789	LU Petit Dejeuner Multicereals (LU France, LU Belgium)	NS	2004	46±3	9	Normal, 11	50	NS	Glucose, 2h	Standard	Capillary, plasma	Enzymatic	UO[5]
790	LU Petit Dejeuner with Fruits and Figs (LU France, LU Belgium)	NS	2004	41±6	8	Normal, 11	50	NS	Glucose, 2h	Standard	Capillary, plasma	Enzymatic	UO[5]
791	LU Petit Dejeuner with Prunes (LU, France)	France	2006	51±6	10	Normal, 12	50	74.5	Glucose, 2h	Standard	Capillary, plasma	Enzymatic	UO[5]
792	Macaroons, containing wheat flour and with coconut flour	Philippines	2002	32±2	6	Normal, 10	50	NS	Bread, 2h	Standard	Capillary, serum	Enzymatic	2
793	Maltmeal wafer (Griffin's Foods Ltd., New Zealand)	New Zealand	2000	50±10	10	Normal, 10	50	NS	Glucose, 2h	Standard	Venous, plasma	Enzymatic	27
794	Montana Chocolate Chip Cookies (Kinnikinnic Foods Inc., Edmonton. AB, Canada)	Canada	2009*	43±3	9	Normal, women, 40	50	63.2	Glucose, 2h	Standard	Capillary, whole blood	YSI	38
795	Montana Chocolate Chip Cookies (Kinnikinnic Foods Inc., Edmonton. AB, Canada)	Canada	2009*	42±2	8	Normal, men, 37	50	63.2	Glucose, 2h	Standard	Capillary, whole blood	YSI	38
796	Montana Chocolate Chip Cookies (Kinnikinnic Foods Inc., Edmonton. AB, Canada)	Canada	2009*	42±3	8	Normal, Caucasian, 40	50	63.2	Glucose, 2h	Standard	Capillary, whole blood	YSI	38

Food Number and Item		Country of food production	Year of test[1]	GI[2] ± SEM (Glu = 100)	GL	Subjects (type & number)	Avail carb (Test portion)	Test portion[3] (g)	Reference food & time period	Timepoints	Sample collection	Sample analysis method[4]	Ref.
797	Montana Chocolate Chip Cookies (Kinnikinnic Foods Inc., Edmonton. AB, Canada)	Canada	2009*	42±3	8	Normal, Non-Caucasian, 37	50	63.2	Glucose, 2h	Standard	Capillary, whole blood	YSI	38
798	Montana Chocolate Chip Cookies (Kinnikinnic Foods Inc., Edmonton. AB, Canada)	Canada	2009*	42±3	8	Normal, ≤40 y, 39	50	63.2	Glucose, 2h	Standard	Capillary, whole blood	YSI	38
799	Montana Chocolate Chip Cookies (Kinnikinnic Foods Inc., Edmonton. AB, Canada)	Canada	2009*	43±3	9	Normal, >40 y, 38	50	63.2	Glucose, 2h	Standard	Capillary, whole blood	YSI	38
800	Montana Chocolate Chip Cookies (Kinnikinnic Foods Inc., Edmonton. AB, Canada)	Canada	2009*	43±3	9	Normal, lean, 37	50	63.2	Glucose, 2h	Standard	Capillary, whole blood	YSI	38
801	Montana Chocolate Chip Cookies (Kinnikinnic Foods Inc., Edmonton. AB, Canada)	Canada	2009*	41±3	8	Normal, overweight, 40	50	63.2	Glucose, 2h	Standard	Capillary, whole blood	YSI	38
	Montana Chocolate Chip Cookies, mean of eight studies			42									
802	Nutrigrain Fruits des bois (Kellogg's, France)	France	2000	57±4	11	Normal, 12	50	79.7	Glucose, 2h	Standard	Capillary, plasma	Enzymatic	UO[5]
803	Oat biscuit	UK	2006	45±7	9	Normal, 10	50	82.2	Glucose, 2h	Standard	Capillary, whole blood	Glucometer[9]	30
804	Oat biscuit	China	2005	55±1	11	Normal, 10	50	NS	Glucose, 2h	Standard	Venous, NS	NS	12
805	Oat biscuit, Nairn's™ Stem Ginger Oat Biscuit (Nairn's Oatcakes Ltd, Edinburgh, Scotland)	UK	2007	55±6	11	Normal, 10	50	73.5	Glucose, 2h	Standard	Capillary, whole blood	HemoCue	UO[26]
806	Oro (Saiwa, Italy)	Italy	2002	61±9	12	Normal, 11-14	50	62	Glucose, 2h	Standard	Capillary, plasma	Enzymatic	53
807	Pavesini (Pavesi, Barilla, Italy)	Italy	2004	52±5	10	Normal, 10	25	57	Glucose, 2h	Standard	Capillary, whole blood	YSI	3
808	Petiki Go ! Fruits and Muesli (LU, Poland)	Poland	2002	45±5	9	Normal, 11	50	69.3	Glucose, 2h	Standard	Capillary, plasma	Enzymatic	UO[5]
809	Petiki Go ! Fruits and Muesli (LU, Poland)	Poland	2003	49±5	10	Normal, 11	50	69.3	Glucose, 2h	Standard	Capillary, plasma	Enzymatic	UO[5]

Food Number and Item		Country of food production	Year of test[1]	GI[2] ± SEM (Glu = 100)	GL	Subjects (type & number)	Avail carb (Test portion)	Test portion[3] (g)	Reference food & time period	Timepoints	Sample collection	Sample analysis method[4]	Ref.
810	Petiki Go ! Fruits and Muesli (LU, Poland)	Poland	2004	47±6	9	Normal, 12	50	69.3	Glucose, 2h	Standard	Capillary, plasma	Enzymatic	UO[5]
	Petiki Go ! Fruits and Muesli, mean of three studies			47									
811	Petit brun extra (LU, France)	France	2002	77±12	15	Normal, 11-14	50	62	Glucose, 2h	Standard	Capillary, plasma	Enzymatic	53
812	Petit LU Normand (LU, France)	France	2000	51±3	10	Normal, 12	50	NS	Glucose, 2h	Standard	Capillary, plasma	Enzymatic	UO[5]
813	Petit LU Roussillon (LU, France)	France	2000	48±4	10	Normal, 12	50	NS	Glucose, 2h	Standard	Capillary, plasma	Enzymatic	UO[5]
814	President's Choice® Blue Menu™ Cranberry Orange Cookies (Loblaw Brands Limited, Canada)	Canada	2004-2007	60±4	12	Normal, 10	50	NS	Bread, 2h	Standard	Capillary, whole blood	YSI	UO[7]
815	President's Choice® Blue Menu™ Crunchy Oat Cookies (Loblaw Brands Limited, Canada)	Canada	2004-2007	62±7	12	Normal, 10	50	NS	Bread, 2h	Standard	Capillary, whole blood	YSI	UO[7]
816	President's Choice® Blue Menu™ Ginger & Lemon Cookies (Loblaw Brands Limited, Canada)	Canada	2004-2007	64±5	13	Normal, 10	50	NS	Bread, 2h	Standard	Capillary, whole blood	YSI	UO[7]
817	Prince Energie+ (LU, France)	France	2000	73±5	15	Normal, 12	50	NS	Glucose, 2h	Standard	Capillary, plasma	Enzymatic	UO[5]
818	Prince fourré chocolat (LU, France)	France	1996-2000	50±5	10	Normal, 12	50	NS	Glucose, 2h	Standard	Capillary, plasma	Enzymatic	UO[23]
819	Prince fourré chocolat (LU, France)	France	1996-2000	53±5	11	Normal, 13	50	NS	Glucose, 2h	Standard	Capillary, plasma	Enzymatic	UO[23]
820	Prince gout chocolat (LU, France)	France	2002	53±5	11	Normal, 11-14	50	69	Glucose, 2h	Standard	Capillary, plasma	Enzymatic	53
821	Prince Petit Déj Cereals (LU, France)	France	2003	52±7	10	Normal, 12	50	NS	Glucose, 2h	Standard	Capillary, plasma	Enzymatic	UO[5]
822	Prince Petit Déj Cereals and Chocolate (LU, France)	France	2004	51±5	10	Normal, 12	50	71.8	Glucose, 2h	Standard	Capillary, plasma	Enzymatic	UO[5]

Food Number and Item		Country of food production	Year of test[1]	GI[2] ± SEM (Glu = 100)	GL	Subjects (type & number)	Avail carb (Test portion)	Test portion[3] (g)	Reference food & time period	Timepoints	Sample collection	Sample analysis method[4]	Ref.
823	Prince Petit Déjeuner Vanille (LU, France and Spain)	NS	1996-2000	45±6	9	Normal, 12	50	NS	Glucose, 2h	Standard	Capillary, plasma	Enzymatic	UO[23]
824	Principe megamanana vanilla (LU, Spain)	Spain	2002	45±6	9	Normal, 11-14	50	69	Glucose, 2h	Standard	Capillary, plasma	Enzymatic	53
825	Rich Tea	UK	2006	40±5	8	Normal, 10	50	70.2	Glucose, 2h	Standard	Capillary, whole blood	Glucometer[9]	30
826	Rotary-Molded biscuit[11]	France	2019*	48±4	10	Normal, 15	50	67.4	Glucose, 2h	Standard	Capillary, plasma	Enzymatic	7
827	Rotary-Molded biscuit[11]	France	2019*	47±4	9	Normal, 15	50	67.4	Glucose, 2h	Standard	Capillary, whole blood	YSI	7
828	Rotary-Molded biscuit[11]	France	2019*	45±6	9	Normal, 13	50	67.4	Glucose, 2h	Standard	Capillary, plasma	Enzymatic	7
	Rotary-Molded biscuit, mean of three studies			47									
829	Sablé des Flandres (LU, France)	France	1996-2000	57±10	11	Normal, 12	50	NS	Glucose, 2h	Standard	Capillary, plasma	Enzymatic	UO[23]
830	Sandwich Rotary-Molded biscuit[11]	France	2019*	44±4	9	Normal, 15	50	63.8	Glucose, 2h	Standard	Capillary, plasma	Enzymatic	7
831	Sandwich Rotary-Molded biscuit[11]	France	2019*	56±4	11	Normal, 15	50	63.8	Glucose, 2h	Standard	Capillary, whole blood	YSI	7
832	Sandwich Rotary-Molded biscuit[11]	France	2019*	52±7	10	Normal, 13	50	63.8	Glucose, 2h	Standard	Capillary, plasma	Enzymatic	7
	Sandwich-Rotary-Molded biscuit, mean of three studies			51									
833	Segreti di Bosco (Mulino Bianco, Barilla, Italy)	Italy	2012	53±7	11	Normal, 9	50	73	Glucose, 2h	Standard	Capillary, whole blood	YSI	3
834	Snack Right Fruit Pillow, Apple and blackberry (Arnotts, Australia)	Australia	2002	43±3	9	Normal, 10	50	76.2	Glucose, 2h	Standard	Capillary, whole blood	HemoCue	UO[5]
835	Snack Right Fruit Pillow, Spicy apple and sultana (Arnotts, Australia)	Australia	2002	45±3	9	Normal, 10	50	76.2	Glucose, 2h	Standard	Capillary, whole blood	HemoCue	UO[5]

Food Number and Item		Country of food production	Year of test[1]	GI[2] ± SEM (Glu = 100)	GL	Subjects (type & number)	Avail carb (Test portion)	Test portion[3] (g)	Reference food & time period	Timepoints	Sample collection	Sample analysis method[4]	Ref.
836	Snack Right Fruit Pillow, Wild berry (Arnott's, Australia)	Australia	2002	52±3	10	Normal, 10	50	70.8	Glucose, 2h	Standard	Capillary, whole blood	HemoCue	UO[5]
837	Snack Right Fruit Slice, Mango and passionfruit (Arnott's, Australia)	Australia	2002	49±3	10	Normal, 10	50	70.1	Glucose, 2h	Standard	Capillary, whole blood	HemoCue	UO[5]
838	Snack Right Fruit Slice, Mixed berry (Arnott's, Australia)	Australia	2002	50±3	10	Normal, 10	50	70.7	Glucose, 2h	Standard	Capillary, whole blood	HemoCue	UO[5]
839	Snack Right Fruit Slice, Original Sultana (97% fat-free) (Arnott's, Australia)	Australia	2002	48±2	10	Normal, 10	50	72.7	Glucose, 2h	Standard	Capillary, whole blood	HemoCue	UO[5]
840	Snack Right Fruit Slice, Sultana with chocolate (Arnott's, Australia)	Australia	2002	45±3	9	Normal, 10	50	75.6	Glucose, 2h	Standard	Capillary, whole blood	HemoCue	UO[5]
841	Sweet biscuit	UK	2007	47±6	9	Normal Caucasian, 10	50	NS	Glucose, 2h	Standard	Capillary, whole blood	Glucometer[9]	58
842	Sweet biscuit	UK	2007	63±4	13	Normal Asian, 10	50	NS	Glucose, 2h	Standard	Capillary, whole blood	Glucometer[9]	58
843	Sweetmeal biscuit	UK	2007	39±4	8	Normal Caucasian, 10	50	NS	Glucose, 2h	Standard	Capillary, whole blood	Glucometer[9]	58
844	Sweetmeal biscuit	UK	2007	42±4	8	Normal Asian, 10	50	NS	Glucose, 2h	Standard	Capillary, whole blood	Glucometer[9]	58
845	Thé (LU, France)	France	202	41±7	8	Normal, 11-14	50	64	Glucose, 2h	Standard	Capillary, plasma	Enzymatic	53
846	Véritable Petit Beurre (LU, France)	France	2002	51±8	10	Normal, 11-14	50	67	Glucose, 2h	Standard	Capillary, plasma	Enzymatic	53
847	Véritable Petit Beurre (LU, France)	France	2002	54±5	11	Normal, 12	50	65.1	Glucose, 2h	Standard	Capillary, plasma	Enzymatic	UO[5]
848	Véritable Petit Beurre (LU, France)	France	2006	54±6	11	Normal, 11	50	63.3	Glucose, 2h	Standard	Capillary, plasma	Enzymatic	UO[5]
	Véritable Petit Beurre, mean of three studies			53									
849	Vitasnella frollini (Saiwa, Italy)	Italy	2002	59±10	12	Normal, 11-14	50	61	Glucose, 2h	Standard	Capillary, plasma	Enzymatic	53

CRACKERS

Average available carbohydrate portion = 15 g, this value was used to determine the nominal GL for each item in this category.

850	BBQ Shapes, savory crackers (Arnott's Pty Ltd, Australia)	Australia	2010	48±4	7	Normal, 10	50	79.0	Glucose, 2h	Standard	Capillary, plasma	Enzymatic	UO[5]
851	Choice grain crackers (Jacob's Bakery, UK)	UK	2006	49±7	7	Normal, 10	50	76.3	Glucose, 2h	Standard	Capillary, whole blood	Glucometer[9]	30
852	Crispbread, soft wheat flour (54%), rye flour (36%), pumpkin seeds (7.5%) (Spianate croccanti con semi di zucca, Grancereale, Barilla, Italy)	Italy	2012	53±5	8	Normal, 10	50	82	Glucose, 2h	Standard	Capillary, whole blood	YSI	3
853	Corn Thins, puffed corn cakes, gluten-free (Real Foods, St Peters, Australia)	Australia	2000	87±10	13	Normal, 10	50	68.8	Glucose, 2h	Standard	Capillary, whole blood	HemoCue	UO[5]
854	Cracker[11]	France	2019*	51±4	8	Normal, 15	50	75.9	Glucose, 2h	Standard	Capillary, plasma	Enzymatic	7
855	Cracker[11]	France	2019*	61±5	9	Normal, 15	50	75.9	Glucose, 2h	Standard	Capillary, whole blood	YSI	7
856	Cracker[11]	France	2019*	60±5	9	Normal, 13	50	75.9	Glucose, 2h	Standard	Capillary, plasma	Enzymatic	7
	Cracker, mean of three studies			57									
857	Crackers, soft wheat flour, salty (Cracker integrali Sfoglia di grano, Mulino Bianco, Barilla, Italy)	Italy	2010	63±5	9	Normal, 10	50	81	Glucose, 2h	Standard	Capillary, whole blood	YSI	3
858	Crackers, whole-meal soft wheat flour (Cracker salati Sfoglia di grano, Mulino Bianco, Barilla, Italy)	Italy	2010	63±8	9	Normal, 10	50	72	Glucose, 2h	Standard	Capillary, whole blood	YSI	3
859	Cream cracker (LU Triumfo, Brazil)	Brazil	2002	64±11	10	Normal, 11-14	50	75	Glucose, 2h	Standard	Capillary, plasma	Enzymatic	53
860	Grain cracker (Bestore, Wuhan, China)	China	2020	25±2	4	Normal, 12	50	100	Glucose, 2h	Standard	Capillary, plasma	Enzymatic	UO[18]
861	Bread stick, Grissini Fibra Activa (with psyllium and oat) (Panmonviso, Italy)	Italy	2008	52±5	8	Normal, 12	50	110	Glucose, 2h	Standard	Capillary, whole blood	YSI	3
862	Bread stick, Grissini classici friabili (Mulino Bianco, Barilla, Italy)	Italy	2004	66±7	10	Normal, 10	50	68	Glucose, 2h	Standard	Capillary, whole blood	YSI	3
863	Protein cracker, Cheese (Herman Brot Pty Ltd, Australia)	Australia	2019	22±2	3	Normal, 10	25	174.6	Glucose, 2h	Standard	Capillary, plasma	Enzymatic	UO[5]

864	Protein cracker, Mediterranean (Herman Brot Pty Ltd, Australia)	Australia	2019	31±3	5	Normal, 10	25	116.8	Glucose, 2h	Standard	Capillary, plasma	Enzymatic	UO[5]

Food Number and Item		Country of food production	Year of test[1]	GI[2] ± SEM (Glu = 100)	GL	Subjects (type & number)	Avail carb (Test portion)	Test portion[3] (g)	Reference food & time period	Timepoints	Sample collection	Sample analysis method[4]	Ref.
865	Protein cracker, Natural (Herman Brot Pty Ltd, Australia)	Australia	2019	24±3	4	Normal, 10	25	139.1	Glucose, 2h	Standard	Capillary, plasma	Enzymatic	UO[5]
866	Protein cracker, Olive (Herman Brot Pty Ltd, Australia)	Australia	2019	29±4	4	Normal, 10	25	102.3	Glucose, 2h	Standard	Capillary, plasma	Enzymatic	UO[5]
867	Protein cracker, Spicy (Herman Brot Pty Ltd, Australia)	Australia	2019	26±3	4	Normal, 10	25	100.1	Glucose, 2h	Standard	Capillary, plasma	Enzymatic	UO[5]
868	Protein cracker, Zatar (Herman Brot Pty Ltd, Australia)	Australia	2019	27±3	4	Normal, 10	25	106.8	Glucose, 2h	Standard	Capillary, plasma	Enzymatic	UO[5]
	Protein cracker (Herman Brot), mean of six foods			27									
869	High-calcium cracker (Jacob's, Malaysia)	Malaysia	2002	52±8	8	Normal, 11-14	50	72	Glucose, 2h	Standard	Capillary, plasma	Enzymatic	53
870	Melba toast/Rusk, wheat flour (Fette Biscottate Le Dorate, [6] Italy)	Italy	2005	64±11	10	Normal, 10	50	66.3	Glucose, 2h	Standard	Capillary, whole blood	YSI	3
871	Melba toast/Rusk, soft wheat flour, whole-meal barley flour (10%), rolled oat (2%) (Fette biscottate Cuori di orzo, Mulino Bianco, Barilla, Italy)	Italy	2012	45±5	7	Normal, 9	50	72	Glucose, 2h	Standard	Capillary, whole blood	YSI	3
872	Melba toast/Rusk, soft wheat flour, bran and rolled oat, with psyllium and soluble fibers (Fette biscottate Fibra Activa, Panmonviso, Italy)	Italy	2009	47±6	7	Normal, 10	50	111	Glucose, 2h	Standard	Capillary, whole blood	YSI	3
873	Melba Toast/Rusk, whole-meal wheat flour (50.1%), soft wheat flour (Fette Biscottate Le Integrali, Mulino Bianco, Barilla, Italy)	Italy	2005	73±9	11	Normal, 9	50	72	Glucose, 2h	Standard	Capillary, whole blood	YSI	3
874	Melba Toast/Rusk made with rice, oat, wheat, corn, barley and rye (Fette Biscottate Le cereal,(Mulino Bianco, Barilla, Italy)	Italy	2007	69±8	10	Normal, 9	50	67	Glucose, 2h	Standard	Capillary, whole blood	YSI	UO[14]
875	9 Grain cracker	Australia	2013	68±5	10	Normal, 10	50	80.3	Glucose, 2h	Standard	Capillary, plasma	Enzymatic	UO[5]

876	Ancient Grains snack crackers, President's Choice® Blue Menu™ (Loblaw Brands Limited, Canada)	Canada	2004-2007	65±6	10	Normal, 10	50	NS	Bread, 2h	Standard	Capillary, whole blood	YSI	UO[7]
877	Wheat & Onion snack crackers, President's Choice® Blue Menu™ (Loblaw Brands Limited, Canada)	Canada	2004-2007	60±6	9	Normal, 10	50	NS	Bread, 2h	Standard	Capillary, whole blood	YSI	UO[7]
878	Wheat & Sesame snack crackers, President's Choice® Blue Menu™ (Loblaw Brands Limited, Canada)	Canada	2004-2007	56±7	8	Normal, 10	50	NS	Bread, 2h	Standard	Capillary, whole blood	YSI	UO[7]
879	Wheat snack crackers, President's Choice® Blue Menu™ (Loblaw Brands Limited, Canada)	Canada	2004-2007	65±7	10	Normal, 10	50	NS	Bread, 2h	Standard	Capillary, whole blood	YSI	UO[7]

Food Number and Item		Country of food production	Year of test[1]	GI[2] ± SEM (glu = 100)	GL	Subjects (type & number)	Avail carb (Test portion)	Test portion[3] (g)	Reference food & time period	Timepoints	Sample collection	Sample analysis method[4]	Ref.
880	Puffed rice cakes, Calrose rice, low-amylose (Rice Growers Co-op, Australia)	Australia	1995	91±7	14	Normal, 9	50	61	Bread, 2h	0,15,30,45,60,75,90,105, 120	Capillary, plasma	Enzymatic	70
	Puffed rice cakes, Doongara rice, high-amylose (Rice 881 Growers Co-op, Australia)	Australia	1995	61±5	9	Normal, 9	50	63	Bread, 2h	0,15,30,45,60,75,90,105, 120	Capillary, plasma	Enzymatic	70
882	Puffed rice cakes, caramel flavored	USA	2002	82±10	12	Normal, 10	25	28.1	Glucose, 2h	Standard	Capillary, whole blood	HemoCue	UO[5]
883	Rice cracker, plain (Sakada, Japan)	Japan	2003	91±11	14	Normal, 10	50	NS	Rice[17], 2h	Standard	Capillary, whole blood	Glucometer[9]	45
884	Rye crackers with oats	UK	2006	64±11	10	Normal, 10	50	78.6	Glucose, 2h	Standard	Capillary, whole blood	Glucometer[9]	30
885	Rye crackers with sesame	UK	2006	57±12	9	Normal, 10	50	77.0	Glucose, 2h	Standard	Capillary, whole blood	Glucometer[9]	30
886	Pumpkin Seeds & Oats cracker, Ryvita® (George Weston Foods, North Ryde, Australia)	Australia	2007	46±7	7	Normal, 10	50	NS	Glucose, 2h	Standard	Capillary, whole blood	HemoCue	UO[15]
887	Sunflower Seeds & Oats cracker, Ryvita® (George Weston Foods, North Ryde, Australia)	Australia	2007	48±8	7	Normal, 10	50	NS	Glucose, 2h	Standard	Capillary, whole blood	HemoCue	UO[15]
888	Wholewheat crackers with pumpkin and thyme	UK	2006	36±3	5	Normal, 10	50	81.6	Glucose, 2h	Standard	Capillary, whole blood	Glucometer[9]	30

Food Number	and Item	Country of food production	Year of test[1]	GI[2] ± SEM (Glu = 100)	GL	Subjects (type & number)	Avail carb (Test portion)	Test portion[3] (g)	Reference food & time period	Timepoints	Sample collection	Sample analysis method[4]	Ref.
889	Wholegrain crackers with sesame seeds and rosemary	UK	2006	53±8	8	Normal, 10	50	80.3	Glucose, 2h	Standard	Capillary, whole blood	Glucometer[9]	30
890	Wholewheat sticks, crunchy, yeast extract flavored	UK	2006	50±8	8	Normal, 10	50	87.3	Glucose, 2h	Standard	Capillary, whole blood	Glucometer[9]	30
	**DAIRY PRODUCTS AND ALTERNATIVES**												
	Average available carbohydrate portion = 10 g, this value was used to determine the nominal GL for each item in this category, except flavored or sweetened dairy products and soy-based alternatives where 20 g was used.												
	**Custard**												
891	Custard, low-fat, vanilla flavored (Nestlé, Australia)	Australia	20062007	29±4	6	Normal, 10	50	520.8	Glucose, 2h	Standard	Capillary, whole blood	HemoCue	UO[10]
892	No Bake Egg Custard, prepared from powder with whole milk (Nestlé, Australia)	Australia	1999	35±2	7	Normal, 10	25	NS	Glucose, 2h	Standard	Capillary, whole blood	HemoCue	UO[5]
893	Reduced-fat custard, TRIM™ (Pauls Ltd, Brisbane, Australia)	Australia	1999	37±4	7	Normal, 10	50	347.2	Bread, 2h	Standard	Capillary, whole blood	HemoCue	UO[5]
894	Custard, low-fat, trifle flavored (Nestlé, Australia)	Australia	2006-2007	30±3	6	Normal, 11	50	625.0	Glucose, 2h	Standard	Capillary, whole blood	HemoCue	UO[10]
	Custard, mean of four foods			33									
	**Crème Fraiche**												
895	Crème fraiche dessert, peach (Finest, UK)	UK	2005	28±8	6	Normal, 9	25	320.5	Glucose, 2h	Standard	Capillary, whole blood	Glucometer[9]	26
896	Crème fraiche dessert, raspberry (Finest, UK)	UK	2005	30±7	6	Normal, 10	25	446.4	Glucose, 2h	Standard	Capillary, whole blood	Glucometer[9]	26
	**Fromage Frais**												
897	Fromage Frais, red fruit: blackcurrant (Healthy Living, UK)	UK	2005	22±5	4	Normal, 10	25	416.7	Glucose, 2h	Standard	Capillary, whole blood	Glucometer[9]	26
898	Fromage Frais, red fruit: raspberry (Healthy Living, UK)	UK	2005	31±8	6	Normal, 10	25	441.2	Glucose, 2h	Standard	Capillary, whole blood	Glucometer[9]	26
899	Fromage Frais, red fruit: red cherry (Healthy Living, UK)	UK	2005	25±6	5	Normal, 10	25	416.7	Glucose, 2h	Standard	Capillary, whole blood	Glucometer[9]	26
900	Fromage Frais, red fruit: strawberry (Healthy Living, UK)	UK	2005	29±9	6	Normal, 9	25	454.5	Glucose, 2h	Standard	Capillary, whole blood	Glucometer[9]	26

901	Fromage Frais, yellow fruit: mandarin and orange (Healthy Living, UK)	UK	2005	19±6	4	Normal, 9	25	428.6	Glucose, 2h	Standard	Capillary, whole blood	Glucometer[9]	26
902	Fruche®, low-fat, apricot vanilla honey (National Foods Ltd, Melbourne, Australia)	Australia	2005	34±5	7	Normal, 10	50	337.8	Glucose, 2h	Standard	Capillary, whole blood	HemoCue	UO[5]
	Fruche®, low-fat, berry compote (National Foods Ltd, 903 Melbourne, Australia)	Australia	2005	37±6	7	Normal, 10	50	357.1	Glucose, 2h	Standard	Capillary, whole blood	HemoCue	UO[5]
904	Fruche®, low-fat, duet peach on pineapple (National Foods Ltd, Melbourne, Australia)	Australia	2005	34±5	7	Normal, 10	50	340.1	Glucose, 2h	Standard	Capillary, whole blood	HemoCue	UO[5]
	Fruche®, low-fat, lemon sorbetto (National Foods Ltd, 905 Melbourne, Australia)	Australia	2005	34±3	7	Normal, 10	50	342.5	Glucose, 2h	Standard	Capillary, whole blood	HemoCue	UO[5]
	Fruche®, low-fat, orange sorbetto (National Foods Ltd, 906 Melbourne, Australia)	Australia	2005	34±3	7	Normal, 10	50	359.7	Glucose, 2h	Standard	Capillary, whole blood	HemoCue	UO[5]
	Fruche®, low-fat, peach roulade (National Foods Ltd, 907 Melbourne, Australia)	Australia	2005	37±6	7	Normal, 10	50	352.1	Glucose, 2h	Standard	Capillary, whole blood	HemoCue	UO[5]
908	Fruche® on Fruit, low-fat, toffee apple (National Foods Ltd, Melbourne, Australia)	Australia	2005	47±6	9	Normal, 10	50	226.2	Glucose, 2h	Standard	Capillary, whole blood	HemoCue	UO[5]
	Fromage frais, mean of 12 foods			32									

Food Number and Item		Country of food production	Year of test[1]	GI[2] ± SEM (Glu = 100)	GL	Subjects (type & number)	Avail carb (Test portion)	Test portion[3] (g)	Reference food & time period	Timepoints	Sample collection	Sample analysis method[4]	Ref.
	**Ice cream, Reduced- or Low-fat**												
909	Ice-cream, low-fat chocolate flavored, Chocollo™ (Wendy's Supa Sundaes Pty Ltd, Australia)	Australia	2006	24±6	5	Normal, 10	50	331.1	Glucose, 2h	Standard	Capillary, whole blood	HemoCue	UO[5]
910	Ice cream, low-fat, Bulla Light Creamy vanilla (Regal Cream Products, VIC, Australia)	Australia	2005	36±5	7	Normal, 10	50	193.8	Glucose, 2h	Standard	Capillary, whole blood	HemoCue	UO[5]
911	Ice cream, low-fat, Bulla Light Real Dairy chocolate (Regal Cream Products, VIC, Australia)	Australia	2005	27±3	5	Normal, 10	50	194.6	Glucose, 2h	Standard	Capillary, whole blood	HemoCue	UO[5]
912	Ice cream, low-fat, Bulla Light Real Dairy English Toffee (Regal Cream Products, VIC, Australia)	Australia	2005	27±3	5	Normal, 10	50	198.4	Glucose, 2h	Standard	Capillary, whole blood	HemoCue	UO[5]

Food Number and Item		Country of food production	Year of test[1]	GI[2] ± SEM (Glu = 100)	GL	Subjects (type & number)	Avail carb (Test portion)	Test portion[3] (g)	Reference food & time period	Timepoints	Sample collection	Sample analysis method[4]	Ref.
913	Ice cream, low-fat, Bulla Light Real Dairy Mango (Regal Cream Products, Australia)	Australia	2005	30±4	6	Normal, 10	50	196.9	Glucose, 2h	Standard	Capillary, whole blood	HemoCue	UO[5]
	Ice cream, low-fat, Light & Creamy, Raspberry Ripple 914 (Nestlé Peter's, Australia)	Australia	2004	55±3	11	Normal, 10	50	160.8	Glucose, 2h	Standard	Capillary, whole blood	HemoCue	UO[5]
	Ice-cream, low-fat,1.2% fat, Light Prestige rich vanilla 915 (Norco, Australia)	Australia	2001	47±5	9	Normal, 10	25	133.0	Glucose, 2h	Standard	Capillary, whole blood	HemoCue	UO[5]
916	Ice-cream, low-fat, 1.4% fat, Light Prestige traditional toffee (Norco, Australia)	Australia	2001	37±4	7	Normal, 10	25	106.4	Glucose, 2h	Standard	Capillary, whole blood	HemoCue	UO[5]
917	Ice-cream, reduced-fat, 7.1% fat, Prestige golden macadamia (Norco, Australia)	Australia	2001	37±3	7	Normal, 10	25	118.5	Glucose, 2h	Standard	Capillary, whole blood	HemoCue	UO[5]
918	Ice cream, low-fat, vanilla, 'Light' (Nestlé Peter's, Australia)	Australia	2003	46±5	9	Normal, 10	50	172.4	Glucose, 2h	Standard	Capillary, whole blood	HemoCue	UO[5]
919	Ice cream, low-fat, Blueberry Pomegranate flavor (ProYo, USA)	USA	2017	32±4	6	Normal, 10	50	NS	Bread, 2h	Standard	Capillary, whole blood	YSI	UO[7]
920	Ice cream, low-fat, Chocolate Toffee flavor (ProYo, USA)	USA	2017	25±2	5	Normal, 10	50	NS	Bread, 2h	Standard	Capillary, whole blood	YSI	UO[7]
921	Ice cream, low-fat, Coconut flavor (ProYo, USA)	USA	2017	39±5	8	Normal, 10	50	NS	Bread, 2h	Standard	Capillary, whole blood	YSI	UO[7]
922	Ice cream, low-fat, Dutch Chocolate flavor (ProYo, USA)	USA	2017	34±6	7	Normal, 10	50	NS	Bread, 2h	Standard	Capillary, whole blood	YSI	UO[7]
923	Ice cream, low-fat, Mint Chip flavor (ProYo, USA)	USA	2017	34±6	7	Normal, 10	50	NS	Bread, 2h	Standard	Capillary, whole blood	YSI	UO[7]
924	Ice cream, low-fat, Mocha flavor (ProYo, USA)	USA	2017	37±5	7	Normal, 10	50	NS	Bread, 2h	Standard	Capillary, whole blood	YSI	UO[7]
925	Ice cream, low-fat, Vanilla Bean flavor (ProYo, USA)	USA	2017	39±5	8	Normal, 10	50	NS	Bread, 2h	Standard	Capillary, whole blood	YSI	UO[7]
	Ice-cream, reduced- or low-fat, mean of 17 foods			36									

	**Ice cream, Premium (high-fat)**												
926	Ice cream, premium, Ultra chocolate, 15% fat (Sara Lee, Gosford, NSW, Australia)	Australia	2000	37±3	7	Normal, 10	50	263.2	Glucose, 2h	Standard	Capillary, plasma	Enzymatic	UO[5]
927	Ice cream, premium, French vanilla, 16% fat (Sara Lee, Australia)	Australia	2000	38±3	8	Normal, 10	50	277.8	Glucose, 2h	Standard	Capillary, plasma	Enzymatic	UO[5]
	**Other ice cream**												
928	Ice-cream, , low-fat chocolate flavored Chocollo™, eaten with a plain cone (Wendy's Supa Sundaes Pty Ltd, Australia)	Australia	2006	44±7	9	Normal, 10	50	276.1	Glucose, 2h	Standard	Capillary, whole blood	HemoCue	UO[5]
929	Ice-cream, low-fat chocolate flavored, Chocollo™, eaten with a waffle cone (Wendy's Supa Sundaes Pty Ltd, Australia)	Australia	2006	55±6	11	Normal, 10	50	199.9	Glucose, 2h	Standard	Capillary, whole blood	HemoCue	UO[5]
930	Gelati, sucrose-free frozen dessert, chocolate flavor (Alba Foods, Newtown, Australia)	Australia	2002	37±5	7	Normal, 10	50	185.9	Glucose, 2h	Standard	Capillary, plasma	Enzymatic	UO[5]
931	Gelati, sucrose-free frozen dessert, vanilla flavor (Alba Foods, Newtown, Australia)	Australia	2002	39±4	8	Normal, 10	50	182.5	Glucose, 2h	Standard	Capillary, plasma	Enzymatic	UO[5]
932	Ice fruit confection, Frosty Fruits® Orange Mango Splits (Nestlé Peter's, Australia)	Australia	2007	59±4	12	Normal, 9	50	216.5	Glucose, 2h	Standard	Capillary, plasma	Enzymatic	UO[5]
	**Kefir**												
933	Kefir, low-fat, plain (Lifeway Foods, Chicago, USA)	USA	2012*	36±9	4	Normal, 10	25	667	Glucose, 2h	Standard	Capillary, serum	Enzymatic	83
934	Kefir, low-fat strawberry (Lifeway Foods, Chicago, USA)	USA	2012*	60±10	12	Normal, 10	50	546	Glucose, 2h	Standard	Capillary, serum	Enzymatic	83
935	Kefir, orange flavor (Lifeway Foods, Chicago, USA)	USA	2012*	48±10	10	Normal, 10	50	548	Glucose, 2h	Standard	Capillary, serum	Enzymatic	83
	**Milk condensed**												
936	Milk, condensed, sweetened (Nestlé, Australia)	Australia	2000	61±6	6	Normal, 11	50	90.2	Glucose, 2h	Standard	Capillary, plasma	Enzymatic	74
	**Milk, full-fat**												
937	Full-fat, milk	Australia	2018	30±3	3	Normal, 13	25	548.5	Glucose, 2h	Standard	Capillary, plasma	Enzymatic	UO[5]
938	Full-fat, pasteurized, fresh, organic milk (Arla, UK)	UK	2005	34±6	3	Normal, 9	25	520.8	Glucose, 2h	Standard	Capillary, whole blood	Glucometer[9]	26

Food Number and Item		Country of food production	Year of test[1]	GI[2] ± SEM (Glu = 100)	GL	Subjects (type & number)	Avail carb (Test portion)	Test portion[3] (g)	Reference food & time period	Timepoints	Sample collection	Sample analysis method[4]	Ref.
939	Full-fat, standardized, homogenized, pasteurized, British (Dairy Crest, UK)	UK	2005	46±10	5	Normal, 10	25	531.9	Glucose, 2h	Standard	Capillary, whole blood	Glucometer[9]	26
	Milk, full-fat, mean of three studies			37									
	**Milk, full-fat, flavored**												
940	Banana, honey and malt flavored milk	Australia	2002	31±4	6	Normal, 10	50	555.5	Glucose, 2h	Standard	Capillary, whole blood	HemoCue	UO[5]
941	Coffee chocolate flavored milk, Masters® mocha (National Foods Ltd, Australia)	Australia	2005	32±4	6	Normal, 10	50	510.2	Glucose, 2h	Standard	Capillary, whole blood	HemoCue	UO[5]
	**Milk, reduced-fat, low fat or skim**												
942	Milk, reduced fat (Dairy Farmers Ltd, Australia)	Australia	2006	26±6	3	Normal, 10	25	428.2	Glucose, 2h	Standard	Capillary, whole blood	HemoCue	UO[5]
943	Milk, semi-skimmed, pasteurized, organic (Arla, UK)	UK	2005	34±8	3	Normal, 9	25	500	Glucose, 2h	Standard	Capillary, whole blood	Glucometer[9]	26
944	Pura™ HiLo milk (National Foods Ltd, Australia)	Australia	2006	20±5	2	Normal, 10	50	862.1	Glucose, 2h	Standard	Capillary, whole blood	HemoCue	UO[5]
945	Pura™ Skimmer milk (National Foods Ltd, Australia)	Australia	2006	20±5	2	Normal, 10	50	862.1	Glucose, 2h	Standard	Capillary, whole blood	HemoCue	UO[5]
946	Pura™ Light Start milk (National Foods Ltd, Australia)	Australia	2005	30±5	3	Normal, 10	50	479.7	Glucose, 2h	Standard	Capillary, whole blood	HemoCue	UO[5]
947	Pura™ Tone milk (National Foods Ltd, Australia)	Australia	2005	30±5	3	Normal, 10	50	419.7	Glucose, 2h	Standard	Capillary, whole blood	HemoCue	UO[5]
	Reduced-fat or low-fat milk, mean of six studies			27									
	**Milk, reduced- or low-fat, flavored**												
948	Flavored milk, chocolate or strawberry, Big M™ (National Foods Ltd, Australia)	Australia	2005	37±4	7	Normal, 10	25	277.8	Glucose, 2h	Standard	Capillary, whole blood	HemoCue	UO[5]
949	Flavored milk, chocolate or mocha, Masters® Light, 99% fat free (National Foods Ltd, Australia)	Australia	2005	27±5	5	Normal, 10	50	561.8	Glucose, 2h	Standard	Capillary, whole blood	HemoCue	UO[5]

Food Number and Item	Country of food production	Year of test[1]	GI[2] ± SEM (Glu = 100)	GL	Subjects (type & number)	Avail carb (Test portion)	Test portion[3] (g)	Reference food & time period	Timepoints	Sample collection	Sample analysis method[4]	Ref.
950 Flavored milk, chocolate or strawberry, Masters®, reduced fat, (National Foods Ltd, Australia)	Australia	2005	35±5	7	Normal, 10	50	531.9	Glucose, 2h	Standard	Capillary, whole blood	HemoCue	UO[5]
951 Flavored milk, Rush® Heavenly Vanilla Malt milk	Australia	2003	31±4	6	Normal, 10	50	490.2	Glucose, 2h	Standard	Capillary, whole blood	HemoCue	UO[5]
952 Flavored milk, Rush® Intense Coffee milk	Australia	2005	24±3	5	Normal, 10	50	457.0	Glucose, 2h	Standard	Capillary, whole blood	HemoCue	UO[5]
953 Flavored milk, Rush® Ultimate Chocolate milk	Australia	2003	26±5	5	Normal, 10	50	537.6	Glucose, 2h	Standard	Capillary, whole blood	HemoCue	UO[5]
954 Flavored milk, Rush® Wicked Latte milk	Australia	2005	26±3	5	Normal, 10	50	473.2	Glucose, 2h	Standard	Capillary, whole blood	HemoCue	UO[5]
Milk, reduced- or low-fat, flavored, mean of seven foods			29									
**Milkshake**												
955 Milkshake, made from Chocollo™, low-fat chocolate ice cream, skim milk and malted milk powder (Wendy's Supa Sundaes Pty Ltd, Australia)	Australia	2006	21±4	4	Normal, 9	50	423.2	Glucose, 2h	Standard	Capillary, whole blood	HemoCue	UO[5]
**Mousse**												
956 Nestlé Milo Combo mousse (Nestlé, Australia)	Australia	2006	42±5	8	Normal, 11	50	289.7	Glucose, 2h	Standard	Capillary, whole blood	HemoCue	UO[10]
957 Nestlé Milo flavored mousse (Nestlé, Australia)	Australia	2006	46±3	9	Normal, 12	50	254.8	Glucose, 2h	Standard	Capillary, whole blood	HemoCue	UO[10]
958 Butterscotch, 1.9% fat (Nestlé, Australia), prepared from commercial mousse mix with water	Australia	2000	36±4	7	Normal, 10	50	82.5	Glucose, 2h	Standard	Capillary, plasma	Enzymatic	UO[5]
959 Chocolate, 2% fat (Nestlé, Australia), prepared from commercial mousse mix with water	Australia	1999	31±4	6	Normal, 10	50	NS	Glucose, 2h	Standard	Capillary, plasma	Enzymatic	UO[5]
960 Citrus (Nestlé, Australia), prepared from commercial mousse mix with water	Australia	2005	47±5	9	Normal, 10	50	NS	Glucose, 2h	Standard	Capillary, plasma	Enzymatic	UO[5]
961 Hazelnut, 2.4% fat (Nestlé, Australia), prepared from commercial mousse mix with water	Australia	2000	36±4	7	Normal, 10	50	81.3	Glucose, 2h	Standard	Capillary, plasma	Enzymatic	UO[5]
962 Mango, 1.8% fat (Nestlé, Australia), prepared from commercial mousse mix with water	Australia	2000	33±5	7	Normal, 10	50	76.4	Glucose, 2h	Standard	Capillary, plasma	Enzymatic	UO[5]

963 Mixed berry, 2.2% fat (Nestlé, Australia), prepared from commercial mousse mix with water	Australia	2000	36±5	7	Normal, 10	50	81.3	Glucose, 2h	Standard	Capillary, plasma	Enzymatic	UO[5]
964 Strawberry, 2.3% fat (Nestlé, Australia), prepared from commercial mousse mix with water	Australia	2000	32±3	6	Normal, 10	50	82.5	Glucose, 2h	Standard	Capillary, plasma	Enzymatic	UO[5]
Mousse, reduced-fat, mean of nine foods			38									
**Pudding**												
965 Flavored milk pudding, chocolate (Danette al cioccolato, Danone, Italy)	Italy	2011	51±5	10	Normal, 9	25	152	Glucose, 2h	Standard	Capillary, whole blood	YSI	3
966 Flavored milk pudding, chocolate, made from powder and whole milk, instant (White Wings, Australia)	Australia	2000	47±4	9	Normal, 10	50	NS	Glucose, 2h	Standard	Capillary, plasma	Enzymatic	UO[5]
967 Flavored milk pudding, vanilla, made from powder and whole milk, instant (White Wings, Australia)	Australia	2000	40±4	8	Normal, 10	50	NS	Glucose, 2h	Standard	Capillary, plasma	Enzymatic	UO[5]
Flavored milk pudding, mean of three foods			46									
968 Milk rice pudding, Yoplait Le Rice®, Apple & Cinnamon flavor (National Foods Ltd, Australia)	Australia	2005	52±4	10	Normal, 10	50	238.1	Glucose, 2h	Standard	Capillary, whole blood	HemoCue	UO[5]

Food Number and Item	Country of food production	Year of test[1]	GI[2] ± SEM (Glu = 100)	GL	Subjects (type & number)	Avail carb (Test portion)	Test portion[3] (g)	Reference food & time period	Timepoints	Sample collection	Sample analysis method[4]	Ref.
969 Milk rice pudding, Yoplait Le Rice®, Apricot & Almond Muesli flavor (National Foods Ltd, Australia)	Australia	2006	45±4	9	Normal, 10	50	231.5	Glucose, 2h	Standard	Capillary, whole blood	HemoCue	UO[5]
Milk rice pudding, Yoplait Le Rice®, Caramel flavor 970 (National Foods Ltd, Australia)	Australia	2005	41±6	8	Normal, 10	50	235.8	Glucose, 2h	Standard	Capillary, whole blood	HemoCue	UO[5]
971 Milk rice pudding, Yoplait Le Rice®, Forest Berries flavor (National Foods Ltd, Australia)	Australia	2006	45±4	9	Normal, 10	50	247.5	Glucose, 2h	Standard	Capillary, whole blood	HemoCue	UO[5]
972 Milk rice pudding, Yoplait Le Rice®, Lemon Meringue flavor (National Foods Ltd, Australia)	Australia	2010	54±4	11	Normal, 10	50	264.6	Glucose, 2h	Standard	Capillary, plasma	Enzymatic	UO[5]
973 Milk rice pudding, Yoplait Le Rice®, Mocha flavor (National Foods Ltd, Australia)	Australia	2009	46±3	9	Normal, 10	50	267.4	Glucose, 2h	Standard	Capillary, plasma	Enzymatic	UO[5]
974 Milk rice pudding, Yoplait Le Rice®, Raspberry & Apple flavor (National Foods Ltd)	Australia	2005	52±4	10	Normal, 10	50	235.8	Glucose, 2h	Standard	Capillary, whole blood	HemoCue	UO[5]

Food Number and Item		Country of food production	Year of test[1]	GI[2] ± SEM (Glu = 100)	GL	Subjects (type & number)	Avail carb (Test portion)	Test portion[3] (g)	Reference food & time period	Timepoints	Sample collection	Sample analysis method[4]	Ref.
975	Milk rice pudding, Yoplait Le Rice®, Strawberry flavor (National Foods Ltd)	Australia	2005	54±6	11	Normal, 10	50	235.8	Glucose, 2h	Standard	Capillary, whole blood	HemoCue	UO[5]
976	Milk rice pudding, Yoplait Le Rice®, Tropical Mango flavor (National Foods Ltd)	Australia	2005	54±6	11	Normal, 10	50	238.1	Glucose, 2h	Standard	Capillary, whole blood	HemoCue	UO[5]
977	Milk rice pudding, Yoplait Le Rice®, Vanilla flavor (National Foods Ltd)	Australia	2005	43±4	9	Normal, 10	50	243.9	Glucose, 2h	Standard	Capillary, whole blood	HemoCue	UO[5]
	Milk rice pudding, Yoplait Le Rice, mean of 10 foods			49									
	**Yoghurt**												
978	Yoghurt, black cherry (Finest, UK)	UK	2005	17±3	3	Normal, 10	25	312.5	Glucose, 2h	Standard	Capillary, whole blood	Glucometer[9]	26
979	Yoghurt, black cherry (Healthy Living Light, UK)	UK	2005	67±15	13	Normal, 10	25	287.4	Glucose, 2h	Standard	Capillary, whole blood	Glucometer[9]	26
980	Yoghurt, bourbon vanilla (Finest, UK)	UK	2005	64±14	13	Normal, 10	25	260.4	Glucose, 2h	Standard	Capillary, whole blood	Glucometer[9]	26
981	Yoghurt, Devonshire fudge (Finest, UK)	UK	2005	37±7	7	Normal, 10	25	223.2	Glucose, 2h	Standard	Capillary, whole blood	Glucometer[9]	26
982	Yoghurt, Greek style, honey topped (UK)	UK	2005	36±7	7	Normal, 9	25	367.6	Glucose, 2h	Standard	Capillary, whole blood	Glucometer[9]	26
983	Yoghurt, Jalna Premium Creamy Vanilla (Jalna Dairy Foods, Australia)	Australia	2004	18±4	4	Normal, 10	50	331.1	Glucose, 2h	Standard	Capillary, whole blood	HemoCue	UO[5]
984	Yoghurt, lemon curd (Finest, UK)	UK	2005	67±14	13	Normal, 10	25	248.8	Glucose, 2h	Standard	Capillary, whole blood	Glucometer[9]	26
985	Yoghurt, peach & apricot (Healthy Living Light, UK)	UK	2005	28±6	6	Normal, 9	25	308.6	Glucose, 2h	Standard	Capillary, whole blood	Glucometer[9]	26
986	Yoghurt, raspberry (Healthy Living Light, UK)	UK	2005	43±8	9	Normal, 10	25	308.6	Glucose, 2h	Standard	Capillary, whole blood	Glucometer[9]	26
987	Yoghurt, red fruit: Morello cherry (Healthy Living Light, UK)	UK	2005	35±6	7	Normal, 9	25	454.5	Glucose, 2h	Standard	Capillary, whole blood	Glucometer[9]	26
988	Yoghurt, red fruit: raspberry and black cherry (Healthy Living	UK	2005	37±8	7	Normal, 9	25	491.8	Glucose, 2h	Standard	Capillary, whole blood	Glucometer[9]	26

	Light, UK)												
989	Yoghurt, Scottish raspberry (Finest, UK)	UK	2005	32±7	6	Normal, 10	25	264.6	Glucose, 2h	Standard	Capillary, whole blood	Glucometer[9]	26
990	Yoghurt, strawberry (Healthy Living Light, UK)	UK	2005	30±6	6	Normal, 10	25	316.5	Glucose, 2h	Standard	Capillary, whole blood	Glucometer[9]	26
991	Yoghurt, strawberry (Astro yoghurt, Canada)	Canada	2010*	44±4	9	Normal, 10	50	250	Bread, 2h	Standard	Capillary, whole blood	YSI	56
992	Yoghurt, strawberry and cream (Finest, UK)	UK	2005	41±6	8	Normal, 10	25	324.7	Glucose, 2h	Standard	Capillary, whole blood	Glucometer[9]	26
993	Yoghurt, summer fruit: apricot (Healthy Living Light, UK)	UK	2005	11±4	2	Normal, 9	25	476.2	Glucose, 2h	Standard	Capillary, whole blood	Glucometer[9]	26
994	Yoghurt, summer fruit: peach and vanilla (Healthy Living Light, UK)	UK	2005	26±7	5	Normal, 9	25	476.2	Glucose, 2h	Standard	Capillary, whole blood	Glucometer[9]	26
995	Yoghurt, summer fruit: raspberry (Healthy Living Light, UK)	UK	2005	28±5	6	Normal, 9	25	501.0	Glucose, 2h	Standard	Capillary, whole blood	Glucometer[9]	26
996	Yoghurt, summer fruit: strawberry (Healthy Living Light, UK)	UK	2005	36±9	7	Normal, 9	25	500.0	Glucose, 2h	Standard	Capillary, whole blood	Glucometer[9]	26
997	Yoghurt, tropical fruit: guava and passionfruit (Healthy Living Light, UK)	UK	2005	24±7	5	Normal, 9	25	447.8	Glucose, 2h	Standard	Capillary, whole blood	Glucometer[9]	26
998	Yoghurt, tropical fruit: mango (Healthy Living Light, UK)	UK	2005	32±8	6	Normal, 9	25	454.5	Glucose, 2h	Standard	Capillary, whole blood	Glucometer[9]	26
999	Yoghurt, tropical fruit: peach and apricot (Healthy Living Light, UK)	UK	2005	27±6	5	Normal, 9	25	468.8	Glucose, 2h	Standard	Capillary, whole blood	Glucometer[9]	26
1000	Yoghurt, tropical pineapple (Healthy Living Light, UK)	UK	2005	38±7	8	Normal, 9	25	461.5	Glucose, 2h	Standard	Capillary, whole blood	Glucometer[9]	26
1001	Yoghurt, Valencia orange (Finest, UK)	UK	2005	34±7	7	Normal, 10	25	301.2	Glucose, 2h	Standard	Capillary, whole blood	Glucometer[9]	26
1002	Yoghurt, Yitangping (Beijing Sanyuan Foods, Beijing, China)	China	2020	21±4	4	Normal, 11	25	454.5	Glucose, 2h	Standard	Capillary, plasma	Enzymatic	UO[18]
	Yoghurt, different flavors, mean of 25 studies			35									

Food Number and Item		Country of food production	Year of test[1]	GI[2] ± SEM (Glu = 100)	GL	Subjects (type & number)	Avail carb (Test portion)	Test portion[3] (g)	Reference food & time period	Timepoints	Sample collection	Sample analysis method[4]	Ref.
	**Yoghurt, frozen**												
1003	Mochaccino frozen yoghurt, President's Choice® Blue Menu™ brand (Loblaw Brands Limited, Canada)	Canada	20042007	51±5	10	Normal, 10	50	NS	Bread, 2h	Standard	Capillary, whole blood	YSI	UO[7]
1004	Strawberry Banana frozen yoghurt, President's Choice® Blue Menu™ brand (Loblaw Brands Limited, Canada)	Canada	2004-2007	55±6	11	Normal, 10	50	NS	Bread, 2h	Standard	Capillary, whole blood	YSI	UO[7]
1005	Vanilla frozen yoghurt, President's Choice® Blue Menu™ brand (Loblaw Brands Limited, Canada)	Canada	2004-2007	46±5	9	Normal, 10	50	NS	Bread, 2h	Standard	Capillary, whole blood	YSI	UO[7]
	Yoghurt, frozen, mean of three foods			51									
	**Yoghurt, low-fat, reduced fat, or non-fat**												
1006	Diet Passionfruit yoghurt, Dairy Dream (Aldi, Australia)	Australia	2010	21±2	4	Normal, 10	50	495	Glucose, 2h	Standard	Capillary, plasma	Enzymatic	UO[5]
1007	Diet Raspberry yoghurt, Dairy Dream (Aldi, Australia)	Australia	2010	21±2	4	Normal, 10	50	515	Glucose, 2h	Standard	Capillary, plasma	Enzymatic	UO[5]
1008	Diet yoghurt, Vaalia™, exotic fruits (Pauls Ltd., Australia), sweetened with acesulfame K and Splenda	Australia	2000	23±2	5	Normal, 10	25	312.5	Glucose, 2h	Standard	Capillary, plasma	Enzymatic	UO[5]
1009	Diet yoghurt, Vaalia™, mango (Pauls Ltd., Australia), sweetened with acesulfame K and Splenda	Australia	2000	23±2	5	Normal, 10	25	367.6	Glucose, 2h	Standard	Capillary, plasma	Enzymatic	UO[5]
1010	Diet yoghurt, Vaalia™, mixed berry (Pauls Ltd., Australia), sweetened with acesulfame K and Splenda	Australia	2000	25±3	5	Normal, 10	25	409.8	Glucose, 2h	Standard	Capillary, plasma	Enzymatic	UO[5]
1011	Diet yoghurt, Vaalia™, strawberry (Pauls Ltd., Australia), sweetened with acesulfame K and Splenda	Australia	2000	23±2	5	Normal, 10	25	423.7	Glucose, 2h	Standard	Capillary, plasma	Enzymatic	UO[5]
1012	Diet yoghurt, Vaalia™, vanilla (Pauls Ltd, Australia) sweetened with acesulfame K and Splenda	Australia	2000	23±2	5	Normal, 10	25	416.7	Glucose, 2h	Standard	Capillary, plasma	Enzymatic	UO[5]
1013	Low-fat, sugar sweetened, strawberry classic, Yoplait™ Lite (National Foods Ltd, Australia)	Australia	2002	33±5	7	Normal, 10	50	324.7	Glucose, 2h	Standard	Capillary, whole blood	HemoCue	UO[5]
1014	Low-fat (0.9%), fruit, wild strawberry, Ski d'lite™ (Dairy Farmers Ltd, Australia)	Australia	2002	31±4	6	Normal, 9	50	342.5	Glucose, 2h	Standard	Capillary, whole blood	HemoCue	UO[5]
1015	Low-fat, Nestlé Diet, Blueberry (Nestlé, Australia)	Australia	2006	32±4	6	Normal, 10	25	446.4	Glucose, 2h	Standard	Capillary, whole blood	HemoCue	UO[10]

Food Number and Item		Country of food production	Year of test[1]	GI[2] ± SEM (Glu = 100)	GL	Subjects (type & number)	Avail carb (Test portion)	Test portion[3] (g)	Reference food & time period	Timepoints	Sample collection	Sample analysis method[4]	Ref.
1016	Low-fat, Nestlé Diet, Mixed Berry (Nestlé, Australia)	Australia	2006	28±4	6	Normal, 12	25	463.0	Glucose, 2h	Standard	Capillary, whole blood	HemoCue	UO[10]
1017	Low-fat, Nestlé Diet, Peaches & Cream (Nestlé, Australia)	Australia	2006	28±3	6	Normal, 10	25	463.0	Glucose, 2h	Standard	Capillary, whole blood	HemoCue	UO[10]
1018	Low-fat, apricot	UK	2005	42±6	8	Normal, 10	25	354.6	Glucose, 2h	Standard	Capillary, whole blood	Glucometer[9]	26
1019	Low-fat, black cherry	UK	2005	41±5	8	Normal, 10	25	349.7	Glucose, 2h	Standard	Capillary, whole blood	Glucometer[9]	26
1020	Low-fat, hazelnut	UK	2005	53±9	11	Normal, 10	25	357.1	Glucose, 2h	Standard	Capillary, whole blood	Glucometer[9]	26
1021	Low-fat, peach melba (Value, UK)	UK	2005	56±11	11	Normal, 10	25	357.1	Glucose, 2h	Standard	Capillary, whole blood	Glucometer[9]	26
1022	Low-fat, raspberry	UK	2005	34±6	7	Normal, 10	25	335.6	Glucose, 2h	Standard	Capillary, whole blood	Glucometer[9]	26
1023	Low-fat, strawberry	UK	2005	61±11	12	Normal, 10	25	347.2	Glucose, 2h	Standard	Capillary, whole blood	Glucometer[9]	26
1024	Low-fat, sugar sweetened, Heavenly Honey flavor, Vaalia (Pauls Ltd)	Australia	2003	32±4	6	Normal, 10	50	280.9	Glucose, 2h	Standard	Capillary, whole blood	HemoCue	UO[5]
	Low-fat, sugar sweetened, Lemon Crème flavor, Vaalia 1025 (Pauls Ltd)	Australia	2003	43±6	9	Normal, 10	50	307.6	Glucose, 2h	Standard	Capillary, whole blood	HemoCue	UO[5]
1026	Low-fat, sugar sweetened, Luscious Berries flavor, Vaalia (Pauls Ltd)	Australia	2003	29±2	6	Normal, 10	50	337.8	Glucose, 2h	Standard	Capillary, whole blood	HemoCue	UO[5]
1027	Low-fat, sugar sweetened, Passionfruit flavor, Vaalia (Pauls Ltd)	Australia	2003	32±3	6	Normal, 10	50	301.2	Glucose, 2h	Standard	Capillary, whole blood	HemoCue	UO[5]
1028	Low-fat, sugar sweetened, Strawberry flavor, Vaalia (Pauls Ltd)	Australia	2003	28±3	6	Normal, 9	50	340.1	Glucose, 2h	Standard	Capillary, whole blood	HemoCue	UO[5]
1029	No Added Sugar Passionfruit yoghurt, Tamar Valley (Aldi, Australia)	Australia	2010	21±2	4	Normal, 10	50	495	Glucose, 2h	Standard	Capillary, plasma	Enzymatic	UO[5]

1030	No Added Sugar Raspberry yoghurt, Tamar Valley (Aldi, Australia)	Australia	2010	21±2	4	Normal, 10	50	515	Glucose, 2h	Standard	Capillary, plasma	Enzymatic	UO[5]
	Low fat yoghurts different flavors, mean of 25 foods			32									
	Reduced-fat, Vaalia™, apricot & mango (Pauls Ltd., 1031 Australia)	Australia	1999	26±4	5	Normal, 10	25	354.6	Bread, 2h	Standard	Capillary, plasma	Enzymatic	UO[5]
1032	Reduced-fat, Vaalia™, French vanilla (Pauls Ltd., Australia)	Australia	1999	26±4	5	Normal, 10	25	292.4	Bread, 2h	Standard	Capillary, plasma	Enzymatic	UO[5]
1033	Reduced-fat, Extra-Lite™, strawberry (Pauls Ltd., Australia)	Australia	1999	28±4	6	Normal, 10	25	310.6	Bread, 2h	Standard	Capillary, plasma	Enzymatic	UO[5]
1034	Reduced-fat, strawberry	Czech Republic	2005	43±5	9	Normal, 20	50	312.5	Glucose, 2h	0,15,30,45,60,75,90,105, 120	Capillary, whole blood	Glucometer[9]	84
	Reduced-fat, Yoplait™ Lite, berry fruit flavors (National 1035 Foods Ltd, Australia)	Australia	2006	25±3	5	Normal, 10	50	290.7	Glucose, 2h	Standard	Capillary, whole blood	HemoCue	UO[5]

**Food Number and Item**		**Country of food production**	**Year of test[1]**	**GI[2] ± SEM (Glu = 100)**	**GL**	**Subjects (type & number)**	**Avail carb (Test portion)**	**Test portion[3] (g)**	**Reference food & time period**	**Timepoints**	**Sample collection**	**Sample analysis method[4]**	**Ref.**
1036	Reduced-fat, Yoplait™ Lite, tropical fruit flavors (National Foods Ltd, Australia)	Australia	2006	37±3	7	Normal, 10	50	312.5	Glucose, 2h	Standard	Capillary, whole blood	HemoCue	UO[5]
1037	Reduced-fat, Yoplait™ Lite, various flavors (National Foods Ltd, Australia)	Australia	2006	27±2	5	Normal, 10	50	271.7	Glucose, 2h	Standard	Capillary, whole blood	HemoCue	UO[5]
	Reduced-fat, Yoplait™ Lite, Fruit Salad flavor (National 1038 Foods Ltd, Australia)	Australia	2006	32±5	6	Normal, 10	50	324.7	Glucose, 2h	Standard	Capillary, whole blood	HemoCue	UO[5]
	Reduced-fat yoghurts different flavors, mean of nine foods			30									
	Non-fat, Honey flavor, sugar sweetened, Ski™ (Dairy 1039 Farmers Ltd, Australia),	Australia	2002	40±2	8	Normal, 10	50	287.4	Glucose, 2h	Standard	Capillary, plasma	Enzymatic	UO[5]
	Non-fat, Vanilla flavor, sugar sweetened, Ski™, (Dairy 1040 Farmers Ltd, Australia),	Australia	2002	40±4	8	Normal, 10	50	316.5	Glucose, 2h	Standard	Capillary, Whole blood	HemoCue	UO[5]
1041	No Fat berry fruit flavors, artificially sweetened yoghurts, Yoplait™ (National Foods Ltd, Australia)	Australia	2006	16±3	3	Normal, 10	25	384.6	Glucose, 2h	Standard	Capillary, Whole blood	HemoCue	UO[5]
1042	No Fat Strawberry Classic flavor, artificially sweetened yoghurts, Yoplait™(National Foods Ltd, Australia)	Australia	2006	19±3	4	Normal, 10	25	357.1	Glucose, 2h	Standard	Capillary, Whole blood	HemoCue	UO[5]

1043 No Fat tropical fruit flavors, artificially sweetened yoghurts, Yoplait™(National Foods Ltd, Australia)	Australia	2006	20±4	4	Normal, 10	25	390.6	Glucose, 2h	Standard	Capillary, Whole blood	HemoCue	UO[5]
1044 No Fat yoghurt, various flavors, artificially sweetened yoghurts, Yoplait™(National Foods Ltd, Australia)	Australia	2006	18±3	4	Normal, 10	25	367.6	Glucose, 2h	Standard	Capillary, Whole blood	HemoCue	UO[5]
1045 Fat-free, Passionfruit yoghurt (Jalna Dairy Foods, Australia)	Australia	2004	27±4	5	Normal, 10	50	359.7	Glucose, 2h	Standard	Capillary, whole blood	HemoCue	UO[5]
Non-fat yoghurts, mean of seven foods			26									
**Yoghurt, natural**												
No Added Sugar Natural yoghurt (Tamar Valley Dairy, 1046 Australia)	Australia	2010	17±2	2	Normal, 10	50	495	Glucose, 2h	Standard	Capillary, plasma	Enzymatic	UO[5]
1047 Yoghurt, low fat, natural	UK	2005	35±10	4	Normal, 9	25	431	Glucose, 2h	Standard	Capillary, whole blood	Glucometer[9]	26
**Yoghurt, probiotic**												
1048 Yoghurt, probiotic, prune	UK	2005	44±10	9	Normal, 10	25	349.7	Glucose, 2h	Standard	Capillary, whole blood	Glucometer[9]	26
1049 Yoghurt, probiotic, raspberry	UK	2005	45±10	9	Normal, 10	25	349.7	Glucose, 2h	Standard	Capillary, whole blood	Glucometer[9]	26
All Yoghurts, mean of 72 studies			33									

Food Number and Item	Country of food production	Year of test[1]	GI[2] ± SEM (Glu = 100)	GL	Subjects (type & number)	Avail carb (Test portion)	Test portion[3] (g)	Reference food & time period	Timepoints	Sample collection	Sample analysis method[4]	Ref.
**Yoghurt drinks**												
1050 Probiotic drink, cranberry	UK	2005	56±9	11	Normal, 10	25	409.8	Glucose, 2h	Standard	Capillary, whole blood	Glucometer[9]	26
1051 Probiotic drink, orange	UK	2005	30±8	6	Normal, 10	25	373.1	Glucose, 2h	Standard	Capillary, whole blood	Glucometer[9]	26
1052 Probiotic drink, original	UK	2005	34±5	7	Normal, 10	25	409.8	Glucose, 2h	Standard	Capillary, whole blood	Glucometer[9]	26
1053 Cultured Multivitamin Milk Fruit Drink, Vitalise (Jalna Dairy Foods, Australia)	Australia	2004	24±5	5	Normal, 10	50	381.7	Glucose, 2h	Standard	Capillary, whole blood	HemoCue	UO[5]

Food Number and Item		Country of food production	Year of test[1]	GI[2] ± SEM (Glu = 100)	GL	Subjects (type & number)	Avail carb (Test portion)	Test portion[3] (g)	Reference food & time period	Timepoints	Sample collection	Sample analysis method[4]	Ref.
1054	Yoghurt drink, Wildberry Yoghurt on the Go (Jalna Dairy Foods, Australia)	Australia	2004	19±4	4	Normal, 10	50	381.7	Glucose, 2h	Standard	Capillary, whole blood	HemoCue	UO[5]
1055	Yoghurt drink, reduced-fat, Vaalia™, tropical passionfruit (Pauls Ltd., Australia)	Australia	1999	38±4	8	Normal, 10	25	173.6	Bread, 2h	Standard	Capillary, plasma	Enzymatic	UO[5]
	**Dairy products with added ingredients**												
1056	Control dairy beverage	Canada	2013*	70±7	7	Normal, 11	50	250	Glucose, 2h	Standard	Venous, plasma/serum	YSI	85
1057	Dairy beverage with soy-soluble polysaccharides (2.5 g fiber)	Canada	2013*	65±11	7	Normal, 11	50	250	Glucose, 2h	Standard	Venous, plasma/serum	YSI	85
1058	Dairy beverage with flaxseed gum (2.5 g fiber)	Canada	2013*	54±9	5	Normal, 11	50	250	Glucose, 2h	Standard	Venous, plasma/serum	YSI	85
1059	Control dairy pudding (0.25 g fiber)	Canada	2013*	63±6	6	Normal, 11	50	250	Glucose, 2h	Standard	Venous, plasma/serum	YSI	85
1060	Dairy pudding with soy-soluble polysaccharides (2.5 g fiber)	Canada	2013*	61±6	6	Normal, 11	50	250	Glucose, 2h	Standard	Venous, plasma/serum	YSI	85
1061	Dairy pudding with flaxseed gum (2.5 g fiber)	Canada	2013*	57±8	6	Normal, 11	50	250	Glucose, 2h	Standard	Venous, plasma/serum	YSI	85
	**Soy-based dairy product alternatives**												
	**Soy milks**												
1062	Soy milk beverage, Original flavored, President's Choice® Blue Menu™ (Loblaw Brands Limited, Canada)	Canada	20042007	15±4	2	Normal, 10	50	NS	Bread, 2h	Standard	Capillary, whole blood	YSI	UO[7]
1063	Soy milk, full-fat, with sucrose (Sanitarium, Australia)	Australia	1997	31±5	3	Normal, 10-12	50	NS	Bread, 2h	Standard	Capillary, plasma	Enzymatic	25
1064	Soy milk, full-fat (3%), Original, 0 mg calcium, with maltodextrin (So Natural Foods, Australia)	Australia	2000	44±5	4	Normal, 10	25	337.8	Glucose, 2h	Standard	Capillary, plasma	Enzymatic	UO[5]
1065	Soy milk, full-fat (3%), Calciforte, 120 mg calcium, with maltodextrin (So Natural Foods, Australia)	Australia	2000	36±4	4	Normal, 10	25	360.0	Glucose, 2h	Standard	Capillary, plasma	Enzymatic	UO[5]
	Soy milk, full-fat (3%), 120 mg calcium, Calciforte (So 1066 Natural Foods, Australia)	Australia	2006	41±4	4	Normal, 15	25	333.3	Glucose, 2h	Standard	Capillary, whole blood	HemoCue	UO[10]

Food Number and Item		Country of food	Year of [1]	GI[2] ± SEM	GL	Subjects (type & number)	Avail carb (Test portion)	Test portion[3] (g)	Reference food & time period	Timepoints	Sample collection	Sample analysis method[4]	Ref.
1067	Soy milk, Vitasoy® Organic (National Foods Ltd, Australia)	Australia	2008	43±3	4	Normal, 10	25	412.5	Glucose, 2h	Standard	Capillary, plasma	Enzymatic	UO[5]
1068	Soy milk, Vitasoy® So Milky Regular (National Foods Ltd, Australia)	Australia	2008	21±5	2	Normal, 10	25	800.8	Glucose, 2h	Standard	Capillary, plasma	Enzymatic	UO[5]
	Soy milk full-fat, mean of five studies			37									
1069	Soy milk, low-fat (0.7%), Vitasoy® Light Original (National Foods Ltd, Australia)	Australia	2005	45±5	5	Normal, 10	25	641.0	Glucose, 2h	Standard	Capillary, whole blood	HemoCue	UO[5]
	Soy milk, reduced-fat (1.5%), Vitasoy® So Milky Lite 1070 (National Foods Ltd, Australia)	Australia	2008	17±3	2	Normal, 10	25	851.7	Glucose, 2h	Standard	Capillary, plasma	Enzymatic	UO[5]
1071	Soy milk, reduced-fat (1.5%), 120 mg calcium, Light, containing maltodextrin (So Natural Foods, Australia)	Australia	2000	44±3	4	Normal, 10	25	463.0	Glucose, 2h	Standard	Capillary, plasma	Enzymatic	UO[5]
	Soy milk, reduced-fat (1.8%), 120 mg calcium, Vitasoy® 1072 Premium Vitality+ (National Foods Ltd, Australia)	Australia	2005	18±3	2	Normal, 10	25	520.8	Glucose, 2h	Standard	Capillary, whole blood	HemoCue	UO[5]
	Soy milk reduced-fat, mean of 4 studies			31									
	**Soy milk drinks, flavored**												
1073	Soy Beverage, Chocolate flavored, President's Choice® Blue Menu™(Loblaw Brands Limited, Canada)	Canada	20042007	40±5	8	Normal, 10	50	NS	Bread, 2h	Standard	Capillary, whole blood	YSI	UO[7]
1074	Soy Beverage, Vanilla flavored, President's Choice® Blue Menu™ (Loblaw Brands Limited, Canada)	Canada	2004-2007	28±3	6	Normal, 10	50	NS	Bread, 2h	Standard	Capillary, whole blood	YSI	UO[7]
	Soy milk, reduced-fat (1.5%), Vitasoy® Lush, Chocolate 1075 (National Foods Ltd, Australia)	Australia	2008	31±3	6	Normal, 10	25	353.4	Glucose, 2h	Standard	Capillary, plasma	Enzymatic	UO[5]
	Soy milk, reduced-fat (1.5%), Vitasoy® Lush, Vanilla 1076 (National Foods Ltd, Australia)	Australia	2008	31±3	6	Normal, 10	25	376.8	Glucose, 2h	Standard	Capillary, plasma	Enzymatic	UO[5]
	**Soy yoghurt**												
1077	Soy yoghurt, peach and mango, 2% fat, sugar (So Natural Foods, Australia)	Australia	2000	50±3	10	Normal, 10	25	268.8	Glucose, 2h	Standard	Capillary, plasma	Enzymatic	UO[5]

		production	test	(Glu = 100)									
	**FRUIT AND FRUIT PRODUCTS**												
	Average available carbohydrate portion = 15 g, this value was used to determine the nominal GL for each item in this category.												
	**Apples**												
1078	Apples, raw	Australia	2020	44±5	7	Normal, 10	50	431.0	Glucose, 2h	Standard	Capillary, plasma	Enzymatic	UO[5]
1079	Apples, canned in juice	Australia	2007	42±5	6	Normal, 10	50	337.8	Glucose, 2h	Standard	Capillary, whole blood	HemoCue	UO[5]
1080	Sliced Apples, canned, solid packed without juice (Langeberg and Ashton Foods Pty Ltd, South Africa)	South Africa	2006	42±2	6	Normal, 10	50	602.4	Glucose, 2h	Standard	Capillary, plasma	Enzymatic	UO[5]
	**Apples, dried**												
1081	Apple, dried	Australia	1998	29±5	4	Normal, 10	50	82.3	Bread, 2h	Standard	Capillary, plasma	Enzymatic	UO[5]
1082	Apples, dried (*Malus pumila* Mill.)	China	2018*	43±4	6	Normal, 11	50	76.8	Glucose, 2h	Standard	Capillary, whole blood	Glucometer[9]	86
	**Apricots**												
1083	Apricots, raw, NS	Australia	2007	34±3	5	Normal, 10	50	324.7	Glucose, 2h	Standard	Capillary, plasma	Enzymatic	UO[5]
1084	Apricots, raw, NS	Canada	2018*	42±5	6	Normal, 10	50	81.4	Bread, 2h	Standard	Capillary, whole blood	YSI	87
1085	Apricot Halves canned in fruit juice (Langeberg and Ashton Foods Pty Ltd, South Africa)	South Africa	2006	51±5	8	Normal, 10	50	495.0	Glucose, 2h	Standard	Capillary, plasma	Enzymatic	UO[5]
	**Apricots, dried**												
1086	Apricots, dried, ready to eat, bite size	UK	2005	32±7	5	Normal, 10	50	137.0	Glucose, 2h	Standard	Capillary, whole blood	Glucometer[9]	26
1087	Apricots, dried (*Armeniaca vulgaris* Lam.)	China	2018*	56±4	8	Normal, 11	50	90.4	Glucose, 2h	Standard	Capillary, whole blood	Glucometer[9]	86
	**Banana, raw**												
1088	Banana, raw	Australia	2008	47±5	7	Normal, 10	50	250	Glucose, 2h	Standard	Capillary, plasma	Enzymatic	UO[5]
1089	Banana, lady finger (Moraitis, Australia)	Australia	2014	49±5	7	Normal, 12	50	259.2	Glucose, 2h	Standard	Capillary, plasma	Enzymatic	UO[5]

Food Number and Item		Country of food production	Year of test[1]	GI[2] ± SEM (Glu = 100)	GL	Subjects (type & number)	Avail carb (Test portion)	Test portion[3] (g)	Reference food & time period	Timepoints	Sample collection	Sample analysis method[4]	Ref.
1090	Banana, lady finger (Moraitis, Australia)	Australia	2014	53±4	8	Normal, 12	50	268.3	Glucose, 2h	Standard	Capillary, plasma	Enzymatic	UO[5]
1091	Banana, raw, overripe	Australia	2008	57±8	9	Normal, 9	50	250	Glucose, 2h	Standard	Capillary, plasma	Enzymatic	UO[5]
	**Blueberries**												
1092	Blueberries, wild (Wild Blueberries Association, Canada)	Canada	20042007	53±7	8	Normal, 10	50	568.2	Bread, 2h	Standard	Capillary, whole blood	YSI	UO[5]
	**Breadfruit (*Artocarpus altilis*)**												
1093	Breadfruit, peeled, boiled 10 min	Jamaica	2005	47±5	7	Normal, 10	50	276.6	Glucose, 2h	Standard	Capillary, plasma	Enzymatic	88
1094	Breadfruit, roasted on preheated charcoal	Jamaica	2005	72±8	11	Normal, 10	50	221.3	Glucose, 2h	Standard	Capillary, plasma	Enzymatic	88
1095	Jackfruit365 Green Jackfruit, prepared as a porridge with water	India	2016	65±5	10	Normal, 10	25	59.2	Glucose, 2h	Standard	Capillary, plasma	Enzymatic	UO[5]
	**Cranberries**												
1096	Cranberries, sweetened, dried (Ocean Spray, USA)	USA	2011	62±6	9	Normal, 10	50	65.1	Glucose, 2h	Standard	Capillary, plasma	Enzymatic	UO[5]
	**Cherries**												
1097	Cherries, sour, pitted, canned (Always Fresh brand, Australia)	Australia	2007	41±5	6	Normal, 10	25	142	Glucose, 2h	Standard	Capillary, plasma	Enzymatic	UO[5]
	**Custard apple**												
1098	Custard apple, raw, flesh only	Australia	2000	54±2	8	Normal, 12	50	316.5	Glucose, 2h	Standard	Capillary, plasma	Enzymatic	74
	**Dates**												
1099	Dates, Bo ma'an variety (Al Saad date factory, Al Ain, UAE)	UAE	2011*	46±7	7	Normal, 13	50	72.7	Glucose, 2h	Standard	Capillary, whole blood	Glucometer[9]	89
1100	Dates, Dabbas variety (Al Saad date factory, Al Ain, UAE)	UAE	2011*	49±7	7	Normal, 13	50	76.2	Glucose, 2h	Standard	Capillary, whole blood	Glucometer[9]	89
1101	Dates, Deglet noir variety	Algeria	2016*	52±3	8	Normal, 10	50	NS	Glucose, 2h	Standard	Capillary, whole blood	Glucometer[9]	90

Food Number and Item		Country of food production	Year of test[1]	GI[2] ± SEM (Glu = 100)	GL	Subjects (type & number)	Avail carb (Test portion)	Test portion[3] (g)	Reference food & time period	Timepoints	Sample collection	Sample analysis method[4]	Ref.
1102	Dates, Fara'd variety (Al Saad date factory, Al Ain, UAE)	UAE	2011*	54±6	8	Normal, 13	50	72.5	Glucose, 2h	Standard	Capillary, whole blood	Glucometer[9]	89
1103	Dates, H'mira variety	Algeria	2016*	48±2	7	Normal, 10	50	NS	Glucose, 2h	Standard	Capillary, whole blood	Glucometer[9]	90
1104	Dates, Khalas (Rutab, soft early ripened) variety	UAE	2002	47±5	7	Normal, 11	50	101.8	Glucose, 2h	Standard	Capillary, whole blood	Glucometer[9]	91
1105	Dates, Khalas variety (Al Saad date factory, Al Ain, UAE)	UAE	2011*	55±8	8	Normal, 13	50	72.6	Glucose, 2h	Standard	Capillary, whole blood	Glucometer[9]	89
1106	Dates, Khalas (*Phoenix dactylifera* L.)	Saudi Arabia	2013*	55±6	8	Normal, 10	50	NS	Glucose, 2h	Standard	Capillary, plasma	YSI	92
1107	Dates, Lulu variety (Al Saad date factory, Al Ain, UAE)	UAE	2011*	54±9	8	Normal, 13	50	73.6	Glucose, 2h	Standard	Capillary, whole blood	Glucometer[9]	89
1108	Dates, NS	Canada	2018*	68±6	10	Normal, 10	50	77.8	Bread, 2h	Standard	Capillary, whole blood	YSI	87
1109	Dates, Tinissine variety	Algeria	2016*	44±2	7	Normal, 10	50	NS	Glucose, 2h	Standard	Capillary, whole blood	Glucometer[9]	90
	Dates, mean of 11 foods			52									
	**Dates, dried**												
1110	Dates, Ajwah cultivar, Tamer stage	Saudi Arabia	2016*	60±6	9	Normal, 10	50	70.1	Glucose, 2h	Standard	Capillary, whole blood	Glucometer[9]	93
1111	Dates, Khalas (Tamer, sun dried stored in traditional Bedu manner) variety	UAE	2002	45±8	7	Normal, 11	50	66.8	Glucose, 2h	Standard	Capillary, whole blood	Glucometer[9]	91
1112	Dates, Khalas variety, dried and vacuum packed	UAE	2002	36±3	5	Normal, 11	50	66.7	Glucose, 2h	Standard	Capillary, whole blood	Glucometer[9]	91
1113	Dates, Khudri cultivar, Tamer stage	Saudi Arabia	2016*	62±7	9	Normal, 10	50	67.1	Glucose, 2h	Standard	Capillary, whole blood	Glucometer[9]	93
1114	Dates, Maktoomi cultivar, Tamer stage	Saudi Arabia	2016*	71±11	11	Normal, 10	50	68.5	Glucose, 2h	Standard	Capillary, whole blood	Glucometer[9]	93
1115	Dates, Medjool cultivar, Tamer stage	Saudi Arabia	2016*	55±7	8	Normal, 10	50	70.5	Glucose, 2h	Standard	Capillary, whole blood	Glucometer[9]	93

Food Number and Item		Country of food production	Year of test[1]	GI[2] ± SEM (Glu = 100)	GL	Subjects (type & number)	Avail carb (Test portion)	Test portion[3] (g)	Reference food & time period	Timepoints	Sample collection	Sample analysis method[4]	Ref.
1116	Dates, Nabtat-ali cultivar, Tamer stage	Saudi Arabia	2016*	60±7	9	Normal, 10	50	69.3	Glucose, 2h	Standard	Capillary, whole blood	Glucometer[9]	93
1117	Dates, Nabtat-seyf cultivar, Tamer stage	Saudi Arabia	2016*	54±10	8	Normal, 9	50	67.0	Glucose, 2h	Standard	Capillary, whole blood	Glucometer[9]	93
1118	Dates, Osilah cultivar, Tamer stage	Saudi Arabia	2016*	57±12	9	Normal, 9	50	82.5	Glucose, 2h	Standard	Capillary, whole blood	Glucometer[9]	93
1119	Dates, Rashodia cultivar, Tamer stage	Saudi Arabia	2016*	51±7	8	Normal, 10	50	67.3	Glucose, 2h	Standard	Capillary, whole blood	Glucometer[9]	93
1120	Dates, Ruthana cultivar, Tamer stage	Saudi Arabia	2016*	53±4	8	Normal, 10	50	73.4	Glucose, 2h	Standard	Capillary, whole blood	Glucometer[9]	93
1121	Dates, Sag'ai cultivar, Tamer stage	Saudi Arabia	2016*	45±6	7	Normal, 9	50	72.7	Glucose, 2h	Standard	Capillary, whole blood	Glucometer[9]	93
1122	Dates, Sayer, dried, Trident™ brand	Australia	2014	56±3	8	Normal, 10	50	63.7	Glucose, 2h	Standard	Capillary, plasma	Enzymatic	UO[5]
1123	Dates, Sellaj cultivar, Tamer stage	Saudi Arabia	2016*	75±10	11	Normal, 9	50	69.0	Glucose, 2h	Standard	Capillary, whole blood	Glucometer[9]	93
1124	Dates, Shaqra cultivar, Tamer stage	Saudi Arabia	2016*	43±6	6	Normal, 10	50	66.9	Glucose, 2h	Standard	Capillary, whole blood	Glucometer[9]	93
1125	Dates, Shishi cultivar, Tamer stage	Saudi Arabia	2016*	50±7	8	Normal, 10	50	71.8	Glucose, 2h	Standard	Capillary, whole blood	Glucometer[9]	93
1126	Dates, Sukkary cultivar, Tamer stage	Saudi Arabia	2016*	43±5	6	Normal, 10	50	77.6	Glucose, 2h	Standard	Capillary, whole blood	Glucometer[9]	93
1127	Dates, Um-Kabar cultivar, Tamer stage	Saudi Arabia	2016*	59±7	9	Normal, 10	50	69.1	Glucose, 2h	Standard	Capillary, whole blood	Glucometer[9]	93
1128	Dates, Wannanah cultivar, Tamer stage	Saudi Arabia	2016*	51±7	8	Normal, 10	50	67.5	Glucose, 2h	Standard	Capillary, whole blood	Glucometer[9]	93
	Dates, dried, mean of 19 foods			54									

Food Number and Item		Country of food production	Year of test[1]	GI[2] ± SEM (Glu = 100)	GL	Subjects (type & number)	Avail carb (Test portion)	Test portion[3] (g)	Reference food & time period	Timepoints	Sample collection	Sample analysis method[4]	Ref.
	**Figs, dried**												
1129	Figs, dried, no added, sugar, organic, Turkish (Woolworths Macro brand, Australia)	Turkey	2018	54±6	8	Normal, 10	50	91.7	Glucose, 2h	Standard	Capillary, plasma	Enzymatic	UO[5]
1130	Figs, dried, tenderized, Dessert Maid brand (Ernest Hall & Sons, Sydney, Australia)	Australia	2002	61±6	9	Normal, 10	50	115.4	Glucose, 2h	Standard	Capillary, plasma	Enzymatic	UO[5]
	**Guava**												
1131	Guava, raw, cut into bite size pieces	Singapore	2017*	29±4	4	Normal, 19	25	280	Glucose, 2h	Standard	Capillary, whole blood	HemoCue	94
1132	Guava, raw, puree	Singapore	2017*	47±4	7	Normal, 19	25	280	Glucose, 2h	Standard	Capillary, whole blood	HemoCue	94
	**Grapes**												
1133	Grapes, Crimson seedless	Australia	2008	50±3	8	Normal, 10	50	279.3	Glucose, 2h	Standard	Capillary, plasma	Enzymatic	UO[5]
1134	Grapes, green, Menidee, seedless	Australia	2008	54±4	8	Normal, 10	50	333.3	Glucose, 2h	Standard	Capillary, plasma	Enzymatic	UO[5]
1135	Grapes, black, Waltham Cross	Australia	2001	59±5	9	Normal, 11	50	330.7	Bread, 2h	Standard	Capillary, plasma	Enzymatic	UO[5]
	**Grapefruit**												
1136	Grapefruit, ruby red segments, canned in juice (Woolworths Select, Australia)	Australia	2008	47±5	7	Normal, 10	50	294.1	Glucose, 2h	Standard	Capillary, plasma	Enzymatic	UO[5]
	**Jujubes**												
1137	Jujubes, dried (*Ziziphus jujubu* Mill.)	China	2018*	55±6	8	Normal, 11	50	84	Glucose, 2h	Standard	Capillary, whole blood	Glucometer[9]	86
	**Lychee**												
1138	Lychee, fresh	Australia	2009	57±3	9	Normal, 9	50	308.6	Glucose, 2h	Standard	Capillary, plasma	Enzymatic	UO[5]
1139	Lychee, canned in syrup and drained, Narcissus brand	China	2000	79±8	12	Normal, 12	50	292.4	Glucose, 2h	Standard	Capillary, plasma	Enzymatic	74

	**Mandarins**												
1140	Mandarin orange (*Citrus reticulata*)[19]	Italy	2004	52±8	8	Normal, ≥9	25	142	Glucose, 2h	Standard	Capillary, whole blood	YSI	UO[14]
1141	Mandarin segments, canned in juice (Woolworths Select, Australia)	Australia	2008	47±2	7	Normal, 10	50	500	Glucose, 2h	Standard	Capillary, plasma	Enzymatic	UO[5]
	**Mango**												
1142	Mango, raw (Chin-Hwang Mango)	Taiwan	2010*	48±0.3	7	Normal, 10	50	NS	Bread, 2h	Standard	Capillary, plasma	Enzymatic	19
1143	Mango, low-fat frozen fruit dessert, Frutia™ (Weis Frozen Foods, Toowong, Australia)	Australia	2000	42±3	6	Normal, 10	50	217.4	Glucose, 2h	Standard	Capillary, plasma	Enzymatic	UO[5]
	**Melon**												
1144	Melon (*Cucumis melon*)[19]	Italy	2004	60±9	9	Normal, ≥9	25	338	Glucose, 2h	Standard	Capillary, whole blood	YSI	UO[14]
1145	Watermelon, raw (*Citrullus vulgaris-red* variety)	Malaysia	2008*	55±3	8	Normal, 10	50	893	Glucose, 2h	Standard	Capillary, plasma	Enzymatic	95
1146	Watermelon (*Citrullus lanatus*), raw, red-fleshed, seeded	Malaysia	2017*	48±1	7	Normal, 14	25	236	Glucose, 2h	Standard	Capillary, plasma	YSI	96
1147	Watermelon (*Citrullus lanatus*), raw, red-fleshed, seedless	Malaysia	2017*	51±2	8	Normal, 14	25	239	Glucose, 2h	Standard	Capillary, plasma	YSI	96
1148	Watermelon (*Citrullus lanatus*), raw, yellow-fleshed, seedless	Malaysia	2017*	47±2	7	Normal, 14	25	233	Glucose, 2h	Standard	Capillary, plasma	YSI	96
	Watermelon, mean of four foods			50									
	**Nectarines**												
1149	Nectarines, raw	Australia	2006	43±6	6	Normal, 10	25	324.7	Glucose, 2h	Standard	Capillary, plasma	Enzymatic	UO[5]

Food Number and Item		Country of food production	Year of test[1]	GI[2] ± SEM (Glu = 100)	GL	Subjects (type & number)	Avail carb (Test portion)	Test portion[3] (g)	Reference food & time period	Timepoints	Sample collection	Sample analysis method[4]	Ref.
	**Orange**												
1150	Oranges, navel	Australia	2020	45±5	7	Normal, 10	50	406.5	Glucose, 2h	Standard	Capillary, plasma	Enzymatic	UO[5]
1151	Orange & Grapefruit segments, canned in juice (Woolworths Select, Australia)	Australia	2008	53±4	8	Normal, 10	50	312.5	Glucose, 2h	Standard	Capillary, plasma	Enzymatic	UO[5]

Food Number and Item	Country of food production	Year of test[1]	GI[2] ± SEM (Glu = 100)	GL	Subjects (type & number)	Avail carb (Test portion)	Test portion[3] (g)	Reference food & time period	Timepoints	Sample collection	Sample analysis method[4]	Ref.
**Passionfruit**												
Passionfruit pulp, canned, 55% passionfruit pulp with added 1152 sugar	Australia	2016	46±5	7	Normal, 10	25	95.8	Glucose, 2h	Standard	Capillary, plasma	Enzymatic	UO[5]
**Paw paw/papaya, raw**												
1153 Papaya/paw paw, raw, cut into bite size pieces	Singapore	2017*	38±2	6	Normal, 19	25	274	Glucose, 2h	Standard	Capillary, whole blood	HemoCue	94
1154 Papaya/paw paw, raw, puree	Singapore	2017*	42±5	6	Normal, 19	25	274	Glucose, 2h	Standard	Capillary, whole blood	HemoCue	94
**Peaches, raw or canned**												
1155 Peach (*Prunus persica*)[19]	Italy	2004	76±9	11	Normal, ≥9	25	410	Glucose, 2h	Standard	Capillary, whole blood	YSI	UO[14]
1156 Peach, canned in natural juice (SPC Ardmona Ltd, Shepparton, VIC, Australia)	Australia	2007	46±4	7	Normal, 9	50	438.6	Glucose, 2h	Standard	Capillary, plasma	Enzymatic	UO[5]
1157 Peach, canned in heavy syrup (Homebrand, Yennora, NSW, Australia)	Australia	2007	64±3	10	Normal, 9	50	314.5	Glucose, 2h	Standard	Capillary, plasma	Enzymatic	UO[5]
1158 Peach & Grapes, canned in natural fruit juice (Woolworths Select, Australia)	Australia	2008	46±5	7	Normal, 10	50	490.2	Glucose, 2h	Standard	Capillary, plasma	Enzymatic	UO[5]
1159 Peach & Pineapple, canned in natural fruit juice (Woolworths Select, Australia)	Australia	2008	45±4	7	Normal, 10	50	454.5	Glucose, 2h	Standard	Capillary, plasma	Enzymatic	UO[5]
**Pears**												
1160 Pear, Williams, raw, under ripe/crunchy	Australia	2015	24±3	4	Normal, 10	25	260.4	Glucose, 2h	Standard	Capillary, plasma	Enzymatic	UO[5]
1161 Pear, Williams, raw, ripe/soft	Australia	2015	33±4	5	Normal, 10	25	260.4	Glucose, 2h	Standard	Capillary, plasma	Enzymatic	UO[5]
1162 Pear slices, canned in natural juice (SPC Ltd, Australia)	Australia	2015	31±2	5	Normal, 10	50	181.3	Glucose, 2h	Standard	Capillary, plasma	Enzymatic	UO[5]
**Persimmon**												
1163 Persimmon (*Diospyros kaki*)[19]	Italy	2004	61±8	9	Normal, ≥9	25	156	Glucose, 2h	Standard	Capillary, whole blood	YSI	UO[14]

	**Pineapple**												
1164	Pineapple, raw (*Ananas comosa*)	Malaysia	2008*	82±4	12	Normal, 9	50	543	Glucose, 2h	Standard	Capillary, plasma	Enzymatic	95
1165	Pineapple pieces, canned in fruit juice (Woolworths Select, Australia)	Australia	2008	43±4	6	Normal, 10	50	312.5	Glucose, 2h	Standard	Capillary, plasma	Enzymatic	UO[5]
1166	Pineapple pieces, canned in natural fruit juice (Woolworths Select, Australia)	Australia	2007	55±5	8	Normal, 10	50	384.6	Glucose, 2h	Standard	Capillary, plasma	Enzymatic	UO[5]
	Pineapple & Papaya pieces, canned in natural juice 1167 (Woolworths Select, Australia)	Australia	2008	53±7	8	Normal, 10	50	424.5	Glucose, 2h	Standard	Capillary, plasma	Enzymatic	UO[5]
	**Prunes**												
1168	Prunes, pitted (Sunsweet Growers Inc., Yuba City, USA)	USA	2000	29±4	4	Normal, 10	50	68.2	Glucose, 2h	Standard	Capillary, plasma	Enzymatic	UO[5]
1169	Prunes, pitted, Californian (Sunsweet Growers Inc., Yuba City, USA)	USA	2009	40±6	6	Normal, 10	50	90.9	Glucose, 2h	Standard	Capillary, plasma	Enzymatic	UO[5]
	**Raisins**												
1170	Raisins (Sun Maid, USA)	USA	2013	49±4	7	Normal, 10	50	69.0	Bread, 2h	Standard	Capillary, whole blood	YSI	97
1171	Raisins	Canada	2018*	55±5	8	Normal, 10	50	64.8	Bread, 2h	Standard	Capillary, whole blood	YSI	87
1172	Raisins (*Vitis vinifera* Linn.)	China	2018*	56±5	8	Normal, 11	50	75.2	Glucose, 2h	Standard	Capillary, whole blood	Glucometer[9]	86
1173	Raisins	USA	2009	61±4	9	Normal, 10	50	79.9	Glucose, 2h	Standard	Capillary, plasma	Enzymatic	UO[5]
	Raisins, mean of four studies			55									
	**Strawberries**												
1174	Strawberries, fresh, raw	Australia	2001	40±7	6	Normal, 10	25	925	Glucose, 2h	Standard	Capillary, plasma	Enzymatic	UO[5]
	**Sultanas**												
1175	Sultanas (Value, UK)	UK	2005	56±6	8	Normal, 10	50	72.0	Glucose, 2h	Standard	Capillary, whole blood	Glucometer[9]	26
1176	Sultanas	Canada	2018*	51±4	8	Normal, 10	50	67	Bread, 2h	Standard	Capillary, whole blood	YSI	87

Food Number and Item		Country of food production	Year of test[1]	GI[2] ± SEM (Glu = 100)	GL	Subjects (type & number)	Avail carb (Test portion)	Test portion[3] (g)	Reference food & time period	Timepoints	Sample collection	Sample analysis method[4]	Ref.
	**Other fruit products**												
1177	Fruit Salad canned in fruit juice, containing peach, pear, apricot, pineapple and cherries (Langeberg and Ashton Foods Pty Ltd, South Africa)	South Africa	2007	54±3	8	Normal, 10	50	526.3	Glucose, 2h	Standard	Capillary, plasma	Enzymatic	UO[5]
1178	Mixed fruit, dried (Value, UK)	UK	2005	60±7	9	Normal, 10	50	73.6	Glucose, 2h	Standard	Capillary, whole blood	Glucometer[9]	26
1179	Frozen fruit dessert, Vitari, wild berry, non-dairy (Nestlé, Australia)	Australia	2000	59±8	9	Normal, 10	50	238.1	Bread, 2h	Standard	Capillary, plasma	Enzymatic	UO[5]
	**Fruit and vegetable juices**												
	Average available carbohydrate portion = 20 g, this value was used to determine the nominal GL for each item in this category, except for tomato juice and carrot juice where 10 g was used.												
	**Apple juice**												
1180	Apple juice, unsweetened, reconstituted (Berrivale Orchards Ltd, Berri, Australia)	Australia	1998	39±5	8	Normal, 10	50	NS	Bread, 2h	Standard	Capillary, plasma	Enzymatic	UO[5]
1181	Apple juice, Granny Smith, unsweetened	Australia	2003	44±3	9	Normal, 10	50	442.5	Glucose, 2h	Standard	Capillary, plasma	Enzymatic	UO[5]
1182	Apple juice, green apple (100%) (Pfanner, Italy)	Italy	2011	67±7	13	Normal, 9	50	446	Glucose, 2h	Standard	Capillary, whole blood	YSI	3
1183	Apple juice, pure, clear, unsweetened (The Wild About Fruit Company, Wandin, VIC, Australia)	Australia	2001	44±2	9	Normal, 10	50	438.6	Glucose, 2h	Standard	Capillary, plasma	Enzymatic	UO[5]
1184	Apple juice, pure, cloudy, unsweetened (The Wild About Fruit Company, Australia)	Australia	2001	37±3	7	Normal, 10	50	476.2	Glucose, 2h	Standard	Capillary, plasma	Enzymatic	UO[5]
	Apple juice, mean of five foods			46									
1185	Apple and blackcurrant juice, no added sugar (Berri Ltd, Australia)	Australia	2003	45±4	9	Normal, 10	50	500.0	Glucose, 2h	Standard	Capillary, whole blood	HemoCue	UO[5]
1186	Apple and cherry juice, pure, unsweetened (The Wild About Fruit Company, Australia)	Australia	2001	43±3	9	Normal, 10	50	378.8	Glucose, 2h	Standard	Capillary, plasma	Enzymatic	UO[5]
1187	Apple and mandarin juice, pure, unsweetened (The Wild About Fruit Company, Australia)	Australia	2002	47±5	9	Normal, 10	50	454.5	Glucose, 2h	Standard	Capillary, plasma	Enzymatic	UO[5]
1188	Apple and mango juice, pure, unsweetened (The Wild About Fruit Company, Australia)	Australia	2004	47±5	9	Normal, 10	50	384.6	Glucose, 2h	Standard	Capillary, plasma	Enzymatic	UO[5]

1189	Apple, pineapple and passionfruit juice, unsweetened (The Wild About Fruit Company, Australia)	Australia	2004	48±5	10	Normal, 10	50	390.6	Glucose, 2h	Standard	Capillary, plasma	Enzymatic	UO[5]
	**Carrot juice**												
1190	Carrot juice, freshly made	Australia	2002	43±3	4	Normal, 9	25	342.5	Glucose, 2h	Standard	Capillary, plasma	Enzymatic	UO[5]

Food Number and Item		Country of food production	Year of test[1]	GI[2] ± SEM (Glu = 100)	GL	Subjects (type & number)	Avail carb (Test portion)	Test portion[3] (g)	Reference food & time period	Timepoints	Sample collection	Sample analysis method[4]	Ref.
	**Cranberry juice**												
1191	Cranberry juice cocktail (Ocean Spray, Melbourne, Australia)	Australia	2000	52±3	10	Normal, 10	50	403	Glucose, 2h	Standard	Capillary, plasma	Enzymatic	UO[5]
	Cranberry juice cocktail (Ocean Spray Inc., Lakeville1192 Middleboro, MA, USA)	USA	2000	68±3	14	Normal, 10	50	357	Glucose, 2h	Standard	Capillary, plasma	Enzymatic	UO[5]
1193	Cranberry juice drink, Ocean Spray® (Gerber Ltd., Bridgewater, Somerset, UK)	UK	2000	56±4	11	Normal, 10	50	431	Glucose, 2h	Standard	Capillary, plasma	Enzymatic	UO[5]
	Cranberry juice, mean of three studies			59									
	**Grape juice**												
1194	Grape juice, 100%, Daily C (Weichuan Shanghai, China)	China	2019	63±5	13	Normal, 10	50	365.9	Glucose, 2h	Standard	Capillary, plasma	Enzymatic	UO[18]
1195	Grape Nectar (Chateau Barossa, Lyndoch, SA, Australia)	Australia	2003	52±5	10	Normal, 9	50	63.9	Glucose, 2h	Standard	Capillary, plasma	Enzymatic	UO[5]
	**Orange juice**												
1196	Orange juice	New Zealand	2019	41±3	8	Normal, 10	50	563.6	Glucose, 2h	Standard	Capillary, plasma	Enzymatic	UO[5]
1197	Orange juice beverage, President's Choice® Blue Menu™ Oh-Mega j (Loblaw Brands Limited, Canada)	Canada	2004-2007	48±4	10	Normal, 10	50	NS	Bread, 2h	Standard	Capillary, whole blood	YSI	UO[7]
1198	Orange juice, unsweetened, reconstituted concentrate, Mr Juicy brand (Berri Ltd, Australia)	Australia	2007	54±5	11	Normal, 10	50	588.2	Glucose, 2h	Standard	Capillary, plasma	Enzymatic	UO[5]
	Orange juice, mean of three foods			48									
	**Pomegranate juice**												
1199	POM Pomegranate juice (POM Wonderful LLC, USA)	USA	2009	53±3	11	Normal, 9	50	313.2	Glucose, 2h	Standard	Capillary, plasma	Enzymatic	UO[5]

	**Prune juice**												
1200	Prune juice (Golden Circle, Australia)	Australia	2004	43±3	9	Normal, 9	50	362.3	Glucose, 2h	Standard	Capillary, whole blood	HemoCue	UO[5]
	**Tomato juice**												
1201	Tomato juice, low sodium, President's Choice® Blue Menu™ (Loblaw Brands Limited, Canada)	Canada	20042007	23±6	2	Normal, 10	50	NS	Bread, 2h	Standard	Capillary, whole blood	YSI	UO[7]
1202	Tomato juice, canned, no added sugar (Berri Ltd, Australia)	Australia	2002	38±4	4	Normal, 10	25	892.9	Glucose, 2h	Standard	Capillary, whole blood	HemoCue	UO[5]
	**Watermelon juice**												
1203	Watermelon (*Citrullus lanatus*), raw, red-fleshed, seedless, blended using an automatic juicer to form a juice	Malaysia	2017*	51±1	10	Normal, 14	25	236	Glucose, 2h	Standard	Capillary, plasma	YSI	96

Food Number and Item		Country of food production	Year of test[1]	GI[2] ± SEM (Glu = 100)	GL	Subjects (type & number)	Avail carb (Test portion)	Test portion[3] (g)	Reference food & time period	Timepoints	Sample collection	Sample analysis method[4]	Ref.
	**Other juices**												
1204	SuperJuice Greenzone, containing apple, orange and pineapple juice	Australia	2006	47±3	9	Normal, 10	50	492.0	Glucose, 2h	Standard	Capillary, whole blood	HemoCue	UO[5]
1205	SuperJuice Immune, containing apple juice, pineapple juice and guava puree	Australia	2006	47±3	9	Normal, 10	50	500.9	Glucose, 2h	Standard	Capillary, whole blood	HemoCue	UO[5]
1206	SuperJuice Kickstart, containing apple juice, blueberry puree and banana puree	Australia	2006	39±3	8	Normal, 10	50	442.2	Glucose, 2h	Standard	Capillary, whole blood	HemoCue	UO[5]
	**Fruit spreads/Jam/Marmalade**												
	Average available carbohydrate portion = 15 g, this value was use[illegible] th[illegible] nominal [illegible]L for each item in this category.												
1207	Apricot 100% Pure Fruit spread, no added sugar (Freedom Foods, Australia)	Australia	2006-2007	43±6	6	Normal, 10	50	122.0	Glucose, 2h	Standard	Capillary, whole blood	HemoCue	UO[10]
1208	Apricot 100% Fruit Spread, Cottees™ (Cadbury Schweppes, Australia)	Australia	2004	50±6	8	Normal, 9	50	80.6	Glucose, 2h	Standard	Capillary, whole blood	HemoCue	UO[5]
1209	Apricot fruit spread, reduced sugar (Glen Ewin Jams, Para Hills, Australia)	Australia	1997	55±7	8	Normal, 10	50	NS	Bread, 2h	Standard	Capillary, whole blood	HemoCue	UO[5]
1210	Apricot spread, President's Choice® Blue Menu™ Twice the Fruit Apricot spread (Loblaw Brands Limited, Canada)	Canada	2007	56±8	8	Normal, 10	50	125.0	Bread, 2h	Standard	Capillary, whole blood	YSI	UO[7]

Food Number and Item		Country of food production	Year of test[1]	GI[2] ± SEM (Glu = 100)	GL	Subjects (type & number)	Avail carb (Test portion)	Test portion[3] (g)	Reference food & time period	Timepoints	Sample collection	Sample analysis method[4]	Ref.
1211	Fruit spread, with thick apricot (Rhapsodie de Fruit Thick Apricot, St Dalfour, Italy)	Italy	2010	63±6	9	Normal, 10	25	50	Glucose, 2h	Standard	Capillary, whole blood	YSI	3
1212	Apricot jam, organic apricot, with apple juice (Fiordifrutta albicocca, Rigoni di Asiago, Italy)	Italy	2010	63±9	9	Normal, 10	25	65	Glucose, 2h	Standard	Capillary, whole blood	YSI	3
1213	Apricot jam (Confettura extra albicocche, Bonne Maman, Italy)	Italy	2010	69±6	10	Normal, 10	25	43	Glucose, 2h	Standard	Capillary, whole blood	YSI	3
	Apricot spread, mean of seven foods			57									
1214	Berry compote, organic (Fruttosa frutti di bosco, Rigoni di Asiago, Italy)	Italy	2009	44±4	7	Normal, 10	25	100	Glucose, 2h	Standard	Capillary, whole blood	YSI	3
1215	Blackberry 100% Fruit Spread, Cottees™ brand (Cadbury Schweppes, Australia)	Australia	2004	46±5	7	Normal, 9	50	101.6	Glucose, 2h	Standard	Capillary, whole blood	HemoCue	UO[5]
1216	Blueberry fruit spread, wild blueberry (Rhapsodie de Fruit Wild Blueberry, St. Dalfour, Italy)	Italy	2010	74±5	11	Normal, 10	25	48	Glucose, 2h	Standard	Capillary, whole blood	YSI	3
1217	Blueberry jam, wild blueberry (Confettura extra mirtilli selvatici, Bonne Maman, Italy)	Italy	2010	63±3	9	Normal, 10	25	44	Glucose, 2h	Standard	Capillary, whole blood	YSI	3
1218	Blueberry jam, organic blueberry, with apple juice (Fiordifrutta Mirtilli neri, Rigoni di Asiago, Italy)	Italy	2010	51±9	8	Normal, 10	25	64	Glucose, 2h	Standard	Capillary, whole blood	YSI	3
	Date spread, Divine (Buderim Ginger, Buderim, QLD, 1219 Australia)	Australia	2006	29±4	4	Normal, 10	50	79.4	Glucose, 2h	Standard	Capillary, whole blood	HemoCue	UO[5]
1220	Ginger Spread, sucrose-free (Buderim Ginger, Australia)	Australia	2006	10±2	2	Normal, 10	50	83.2	Glucose, 2h	Standard	Capillary, whole blood	HemoCue	UO[5]
1221	Ginger marmalade, Original (Buderim Ginger, Australia)	Australia	2006	50±7	8	Normal, 10	50	67.2	Glucose, 2h	Standard	Capillary, whole blood	HemoCue	UO[5]
1222	Orange fruit spread, with thick cut orange (Rhapsodie de Fruit Orange Spread, Thick Cut St Dalfour, Italy)	Italy	2010	68±6	10	Normal, 10	25	53	Glucose, 2h	Standard	Capillary, whole blood	YSI	3
	Orange jam, organic bitter orange, with apple juice 1223 (Fiordifrutta arance amare, Rigoni di Asiago, Italy)	Italy	2010	51±8	8	Normal, 10	25	52	Glucose, 2h	Standard	Capillary, whole blood	YSI	3
1224	Orange marmalade, bitter orange (Marmellata arance amare, Bonne Maman, Italy)	Italy	2010	55±6	8	Normal, 10	25	42	Glucose, 2h	Standard	Capillary, whole blood	YSI	3

1225 Orange marmalade, orange 100% Pure Fruit spread, no added sugar (Freedom Foods, Australia)	Australia	2006	27±3	4	Normal, 9	50	111.1	Glucose, 2h	Standard	Capillary, whole blood	HemoCue	UO[10]
1226 Orange marmalade	Australia	1997	48±9	7	Normal, 9	50	NS	Bread, 2h	Standard	Capillary, plasma	Enzymatic	UO[5]
1227 Orange marmalade 100% Fruit Spread, Cottees™ (Cadbury Schweppes, Australia)	Australia	2004	55±4	8	Normal, 9	50	73.4	Glucose, 2h	Standard	Capillary, plasma	Enzymatic	UO[5]
Orange marmalade, mean of six foods			51									
1228 Peach/Apricot compote, organic (Fruttosa pesca/albicocca, Rigoni di Asiago, Italy)	Italy	2009	47±4	7	Normal, 10	25	100	Glucose, 2h	Standard	Capillary, whole blood	YSI	3
1229 Raspberry 100% Pure Fruit spread, no added sugar (Freedom Foods, Australia)	Australia	2006	26±4	4	Normal, 10	50	108.7	Glucose, 2h	Standard	Capillary, whole blood	HemoCue	UO[10]
1230 Raspberry 100% Fruit Spread, Cottees™ brand (Cadbury Schweppes, Australia)	Australia	2004	46±5	7	Normal, 9	50	73.6	Glucose, 2h	Standard	Capillary, whole blood	HemoCue	UO[5]
1231 Strawberry jam	Australia	1998	51±10	8	Normal, 9	50	NS	Bread, 2h	Standard	Capillary, plasma	Enzymatic	UO[5]
1232 Strawberry 100% Pure Fruit spread, no added sugar (Freedom Foods, Australia)	Australia	2006	29±4	4	Normal, 10	50	110.4	Glucose, 2h	Standard	Capillary, whole blood	HemoCue	UO[10]
1233 Strawberry100% Fruit Spread, Cottees™ brand (Cadbury Schweppes, Australia)	Australia	2004	46±5	7	Normal, 9	50	82.0	Glucose, 2h	Standard	Capillary, whole blood	HemoCue	UO[5]
1234 Strawberry & Rhubarb Fruit Spread, President's Choice® Blue Menu™ Twice the Fruit Spread (Loblaw Brands Limited, Canada)	Canada	2007	69±12	10	Normal, 10	50	150.0	Bread, 2h	Standard	Capillary, whole blood	YSI	UO[7]

Food Number and Item	Country of food production	Year of test[1]	GI[2] ± SEM (Glu = 100)	GL	Subjects number)(type &	Avail carb (Test portion)	Test portion[3] (g)	Reference food & time period	Timepoints	Sample collection	Sample analysis method[4]	Ref.

INFANT FORMULA AND WEANING FOODS

Average available carbohydrate portion = 10 g, this value was used to determine the nominal GL for each item in this category.

1235	Breast milk, human, unpasteurized	Australia	2011	38±7	4	Normal, 10 (adults)	25	NS	Glucose, 2h	Standard	Capillary, plasma	Enzymatic	98
	**Infant formula**												
1236	Infasoy™, soy-based, milk-free (Wyeth Nutritionals, Baulkham Hills, NSW, Australia)	Australia	2001	55±6	6	Normal, 11 (adults)	25	47.8	Glucose, 2h	Standard	Capillary, plasma	Enzymatic	UO[5]
1237	Karicare from Birth Infant formula, containing 83% lactose, 17% maltodextrins (Nutricia Australia Pty Ltd) (40.8 g powder + 271.7 g water)	Australia	2010	30±3	3	Normal, 10 (adults)	25	312.5	Glucose, 2h	Standard	Capillary, plasma	Enzymatic	98
1238	Karicare DeLact Formula for special medical needs, containing 100% corn syrup solids (Nutricia Australia Pty Ltd) (42.0 g powder + 291.3 g water)	Australia	2010	67±6	7	Normal, 10 (adults)	25	33.3	Glucose, 2h	Standard	Capillary, plasma	Enzymatic	98
1239	Karicare™ gold starter formula with omega plus LCP oils (Nutricia, Auckland, New Zealand)	New Zealand	2001	35±5	4	Normal, 10 (adults)	25	46.3	Glucose, 2h	Standard	Capillary, plasma	Enzymatic	UO[5]
1240	Karicare Soy Formula for special medical needs, containing 51% corn syrup solids, 49% maltodextrins (Nutricia Australia Pty Ltd) (47.0 g powder + 331.8 g water)	Australia	2010	60±5	6	Normal, 10 (adults)	25	378.8	Glucose, 2h	Standard	Capillary, plasma	Enzymatic	98
1241	Nan-1™ infant formula with iron (Nestlé, Australia)	Australia	2001	30±6	3	Normal, 9 (adults)	25	43.4	Glucose, 2h	Standard	Capillary, plasma	Enzymatic	UO[5]
1242	Novalac Colic Formula for special medical needs, containing maltodextrins, lactose (Bayer Australia Limited) (42.9 g powder + 299.6 g water)	Australia	2010	57±7	6	Normal, 10 (adults)	25	342.5	Glucose, 2h	Standard	Capillary, plasma	Enzymatic	98
1243	Novalac Reflux Formula for special medical needs, containing lactose, starch (Bayer Australia Limited) (42.4 g powder + 295.4 g water)	Australia	2010	38±5	4	Normal, 10 (adults)	25	337.8	Glucose, 2h	Standard	Capillary, plasma	Enzymatic	98
1244	Novalac Sweet Dreams Formula for special medical needs, containing maltodextrins, lactose, dextrose, starch (Bayer Australia Limited) (43.5 g powder + 303.7 g water)	Australia	2010	52±6	5	Normal, 10 (adults)	25	347.2	Glucose, 2h	Standard	Capillary, plasma	Enzymatic	98
1245	S-26™ infant formula (Wyeth Nutritionals, Australia)	Australia	2001	36±6	4	Normal, 10 (adults)	25	NS	Glucose, 2h	Standard	Capillary, plasma	Enzymatic	UO[5]
1246	S-26 Gold Newborn Infant formula, containing 100% lactose (Wyeth Australia Pty Ltd) (42.5 g powder + 300 g water)	Australia	2010	19±3	2	Normal, 10 (adults)	25	342.5	Glucose, 2h	Standard	Capillary, plasma	Enzymatic	98
1247	S-26 Gold PremGro Formula for special medical needs, containing 76% lactose, 24% corn syrup solids (Wyeth Australia Pty Ltd) (45.2 g powder + 288.1 g water)	Australia	2010	26±4	3	Normal, 10 (adults)	25	333.3	Glucose, 2h	Standard	Capillary, plasma	Enzymatic	98

Food Number and Item		Country of food production	Year of test[1]	GI[2] ± SEM (Glu = 100)	GL	Subjects (type & number)	Avail carb (Test portion)	Test portion[3] (g)	Reference food & time period	Timepoints	Sample collection	Sample analysis method[4]	Ref.
1248	S-26 Gold Progress Infant formula, containing 100% lactose (Wyeth Australia Pty Ltd) (44.0 g powder + 303.2 g water)	Australia	2010	34±7	3	Normal, 10 (adults)	25	347.2	Glucose, 2h	Standard	Capillary, plasma	Enzymatic	98
	**Follow-on/Toddler formula**												
1249	Karicare Follow On Follow-on formula, containing 86% lactose, 14% maltodextrins (Nutricia Australia Pty Ltd) (39.9 g powder + 261.9 g water)	Australia	2010	37±4	4	Normal, 10 (adults)	25	301.8	Glucose, 2h	Standard	Capillary, plasma	Enzymatic	98
1250	S-26 Gold Progress Follow-on formula, containing 100% lactose (Wyeth Australia Pty Ltd) (44.0 g powder + 303.2 g water)	Australia	2010	23±6	2	Normal, 10 (adults)	25	347.2	Glucose, 2h	Standard	Capillary, plasma	Enzymatic	98
1251	S-26 Gold Toddler Follow-on formula, containing 86% lactose, 14% maltodextrins (Wyeth Australia Pty Ltd) (16.7 g powder + 233.3 g water)	Australia	2010	18±3	2	Normal, 10 (adults)	25	250	Glucose, 2h	Standard	Capillary, plasma	Enzymatic	98
1252	Growing up beverage powder, product 1	Malaysia	2011	66±6	7	Normal, 11 (adults)	25	36	Glucose, 2h	Standard	Capillary, plasma	Enzymatic	99
1253	Growing up beverage powder, product 2	Indonesia	2011	55±5	6	Normal, 11 (adults)	25	41	Glucose, 2h	Standard	Capillary, plasma	Enzymatic	99
1254	Growing up beverage powder, product 3	Malaysia	2011	23±4	2	Normal, 11 (adults)	25	64	Glucose, 2h	Standard	Capillary, plasma	Enzymatic	99
1255	Growing up beverage powder, product 4	Indonesia	2011	51±3	5	Normal, 11 (adults)	25	38	Glucose, 2h	Standard	Capillary, plasma	Enzymatic	99
1256	Growing up beverage powder, product 5	Malaysia	2011	54±5	5	Normal, 11 (adults)	25	42	Glucose, 2h	Standard	Capillary, plasma	Enzymatic	99
1257	Growing up beverage powder, product 6	Indonesia	2011	43±3	4	Normal, 11 (adults)	25	38	Glucose, 2h	Standard	Capillary, plasma	Enzymatic	99
1258	Growing up beverage powder, product 7	Malaysia	2011	68±6	7	Normal, 11 (adults)	25	42	Glucose, 2h	Standard	Capillary, plasma	Enzymatic	99
	**Weaning Foods**												
1259	Apple, apricot and banana cereal, Robinsons First Tastes from 4 months (Nutricia, Wells, UK)	UK	2001	56±8	6	Normal, 11 (adults)	25	144.3	Glucose, 2h	Standard	Capillary, plasma	Enzymatic	UO[5]

Food Number and Item	Country of food production	Year of test[1]	GI[2] ± SEM (Glu = 100)	GL	Subjects (type & number)	Avail carb (Test portion)	Test portion[3] (g)	Reference food & time period	Timepoints	Sample collection	Sample analysis method[4]	Ref.
1260 Apple baby food	Czech Republic	2005	46±5	5	Normal, 20 (adults)	50	277.8	Glucose, 2h	0,15,30,45,60,75,90,105, 120	Capillary, whole blood	Glucometer[9]	84
1261 Apple, pear and cinnamon fruit puree, homemade, puree consistency	Australia	2010	29±4	3	Normal, 10 (adults)	25	238	Glucose, 2h	Standard	Capillary, plasma	Enzymatic	UO[5]
1262 Apple, Pear and Cinnamon fruit puree, Rafferty's Garden, for ages 4 – 6 months, commercial	Australia	2010	44±4	4	Normal, 10 (adults)	25	325	Glucose, 2h	Standard	Capillary, plasma	Enzymatic	UO[5]
Chicken and noodles with vegetables, strained, Heinz for 1263 Baby from 4 months (Heinz Wattie's Ltd, Australia)	Australia	2001	67±11	7	Normal, 10 (adults)	25	416.7	Glucose, 2h	Standard	Capillary, plasma	Enzymatic	UO[5]
1264 Creamed rice porridge Robinsons First Tastes from 4 months (Nutricia, Wells, UK)	UK	2001	59±8	6	Normal, 11 (adults)	25	203.7	Glucose, 2h	Standard	Capillary, plasma	Enzymatic	UO[5]
1265 Fruit Muesli, containing wheat germ, rolled oats, apple juice, pear, and orange juice, homemade, porridge with lumps consistency	Australia	2010	38±3	4	Normal, 10 (adults)	25	122	Glucose, 2h	Standard	Capillary, plasma	Enzymatic	UO[5]
1266 Fruity Muesli, Farex™, ages 7 – 9 months, prepared with 272 mL water	Australia	2010	65±7	7	Normal, 10 (adults)	25	36	Glucose, 2h	Standard	Capillary, plasma	Enzymatic	UO[5]
1267 Lentils and vegetable puree, containing red lentils, sweet potato, and carrot, homemade, mashed with lumps consistency	Australia	2010	36±4	4	Normal, 10 (adults)	25	201	Glucose, 2h	Standard	Capillary, plasma	Enzymatic	UO[5]
1268 Lentils and vegetables puree, commercial, for ages 7 – 9 months, Heinz™ Mum's Recipe (Heinz Wattie's Ltd, Australia)	Australia	2010	52±4	5	Normal, 10 (adults)	25	284	Glucose, 2h	Standard	Capillary, plasma	Enzymatic	UO[5]
1269 Farex™ baby rice (Heinz Wattie's Ltd., Malvern, Australia), prepared with 239 mL water	Australia	2010	74±7	7	Normal, 10 (adults)	25	30	Glucose, 2h	Standard	Capillary, plasma	Enzymatic	UO[5]
1270 Farex™ baby rice, 7 g cereal with 80 g water (Heinz Wattie's Ltd., Malvern, Australia)	Australia	2001	95±13	10	Normal, 11 (adults)	25	29.7	Glucose, 2h	Standard	Capillary, plasma	Enzymatic	UO[5]
1271 Organix™ organic wholegrain baby rice, prepared with 259 mL water	Australia	2010	74±8	7	Normal, 10 (adults)	25	30	Glucose, 2h	Standard	Capillary, plasma	Enzymatic	UO[5]
Rice cereal, mean of three foods		81										

1272	Rice pudding, Robinsons First Tastes from 4 months (Nutricia, Wells, UK)	UK	2001	59±6	6	Normal, 11 (adults)	25	170.4	Glucose, 2h	Standard	Capillary, plasma	Enzymatic	UO[5]
1273	Sweetcorn and rice, Heinz for Baby from 4 months (Heinz Wattie's Ltd, Australia)	Australia	2001	65±13	7	Normal, 11 (adults)	25	218.2	Glucose, 2h	Standard	Capillary, plasma	Enzymatic	UO[5]
1274	Sweet potato and pumpkin vegetable puree, homemade, puree consistency	Australia	2010	61±3	6	Normal, 10 (adults)	25	252	Glucose, 2h	Standard	Capillary, plasma	Enzymatic	UO[5]
1275	Pasta and vegetables puree, containing pasta, tomatoes, sweet potato, carrot and onion, homemade, pureed with soft pieces consistency	Australia	2010	54±7	5	Normal, 10 (adults)	25	209	Glucose, 2h	Standard	Capillary, plasma	Enzymatic	UO[5]
1276	Vegetable Mash, Harvest Veggies with Pasta puree, commercial, for ages 7 – 9 months, Heinz™ Mum's Recipe (Heinz Wattie's Ltd, Australia)	Australia	2010	46±4	5	Normal, 10 (adults)	25	275	Glucose, 2h	Standard	Capillary, plasma	Enzymatic	UO[5]
1277	Vegetable Mash, Heinz™ Organic Golden commercial, containing pumpkin and sweet potato puree, for aged 4 – 6 months	Australia	2010	43±5	4	Normal, 10 (adults)	25	240	Glucose, 2h	Standard	Capillary, plasma	Enzymatic	UO[5]

Food Number and Item		Country of food production	Year of test[1]	GI[2] ± SEM (Glu = 100)	GL	Subjects number)(type &	Avail carb (Test portion)	Test portion[3] (g)	Reference food & time period	Timepoints	Sample collection	Sample analysis method[4]	Ref.
	**LEGUMES**												
	Average available carbohydrate portion = 15 g, this value was used to determine the nominal GL for each item in this category.												
	**Baked Beans**												
1278	Baked Beans in Cheesy Tomato sauce (HJ Heinz, Australia)	Australia	2006	44±4	7	Normal, 9	50	318.5	Glucose, 2h	Standard	Capillary, whole blood	HemoCue	UO[10]
1279	Baked Beans in Barbecue sauce (HJ Heinz, Australia)	Australia	2006	47±4	7	Normal, 9	50	274.7	Glucose, 2h	Standard	Capillary, whole blood	HemoCue	UO[10]
1280	Baked Beans in Ham sauce (HJ Heinz, Australia)	Australia	2006	53±4	8	Normal, 10	50	299.4	Glucose, 2h	Standard	Capillary, whole blood	HemoCue	UO[10]
1281	Baked Beans in Sweet Chilli sauce (HJ Heinz, Australia)	Australia	2006	46±3	7	Normal, 10	50	292.4	Glucose, 2h	Standard	Capillary, whole blood	HemoCue	UO[10]
1282	Baked Beans in Tomato sauce (HJ Heinz, Australia)	Australia	2006	40±5	6	Normal, 10	50	381.7	Glucose, 2h	Standard	Capillary, whole blood	HemoCue	UO[10]

1283	Baked Beans in Tomato sauce, canned, reheated in microwave for 1.5 min (HJ Heinz, Australia)	Australia	2003	57±7	9	Normal, 10	50	322.6	Glucose, 2h	Standard	Capillary, whole blood	HemoCue	UO[5]
	Baked Beans, mean of six foods			48									
	**Chickpeas and chickpea products**												
1284	Chickpeas, canned, drained (Edgell, Simplot Australia, Australia)	Australia	2018	35±3	5	Normal, 12	25	131.6	Glucose, 2h	Standard	Capillary, plasma	Enzymatic	UO[5]
1285	Chickpeas (Garbanzo beans, Bengal gram), canned, drained, Edgell's™ brand (Simplot Australia, Australia)	Australia	2003	38±3	6	Normal, 10	25	168.9	Glucose, 2h	Standard	Capillary, whole blood	HemoCue	UO[5]
1286	Chickpeas, dried, roasted (‘chicknuts’)	Crete	2006	39±4	6	Normal, 10	50	82.0	Glucose, 2h	Standard	Capillary, plasma	Enzymatic	UO[5]
1287	Chickpea Hommus dip, Chris' Traditional brand (Capitol Chilled Foods Pty Ltd, ACT, Australia)	Australia	2007	22±5	3	Normal, 11	25	140.4	Glucose, 2h	Standard	Capillary, plasma	Enzymatic	UO[5]
1288	Hummus (Sabra Classic Hummus; Sabra Dipping Co., S. Chesterfield, VA)	USA	2016	15±3	2	Normal, 10	25	259	Bread, 2h	Standard	Capillary, plasma	YSI	100
	**Kidney Beans**												
1289	Dark Red Kidney beans, canned in brine, drained, Edgell's™ brand (Simplot Australia, Australia)	Australia	2003	43±5	6	Normal, 10	25	148.9	Glucose, 2h	Standard	Capillary, whole blood	HemoCue	UO[5]
1290	Red Kidney beans, canned, drained, Edgell's™ brand (Simplot Australia, Australia)	Australia	2003	36±4	5	Normal, 10	25	165.6	Glucose, 2h	Standard	Capillary, whole blood	HemoCue	UO[5]

**Food Number and Item**		**Country of food production**	**Year of test[1]**	**GI[2] ± SEM** (Glu = 100)	**GL**	**Subjects** (type & number)	**Avail carb** (Test portion)	**Test portion[3]** (g)	**Reference food & time period**	**Timepoints**	**Sample collection**	**Sample analysis method[4]**	**Ref.**
	**Lentils, boiled**												
1291	Lentil, Asterix, washed, brought to boil in water (1:2.5 seed:water ratio) at 500°C (~11 min), once boiling was achieved temperature reduced to 280°C and continued to boil until soft (Saskatchewan Pulse Growers, Canada)	Canada	2017*	10±3	2	Normal, 10	25	170.1	Bread, 2h	Standard	Capillary, whole blood	YSI	101
1292	Lentil, Greenland, washed, brought to boil in water (1:2.5 seed:water ratio) at 500°C (~11 min), once boiling was achieved temperature reduced to 280°C and continued to boil until soft (Saskatchewan Pulse Growers, Canada)	Canada	2017*	23±5	3	Normal, 10	25	192.3	Bread, 2h	Standard	Capillary, whole blood	YSI	101

1293	Lentil, Imigreen, washed, brought to boil in water (1:2.5 seed:water ratio) at 500°C (~11 min), once boiling was achieved temperature reduced to 280°C and continued to boil until soft (Saskatchewan Pulse Growers, Canada)	Canada	2017*	14±3	2	Normal, 10	25	158.2	Bread, 2h	Standard	Capillary, whole blood	YSI	101
1294	Lentil, Impower, washed, brought to boil in water (1:2.5 seed:water ratio) at 500°C (~11 min), once boiling was achieved temperature reduced to 280°C and continued to boil until soft (Saskatchewan Pulse Growers, Canada)	Canada	2017*	17±4	3	Normal, 10	25	153.4	Bread, 2h	Standard	Capillary, whole blood	YSI	101
1295	Lentil, Improve, washed, brought to boil in water (1:2.5 seed:water ratio) at 500°C (~11 min), once boiling was achieved temperature reduced to 280°C and continued to boil until soft (Saskatchewan Pulse Growers, Canada)	Canada	2017*	15±5	2	Normal, 10	25	206.6	Bread, 2h	Standard	Capillary, whole blood	YSI	101
1296	Lentil, Redberry, washed, brought to boil in water (1:2.5 seed:water ratio) at 500°C (~11 min), once boiling was achieved temperature reduced to 280°C and continued to boil until soft (Saskatchewan Pulse Growers, Canada)	Canada	2017*	13±2	2	Normal, 10	25	200	Bread, 2h	Standard	Capillary, whole blood	YSI	101
1297	Lentil, Redbow, washed, brought to boil in water (1:2.5 seed:water ratio) at 500°C (~11 min), once boiling was achieved temperature reduced to 280°C and continued to boil until soft (Saskatchewan Pulse Growers, Canada)	Canada	2017*	18±3	3	Normal, 10	25	204.9	Bread, 2h	Standard	Capillary, whole blood	YSI	101
1298	Lentil, Redcliff, washed, brought to boil in water (1:2.5 seed:water ratio) at 500°C (~11 min), once boiling was achieved temperature reduced to 280°C and continued to boil until soft (Saskatchewan Pulse Growers, Canada)	Canada	2017*	18±3	3	Normal, 10	25	188	Bread, 2h	Standard	Capillary, whole blood	YSI	101
	Lentils, boiled, mean of eight foods			16									
1299	Lentils, brown, canned, drained, Edgell's™ brand (Simplot Australia, Australia)	Australia	2008	42±5	6	Normal, 10	25	182.5	Glucose, 2h	Standard	Capillary, plasma	Enzymatic	UO[5]

Food Number and Item		Country of food production	Year of test[1]	GI[2] ± SEM (Glu = 100)	GL	Subjects (type & number)	Avail carb (Test portion)	Test portion[3] (g)	Reference food & time period	Timepoints	Sample collection	Sample analysis method[4]	Ref.
	**Mung beans**												
1300	Mung bean, fried	Australia	1998	53±8	8	Normal, 10	50	NS	Bread, 2h	Standard	Capillary, plasma	Enzymatic	UO[5]

1301	Mung bean, germinated	Australia	1998	25±4	4	Normal, 10	50	NS	Bread, 2h	Standard	Capillary, plasma	Enzymatic	UO[5]
1302	Mung bean, pressure cooked	Australia	1998	42±5	6	Normal, 10	50	NS	Bread, 2h	Standard	Capillary, plasma	Enzymatic	UO[5]
	**Legumes, other**												
1303	Broad beans, frozen, heated in microwave (Logan Farm Pty Ltd, Australia)	Australia	2007	63±9	9	Normal, 10	25	423.7	Glucose, 2h	Standard	Capillary, plasma	Enzymatic	UO[5]
	Butter beans, canned, drained, Edgell's™ brand (Simplot 1304 Australia, Cheltenham, Australia)	Australia	2003	36±3	5	Normal, 10	25	163.4	Glucose, 2h	Standard	Capillary, whole blood	HemoCue	UO[5]
1305	Cannellini beans, canned, drained, Edgell's™ brand (Simplot Australia, Cheltenham, Australia)	Australia	2003	31±5	5	Normal, 10	25	178.6	Glucose, 2h	Standard	Capillary, whole blood	HemoCue	UO[5]
1306	Four bean mix, canned, drained, Edgell's™ brand (Simplot Australia, Australia)	Australia	2003	37±5	6	Normal, 10	25	154.3	Glucose, 2h	Standard	Capillary, whole blood	HemoCue	UO[5]
1307	Freekeh, Lentils & Beans Superblend Fibre, dried, cooked in boiling water for 15 min (Ward McKenzies Pty Ltd, Australia)	Australia	2016	29±3	4	Normal, 10	25	50.7	Glucose, 2h	Standard	Capillary, plasma	Enzymatic	UO[5]
1308	Lentils, Quinoa & Beans Superblend Protein, dried, cooked in boiling water for 15 min (Ward McKenzies Pty Ltd, Australia)	Australia	2017	36±4	5	Normal, 10	25	53.1	Glucose, 2h	Standard	Capillary, plasma	Enzymatic	UO[5]
1309	Refried Pinto beans, Casa Fiesta™ brand (Capital Foods Pty Ltd, Australia), heated in microwave for 1.5 min	Australia	2006	38±3	6	Normal, 10	50	285.7	Glucose, 2h	Standard	Capillary, plasma	Enzymatic	UO[5]
	**DUCTS**												
	**MEAL REPLACEMENT & WEIGHT MANAGEMENT PRO**												
	Average available carbohydrate portion = 20 g, this value was used to determine the nominal GL for each item in this category.												
	**Meal replacement bars, cookies and desserts**												
1310	Chocolate weight management bar (Shaklee Corporation, Pleasanton, CA, USA)	USA	2006	29±4	6	Normal, 9	50	129.2	Glucose, 2h	Standard	Capillary, plasma	Enzymatic	UO[5]
1311	Hazelnut & Apricot bar (Dietworks, South Yarra, Australia)	Australia	1999	42±7	8	Normal, 10	50	105.0	Bread, 2h	Standard	Capillary, plasma	Enzymatic	UO[5]
1312	L.E.A.N Fibergy™ bar, Harvest Oat (Usana Inc., Salt Lake	USA	2000	45±4	9	Normal, 10	50	85.7	Glucose, 2h	Standard	Capillary, plasma	Enzymatic	UO[5]

City, UT, USA)

Food Number and Item		Country of food production	Year of test[1]	GI[2] ± SEM (Glu = 100)	GL	Subjects (type & number)	Avail carb (Test portion)	Test portion[3] (g)	Reference food & time period	Timepoints	Sample collection	Sample analysis method[4]	Ref.
1313	L.E.A.N (Life long) Nutribar™, Peanut Crunch (Usana Inc., Salt Lake City, UT, USA)	USA	2000	30±4	6	Normal, 10	50	250	Glucose, 2h	Standard	Capillary, plasma	Enzymatic	UO[5]
1314	L.E.A.N (Life long) Nutribar™, Chocolate Crunch (Usana Inc., Salt Lake City, UT, USA)	USA	2000	32±4	6	Normal, 10	50	107.9	Glucose, 2h	Standard	Capillary, plasma	Enzymatic	UO[5]
1315	Lemon weight management bar (Shaklee Corporation, USA)	USA	2006	23±4	5	Normal, 10	50	129.2	Glucose, 2h	Standard	Capillary, plasma	Enzymatic	UO[5]
1316	Optifast® VLCD™ Berry Crunch Flavor Bar (Nestlé Health Science, Australia)	Australia	2013	25±2	5	Normal, 10	50	144.1	Glucose, 2h	Standard	Capillary, plasma	Enzymatic	UO[5]
1317	Optifast® VLCD™ Cappuccino Flavor Bar (Nestlé Health Science, Australia)	Australia	2013	29±4	6	Normal, 10	50	147.1	Glucose, 2h	Standard	Capillary, plasma	Enzymatic	UO[5]
1318	Optifast® VLCD™ Cereal with Cranberry Bar (Nestlé Health Science, Australia)	Australia	2018	30±3	6	Normal, 10	25	88.3	Glucose, 2h	Standard	Capillary, plasma	Enzymatic	UO[5]
1319	Optifast® VLCD™ Chocolate Bar (Nestlé Health Science, Australia)	Australia	2013	20±2	4	Normal, 10	25	124.4	Glucose, 2h	Standard	Capillary, plasma	Enzymatic	UO[5]
1320	Optifast® VLCD™ Chocolate Dessert (Nestlé Health Science, Australia)	Australia	2013	27±4	5	Normal, 10	50	135.3	Glucose, 2h	Standard	Capillary, plasma	Enzymatic	UO[5]
1321	Optifast® VLCD™ Lemon Crème Dessert (Nestlé Health Science, Australia)	Australia	2013	27±4	5	Normal, 10	50	135.3	Glucose, 2h	Standard	Capillary, plasma	Enzymatic	UO[5]
1322	Peanut Butter weight management bar (Shaklee Corporation, USA)	USA	2006	22±4	4	Normal, 10	50	129.2	Glucose, 2h	Standard	Capillary, plasma	Enzymatic	UO[5]
1323	Protein Meal Replacement Cookies (Bao Bei Er (Chengdu Tianyi Cuisine Nutritional Food, China)	China	2019	43±4	9	Normal, 12	25	68.2	Glucose, 2h	Standard	Capillary, plasma	Enzymatic	UO[18]
	SlimFast® bar, chocolate caramel meal replacement bar 1324 (SlimFast Foods Ltd, Slough, Berks, UK)	UK	2003	54±3	11	Normal, 10	50	75.6	Glucose, 2h	Standard	Capillary, plasma	Enzymatic	UO[5]

	SlimFast® bar, chocolate meal replacement bar (SlimFast 1325 Foods Ltd, UK)	UK	2003	27±3	5	Normal, 10	50	108.2	Glucose, 2h	Standard	Capillary, plasma	Enzymatic	UO[5]
1326	SlimFast® bar, Chocolate Muesli snack bar (SlimFast Foods Ltd, UK)	UK	2003	49±6	10	Normal, 10	50	77.5	Glucose, 2h	Standard	Capillary, plasma	Enzymatic	UO[5]
	**Meal replacement beverages**												
	Aussie Bodies Trim Protein Shake, Chocolate flavored 1327 (Aussie Bodies Pty Ltd, Australia)	Australia	20062007	39±4	8	Normal, 10	50	146.2	Glucose, 2h	Standard	Capillary, whole blood	HemoCue	UO[10]
1328	Aussie Bodies Trim Protein Shake, French Vanilla flavored (Aussie Bodies Pty Ltd, Australia)	Australia	2006-2007	41±3	8	Normal, 10	50	142.9	Glucose, 2h	Standard	Capillary, whole blood	HemoCue	UO[10]
1329	Formula 1 Healthy Meal Nutritional Shake Mix: Doce de leite (Herbalife International of America, Inc.)	Brazil	2020	22±3	4	Normal, 10	25	98.4	Glucose, 2h	Standard	Capillary, plasma	YSI	UO[7]

Food Number and Item		Country of food production	Year of test[1]	GI[2] ± SEM (Glu = 100)	GL	Subjects (type & number)	Avail carb (Test portion)	Test portion[3] (g)	Reference food & time period	Timepoints	Sample collection	Sample analysis method[4]	Ref.
1330	Formula 1 Healthy Meal Nutritional Shake Mix: Caramel Apple (Herbalife International of America, Inc.)	USA	2018	15±2	3	Normal, 10	25	62.5	Glucose, 2h	Standard	Capillary, whole blood	YSI	UO[7]
1331	Formula 1 Healthy Meal Nutritional Shake Mix: Pralines & Cream (Herbalife International of America, Inc.)	USA	2018	15±2	3	Normal, 10	25	62.5	Glucose, 2h	Standard	Capillary, whole blood	YSI	UO[7]
1332	Formula 1 Healthy Meal Nutritional Shake Mix: Cookies and Cream (1 serve) + Protein Drink Mix: Vanilla (1/2 serve) + Prolessa Duo (1 serve) (Herbalife International of America, Inc.)	USA	2019	29±4	6	Normal, 10	25	104.2	Glucose, 2h	Standard	Capillary, plasma	YSI	UO[7]
1333	Formula 1 Healthy Meal Nutritional Shake Mix: Cookies and Cream (1 serve) + Protein Drink Mix: Vanilla (1 serve) + Prolessa Duo (1 serve) (Herbalife International of America, Inc.)	USA	2019	29±4	6	Normal, 10	25	133.3	Glucose, 2h	Standard	Capillary, plasma	YSI	UO[7]
1334	Formula 1 Healthy Meal Nutritional Shake Mix: French Vanilla (1 serve) + Protein Drink Mix: Vanilla (1/2 serve) + Prolessa Duo (1 serve) (Herbalife International of America, Inc.)	USA	2019	32±4	6	Normal, 10	25	132.5	Glucose, 2h	Standard	Capillary, plasma	YSI	UO[7]
1335	Formula 1 Healthy Meal Nutritional Shake Mix: French Vanilla (1 serve) + Protein Drink Mix: Vanilla (1 serve) + Prolessa Duo (1 serve) (Herbalife International of America, Inc.)	USA	2019	29±3	6	Normal, 10	25	139.6	Glucose, 2h	Standard	Capillary, plasma	YSI	UO[7]

Food Number and Item		Country of food production	Year of test[1]	GI[2] ± SEM (Glu = 100)	GL	Subjects (type & number)	Avail carb (Test portion)	Test portion[3] (g)	Reference food & time period	Timepoints	Sample collection	Sample analysis method[4]	Ref.
1336	Formula 1 Nutritional Shake Mix: Berry (Herbalife Australasia Pty Ltd, Australia), prepared with water	Australia	2014	21±2	4	Normal, 10	25	46.7	Glucose, 2h	Standard	Capillary, plasma	Enzymatic	UO[5]
1337	Formula 1 Nutritional Shake Mix: Cookies n' Cream (Herbalife Australasia Pty Ltd, Australia), prepared with water	Australia	2014	15±2	3	Normal, 10	25	46.7	Glucose, 2h	Standard	Capillary, plasma	Enzymatic	UO[5]
1338	Formula 1 Nutritional Shake Mix: Dutch Chocolate (Herbalife Australasia Pty Ltd, Australia), prepared with water	Australia	2014	19±2	4	Normal, 10	25	50.0	Glucose, 2h	Standard	Capillary, plasma	Enzymatic	UO[5]
1339	Formula 1 Nutritional Shake Mix: French Vanilla (Herbalife Australasia Pty Ltd, Australia), prepared with water	Australia	2014	20±2	4	Normal, 11	25	46.7	Glucose, 2h	Standard	Capillary, plasma	Enzymatic	UO[5]
1340	Fruit & Vegetable cereal powder A (The Fast Diet A, Ebany Biomedical Technology, Beijing, China)	China	2019	35±4	7	Normal, 10	25	45.5	Glucose, 2h	Standard	Capillary, plasma	Enzymatic	UO[7]
1341	Fruit & Vegetable cereal powder B (The Fast Diet A, Ebany Biomedical Technology, Beijing, China)	China	2019	47±3	9	Normal, 10	25	45.5	Glucose, 2h	Standard	Capillary, plasma	Enzymatic	UO[7]
1342	Herbalife24 Achieve Protein Bar: Dark Chocolate Brownie (Herbalife International of America, Inc.)	USA	2017	49±8	10	Normal, 10	25	141.7	Glucose, 2h	Standard	Capillary, whole blood	YSI	UO[7]
1343	Herbalife24 Achieve Protein Bar: Chocolate Chip Cookie Dough (Herbalife International of America, Inc.)	USA	2017	42±6	8	Normal, 10	25	138.5	Glucose, 2h	Standard	Capillary, whole blood	YSI	UO[7]
1344	Isowhey Complete Double Chocolate meal replacement beverage powder, prepared with water (FIT Bioceuticals Pty Ltd, Australia)	Australia	2009	23±3	5	Normal, 10	25	136.2	Glucose, 2h	Standard	Capillary, plasma	Enzymatic	UO[5]
1345	Isowhey Complete Strawberries & Cream meal replacement beverage powder, prepared with water (FIT Bioceuticals Pty Ltd, Australia)	Australia	2009	30±4	6	Normal, 10	25	101.2	Glucose, 2h	Standard	Capillary, plasma	Enzymatic	UO[5]
1346	Isowhey Complete French Vanilla meal replacement beverage powder, prepared with water (FIT Bioceuticals Pty Ltd, Australia)	Australia	2009	30±4	6	Normal, 10	25	101.2	Glucose, 2h	Standard	Capillary, plasma	Enzymatic	UO[5]
1347	Juice Plus+® Complete meal replacement powder, Dutch chocolate flavor	Australia	2004	35±5	7	Normal, 10	25	73.3	Glucose, 2h	Standard	Capillary, whole blood	HemoCue	UO[5]

Food Number and Item		Country of food production	Year of test[1]	GI[2] ± SEM (glu = 100)	GL	Subjects (type & number)	Avail carb (Test portion)	Test portion[3] (g)	Reference food & time period	Timepoints	Sample collection	Sample analysis method[4]	Ref.
1348	Juice Plus+® Complete meal replacement powder, French vanilla flavor	Australia	2004	32±4	6	Normal, 10	25	59.0	Glucose, 2h	Standard	Capillary, whole blood	HemoCue	UO[5]
1349	Meal Shake Solid Beverage, Lychee Rose (Mary Kay, China)	China	2020	44±4	9	Normal, 12	25	73.8	Glucose, 2h	Standard	Capillary, plasma	Enzymatic	UO[18]
1350	Nongfu Spring Meal Replacement, low GI (Nongfu Spring Hangzhou, China)	China	2020	27±4	5	Normal, 12	25	358.7	Glucose, 2h	Standard	Capillary, plasma	Enzymatic	UO[18]
1351	Nutrimeal™, drink powder, Dutch Chocolate (Usana, Salt Lake City, USA)	USA	2000	26±3	5	Normal, 10	25	545.9	Glucose, 2h	Standard	Capillary, plasma	Enzymatic	UO[5]
1352	Nutrimeal™, meal replacement drink, all flavors, prepared with 600 mL water (Usana Australia Pty Ltd, Baulkham Hills, Australia)	USA	2007	20±2	4	Normal, 10	50	126.9	Glucose, 2h	Standard	Capillary, plasma	Enzymatic	UO[5]
1353	Nutrimeal™ Free Vanilla vegan meal replacement beverage powder, prepared with 434.5 mL water (Usana Australia Pty Ltd, Baulkham Hills, Australia)	USA	2013	49±4	10	Normal, 10	25	73.1	Glucose, 2h	Standard	Capillary, plasma	Enzymatic	UO[5]
1354	Optifast® VLCD™ Shake, Banana (Nestlé Health Science, Australia), prepared with water	Australia	2012	24±3	5	Normal, 10	50	120.2	Glucose, 2h	Standard	Capillary, plasma	Enzymatic	UO[5]
1355	Optifast® VLCD™ Shake, Caramel (Nestlé Health Science, Australia), prepared with water	Australia	2014	34±4	7	Normal, 10	50	120.2	Glucose, 2h	Standard	Capillary, plasma	Enzymatic	UO[5]
1356	Optifast® VLCD™ Shake, Chocolate (Nestlé Health Science, Australia), prepared with water	Australia	2013	31±3	6	Normal, 10	50	120.2	Glucose, 2h	Standard	Capillary, plasma	Enzymatic	UO[5]
1357	Optifast® VLCD™ Shake, Coffee (Nestlé Health Science, Australia), prepared with water	Australia	2013	31±3	6	Normal, 10	50	120.2	Glucose, 2h	Standard	Capillary, plasma	Enzymatic	UO[5]
1358	Optifast® VLCD™ Shake, Strawberry (Nestlé Health Science, Australia), prepared with water	Australia	2013	27±3	5	Normal, 10	50	120.2	Glucose, 2h	Standard	Capillary, plasma	Enzymatic	UO[5]
1359	Optifast® VLCD™ Shake, Vanilla (Nestlé Health Science, Australia), prepared with water	Australia	2013	27±3	5	Normal, 10	50	120.2	Glucose, 2h	Standard	Capillary, plasma	Enzymatic	UO[5]

1360	Provide™ nutritious beverage powder, prepared with water (Indevex AB, Sweden)	Sweden	2004	28±5	6	Normal, 10	25	NS	Glucose, 2h	Standard	Capillary, plasma	Enzymatic	UO[5]
1361	SICCON D2™ nutritious beverage powder, prepared with water (Indevex AB, Sweden)	Sweden	2004	28±5	6	Normal, 10	25	NS	Glucose, 2h	Standard	Capillary, plasma	Enzymatic	UO[5]
1362	SlimFast® beverage, French Vanilla ready-to-drink shake (Slim Fast Foods Company, Englewood, NJ, USA)	USA	2006	37±5	7	Normal, 10	50	500.0	Glucose, 2h	Standard	Capillary, plasma	Enzymatic	UO[5]
1363	SlimFast® beverage, Strawberry Supreme ready-to-drink shake (SlimFast Foods Ltd, UK)	UK	2003	33±3	7	Normal, 9	50	445.0	Glucose, 2h	Standard	Capillary, plasma	Enzymatic	UO[5]
1364	Tony Ferguson's Meal Replacement Shake, all flavors, prepared with 356.2 mL water	Australia	2006	22±2	4	Normal, 10	50	92.6	Glucose, 2h	Standard	Capillary, plasma	Enzymatic	UO[5]
	**Meal replacement soups**												
1365	Optifast® VLCD™ Soup, Chicken (Nestlé Health Science, Australia), prepared with water	Australia	2013	31±3	6	Normal, 9	50	141.2	Glucose, 2h	Standard	Capillary, plasma	Enzymatic	UO[5]
1366	Optifast® VLCD™ Soup, Tomato (Nestlé Health Science, Australia), prepared with water	Australia	2014	25±3	5	Normal, 10	50	122.9	Glucose, 2h	Standard	Capillary, plasma	Enzymatic	UO[5]
1367	Optifast® VLCD™ Soup, Vegetable (Nestlé Health Science, Australia), prepared with water	Australia	2013	24±3	5	Normal, 10	50	120.0	Glucose, 2h	Standard	Capillary, plasma	Enzymatic	UO[5]
1368	SlimFast®, Garden vegetable soup with peppers and croutons (SlimFast Foods Ltd, UK), prepared with water	UK	2003	20±2	4	Normal, 10	50	119.0	Glucose, 2h	Standard	Capillary, plasma	Enzymatic	UO[5]

NUTRITIONAL SUPPORT PRODUCTS

Average available carbohydrate portion = 30 g, this value was used to determine the nominal GL for each item in this category.

1369	Diasip ready-to-drink (Nutricia Advanced Medical Nutrition, Zoetermeer, Netherlands)	Netherlands	2016*	34±4	10	Normal, Chinese, 16	50	427	Glucose, 2h	Standard	Capillary, whole blood	HemoCue	102
1370	Diasip, ready-to-drink (Nutricia Advanced Medical Nutrition, Zoetermeer, Netherlands)	Netherlands	2016*	33±6	10	Normal, Indian, 16	50	427	Glucose, 2h	Standard	Capillary, whole blood	HemoCue	102
1371	Diasip, ready-to-drink (Nutricia Advanced Medical Nutrition, Zoetermeer, Netherlands)	Netherlands	2016*	28±4	8	Normal, Malay, 16	50	427	Glucose, 2h	Standard	Capillary, whole blood	HemoCue	102
	Diasip, ready-to-drink, mean of 3 studies			32									
	Ensure™, vanilla (Abbott Australasia, Kurnell, NSW, 1372 Australia)	Australia	1998	50±8	15	Normal, 10	50	344.8	Bread, 2h	Standard	Capillary, plasma	Enzymatic	103

1373	Ensure™, vanilla (Abbott Australasia)	Australia	1999	48±3	14	Normal, 10	50	NS	Glucose, 2h	Standard	Capillary, whole blood	HemoCue	UO[5]
1374	Ensure™ bar, chocolate fudge brownie (Abbott Australasia)	Australia	2000	43±3	13	Normal, 10	50	97.4	Glucose, 2h	Standard	Capillary, plasma	Enzymatic	UO[5]

**Food Number and Item**		**Country of food production**	**Year of test[1]**	**GI[2] ± SEM** (Glu = 100)	**GL**	**Subjects** (type & number)	**Avail carb** (Test portion)	**Test portion[3]** (g)	**Reference food & time period**	**Timepoints**	**Sample collection**	**Sample analysis method[4]**	**Ref.**
1375	Ensure™ Hospital, nutritional supplement powder, prepared with 304.2 mL water (Abbott Nutrition International)	Australia	2005	51±5	15	Normal, 9	50	85.5	Glucose, 2h	Standard	Capillary, plasma	Enzymatic	UO[5]
1376	Ensure Plus™, vanilla (Abbott Australasia)	Australia	2000	40±4	12	Normal, 10	50	250	Glucose, 2h	Standard	Capillary, plasma	Enzymatic	UO[5]
1377	Ensure Pudding™, old fashioned vanilla (Abbott Laboratories Inc, Ashland, USA)	USA	2000	36±4	11	Normal, 10	50	216.5	Glucose, 2h	Standard	Capillary, plasma	Enzymatic	UO[5]
	GluCare Gold beverage powder (Nutricare Nutrition, 1378 Vietnam), prepared with water	Vietnam	2018	48±3	14	Normal, 10	50	77.4	Glucose, 2h	Standard	Capillary, plasma	Enzymatic	UO[5]
1379	Glucerna Chocolate Graham nutritional supplement bar (Ross Nutrition, Canada)	Canada	2002	20±2	6	Normal, 9	50	79.4	Glucose, 2h	Standard	Capillary, plasma	Enzymatic	UO[5]
1380	Glucerna, diabetes-specific enteral formula (Abbott Laboratories Inc, USA)	USA		15±3	5	Normal, 9	50	NS	Glucose, 2h	Standard	Capillary, plasma	Enzymatic	UO[5]
1381	Glucerna™, vanilla flavor (Abbott Nutrition International)	Australia	2005	19±4	6	Normal, 10	25	319.4	Glucose, 2h	Standard	Capillary, plasma	Enzymatic	UO[5]
1382	Glucerna™, vanilla flavor (Abbott Nutrition International)	Japan	2005	20±4	6	Normal, 10	25	327.3	Glucose, 2h	Standard	Capillary, plasma	Enzymatic	UO[5]
	Glucerna Nutritional Drink, Vanilla Flavor (Abbott 1383 Laboratories)	Canada	2018	23±4	7	Normal, 10	25	257.6	Glucose, 2h	Standard	Capillary, whole blood	YSI	UO[7]
1384	Glucerna™, vanilla (Abbott Laboratories Inc., USA)	USA	2005	31±2	9	Normal, 10	25	NS	Glucose, 2h	Standard	Capillary, plasma	Enzymatic	UO[5]
	Vanilla Glucerna™, mean of four studies			23									
	Glucerna Select, enteral nutritional supplement (Abbott 1385 Nutrition International)	Australia	2006	16±3	5	Normal, 9	25	395.6	Glucose, 2h	Standard	Capillary, plasma	Enzymatic	UO[5]
	Glucerna SR, diabetes-specific enteral formula (Abbott 1386 Laboratories Inc., USA)	USA	2002	23±5	7	Normal, 9	25	502.2	Glucose, 2h	Standard	Capillary, plasma	Enzymatic	UO[5]

Food Number and Item		Country of food production	Year of test[1]	GI[2] ± SEM (Glu = 100)	GL	Subjects (type & number)	Avail carb (Test portion)	Test portion[3] (g)	Reference food & time period	Timepoints	Sample collection	Sample analysis method[4]	Ref.
1387	High protein, high fiber nutritional supplement (flavor 1) mixed in 670 mL water (Dr. Reddy's Laboratories, India)	India	2019*	27±5	8	Normal, 15	25	134	Glucose, 2h	Standard	Capillary, whole blood	HemoCue	104
1388	High protein, high fiber nutritional supplement (flavor 2) mixed in 765 mL water (Dr. Reddy's Laboratories, India)	India	2019*	37±4	11	Normal, 15	25	153	Glucose, 2h	Standard	Capillary, whole blood	HemoCue	104
1389	HINEX Diabetes Care Vanilla powder (Otsuka Pharmaceutical India Pvt. Ltd.), prepared with 457.8 mL water	India	2020	36±4	11	Normal, 10	50	120.5	Glucose, 2h	Standard	Capillary, plasma	Enzymatic	UO[5]
1390	Inslow™ (Abbott Nutrition International -Japan)	Japan	2005	25±3	8	Normal, 9	25	246.1	Glucose, 2h	Standard	Capillary, plasma	Enzymatic	UO[5]
1391	Isocal, ready-to-drink (Nestlé Nutrition, Tainan, Taiwan)	Taiwan	2016*	79±9	24	Normal, Chinese, 16	50	370	Glucose, 2h	Standard	Capillary, whole blood	HemoCue	102
1392	Isocal, ready-to-drink (Nestlé Nutrition, Tainan, Taiwan)	Taiwan	2016*	82±13	25	Normal, Indian, 16	50	370	Glucose, 2h	Standard	Capillary, whole blood	HemoCue	102
1393	Isocal, ready-to-drink (Nestlé Nutrition, Tainan, Taiwan)	Taiwan	2016*	85±9	26	Normal, Malay, 16	50	370	Glucose, 2h	Standard	Capillary, whole blood	HemoCue	102
	Isocal, ready-to-drink, mean of three studies			82									
1394	Jevity™ (Abbott Australasia)	Australia	2000	48±3	14	Normal, 10	50	364.2	Glucose, 2h	Standard	Capillary, plasma	Enzymatic	UO[5]
1395	Jevity™ HiCal, enteral nutritional supplement (Abbott Nutrition International)	Australia	2005	59±6	18	Normal, 9	50	269.4	Glucose, 2h	Standard	Capillary, plasma	Enzymatic	UO[5]
1396	Lactona Dia-β beverage powder, tested prepared with 269.6 mL water (PT Mirota KSM, Indonesia)	Indonesia	2010	27±3	8	Normal, 11	50	80.9	Glucose, 2h	Standard	Capillary, plasma	Enzymatic	UO[5]
1397	Nutritional beverage powder (SU YOUTH (Ningbo Yu Fang Tang Biological Science and Technology, China)	China	2019	52±5	16	Normal, 12	25	62.5	Glucose, 2h	Standard	Capillary, plasma	Enzymatic	UO18
1398	Peptide 1.5 Plain nutrition formula (Kate Farms, Inc.)	USA	2019	26±4	8	Normal, 10	25	207.4	Glucose, 2h	Standard	Capillary, plasma	YSI	UO[7]
1399	Promote with fiber™ nutritional supplement (Ross Nutrition, USA)	USA	2005	49±8	15	Normal, 10	50	389.1	Glucose, 2h	Standard	Capillary, plasma	Enzymatic	UO[5]
1400	Prosure™, ready-to-drink supplement, vanilla flavor (Abbott Nutrition International)	USA	2005	54±5	16	Normal, 10	50	317.3	Glucose, 2h	Standard	Capillary, plasma	Enzymatic	UO[5]

Food Number and Item		Country of food production	Year of test[1]	GI[2] ± SEM (Glu = 100)	GL	Subjects (type & number)	Avail carb (Test portion)	Test portion[3] (g)	Reference food & time period	Timepoints	Sample collection	Sample analysis method[4]	Ref.
1401	Prosure™, ready-to-drink supplement, vanilla flavor (Abbott Nutrition International)	Australia	2005	55±5	17	Normal, 9	50	283.5	Glucose, 2h	Standard	Capillary, plasma	Enzymatic	UO[5]
	Resource Diabetic™, French vanilla (Novartis Nutrition 1402 Corp., Young America, USA), prepared with water	USA	2000	34±3	10	Normal, 10	25	29.1	Glucose, 2h	Standard	Capillary, plasma	Enzymatic	UO[5]
1403	Resource Diabetic™, Swiss chocolate (Novartis, Auckland, New Zealand)	New Zealand	2000	16±4	5	Normal, 11	50	NS	Glucose, 2h	Standard	Venous, plasma	Enzymatic	27
1404	Resource™ fruit beverage, peach flavor (Novartis, New Zealand)	New Zealand	2000	40±8	12	Normal, 11	50	NS	Glucose, 2h	Standard	Venous, plasma	Enzymatic	27
	Resource™ thickened orange juice, honey consistency 1405 (Novartis, New Zealand)	New Zealand	2000	47±9	14	Normal, 11	50	NS	Glucose, 2h	Standard	Venous, plasma	Enzymatic	27
	Resource™ thickened orange juice, nectar consistency 1406 (Novartis, New Zealand)	New Zealand	2000	54±7	16	Normal, 11	50	NS	Glucose, 2h	Standard	Venous, plasma	Enzymatic	27
1407	SDC (SDS (SUSTRA™ 2434 slowly digestible carbohydrate; blend of tapioca flour and corn starch) (Ingredion Incorporated, Bridgewater, NJ), prepared with water	USA	2019*	27±2	8	Normal, 14	50	473.2	Glucose, 2h	Standard	Capillary, whole blood	YSI	105
1408	Sport beverage based on oligosaccharides (low molecular weight carbohydrate), orange flavor (CPT, Competitor Co. Ltd., Beijing, China)	China	2017*	118±14	35	Normal, 10	50	NS	Glucose, 2h	Standard	Capillary, whole blood	YSI	22
1409	Sport beverage based on hydrolyzed starch (high molecular weight carbohydrate), orange flavor (Vitargo, Swecarb AB, Kalmar, Sweden)	Sweden	2017*	106±13	32	Normal, 10	50	NS	Glucose, 2h	Standard	Capillary, whole blood	YSI	22
1410	Standard 1.0 Chocolate nutrition formula (Kate Farms, Inc., USA)	USA	2019	31±3	9	Normal, 10	25	249.7	Glucose, 2h	Standard	Capillary, plasma	YSI	UO[7]
	Standard 1.0 Vanilla nutrition formula (Kate Farms, Inc., 1411 USA)	USA	2019	26±2	8	Normal, 10	25	267.1	Glucose, 2h	Standard	Capillary, plasma	YSI	UO[7]
1412	Standard 1.4 Plain nutrition formula (Kate Farms, Inc., USA)	USA	2019	51±5	15	Normal, 10	25	182.3	Glucose, 2h	Standard	Capillary, plasma	YSI	UO[7]
1413	Sustagen® Diabetic, Vanilla (Nestlé Health Science, Australia), prepared with water	Australia	2013	34±4	10	Normal, 10	50	112.4	Glucose, 2h	Standard	Capillary, plasma	Enzymatic	UO[5]

Food Number and Item		Country of food production	Year of test[1]	GI[2] ± SEM (Glu = 100)	GL	Subjects (type & number)	Avail carb (Test portion)	Test portion[3] (g)	Reference food & time period	Timepoints	Sample collection	Sample analysis method[4]	Ref.
1414	Sustagen® Diabetic Ready To Drink, Vanilla (Nestlé Health Science, Australia)	Australia	2013	30±3	9	Normal, 10	50	542.3	Glucose, 2h	Standard	Capillary, plasma	Enzymatic	UO[5]
1415	Sustagen™, Dutch Chocolate (Mead Johnson, Bristol Myers Squibb, Rydalmere, Australia)	Australia	1999	31±4	9	Normal, 10	50	303.0	Bread, 2h	Standard	Capillary, plasma	Enzymatic	UO[5]
1416	Sustagen® Hospital with extra fiber, drink made from powdered mix (Mead Johnson, Australia), prepared with water	Australia	1999	33±4	10	Normal, 10	50	101.0	Bread, 2h	Standard	Capillary, plasma	Enzymatic	UO[5]
1417	Sustagen® Hospital Formula Active, Banana (Nestlé Health Science, Australia), prepared with water	Australia	2018	46±3	14	Normal, 10	50	76.9	Glucose, 2h	Standard	Capillary, plasma	Enzymatic	UO[5]
1418	Sustagen® Hospital Formula Active, Chocolate (Nestlé Health Science, Australia), prepared with water	Australia	2015	49±5	15	Normal, 10	50	76.9	Glucose, 2h	Standard	Capillary, plasma	Enzymatic	UO[5]
1419	Sustagen® Hospital Formula Active, Coffee (Nestlé Health Science, Australia), prepared with water	Australia	2015	48±3	14	Normal, 10	50	76.9	Glucose, 2h	Standard	Capillary, plasma	Enzymatic	UO[5]
1420	Sustagen® Hospital Formula Active, Neutral (Nestlé Health Science, Australia), prepared with water	Australia	2012	46±5	14	Normal, 9	50	76.9	Glucose, 2h	Standard	Capillary, plasma	Enzymatic	UO[5]
	Sustagen® Hospital Formula Active, Strawberry (Nestlé 1421 Health Science, Australia), prepared with water	Australia	2016	37±3	11	Normal, 10	50	76.9	Glucose, 2h	Standard	Capillary, plasma	Enzymatic	UO[5]
1422	Sustagen® Hospital Formula Active, Vanilla (Nestlé Health Science, Australia), prepared with water	Australia	2015	54±3	16	Normal, 10	50	76.9	Glucose, 2h	Standard	Capillary, plasma	Enzymatic	UO[5]
1423	Sustagen® Hospital Formula Active Plus Fibre, Chocolate (Nestlé Health Science, Australia), prepared with water	Australia	2015	42±5	13	Normal, 10	50	81.2	Glucose, 2h	Standard	Capillary, plasma	Enzymatic	UO[5]
	Sustagen® Hospital Formula Active Plus Fibre, Vanilla 1424 (Nestlé Health Science, Australia), prepared with water	Australia	2015	45±4	14	Normal, 10	50	80.5	Glucose, 2h	Standard	Capillary, plasma	Enzymatic	UO[5]
1425	Sustagen® Instant Pudding, vanilla, made from powdered mix (Mead Johnson, Australia), prepared with water	Australia	1999	27±3	8	Normal, 10	50	84.7	Bread, 2h	Standard	Capillary, plasma	Enzymatic	UO[5]

1426	Sustagen® Optimum, Vanilla (Nestlé Health Science, Australia), prepared with water	Australia	2014	45±5	14	Normal, 10	50	94.0	Glucose, 2h	Standard	Capillary, plasma	Enzymatic	UO[5]
1427	Sustagen® Ready to Drink, Vanilla (Nestlé Health Science, Australia)	Australia	2012	39±4	12	Normal, 10	50	330	Glucose, 2h	Standard	Capillary, plasma	Enzymatic	UO[5]
1428	Tapion™ nutritional supplement (Abbott Nutrition International - Japan)	Japan	2005	27±5	8	Normal, 9	25	240.9	Glucose, 2h	Standard	Capillary, plasma	Enzymatic	UO[5]
1429	TwoCal HN™, high nitrogen nutritional supplement, vanilla flavor (Abbott Nutrition International)	Australia	2005	55±5	17	Normal, 10	50	246.7	Glucose, 2h	Standard	Capillary, plasma	Enzymatic	UO[5]
1430	ViPlus Diabetic Formula, Vanilla flavor, prepared with water (Viplus Dairy Pty Ltd, Australia)	Australia	2016	42±2	13	Normal, 10	50	102.5	Glucose, 2h	Standard	Capillary, plasma	Enzymatic	UO[5]
	**NUTS**												
	Average carbohydrate portion = 5 g, this value was used to determine the nominal GL for each item in this c tegory.												
1431	Cashew nuts, salted (Coles Supermarkets, Australia)	Australia	2001	22±5	1	Normal, 10	25	98.0	Glucose, 2h	Standard	Capillary, plasma	Enzymatic	UO[5]
1432	Mixed nuts and raisins	UK	2005	21±5	1	Normal, 9	50	161.3	Glucose, 2h	Standard	Capillary, whole blood	Glucometer[9]	26
1433	Mixed nuts, roasted and salted	UK	2005	24±10	1	Normal, 9	25	128.2	Glucose, 2h	Standard	Capillary, whole blood	Glucometer[9]	26
	**PASTA and NOODLES**												
	Average carbohydrate portion = 40 g, this value was used to determine the nominal GL for each item in this ategory.												
	**Barley pasta**												
1434	Barley pasta (celebrity, whole grain fraction), cooked 5 min	Canada	2012*	71±6	28	Normal, 10	50	NS	Bread, 2h	Standard	Capillary, whole blood	YSI	61
1435	Barley pasta (celebrity white pearled fraction), cooked 5 min	Canada	2012*	58±4	23	Normal, 10	50	NS	Bread, 2h	Standard	Capillary, whole blood	YSI	61
1436	Barley pasta (AC Parkhill, whole grain fraction), cooked 5 min	Canada	2012*	73±7	29	Normal, 10	50	NS	Bread, 2h	Standard	Capillary, whole blood	YSI	61
1437	Barley pasta (AC Parkhill, white pearled fraction), cooked 5 min	Canada	2012*	64±4	26	Normal, 10	50	NS	Bread, 2h	Standard	Capillary, whole blood	YSI	61
1438	70%+ Black Highland Barley Noodles (COFCO Flour Industry, Puyang, China)	China	2020	46±3	18	Normal, 12	50	67.3	Glucose, 2h	Standard	Capillary, plasma	Enzymatic	UO[18]
	Barley pasta, mean of five studies			62									

Food Number and Item		Country of food production	Year of test[1]	GI[2] ± SEM (Glu = 100)	GL	Subjects (type & number)	Avail carb (Test portion)	Test portion[3] (g)	Reference food & time period	Timepoints	Sample collection	Sample analysis method[4]	Ref.
	**Fusilli pasta**												
1439	Fusilli pasta twists (Tesco Stores Ltd, UK), boiled 10 min in salted water	UK	2005	61±9	24	Normal, 10	50	68.9	Glucose, 2h	Standard	Capillary, whole blood	Glucometer[9]	49
1440	Fusilli pasta twists, wholewheat, dry pasta, boiled 10 min in unsalted water	UK	2005	55±8	22	Normal, 10	50	80.0	Glucose, 2h	Standard	Capillary, whole blood	Glucometer[9]	26
	Fusilli pasta, mean of two foods			58									
	**Gluten-free pasta**												
1441	Corn pasta, gluten-free (Orgran Natural Foods, Carrum Downs, VIC, Australia)	Australia	2007	68±7	27	Normal, 9	50	67.9	Glucose, 2h	Standard	Capillary, plasma	Enzymatic	UO[5]
1442	Corn pasta, gluten-free (Orgran Natural Foods, Australia)	Australia	2000	78±10	31	Normal, 10	50	63.8	Glucose, 2h	Standard	Capillary, plasma	Enzymatic	UO[5]
1443	Pasta, Penne, gluten free (Dr Schär AG/SPA, Italy)[8]	Italy	2015*	48±6	19	Normal, 10	50	64	Glucose, 2h	Standard	Capillary, whole blood	YSI	5
1444	Rice and Hi-Maize (high-amylose) flour spaghetti, gluten-free (Freedom Foods, Australia)	Australia	2006	51±5	20	Normal, 10	50	64.1	Glucose, 2h	Standard	Capillary, whole blood	HemoCue	UO[10]
1445	Rice and maize pasta, gluten-free, Ris'O'Mais (Orgran Foods, Australia)	Australia	2000	76±6	30	Normal, 9	50	66.5	Glucose, 2h	Standard	Capillary, plasma	Enzymatic	UO[5]
1446	Rice pasta, gluten-free (Freedom Foods, Australia)	Australia	2006	51±6	20	Normal, 10	50	NS	Glucose, 2h	Standard	Capillary, whole blood	HemoCue	UO[10]
1447	Split pea and soya pasta shells, gluten-free (Orgran Foods, Australia)	Australia	2000	29±6	12	Normal, 9	50	131.6	Glucose, 2h	Standard	Capillary, plasma	Enzymatic	UO[5]
1448	Tagliatelle pasta, fresh, gluten free (Sine srl, Italy)[8]	Italy	2015*	61±6	24	Normal, 10	50	106	Glucose, 2h	Standard	Capillary, whole blood	YSI	5
1449	Tortellini pasta, fresh, gluten free (Sine srl, Italy)[8]	Italy	2015*	60±7	24	Normal, 10	50	145	Glucose, 2h	Standard	Capillary, whole blood	YSI	5
	Gluten-free pasta, mean of nine foods			58									
1450	Spaghetti, gluten-free, rice and split pea, canned in tomato sauce	Australia	2000	68±9	27	Normal, 10	50	NS	Glucose, 2h	Standard	Capillary, plasma	Enzymatic	UO[5]
	**Instant noodles**												
1451	Instant 'two-minute' noodles, Maggi® (Nestlé, Auckland, New Zealand)	New Zealand	2000	48±8	19	Normal, 15	50	NS	Glucose, 2h	Standard	Venous, plasma	Enzymatic	27
1452	Instant 'two-minute' noodles, Maggi®, all flavors (Nestlé Australia), including noodle broth	Australia	2005	52±7	21	Normal, 9	50	357.1	Glucose, 2h	Standard	Capillary, plasma	Enzymatic	UO[5]

Food Number and Item		Country of food production	Year of test[1]	GI[2] ± SEM (Glu = 100)	GL	Subjects (type & number)	Avail carb (Test portion)	Test portion[3] (g)	Reference food & time period	Timepoints	Sample collection	Sample analysis method[4]	Ref.
1453	Instant noodles, all flavors (Woolworths Select, Australia), including noodle broth	Australia	2008	52±5	21	Normal, 10	50	416.7	Glucose, 2h	Standard	Capillary, plasma	Enzymatic	UO[5]
	Instant noodles, mean of three foods			51									
	**Penne**												
1454	Penne, durum wheat flour (Barilla, Italy)	Italy	2006	50±7	20	Normal, 10	50	71	Glucose, 2h	Standard	Capillary, whole blood	YSI	3
1455	Penne mezzane, durum wheat flour (De Cecco, Italy)	Italy	2008	47±4	19	Normal, 9	50	68	Glucose, 2h	Standard	Capillary, whole blood	YSI	3
1456	Penne pasta, white (SanRemo Penne, San Remo Macaroni Co. Pty Ltd, SA), tested by subjects with a low AMY1 copy number	Australia	2014	44±2	18	Normal, 19	50	69.4	Glucose, 2h	Standard	Capillary, plasma	Enzymatic	40
1457	Penne pasta, white (SanRemo Penne, San Remo Macaroni Co. Pty Ltd, SA), tested by subjects with a high AMY1 copy number	Australia	2014	50±2	20	Normal, 19	50	69.4	Glucose, 2h	Standard	Capillary, plasma	Enzymatic	40
1458	Pennette integrali, durum wheat wholemeal (Coop, Italy)	Italy	2008	48±9	19	Normal, 10	50	74	Glucose, 2h	Standard	Capillary, whole blood	YSI	3
1459	Pennette rigate, durum wheat flour (Barilla, Italy)	Italy	2008	53±5	21	Normal, 10	50	69	Glucose, 2h	Standard	Capillary, whole blood	YSI	3
1460	Pennette rigate integrali, durum wheat wholemeal (Barilla, Italy)	Italy	2008	73±3	29	Normal, ≥9	50	74	Glucose, 2h	Standard	Capillary, whole blood	YSI	UO[14]
1461	Penne rigate, mini (Piccolini, Barilla, Italy)	Italy	2009	59±11	24	Normal, 10	50	66	Glucose, 2h	Standard	Capillary, whole blood	YSI	3
1462	Penne rigate, mini, durum wheat flour, zucchini and spinach pulps (penne rigate con zucchine e spinaci, Piccolini, Barilla, Italy)	Italy	2011	48±5	19	Normal, 10	50	72	Glucose, 2h	Standard	Capillary, whole blood	YSI	3
	Penne pasta, mean of nine foods			52									
	**Rice noodles/pasta**												
1463	Rice noodles, dried, boiled (Thai World, Bangkok, Thailand)	Thailand	2000	61±6	24	Normal, 12	50	61.3	Glucose, 2h	Standard	Capillary, plasma	Enzymatic	74
1464	Rice noodles, freshly made, boiled (Sydney, NSW, Australia)	Australia	2000	40±4	16	Normal, 12	50	110.0	Glucose, 2h	Standard	Capillary, plasma	Enzymatic	74

Food Number and Item		Country of food production	Year of test[1]	GI[2] ± SEM (Glu = 100)	GL	Subjects (type & number)	Avail carb (Test portion)	Test portion[3] (g)	Reference food & time period	Timepoints	Sample collection	Sample analysis method[4]	Ref.
	**Spaghetti**												
	**Spaghetti, white, boiled**												
1465	Spaghetti n°5 (Barilla, Italy)	Italy	2005	33±6	13	Normal, 9	50	69	Glucose, 2h	Standard	Capillary, whole blood	YSI	3
	Spaghetti, white, boiled 15 min in salted water (Unico, 1466 Concord, ON, Canada)	Canada	1999	44±3	18	Normal, 10	50	72.2	Glucose, 2h	Standard	Capillary, plasma	Enzymatic	UO[5]
1467	Spaghetti, white (Unico, Canada)	Canada	2001	45±9	18	Normal, 21	50	NS	Glucose, 2h	Standard	Venous, NS	Enzymatic	42
1468	Spaghetti, white (Nishin Shokuhin, Japan)	Japan	2003	46±9	18	Normal, 10	50	121.0	Rice[17], 2h	Standard	Capillary, whole blood	Glucometer[9]	45
1469	Spaghetti, white (Unico, Canada)	Canada	2001	48±3	19	Normal, 47	50	NS	Glucose, 2h	Standard	Capillary, NS	YSI or Enzymatic	42
1470	Spaghetti, *Svevo 2017* durum wheat line	Italy	2019	48±4	19	Normal, 10	50	67.5	Glucose, 2h	Standard	Capillary, plasma	Enzymatic	106
	Spaghetti, white (Vetta, Greens Foods, Glendenning, 1471 Australia), boiled for 12 min	Australia	1996	49±7	20	Normal, 12	50	67.0	Bread, 2h	Standard	Capillary, plasma	Enzymatic	UO[5]
1472	Spaghetti n°12 (De Cecco, Italy)	Italy	2005	50±9	20	Normal, 10	50	88	Glucose, 2h	Standard	Capillary, whole blood	YSI	3
1473	Spaghetti classici (Voiello, Italy)	Italy	2010	51±9	20	Normal, 9	50	71	Glucose, 2h	Standard	Capillary, whole blood	YSI	3
1474	Spaghetti, *Svevo 2016* durum wheat line	Italy	2017	52±3	21	Normal, 10	50	77.9	Glucose, 2h	Standard	Capillary, plasma	Enzymatic	106
1475	Spaghetti pasta, dried (Budget, Safeway Traders Ltd, New Zealand) [13]	New Zealand	2011*	56±4	22	Normal, 30	50	NS	Glucose, 2h	Standard	Capillary, whole blood	HemoCue	69
	White spaghetti, boiled, mean of 11 foods			47									
1476	Spaghetti, *Svevo* durum wheat line SSIIa (silencing of starch synthases enzymes leading to an 28% increase in amylose and 2.8-fold increase in resistant starch)	Italy	2017	49±3	20	Normal, 10	50	87.7	Glucose, 2h	Standard	Capillary, plasma	Enzymatic	106
1477	Spaghetti, *Svevo* durum wheat line SBEIIa (silencing of starch branching enzymes leading to an 74% increase in amylose and 3.5-fold increase in resistant starch)	Italy	2019	38±3	15	Normal, 10	50	76.6	Glucose, 2h	Standard	Capillary, plasma	Enzymatic	106
	**Spaghetti, wholemeal, boiled**												
1478	President's Choice® Blue Menu™ Whole Wheat spaghetti	Canada	20042007	45±5	18	Normal, 10	50	NS	Bread, 2h	Standard	Capillary, whole blood	YSI	UO[7]

	(Loblaw Brands Limited, Canada)												
1479	Spaghetti integrali (wholemeal durum wheat flour) (Barilla, Italy)	Italy	2006	35±3	14	Normal, 9	50	73	Glucose, 2h	Standard	Capillary, whole blood	YSI	3
1480	Spaghetti integrali (wholemeal durum wheat flour) (De Cecco, Italy)	Italy	2005	55±10	22	Normal, 10	50	93	Glucose, 2h	Standard	Capillary, whole blood	YSI	3
1481	Spaghetti Integrali (wholemeal durum wheat flour) (Misura, Italy)	Italy	2005	53±10	21	Normal, 10	50	83	Glucose, 2h	Standard	Capillary, whole blood	YSI	3
1482	Wholemeal spaghetti, boiled for 8 min	Australia	2012	62±4	25	Normal, 9	50	80.0	Glucose, 2h	Standard	Capillary, plasma	Enzymatic	UO[5]
	Wholemeal spaghetti, boiled, mean of five foods			50									

Food Number and Item		Country of food production	Year of test[1]	GI[2] ± SEM (Glu = 100)	GL	Subjects (type & number)	Avail carb (Test portion)	Test portion[3] (g)	Reference food & time period	Timepoints	Sample collection	Sample analysis method[4]	Ref.
	**Pasta, other**												
1483	Buckwheat noodles	China	2005	59±1	24	Normal, 9	50	NS	Glucose, 2h	Standard	Venous, NS	NS	12
1484	Farfalle, mini, durum wheat flour, carrot and pumpkin pulps (Farfalle con carote e zucca, Piccolini, Barilla, Italy)	Italy	2011	44±5	18	Normal, 9	50	71	Glucose, 2h	Standard	Capillary, whole blood	YSI	3
1485	Fettuccine, egg (Mother Earth Fine Foods, Rowville, Australia)	Australia	2000	47±6	19	Normal, 14	50	NS	Glucose, 2h	Standard	Venous, plasma	Enzymatic	27
1486	Lower Carb Pasta (Herman Brot Pty Ltd, Australia), boiled in water for 7 min	Australia	2015	22±3	9	Normal, 10	25	160.3	Glucose, 2h	Standard	Capillary, plasma	Enzymatic	UO[5]
1487	Hokkien noodles (Fantastic Fresh, Australia)	Australia	2015	58±3	23	Normal, 10	50	135.8	Glucose, 2h	Standard	Capillary, plasma	Enzymatic	UO[5]
1488	Lasagne, egg, dry pasta, boiled in unsalted water for 10 min	UK	2005	53±9	21	Normal, 10	50	75.5	Glucose, 2h	Standard	Capillary, whole blood	Glucometer[9]	26
1489	LGI & HDF Noodles, low GI and high fiber (SCIQ Biotech Pty Ltd, Australia)	Australia	2020	39±4	16	Normal, 10	50	109.6	Glucose, 2h	Standard	Capillary, plasma	Enzymatic	UO[5]

Food Number and Item		Country of food production	Year of test[1]	GI[2] ± SEM (Glu = 100)	GL	Subjects (type & number)	Avail carb (Test portion)	Test portion[3] (g)	Reference food & time period	Timepoints	Sample collection	Sample analysis method[4]	Ref.
1490	Linguine, fresh (Hollywood Foods More than Pasta, Canada)	Canada	2011	31±4	12	Normal, 10	50	NS	Glucose, 2h	Standard	Capillary, whole blood	YSI	UO[5]
1491	Low GI Noodles (Holista Biotech Corp., USA)	USA	2017	38±4	15	Normal, 10	25	37.3	Bread, 2h	Standard	Capillary, whole blood	YSI	UO[7]
1492	Macaroni short pasta (ANCO professional, Soubry N.V., 11 Roeselare, Belgium)	Belgium	2019*	63±3	25	Normal, 13	50	176.4	Glucose, 2h	Standard	Capillary, plasma	YSI	80
1493	Mung bean noodles (Longkou beanthread), dried, boiled (Yantai cereals, China)	China	2000	39±9	16	Normal, 11	50	60.4	Glucose, 2h	Standard	Capillary, plasma	Enzymatic	74
1494	Noodles, Jianxi rice vermicelli, boiled 8 min	China	2010*	56±7	22	Normal, 10	50	63.3	Glucose, 2h	Standard	Capillary, whole blood	HemoCue	28
1495	Noodles, potato	China	2005	14±1	6	Normal, 9	50	NS	Glucose, 2h	Standard	Venous, NS	NS	12
1496	Noodles, Sau tao Beijing noodles, wheat flour and tapioca starch, boiled 3 min	China	2010*	61±5	24	Normal, 10	50	69.2	Glucose, 2h	Standard	Capillary, whole blood	HemoCue	28
1497	Noodles, Taiwan vermicelli, rice and maize starch, boiled 2 min	China	2010*	68±12	27	Normal, 10	50	54.4	Glucose, 2h	Standard	Capillary, whole blood	HemoCue	28
1498	Pasta, NS	Canada	2010*	67±8	27	Normal, 10	50	73	Bread, 2h	Standard	Capillary, whole blood	YSI	81
1499	Pipe rigate, mini, durum wheat flour, tomato and carrot pulps (Pipe rigate con pomodori e carote, Piccolini, Barilla, Italy)	Italy	2011	47±7	19	Normal, 10	50	73	Glucose, 2h	Standard	Capillary, whole blood	YSI	3
1500	Proti pasta, protein-enriched, boiled in water Vital Nature Inc, San Antonio, TX, USA)	USA	2003	28±1	11	Normal, 10	50	144.9	Glucose, 2h	Standard	Capillary, plasma	Enzymatic	UO[5]
1501	Ravioli, durum wheat flour, stuffed with calf meat, fresh (Raviolini con Carne di Vitello, Pastificio Rana SpA, Italy)	Italy	2011	58±7	23	Normal, 10	50	123	Glucose, 2h	Standard	Capillary, whole blood	YSI	3
1502	Rotini (Banza, LLC, USA)	USA	2018	44±9	18	Normal, 10	25	47.6	Glucose, 2h	Standard	Capillary, whole blood	YSI	UO[7]
1503	Semolina pasta	Canada		78±8	31	Normal, 10	50	NS	Bread, 2h	Standard	Capillary, whole blood	YSI	61
1504	Spaghetti, soy, boiled 4 min (Revival Soy®, Physicians Pharmaceuticals, Inc, USA)	USA	2004	47±7	19	Normal, 10	25	42.4	Glucose, 2h	Standard	Capillary, whole blood	HemoCue	21

Food Number and Item		Country of food production	Year of test[1]	GI[2] ± SEM (Glu = 100)	GL	Subjects (type & number)	Avail carb (Test portion)	Test portion[3] (g)	Reference food & time period	Timepoints	Sample collection	Sample analysis method[4]	Ref.
1505	Tagliatelle all'uovo (durum wheat flour, eggs, dry) (Barilla, Italy)	Italy	2005	55±4	22	Normal, 9	50	76	Glucose, 2h	Standard	Capillary, whole blood	YSI	3
1506	Tagliatelle all'uovo (durum wheat flour, eggs) (fresh pasta) (Pastificio Lo Scoiattolo, Italy)	Italy	2011	51±7	20	Normal, 10	50	70	Glucose, 2h	Standard	Capillary, whole blood	YSI	3
1507	Udon noodles, plain, reheated 5 min (Fantastic, Windsor Gardens, SA, Australia)	Australia	2001	62±8	25	Normal, 10	25	185.9	Glucose, 2h	Standard	Capillary, plasma	Enzymatic	UO[5]
1508	Vegie Pumpkin & Spinach Linguine, Latina™ Fresh, made from durum semolina, 25% pumpkin puree and 5% spinach puree, boiled in water for 2 min	Australia	2018	67±5	27	Normal, 10	50	172.4	Glucose, 2h	Standard	Capillary, plasma	Enzymatic	UO[5]
1509	Wheat noodle, dried	China	2005	46±2	18	Normal, 10	50	NS	Glucose, 2h	Standard	Venous, NS	NS	12
1510	Wheat noodle, dried, boiled for 5 min, drained (Sanlian Food, Shandong, China)	China	2016	62±8	25	Normal, 10	25	32.9	Glucose, 2h	Standard	Capillary, whole blood	Enzymatic	UO[18]

SNACK FOODS AND CONFECTIONERY

Average carbohydrate portion = 25 g, this value was used to determine the nominal GL for each item in this category.

Fruit bars & snacks

Food Number and Item		Country of food production	Year of test[1]	GI[2] ± SEM (Glu = 100)	GL	Subjects (type & number)	Avail carb (Test portion)	Test portion[3] (g)	Reference food & time period	Timepoints	Sample collection	Sample analysis method[4]	Ref.
1511	Apple Fruit Bar, fat-free, President's Choice® Blue Menu™ (Loblaw Brands Limited, Canada)	Canada	2007	90±13	23	Normal, 10	50	66.7	Bread, 2h	Standard	Capillary, whole blood	YSI	UO[7]
1512	Apple Cinnamon bar, Fruit & Yogurt Apple Cinnamon Chewy Bars (Soy), President's Choice® Blue Menu™ (Loblaw Brands Limited, Canada)	Canada	2007	34±3	9	Normal, 10	50	95.2	Bread, 2h	Standard	Capillary, whole blood	YSI	UO[7]
1513	Apricot and Almond bar	UK	2006	34±6	9	Normal, 10	50	97.3	Glucose, 2h	Standard	Capillary, whole blood	Glucometer[9]	30
1514	Apricot dried fruit snack	Australia	2001	42±3	11	Normal, 10	50	59.1	Glucose, 2h	Standard	Capillary, plasma	Enzymatic	UO[5]
1515	Apricot & apple fruit strips, gluten-free (Freedom Foods, Australia)	Australia	2006	29±6	7	Normal, 10	50	62.9	Glucose, 2h	Standard	Capillary, whole blood	HemoCue	UO[10]
1516	Apricot filled fruit bar, pureed dried apricot filling in wholemeal pastry (Mother Earth, New Zealand)	New Zealand	2000	50±8	13	Normal, 15	50	NS	Glucose, 2h	Standard	Venous, plasma	Enzymatic	27

1517	Apricot & Yoghurt Healtheries Simple Bar, wheat and gluten-free (Aussie Bodies Pty. Ltd., Australia)	Australia	2007	40±5	10	Normal, 16	50	101.8	Glucose, 2h	Standard	Capillary, whole blood	HemoCue	UO[10]
1518	Banana chips, dried banana pieces	Australia	2009	44±5	11	Normal, 10	50	75.8	Glucose, 2h	Standard	Capillary, plasma	Enzymatic	UO[5]
1519	Banana Fruit Fingers, Heinz Kidz™ (HJ Heinz, Australia)	Australia	2000	61±11	15	Normal, 10	50	76.9	Glucose, 2h	Standard	Capillary, plasma	Enzymatic	UO[5]
1520	Banana, processed fruit fingers, Heinz Kidz™ (H J Heinz, Malvern, Australia)	Australia	2000	61±11	15	Normal, 10	50	76.9	Glucose, 2h	Standard	Capillary, plasma	Enzymatic	UO[5]
1521	Berry & Yoghurt Healtheries Simple Bar, wheat and gluten free (Aussie Bodies Pty. Ltd., Australia)	Australia	2007	51±4	13	Normal, 15	50	95.8	Glucose, 2h	Standard	Capillary, whole blood	HemoCue	UO[10]
1522	Berry bar, Berrylicious Trim Bar (Aussie Bodies Pty. Ltd., Australia)	Australia	2007	46±4	12	Normal, 10	50	146.2	Glucose, 2h	Standard	Capillary, whole blood	HemoCue	UO[10]
1523	Blackberry Sour Buzz dried fruit leather, Sunripe School Straps (Windsor Farm Foods, Australia)	Australia	2002	35±5	9	Normal, 10	50	67.0	Glucose, 2h	Standard	Capillary, plasma	Enzymatic	UO[5]
1524	Citrus Lemon Protein Bar (Herbalife of Japan K.K.)	Japan	2018	31±4	8	Normal, 10	50	109.4	Glucose, 2h	Standard	Capillary, plasma	Enzymatic	UO[5]
1525	Cranberry, Blueberry bar, Fruit & Yogurt Cranberry Blueberry Bars (Soy), President's Choice® Blue Menu™ (Loblaw Brands Limited, Canada)	Canada	2007	33±2	8	Normal, 10	50	95.2	Bread, 2h	Standard	Capillary, whole blood	YSI	UO[7]
1526	Fig Fruit bar, President's Choice® Blue Menu™ (Loblaw Brands Limited, Canada)	Canada	2007	70±8	18	Normal, 10	50	66.7	Bread, 2h	Standard	Capillary, whole blood	YSI	UO[7]
1527	Fruit and nut mix (Finest, UK)	UK	2005	15±3	4	Normal, 10	50	106.2	Glucose, 2h	Standard	Capillary, whole blood	Glucometer[9]	26
1528	Fruit snack processed, Roll-Ups® (Uncle Toby's, Australia)	Australia	1998	99±12	25	Normal, 10	50	60.8	Bread, 2h	Standard	Capillary, plasma	Enzymatic	UO[5]
1529	Ginger, candied, sucrose-free (Buderim Ginger, Australia)	Australia	2006	10±4	3	Normal, 10	50	64.9	Glucose, 2h	Standard	Capillary, whole blood	HemoCue	UO[5]
1530	Peach & pear fruit strips, gluten-free (Freedom Foods, Australia)	Australia	2006	29±6	7	Normal, 10	50	62.9	Glucose, 2h	Standard	Capillary, whole blood	HemoCue	UO[10]
1531	Raspberry Fruit bar, fat-free, President's Choice® Blue Menu™ (Loblaw Brands Limited, Canada)	Canada	2007	74±11	19	Normal, 10	50	64.5	Bread, 2h	Standard	Capillary, whole blood	YSI	UO[7]

Food Number and Item		Country of food production	Year of test[1]	GI[2] ± SEM (Glu = 100)	GL	Subjects (type & number)	Avail carb (Test portion)	Test portion[3] (g)	Reference food & time period	Timepoints	Sample collection	Sample analysis method[4]	Ref.
	Strawberry processed fruit bar, Real Fruit (Uncle Toby's, 1532 Australia)	Australia	1998	90±12	23	Normal, 10	50	60.5	Glucose, 2h	Standard	Capillary, plasma	Enzymatic	UO[5]
	Strawberry Fruit Bar, Kettle Valley (SunOpta, Inc., 1533 Brampton, ON, Canada)	Canada	2009*	32±2	8	Normal, 40	50	69	Glucose, 2h	Standard	Capillary, whole blood	YSI	38
	Strawberry Fruit Bar, Kettle Valley (SunOpta, Inc., 1534 Brampton, ON, Canada)	Canada	2009*	34±2	9	Normal, 37	50	69	Glucose, 2h	Standard	Capillary, whole blood	YSI	38
	Strawberry Fruit Bar, Kettle Valley (SunOpta, Inc., 1535 Brampton, ON, Canada)	Canada	2009*	35±2	9	Normal, Caucasian, 40	50	69	Glucose, 2h	Standard	Capillary, whole blood	YSI	38
	Strawberry Fruit Bar, Kettle Valley (SunOpta, Inc., 1536 Brampton, ON, Canada)	Canada	2009*	31±2	8	Normal, Non-Caucasian, 37	50	69	Glucose, 2h	Standard	Capillary, whole blood	YSI	38
	Strawberry Fruit Bar, Kettle Valley (SunOpta, Inc., 1537 Brampton, ON, Canada)	Canada	2009*	35±2	9	Normal, ≤40 y, 39	50	69	Glucose, 2h	Standard	Capillary, whole blood	YSI	38
	Strawberry Fruit Bar, Kettle Valley (SunOpta, Inc., 1538 Brampton, ON, Canada)	Canada	2009*	31±2	8	Normal, >40 y, 38	50	69	Glucose, 2h	Standard	Capillary, whole blood	YSI	38
	Strawberry Fruit Bar, Kettle Valley (SunOpta, Inc., 1539 Brampton, ON, Canada)	Canada	2009*	36±2	9	Normal, lean, 37	50	69	Glucose, 2h	Standard	Capillary, whole blood	YSI	38
	Strawberry Fruit Bar, Kettle Valley (SunOpta, Inc., 1540 Brampton, ON, Canada)	Canada	2009*	30±2	8	Normal, overweight, 40	50	69	Glucose, 2h	Standard	Capillary, whole blood	YSI	38
	Strawberry Fruit Bar, Kettle Valley *mean of eight studies*			33									
1541	Strawberry fruit leather (Stretch Island Fruit Company™, WA, USA)	USA	2006	29±4	7	Normal, 12	50	63.6	Glucose, 2h	Standard	Capillary, plasma	Enzymatic	UO[5]
	Sweet strawberry fruit leather (Stretch Island Fruit 1542 Company™, WA, USA), tested with water	USA	2009*	25±5	6	Normal, 10	50	64	Bread, 2h	Standard	Capillary, whole blood	YSI	107
	Sweet strawberry fruit leather (Stretch Island Fruit 1543 Company™, WA, USA), tested with coffee or tea	USA	2009*	22±3	6	Normal, 10	50	64	Bread, 2h	Standard	Capillary, whole blood	YSI	107

Food Number	and Item	Country of food production	Year of test[1]	GI[2] ± SEM (Glu = 100)	GL	Subjects (type & number)	Avail carb (Test portion)	Test portion[3] (g)	Reference food & time period	Timepoints	Sample collection	Sample analysis method[4]	Ref.
1544	Sweet strawberry fruit leather (Stretch Island Fruit Co, Allyn, WA, USA)	USA	2008*	33±7	8	Normal, 290	50	63.6	Glucose, 2h	Standard	Various	Various	108
	Sweet strawberry fruit leather, mean of three studies			27									
1545	Strawberry & wildberry dried fruit leather, Sunripe School Straps (Windsor Farm Foods, Australia)	Australia	2002	40±5	10	Normal, 10	50	67.0	Glucose, 2h	Standard	Capillary, plasma	Enzymatic	UO[5]
1546	Tropical dried fruit snack	Australia	2001	41±3	10	Normal, 10	50	59.0	Glucose, 2h	Standard	Capillary, plasma	Enzymatic	UO[5]
1547	Tropical fruit and nut mix (Finest, UK)	UK	2005	49±7	12	Normal, 9	50	89.8	Glucose, 2h	Standard	Capillary, whole blood	Glucometer[9]	26
1548	Wild berry dried fruit snack	Australia	2001	35±4	9	Normal, 10	50	58.3	Glucose, 2h	Standard	Capillary, plasma	Enzymatic	UO[5]
	**Savory snack foods**												
1549	Chick Pea chips (Freedom Foods, Australia)	Australia	2006	44±7	11	Normal, 9	50	135.5	Glucose, 2h	Standard	Capillary, whole blood	HemoCue	UO[10]
	**Cheese-flavored snacks**												
1550	Combos Snacks Cheddar Cheese Crackers (M&M/Mars, USA)	USA	2003	54±6	14	Normal, 10	50	79.0	Glucose, 2h	Standard	Capillary, plasma	Enzymatic	UO[5]
1551	Combos Snacks Cheddar Cheese Pretzels (M&M/Mars, USA)	USA	2003	52±6	13	Normal, 10	50	70.5	Glucose, 2h	Standard	Capillary, plasma	Enzymatic	UO[5]
1552	Cheese Puffs, made from rice and corn puffs, aged white cheddar extruded snack, Pirate's Booty brand (Robert's American Gourmet, Sea Cliff, NY, USA)	USA	2006	70±5	18	Normal, 12	50	82.4	Glucose, 2h	Standard	Capillary, whole blood	HemoCue	UO[5]
1553	Cheese Puffs, Pirates Booty brand (Robert's American Gourmet, Sea Cliff, NY, USA)	USA	2008*	74±11	19	Normal, 290	50	82.4	Glucose, 2h	Standard	Various	Various	108
1554	Cheese Puffs, Pirates Booty brand (Robert's American Gourmet, Sea Cliff, NY, USA), tested with water	USA	2009*	53±6	13	Normal, 10	50	84	Bread, 2h	Standard	Capillary, whole blood	YSI	107
1555	Cheese Puffs, Pirates Booty brand (Robert's American Gourmet, Sea Cliff, NY, USA), tested with coffee or tea	USA	2009*	53±6	13	Normal, 10	50	84	Bread, 2h	Standard	Capillary, whole blood	YSI	107

Food Number and Item		Country of food production	Year of test[1]	GI[2] ± SEM (Glu = 100)	GL	Subjects (type & number)	Avail carb (Test portion)	Test portion[3] (g)	Reference food & time period	Timepoints	Sample collection	Sample analysis method[4]	Ref.
	Cheese Puffs, mean of four studies			63									
1556	Rice and corn cheese flavored extruded snack, Twisties™ (Smith's Snackfood Co., Australia)	Australia	1999	74±5	19	Normal, 10	50	87.6	Glucose, 2h	Standard	Capillary, plasma	Enzymatic	UO[5]
	**Chips or Crisps, made from cereal grains**												
1557	Brown rice chips with wild rice, gluten-free, SunRice™	Australia	2018	70±6	18	Normal, 10	50	75.9	Glucose, 2h	Standard	Capillary, plasma	Enzymatic	UO[5]
1558	Corn chips, plain, salted (Doritos™ original, Smith's Snack Food Co., Australia)	Australia	1998	42±4	11	Normal, 10	50	NS	Bread, 2h	Standard	Capillary, plasma	Enzymatic	UO[5]
1559	Grain Waves Original flavored wholegrain chips, Smith's™	Australia	2010	51±5	13	Normal, 10	50	79.0	Glucose, 2h	Standard	Capillary, plasma	Enzymatic	UO[5]
1560	Finger millet extruded snack	India	2018*	65±7	16	Normal, 12	25	39.6	Glucose, 2h	Standard	Capillary, whole blood	HemoCue	109
1561	Flaxseed Tortilla chips, Sea Salt, President's Choice® Blue Menu™ (Loblaw Brands Limited, Canada)	Canada	2007	45±6	11	Normal, 10	50	125.0	Bread, 2h	Standard	Capillary, whole blood	YSI	UO[7]
	Flaxseed Tortilla chips, Spicy, President's Choice® Blue 1562 Menu™ (Loblaw Brands Limited, Canada)	Canada	2007	34±5	9	Normal, 10	50	125.0	Bread, 2h	Standard	Capillary, whole blood	YSI	UO[7]
1563	Japanese Tamari Rice & Corn Crisps, President's Choice® Blue Menu™ (Loblaw Brands Limited, Canada)	Canada	2004-2007	91±6	23	Normal, 10	50	NS	Bread, 2h	Standard	Capillary, whole blood	YSI	UO[7]
1564	Japanese Wasabi & Honey Rice & Corn Crisps, President's ® Blue Menu™ (Loblaw Brands Limited, Canada) Choice	Canada	2004-2007	82±7	21	Normal, 10	50	NS	Bread, 2h	Standard	Capillary, whole blood	YSI	UO[7]
1565	Prawn crackers, BBQ Rib flavor, Infuzions™, made from tapioca and wheat (Majans Pty Ltd, QLD, Australia)	Australia	2017	74±5	19	Normal, 10	25	36.4	Glucose, 2h	Standard	Capillary, plasma	Enzymatic	UO[5]
1566	Pretzels, oven-baked, traditional wheat flavor, 97% fat free (Parker's, Smith's Snack Food Co., Australia)	Australia	2005	84±6	21	Normal, 10	50	62.5	Glucose, 2h	Standard	Capillary, plasma	Enzymatic	UO[5]
1567	Thai Curry Rice & Corn Crisps, President's Choice® Blue	Canada	2004-2007	84±6	21	Normal, 10	50	NS	Bread, 2h	Standard	Capillary, whole blood	YSI	UO[7]

	Menu™ (Loblaw Brands Limited, Canada)												
	**Popcorn**												
1568	Poppin Microwave Popcorn, butter flavor Green's Foods, Australia)	Australia	2013	51±6	13	Normal, 10	25	46.5	Glucose, 2h	Standard	Capillary, plasma	Enzymatic	UO[5]
1569	Microwave Popping Corn, natural flavor, President's Choice® Blue Menu™ (Loblaw Brands Limited, Canada)	Canada	2004-2007	58±8	15	Normal, 10	50	NS	Bread, 2h	Standard	Capillary, whole blood	YSI	UO[7]
1570	Poppin Microwave Popcorn, butter flavor Green's Foods, Australia)	Australia	2007	62±8	16	Normal, 9	25	49.6	Glucose, 2h	Standard	Capillary, whole blood	HemoCue	UO[5]
1571	Poppin Lite Microwave Popcorn, butter flavor, 50% reduced fat (Green's Foods, Australia)	Australia	2007	67±8	17	Normal, 9	25	44.7	Glucose, 2h	Standard	Capillary, whole blood	HemoCue	UO[5]
1572	Microwave Popping Corn, butter flavor, President's Choice® Blue Menu™ (Loblaw Brands Limited, Canada)	Canada	2004-2007	72±8	18	Normal, 10	50	NS	Bread, 2h	Standard	Capillary, whole blood	YSI	UO[7]
	Popcorn, mean of five studies			62									
	**Potato crisps**												
1573	Potato chips, crinkle cut, Smith's™ Original	Australia	2012	48±5	12	Normal, 10	50	113.4	Glucose, 2h	Standard	Capillary, plasma	Enzymatic	UO[5]
1574	Potato chips, Pringles™ Original	Australia	2012	57±5	14	Normal, 10	50	96.2	Glucose, 2h	Standard	Capillary, plasma	Enzymatic	UO[5]
1575	Potato crisps	China	2005	60±2	15	Normal, 10	50	NS	Glucose, 2h	Standard	Venous, NS	NS	12
1576	Baked potato chips (Baked Lays, Original Potato Crisps)	Canada	2009	72±6	18	Normal, 10	50	66	Bread, 2h	Standard	Capillary, whole blood	YSI	110
1577	Baked potato chips (Baked Lays, Original Potato Crisps)	Canada	2009	71±8	18	Normal, 10	50	66	Bread, 2h	Standard	Capillary, whole blood	YSI	110
	Potato crisps, mean of five studies			56									
1578	Sweet potato crisps, Red Rock Deli™ (The Red Rock Deli Chip Company, VIC, Australia)	Australia	2016	41±4	10	Normal, 10	25	60.7	Glucose, 2h	Standard	Capillary, plasma	Enzymatic	UO[5]

Food Number and Item		Country of food production	Year of test[1]	GI[2] ± SEM (Glu = 100)	GL	Subjects (type & number)	Avail carb (Test portion)	Test portion[3] (g)	Reference food & time period	Timepoints	Sample collection	Sample analysis method[4]	Ref.
	**Soy snacks**												
1579	Soy based snack product, SoyCarat Cheese flavor (Otsuka Pharmaceutical Co, Ltd, Japan)	Japan	2014	44±4	11	Normal, 10	50	125.5	Glucose, 2h	Standard	Capillary, plasma	Enzymatic	UO[5]
1580	Soy based snack product, SoyCarat Olive Oil & Garlic flavor (Otsuka Pharmaceutical Co, Ltd, Japan)	Japan	2014	41±4	10	Normal, 10	50	109.4	Glucose, 2h	Standard	Capillary, plasma	Enzymatic	UO[5]
	Soy based snack product, SoyCarat Nori & Natto flavor 1581 (Otsuka Pharmaceutical Co, Ltd, Japan)	Japan	2014	37±5	9	Normal, 10	50	107.6	Glucose, 2h	Standard	Capillary, plasma	Enzymatic	UO[5]
1582	Sunshine™ soy protein chips, lightly salted (Revival Soy®, Physicians Pharmaceuticals, Inc., USA)	USA	2004	87±8	22	Normal, 10	25	48.1	Glucose, 2h	Standard	Capillary, whole blood	HemoCue	21
	**Other savory snacks**												
1583	Puffed rice squares	Czech Republic	2005	77±5	19	Normal, 20	50	60.3	Glucose, 2h	0,15,30,45, 60,75,90, 105,120	Capillary, whole blood	Glucometer[9]	84
1584	Wasabi peas	Australia	2018	41±4	10	Normal, 10	25	44.0	Glucose, 2h	Standard	Capillary, plasma	Enzymatic	UO[5]
	**Sweet snack foods**												
1585	Almond Chocolate (Meiji Co. Ltd, Japan)	Japan	2019	24±2	6	Normal, 10	50	115.7	Glucose, 2h	Standard	Capillary, plasma	Enzymatic	UO[5]
1586	Chocolate crinkles, containing wheat flour and coconut flour	Philippines	2002	44±4	11	Normal, 10	50	NS	Bread, 2h	Standard	Capillary, serum	Enzymatic	2
1587	Chocolate, dark, Dove® (M&M/Mars, USA)	USA	2005	23±3	6	Normal, 10	50	95.0	Glucose, 2h	Standard	Capillary, plasma	Enzymatic	UO[5]
	Chocolate, dark, 74% Cocoa (Alpes d'Or, Hangzhou 1588 Qiaokemei, China)	China	2019	44±6	11	Normal, 12	25	83.3	Glucose, 2h	Standard	Capillary, plasma	Enzymatic	UO[18]
1589	Chocolate Kouka Cacao 86% (Meiji Co. Ltd, Japan)	Japan	2018	18±2	5	Normal, 10	50	103.3	Glucose, 2h	Standard	Capillary, plasma	Enzymatic	UO[5]
1590	Chocolate Kouka Cacao 72% (Meiji Co. Ltd, Japan)	Japan	2018	29±4	7	Normal, 10	50	71.4	Glucose, 2h	Standard	Capillary, plasma	Enzymatic	UO[5]
	Chocolate dark, mean of four foods			29									

Food Number and Item		Country of food production	Year of test[1]	GI[2] ± SEM (Glu = 100)	GL	Subjects (type & number)	Avail carb (Test portion)	Test portion[3] (g)	Reference food & time period	Timepoints	Sample collection	Sample analysis method[4]	Ref.
1591	Chocolate, dark with raisins, peanuts and jam	Czech Republic	2005	44±5	11	Normal, 20	50	91.9	Glucose, 2h	0,15,30,45, 60,75,90, 105,120	Capillary, whole blood	Glucometer[9]	84
1592	Chocolate, milk, Meiji brand (Meiji Co. Ltd, Japan)	Japan	2018	39±3	10	Normal, 10	50	47.9	Glucose, 2h	Standard	Capillary, plasma	Enzymatic	UO[5]
1593	Chocolate, milk, Dove® (Mars Confectionery, Ballarat, Australia)	Australia	1997	45±8	11	Normal, 10-12	50	NS	Bread, 2h	Standard	Capillary, plasma	Enzymatic	25
1594	Chocolate, milk (Milka, Mondelez, Italy)	Italy	2012	54±5	14	Normal, 10	25	43	Glucose, 2h	Standard	Capillary, whole blood	YSI	3
1595	Chocolate, milk (Nestlé, Sydney, NSW, Australia)	Australia	2000	42±8	11	Normal, 10	50	NS	Glucose, 2h	Standard	Capillary, plasma	Enzymatic	UO[5]
	Chocolate, milk, plain, mean of four studies			45									
1596	Milk chocolate egg (Ovetto cioccolato, Dolci Preziosi, Italy)	Italy	2012	48±9	12	Normal, 10	25	43	Glucose, 2h	Standard	Capillary, whole blood	YSI	3
1597	Chocolate, NS	China	2005	49±3	12	Normal, 10	50	NS	Glucose, 2h	Standard	Venous, NS	NS	12
1598	Chocolate, white, Lindt™ Lindor	Switzerland	2012	34±4	9	Normal, 10	25	58.0	Glucose, 2h	Standard	Capillary, plasma	Enzymatic	UO[5]
1599	Chocolate, white, Milky Bar® (Nestlé, Australia)	Australia	2012	41±6	10	Normal, 10	50	90.6	Glucose, 2h	Standard	Capillary, plasma	Enzymatic	UO[5]
1600	Chocolate, white, Milky Bar® (Nestlé, Australia)	Australia	2000	44±6	11	Normal, 10	50	85.9	Glucose, 2h	Standard	Capillary, plasma	Enzymatic	UO[5]
1601	White chocolate bar (Galak, Nestlè, Italy)	Italy	2012	44±5	11	Normal, 10	25	41	Glucose, 2h	Standard	Capillary, whole blood	YSI	3
	Chocolate, white, mean of four studies			41									
1602	Chocolate candy, sugar free, Dove® (M&M/Mars, USA)	USA	2005	23±3	6	Normal, 10	25	83.3	Glucose, 2h	Standard	Capillary, plasma	Enzymatic	UO[5]
	Cocoavia™ high flavanol chocolate covered almonds 1603 (M&M/Mars, USA)	USA	2005	21±3	5	Normal, 10	25	144.4	Glucose, 2h	Standard	Capillary, plasma	Enzymatic	UO[5]
1604	Chocolate coated wafer, with whole hazelnut and chocolate cream filling, Duplo nocciolato (Ferrero, Italy)	Italy	2012	59±6	15	Normal, 10	25	54	Glucose, 2h	Standard	Capillary, whole blood	YSI	3

1605	Chocolate coated chocolate soaked cookie, Galbo Chocolate (Meiji Co. Ltd, Japan)	Japan	2019	37±4	9	Normal, 10	50	97.7	Glucose, 2h	Standard	Capillary, plasma	Enzymatic	UO[5]
1606	Jelly, made from commercial jelly crystals, Raspberry Razzle flavor (Aeroplane Jelly Company, West Ryde, Australia), prepared with water	Australia	2005	53±6	13	Normal, 10	50	342.5	Glucose, 2h	Standard	Capillary, plasma	Enzymatic	UO[5]
1607	Jelly beans, assorted colors (Savings, Grocery Holdings, Tooronga, Australia)	Australia	1998	76±6	19	Normal, 12	50	NS	Bread, 2h	Standard	Capillary, plasma	Enzymatic	UO[5]
1608	Kinder Joy/ Merendero (Hazelnut and vanilla cream, with wafer balls coated with chocolate and hazelnut crumbs) (Kinder, Ferrero, Italy)	Italy	2012	42±5	11	Normal, 10	25	43	Glucose, 2h	Standard	Capillary, whole blood	YSI	3
1609	Kinder Softy nocciola (Praline with hazelnut cream) (Kinder, Ferrero, Italy)	Italy	2012	43±7	11	Normal, 10	25	51	Glucose, 2h	Standard	Capillary, whole blood	YSI	3

Food Number and Item		Country of food production	Year of test[1]	GI[2] ± SEM (Glu = 100)	GL	Subjects (type & number)	Avail carb (Test portion)	Test portion[3] (g)	Reference food & time period	Timepoints	Sample collection	Sample analysis method[4]	Ref.
1610	Licorice pieces	Australia	2012	69±8	17	Normal, 10	50	68.0	Glucose, 2h	Standard	Capillary, plasma	Enzymatic	UO[5]
1611	Licorice, soft (Coles Supermarkets, Australia)	Australia	2003	78±11	20	Normal, 10	50	70.9	Glucose, 2h	Standard	Capillary, whole blood	HemoCue	UO[5]
1612	Macadamia Chocolate (Meiji Co. Ltd, Japan)	Japan	30	24±2	6	Normal, 10	50	113.6	Glucose, 2h	Standard	Capillary, plasma	Enzymatic	UO[5]
1613	M&M's®, peanut (Mars Confectionery, Australia)	Australia	1997	33±3	8	Normal, 10-12	50	NS	Bread, 2h	Standard	Capillary, plasma	Enzymatic	25
1614	Mars Bar® (Mars Confectionery, Australia)	Australia	1997	62±8	16	Normal, 10-12	50	NS	Bread, 2h	Standard	Capillary, plasma	Enzymatic	25
1615	Marshmallows (Candy Lane, Australia)	Australia	2003	62±6	16	Normal, 10	50	62.3	Glucose, 2h	Standard	Capillary, plasma	Enzymatic	UO[5]
1616	Marshmallows (Woolworths Homebrand, Australia)	Australia	2012	67±4	17	Normal, 10	50	59.1	Glucose, 2h	Standard	Capillary, plasma	Enzymatic	UO[5]
1617	Nutella®, chocolate hazelnut spread (Ferrero Australia, Milson's Point, NSW, Australia)	Australia	1997	33±4	8	Normal, 10-12	50	NS	Bread, 2h	Standard	Capillary, plasma	Enzymatic	25

	Nutella®, chocolate hazelnut spread (Ferrero Australia, 1618 Australia)	Australia	2003	30±4	8	Normal, 10	50	NS	Glucose, 2h	Standard	Capillary, whole blood	HemoCue	UO[5]
	Nutella®, chocolate hazelnut spread (Ferrero Australia, 1619 Australia)	Australia	2007	25±4	6	Normal, 10	50	91.4	Glucose, 2h	Standard	Capillary, whole blood	HemoCue	UO[5]
1620	Nutella® (Ferrero, Italy)	Italy	2012	45±4	11	Normal, 10	25	44	Glucose, 2h	Standard	Capillary, whole blood	YSI	3
	Nutella®, mean of four studies			33									
1621	Nutella & Go (Hazelnut chocolate spread with breadsticks) (Ferrero, Italy)	Italy	2012	57±9	14	Normal, 10	25	41	Glucose, 2h	Standard	Capillary, whole blood	YSI	3
1622	Nutella B-ready (Wafer bar, hazelnut cream-filled) (Ferrero, Italy)	Italy	2012	55±7	14	Normal, 10	25	43	Glucose, 2h	Standard	Capillary, whole blood	YSI	3
1623	Pocket Coffee (Praline, with coffee cream) (Ferrero, Italy)	Italy	2014	58±9	15	Normal, 10	25	43	Glucose, 2h	Standard	Capillary, whole blood	YSI	3
1624	Pop Tarts™, double choc (Kellogg's, Australia)	Australia	2000	70±2	18	Normal, 10	50	74.4	Glucose, 2h	Standard	Capillary, whole blood	HemoCue	UO[5]
1625	Raffaello (Ferrero, Italy)	Italy	2014	52±7	13	Normal, 10	25	66	Glucose, 2h	Standard	Capillary, whole blood	YSI	3
1626	Rocher (Ferrero, Italy)	Italy	2014	53±6	13	Normal, 10	25	56	Glucose, 2h	Standard	Capillary, whole blood	YSI	3

Food Number and Item		Country of food production	Year of test[1]	GI[2] ± SEM (Glu = 100)	GL	Subjects (type & number)	Avail carb (Test portion)	Test portion[3] (g)	Reference food & time period	Timepoints	Sample collection	Sample analysis method[4]	Ref.
1627	Rondnoir (Ferrero, Italy)	Italy	2014	53±7	13	Normal, 10	25	48	Glucose, 2h	Standard	Capillary, whole blood	YSI	3
1628	Rum balls, containing dark chocolate and coconut	Australia	2009	50±6	13	Normal, 10	50	89.3	Glucose, 2h	Standard	Capillary, plasma	Enzymatic	UO[5]
1629	Skittles® (Mars Confectionery, Australia)	Australia	1997	70±5	18	Normal, 10-12	50	NS	Bread, 2h	Standard	Capillary, plasma	Enzymatic	25
1630	Slush, lemon (Granigel la granita classica siciliana, Dolfin, Italy)	Italy	2009	66±8	17	Normal, 10	25	149	Glucose, 2h	Standard	Capillary, whole blood	YSI	3

Food Number and Item		Country of food production	Year of test[1]	GI[2] ± SEM (Glu = 100)	GL	Subjects (type & number)	Avail carb (Test portion)	Test portion[3] (g)	Reference food & time period	Timepoints	Sample collection	Sample analysis method[4]	Ref.
1631	Sorbet, caffè/cappuccino (GranSoleil Gran dessert Caffè/Cappuccino, Ferrero, Italy)	Italy	2009	62±5	16	Normal, 9	25	91	Glucose, 2h	Standard	Capillary, whole blood	YSI	3
	Sorbet, lemon (GranSoleil Gran dessert Limone, Ferrero, 1632 Italy)	Italy	2009	58±7	15	Normal, 9	25	86	Glucose, 2h	Standard	Capillary, whole blood	YSI	3
1633	Sorbet, lemon (Sorbetto al limone, Carte d'or, Algida, Italy)	Italy	2009	72±6	18	Normal, 9	25	96	Glucose, 2h	Standard	Capillary, whole blood	YSI	3
1634	Sorbet, vanilla (GranSoleil Gran dessert Vaniglia, Ferrero, Italy)	Italy	2009	57±8	14	Normal, 9	25	107	Glucose, 2h	Standard	Capillary, whole blood	YSI	3
1635	Sour Straws, strawberry flavor, gummy/chewy confectionery	Australia	2017	76±7	19	Normal, 10	25	31.3	Glucose, 2h	Standard	Capillary, plasma	Enzymatic	UO[5]
1636	Yummiees lollies (Allseps Pty. Ltd., Australia)	Australia	2006	43±3	11	Normal, 14	50	71.1	Glucose, 2h	Standard	Capillary, whole blood	HemoCue	UO[10]
	**Snack bars**												
1637	Cold-pressed bar, made with 22.1 g maltodextrin (Globe Plus 10 DE maltodextrin) and 21.8 g corn syrup (Globe Plus 63 DE Maltodextrin)	USA	2017*	93±8	23	Normal, 14	50	70	Glucose, 2h	Standard	Capillary, whole blood	YSI	111
	Cold-pressed bar, made with 24.9 g SDS (SUSTRA™ 2434 slowly digestible carbohydrate) (Ingredion Incorporated, 1638 Bridgewater, NJ) and 18.9 g corn syrup (Globe Plus 63 DE Maltodextrin)	USA	2017*	50±5	13	Normal, 14	50	72	Glucose, 2h	Standard	Capillary, whole blood	YSI	111
1639	Chewy Chocolate Chip & Marshmallow Granola bar, President's Choice® Blue Menu™ (Loblaw Brands Limited, Canada)	Canada	2007	78±6	20	Normal, 10	50	65.0	Bread, 2h	Standard	Capillary, whole blood	YSI	UO[7]
1640	Chewy Cranberry Apple Granola Bar, President's Choice® Blue Menu™(Loblaw Brands Limited, Canada)	Canada	2007	58±5	15	Normal, 10	50	65.0	Bread, 2h	Standard	Capillary, whole blood	YSI	UO[7]
	Chocolate Healtheries Simple bar, wheat and gluten free 1641 (Aussie Bodies Pty. Ltd., Australia)	Australia	2007	35±3	9	Normal, 15	50	91.7	Glucose, 2h	Standard	Capillary, whole blood	HemoCue	UO[10]
	Cocoavia™ high flavanol chocolate almond Snack bar 1642 (M&M/Mars, USA)	USA	2005	63±7	16	Normal, 10	50	91.7	Glucose, 2h	Standard	Capillary, plasma	Enzymatic	UO[5]

1643	Cocoavia™ Crispy Chocolate bar (M&M/Mars, USA)	USA	2005	33±5	8	Normal, 10	50	100.0	Glucose, 2h	Standard	Capillary, plasma	Enzymatic	UO[5]
1644	ExtendBar™ Apple Cinnamon Delight bar (ExtendBar, USA)	USA	2004	33±7	8	Normal, 10	25	83.3	Glucose, 2h	Standard	Capillary, plasma	Enzymatic	UO[5]
1645	ExtendBar™ Chocolate Delight bar (ExtendBar, USA)	USA	2004	41±6	10	Normal, 10	25	62.5	Glucose, 2h	Standard	Capillary, plasma	Enzymatic	UO[5]
1646	ExtendBar™ Peanut Delight bar (ExtendBar, USA)	USA	2004	32±5	8	Normal, 10	25	62.5	Glucose, 2h	Standard	Capillary, plasma	Enzymatic	UO[5]
1647	Fruit and Nuts Superfoods bar (SoLo GI Nutrition, Inc, Canada)	Canada	2018	40±6	10	Normal, 10	25	71.4	Glucose, 2h	Standard	Capillary, whole blood	YSI	UO[7]
1648	Granola bar, containing wheat flour and coconut flour	Philippines	2002	46±3	12	Normal, 10	50	NS	Bread, 2h	Standard	Capillary, serum	Enzymatic	2
1649	Griffin's Fruitli bar, apricot (Griffins, Auckland, New Zealand)[13]	New Zealand	2010*	54±5	14	Normal, 20	50	85	Glucose, 2h	Standard	Capillary, whole blood	HemoCue	112
1650	Kinder Bueno white (Wafer bar, milk and hazelnut spread-filled, coated white chocolate) (Kinder, Ferrero, Italy)	Italy	2012	47±5	12	Normal, 10	25	48	Glucose, 2h	Standard	Capillary, whole blood	YSI	3
1651	Kinder Choco fresh (Chocolate bar, milk cream-filled, fresh) (Kinder, Ferrero, Italy)	Italy	2012	55±5	14	Normal, 10	25	62	Glucose, 2h	Standard	Capillary, whole blood	YSI	3
1652	Kinder Happy Hippo (Wafer bar, milk and hazelnut spread-filled, partially coated with white chocolate) (Kinder, Ferrero)	Italy	2014	47±4	12	Normal, 10	25	50	Glucose, 2h	Standard	Capillary, whole blood	YSI	3
1653	Kinder Maxi king (Milk cream bar, sponge cake-filled, coated with chocolate and hazelnut crumbs, fresh) (Kinder, Ferrero, Italy)	Italy	2012	58±7	15	Normal, 10	25	65	Glucose, 2h	Standard	Capillary, whole blood	YSI	3
1654	Kinder Pinguì cacao (Milk cream bar, sponge cake-filled, coated with chocolate, fresh) (Kinder, Ferrero, Italy)	Italy	2012	51±5	13	Normal, 10	25	64	Glucose, 2h	Standard	Capillary, whole blood	YSI	3
1655	Kinder Pinguì cocco (Milk cream bar, sponge cake-filled, coated with chocolate and coconut, fresh) (Kinder, Ferrero, Italy)	Italy	2012	53±6	13	Normal, 10	25	65	Glucose, 2h	Standard	Capillary, whole blood	YSI	3
1656	KitKat (Nestlè, Italy)	Italy	2012	50±7	13	Normal, 10	25	40	Glucose, 2h	Standard	Capillary, whole blood	YSI	3
1657	Kudos Milk Chocolate Granola bar with M&M's Milk Chocolate Mini Baking Bits (M&M/Mars, USA)	USA	2003	52±4	13	Normal, 10	50	69.0	Glucose, 2h	Standard	Capillary, plasma	Enzymatic	UO[5]

Food Number and Item		Country of food production	Year of test[1]	GI[2] ± SEM (Glu = 100)	GL	Subjects (type & number)	Avail carb (Test portion)	Test portion[3] (g)	Reference food & time period	Timepoints	Sample collection	Sample analysis method[4]	Ref.
	Kudos Whole-Grain bars, chocolate chip (M&M/Mars, 1658 Hackettstown, USA)	USA	1997	62±8	16	Normal, 10-12	50	74.7	Bread, 2h	Standard	Capillary, plasma	Enzymatic	25
1659	Kudos Milk Chocolate Granola bars, Peanut Butter flavor (M&M/Mars, USA)	USA	2003	45±6	11	Normal, 10	50	83.3	Glucose, 2h	Standard	Capillary, plasma	Enzymatic	UO[5]
1660	Milky Way® bar (M&M/Mars, USA)	USA	1997	62±8	16	Normal, 10-12	50	NS	Bread, 2h	Standard	Capillary, plasma	Enzymatic	UO[5]
1661	Milky Way® Lite bar (M&M/Mars, USA)	USA	1997	45±5	11	Normal, 10-12	50	NS	Bread, 2h	Standard	Capillary, plasma	Enzymatic	UO[5]
1662	Munch Peanut Butter bar (M&M/Mars, USA)	USA	2004	27±5	7	Normal, 10	50	134.4	Glucose, 2h	Standard	Capillary, plasma	Enzymatic	UO[5]
	Nuts and Seeds Superfoods bar (SoLo GI Nutrition, Inc, 1663 Canada)	Canada	2018	33±6	8	Normal, 10	25	90.9	Glucose, 2h	Standard	Capillary, whole blood	YSI	UO[7]
1664	Nutbutter bar (SoLo GI Nutrition, Inc, Canada, Canada)	Canada	2018	22±1	6	Normal, 10	25	89.6	Glucose, 2h	Standard	Capillary, whole blood	YSI	UO[7]
1665	Omega bar, containing linseeds, sunflower kernels and nuts, gluten-free (Freedom Foods, Australia)	Australia	2007	21±2	5	Normal, 9	50	118.8	Glucose, 2h	Standard	Capillary, whole blood	HemoCue	UO[10]
1666	Peak Fuel Summit bar, Peanut Brownie (Peak Fuel, Hamner Springs, New Zealand)[13]	New Zealand	2010*	60±5	15	Normal, 20	50	114	Glucose, 2h	Standard	Capillary, whole blood	HemoCue	112
1667	Peanut Butter Chocolate Pal™ bar (Revival Soy®, Physicians Pharmaceuticals, Inc., USA)	USA	2004	52±4	13	Normal, 10	25	48.4	Glucose, 2h	Standard	Capillary, whole blood	HemoCue	21
1668	60% Whole Wheat Fig Fruit bar, President's Choice® Blue Menu™ (Loblaw Brands Limited, Canada)	Canada	2007	72±6	18	Normal, 10	50	69.0	Bread, 2h	Standard	Capillary, whole blood	YSI	UO[7]
1669	Snack bar, Apple Cinnamon (Con Agra Inc., Omaha, NE, USA)	USA	1999	40±8	10	Normal, 10	50	85.2	Bread, 2h	Standard	Capillary, plasma	Enzymatic	UO[5]
	Snack bar, Peanut Butter and Choc-Chip (Con Agra Inc., 1670 USA)	USA	1999	37±6	9	Normal, 10	50	91.6	Bread, 2h	Standard	Capillary, plasma	Enzymatic	UO[5]

Food Number and Item		Country of food production	Year of test[1]	GI[2] ± SEM (Glu = 100)	GL	Subjects (type & number)	Avail carb (Test portion)	Test portion[3] (g)	Reference food & time period	Timepoints	Sample collection	Sample analysis method[4]	Ref.
1671	Soy Joy Fruit & Soy Bar®, Apple (Otsuka Pharmaceutical Co Ltd, Tokyo, Japan)	Japan	2007	19±2	5	Normal, 10	50	130.4	Glucose, 2h	Standard	Capillary, plasma	Enzymatic	UO[5]
1672	Soy Joy Fruit & Soy Bar®, Apricot (Otsuka Pharmaceutical Co Ltd, Tokyo, Japan)	Japan	2007	27±4	7	Normal, 10	50	127.1	Glucose, 2h	Standard	Capillary, plasma	Enzymatic	UO[5]
	Soy Joy Fruit & Soy Bar®, Chocolate Orange (Otsuka 1673 Pharmaceutical Co Ltd, Tokyo, Japan)	Japan	2007	41±7	10	Normal, 10	50	127.1	Glucose, 2h	Standard	Capillary, plasma	Enzymatic	UO[5]
	Soy Joy Fruit & Soy Bar®, Mango Coconut (Otsuka 1674 Pharmaceutical Co Ltd, Tokyo, Japan)	Japan	2007	28±2	7	Normal, 10	50	131.6	Glucose, 2h	Standard	Capillary, plasma	Enzymatic	UO[5]
1675	Soy Joy Fruit & Soy Bar®, Plum + Fe (Otsuka Pharmaceutical Co Ltd, Tokyo, Japan)	Japan	2007	25±3	6	Normal, 10	50	122.0	Glucose, 2h	Standard	Capillary, plasma	Enzymatic	UO[5]
	Soy Joy Fruit & Soy Bar®, Plum + Folic acid (Otsuka 1676 Pharmaceutical Co Ltd, Tokyo, Japan)	Japan	2007	27±2	7	Normal, 10	50	129.3	Glucose, 2h	Standard	Capillary, plasma	Enzymatic	UO[5]
1677	Soy Joy Fruit & Soy Bar®, Raisin Almond (Otsuka Pharmaceutical Co Ltd, Tokyo, Japan)	Japan	2007	28±4	7	Normal, 10	50	128.2	Glucose, 2h	Standard	Capillary, plasma	Enzymatic	UO[5]
1678	Soy Joy Fruit & Soy Bar®, Sanzashi (Otsuka Pharmaceutical Co Ltd, Tokyo, Japan)	Japan	2007	29±5	7	Normal, 10	50	109.5	Glucose, 2h	Standard	Capillary, plasma	Enzymatic	UO[5]
1679	Soy Joy Fruit & Soy Bar®, Strawberry (Otsuka Pharmaceutical Co Ltd, Tokyo, Japan)	Japan	2007	33±2	8	Normal, 9	50	120.0	Glucose, 2h	Standard	Capillary, plasma	Enzymatic	UO[5]
1680	Trim bar, Chocorama (Aussie Bodies Pty. Ltd., Australia)	Australia	2007	31±3	8	Normal, 10	50	181.2	Glucose, 2h	Standard	Capillary, whole blood	HemoCue	UO[10]
1681	Trim bar, Mocha flavor (Aussie Bodies Pty. Ltd., Australia)	Australia	2007	25±3	6	Normal, 10	50	215.5	Glucose, 2h	Standard	Capillary, whole blood	HemoCue	UO[10]
1682	Tronky Nocciola (Wafer bar, hazelnut cream-filled with hazelnut crumbs) (Ferrero, Italy)	Italy	2012	61±7	15	Normal, 10	25	48	Glucose, 2h	Standard	Capillary, whole blood	YSI	3
1683	Twix® Cookie Bar, caramel (M&M/Mars, USA)	USA	1997	44±6	11	Normal, 10-12	50	NS	Bread, 2h	Standard	Capillary, plasma	Enzymatic	25

Food Number and Item		Country of food production	Year of test[1]	GI[2] ± SEM (Glu = 100)	GL	Subjects (type & number)	Avail carb (Test portion)	Test portion[3] (g)	Reference food & time period	Timepoints	Sample collection	Sample analysis method[4]	Ref.
	**Sports (Energy) bars**												
1684	Chocolate Cherry Almond bar (Beachbody, LLC, USA)	USA	2019	22±2	6	Normal, 10	25	86.5	Glucose, 2h	Standard	Capillary, plasma	YSI	UO[7]
	Clif bar, Chocolate Brownie Energy bar (Clif Bar Inc., 1685 Berkeley, USA)	USA	2006	57±6	14	Normal, 10	50	85.0	Glucose, 2h	Standard	Capillary, plasma	Enzymatic	UO[5]
1686	Energy bar, containing with soy protein isolate, peanut butter and corn syrup	Australia	2014*	45±6	11	Normal 10	50	97	Glucose, 2h	Standard	Capillary, plasma	Enzymatic	17
1687	Energy bar with 2.0 g Filtered Molasses Concentrate added per 100 g (4.01 g FMC/100 g carbohydrate)	Australia	2014*	40±4	10	Normal 10	50	100	Glucose, 2h	Standard	Capillary, plasma	Enzymatic	17
1688	Peanut Butter Chocolate bar (Beachbody, LLC)	USA	2019	32±4	8	Normal, 10	25	94.0	Glucose, 2h	Standard	Capillary, plasma	YSI	UO[7]
1689	Performance Chocolate Energy bar (Power Bar, USA)	USA	2006	53±6	13	Normal, 9	50	77.4	Glucose, 2h	Standard	Capillary, plasma	Enzymatic	UO[5]
1690	Power Bar®, chocolate **(**Powerfood Inc., Berkeley, USA)	USA	1997	58±5	15	Normal, 10	50	NS	Bread, 2h	Standard	Capillary, plasma	Enzymatic	25
1691	SmartZone Chocolate flavor, Nutrition bar (Hershey's Food Corporation, Hershey, PA, USA)	USA	2004	16±3	4	Normal, 10	50	138.9	Glucose, 2h	Standard	Capillary, plasma	Enzymatic	UO[5]
1692	SmartZone Crunchy Blueberry flavor, Nutrition bar (Hershey's Food Corporation, Hershey, PA, USA)	USA	2005	15±3	4	Normal, 10	50	125.0	Glucose, 2h	Standard	Capillary, plasma	Enzymatic	UO[5]
1693	SmartZone Crunchy Chocolate Brownie flavor, Nutrition bar (Hershey's Food Corporation, Hershey, PA, USA)	USA	2005	23±5	6	Normal, 10	50	138.9	Glucose, 2h	Standard	Capillary, plasma	Enzymatic	UO[5]
1694	SmartZone Crunchy Chocolate Caramel flavor, Nutrition bar (Hershey's Food Corporation, Hershey, PA, USA)	USA	2005	16±4	4	Normal, 10	50	119.0	Glucose, 2h	Standard	Capillary, plasma	Enzymatic	UO[5]
1695	SmartZone Crunchy Chocolate Peanut Butter flavor, Nutrition bar (Hershey's Food Corporation, Hershey, PA, USA)	USA	2005	14±3	4	Normal, 10	50	138.9	Glucose, 2h	Standard	Capillary, plasma	Enzymatic	UO[5]
1696	SmartZone Crunchy Key Lime flavor, Nutrition bar (Hershey's Food Corporation, Hershey, PA, USA)	USA	2005	14±3	4	Normal, 10	50	125.0	Glucose, 2h	Standard	Capillary, plasma	Enzymatic	UO[5]
1697	SmartZone Peanut Butter flavor, Nutrition bar (Hershey's Food Corporation, Hershey, PA, USA)	USA	2005	18±2	5	Normal, 10	50	138.9	Glucose, 2h	Standard	Capillary, plasma	Enzymatic	UO[5]

Food Number and Item	Country of food production	Year of test[1]	GI[2] ± SEM (glu = 100)	GL	Subjects (type & number)	Avail carb (Test portion)	Test portion[3] (g)	Reference food & time period	Timepoints	Sample collection	Sample analysis method[4]	Ref.
1698 Snickers® Marathon Energy bar, Chewy Chocolate Peanut flavor (M&M/Mars, USA)	USA	2005	36±5	9	Normal, 10	50	97.4	Glucose, 2h	Standard	Capillary, plasma	Enzymatic	UO[5]
1699 Snickers® Marathon Energy bar, Cookies & Crème flavor (M&M/Mars, USA)	USA	2005	50±4	13	Normal, 10	50	91.7	Glucose, 2h	Standard	Capillary, plasma	Enzymatic	UO[5]
1700 Snickers® Marathon Energy bar, Multi Grain Crunch flavor (M&M/Mars, USA)	USA	2005	50±4	13	Normal, 10	50	92.6	Glucose, 2h	Standard	Capillary, plasma	Enzymatic	UO[5]
Snickers® Marathon Energy bar, Peanut Butter flavor 1701 (M&M/Mars, USA)	USA	2005	34±5	9	Normal, 10	50	105.8	Glucose, 2h	Standard	Capillary, plasma	Enzymatic	UO[5]
Snickers® Marathon Low Carb Lifestyle Energy bar, 1702 Chocolate Fudge Brownie flavor (M&M/Mars, USA)	USA	2003	20±4	5	Normal, 10	50	NS	Glucose, 2h	Standard	Capillary, plasma	Enzymatic	UO[5]
1703 Snickers® Marathon Low Carb Lifestyle Energy bar, Peanut Butter flavor (M&M/Mars, USA)	USA	2003	21±4	5	Normal, 10	50	NS	Glucose, 2h	Standard	Capillary, plasma	Enzymatic	UO[5]
1704 Snickers® Marathon Nutrition bar, Dark Chocolate Crunch flavor (M&M/Mars, USA)	USA	2003	49±6	12	Normal, 10	50	NS	Glucose, 2h	Standard	Capillary, plasma	Enzymatic	UO[5]
1705 Snickers® Marathon Nutrition bar, Honey & Roasted Almond flavor (M&M/Mars, USA)	USA	2003	41±3	10	Normal, 10	50	NS	Glucose, 2h	Standard	Capillary, plasma	Enzymatic	UO[5]
1706 Snickers® Marathon Protein Performance bar, Caramel Nut Rush flavor (M&M/Mars, USA)	USA	2003	26±3	7	Normal, 10	50	NS	Glucose, 2h	Standard	Capillary, plasma	Enzymatic	UO[5]
1707 Snickers® Marathon Protein Performance bar, Chocolate Nut Burst flavor (M&M/Mars, USA)	USA	2003	32±4	8	Normal, 10	50	NS	Glucose, 2h	Standard	Capillary, plasma	Enzymatic	UO[5]
1708 SoLo GI Berry Bliss Nutrition bar (New Era Nutrition Inc, Alberta, Canada)	Canada	2004-2007	29±5	7	Normal, 10	50	113.6	Bread, 2h	Standard	Capillary, whole blood	YSI	UO[7]
1709 SoLo GI Chocolate Charger Nutrition bar (New Era Nutrition Inc, Alberta, Canada)	Canada	2004-2007	28±2	7	Normal, 10	50	113.6	Bread, 2h	Standard	Capillary, whole blood	YSI	UO[7]
1710 SoLo GI Lemon Lift Nutrition bar (New Era Nutrition Inc, Alberta, Canada)	Canada	2004-2007	29±5	7	Normal, 10	50	113.6	Bread, 2h	Standard	Capillary, whole blood	YSI	UO[7]
1711 SoLo GI Mint Mania Nutrition bar (New Era Nutrition Inc, Alberta, Canada)	Canada	2004-2007	23±3	6	Normal, 10	50	113.6	Bread, 2h	Standard	Capillary, whole blood	YSI	UO[7]

1712	SoLo GI Peanut Power Nutrition bar (New Era Nutrition Inc, Alberta, Canada)	Canada	2004-2007	27±4	7	Normal, 10	50	125	Bread, 2h	Standard	Capillary, whole blood	YSI	UO[7]
1713	SoLo GI Berry Bliss Snack bar (New Era Nutrition Inc, Alberta, Canada)	Canada	2004-2007	29±5	7	Normal, 10	50	113.6	Bread, 2h	Standard	Capillary, whole blood	YSI	UO[7]
1714	SoLo GI Chocolate Charger Snack bar (New Era Nutrition Inc, Alberta, Canada)	Canada	2004-2007	28±2	7	Normal, 10	50	125.0	Bread, 2h	Standard	Capillary, whole blood	YSI	UO[7]
1715	SoLo GI Lemon Lift Snack bar (New Era Nutrition Inc, Alberta, Canada)	Canada	2004-2007	29±5	7	Normal, 10	50	113.6	Bread, 2h	Standard	Capillary, whole blood	YSI	UO[7]
1716	SoLo GI Mint Mania Snack bar (New Era Nutrition Inc, Alberta, Canada)	Canada	2004-2007	23±3	6	Normal, 10	50	125.0	Bread, 2h	Standard	Capillary, whole blood	YSI	UO[7]
1717	SoLo GI Peanut Power Snack bar (New Era Nutrition Inc, Alberta, Canada)	Canada	2004-2007	27±4	7	Normal, 10	50	125.0	Bread, 2h	Standard	Capillary, whole blood	YSI	UO[7]
1718	VO_2 Max Chocolate Energy bar (M&M/Mars, USA)	USA	1997	49±8	12	Normal, 10-12	50	NS	Bread, 2h	Standard	Capillary, plasma	Enzymatic	25

SOUPS

Average carbohydrate portion = 20 g, this value was used to determine the nominal GL for each item in this category.

1719	Barley Vegetable Instant soup, low-fat, President's Choice® Blue Menu™ (Loblaw Brands Limited, Canada)	Canada	2004-2007	41±3	8	Normal, 10	50	NS	Bread, 2h	Standard	Capillary, whole blood	YSI	UO[7]
1720	Black Bean Instant soup, low-fat, spicy, President's Choice® Blue Menu™ (Loblaw Brands Limited, Canada)	Canada	2004-2007	57±5	11	Normal, 10	50	NS	Bread, 2h	Standard	Capillary, whole blood	YSI	UO[7]
1721	Black Bean with Vegetables soup, spicy, President's Choice® Blue Menu™ (Loblaw Brands Limited, Canada)	Canada	2007	46±5	9	Normal, 10	50	463.0	Bread, 2h	Standard	Capillary, whole blood	YSI	UO[7]
1722	Carrot soup, President's Choice® Blue Menu™ Soupreme (Loblaw Brands Limited, Canada)	Canada	2007	35±4	7	Normal, 10	50	961.5	Bread, 2h	Standard	Capillary, whole blood	YSI	UO[7]
1723	Chicken & Vegetable with wholegrain pasta, Country Ladle® (Campbell's Soups, Homebush, NSW, Australia)	Australia	2008	43±3	9	Normal, 9	25	558.0	Glucose, 2h	Standard	Capillary, plasma	Enzymatic	UO[5]
1724	Chunky Roast Chicken and Vegetable soup (Campbell's Soups, Homebush, NSW, Australia)	Australia	2016	52±3	10	Normal, 9	50	724.6	Glucose, 2h	Standard	Capillary, plasma	Enzymatic	UO[5]
1725	Lentil Instant soup, low-fat, Indian, President's Choice® Blue Menu™ (Loblaw Brands Limited, Canada)	Canada	2004-2007	62±7	12	Normal, 10	50	100.0	Bread, 2h	Standard	Capillary, whole blood	YSI	UO[7]
1726	Lentil, ready-to-serve, President's Choice® Blue Menu™ (Loblaw Brands Limited, Canada)	Canada	2004-2007	57±9	11	Normal, 10	50	657.9	Bread, 2h	Standard	Capillary, whole blood	YSI	UO[7]
1727	Minestrone, condensed, prepared with water (Campbell's Soup Company, Camden, NJ, USA)	USA	2002	48±6	10	Normal, 10	50	450	Glucose, 2h	Standard	Capillary, whole blood	HemoCue	UO[5]

Food Number and Item		Country of food production	Year of test[1]	GI[2] ± SEM (lu = 100)	GL	Subjects (type & number)	Avail carb (Test portion)	Test portion[3] (g)	Reference food & time period	Timepoints	Sample collection	Sample analysis method[4]	Ref.
1728	Minestrone, Traditional, Country Ladle® (Campbell's Soups, Australia)	Australia	2001	39±3	8	Normal, 10	25	833.3	Glucose, 2h	Standard	Capillary, whole blood	HemoCue	UO[5]
1729	Minestrone & Pasta Instant soup, low-fat, President's Choice® Blue Menu™ (Loblaw Brands Limited, Canada)	Canada	2004-2007	54±3	11	Normal, 10	50	NS	Bread, 2h	Standard	Capillary, whole blood	YSI	UO[7]
	Minestrone mean of three foods			47									
1730	Mushroom Barley, ready-to-serve, President's Choice® Blue Menu™ (Loblaw Brands Limited, Canada)	Canada	2004-2007	45±10	9	Normal, 10	50	NS	Bread, 2h	Standard	Capillary, whole blood	YSI	UO[7]
1731	Pasta & Fagioli soup, ready-to-serve, President's Choice® Blue Menu™ (Loblaw Brands Limited, Canada)	Canada	2004-2007	52±8	10	Normal, 10	50	NS	Bread, 2h	Standard	Capillary, whole blood	YSI	UO[7]
1732	Potato & Bacon soup, chunky (Campbell's Soups, Homebush, NSW, Australia)	Australia	2016	41±4	8	Normal, 10	50	625.0	Glucose, 2h	Standard	Capillary, plasma	Enzymatic	UO[5]
1733	Pumpkin Soup, creamy, Heinz® Very Special™, with pumpkin, cream, potatoes (Heinz Wattie's Ltd, Australia)	Australia	2004	76±7	15	Normal, 10	50	694.4	Glucose, 2h	Standard	Capillary, plasma	Enzymatic	UO[5]
1734	Thai Instant soup, low-fat, President's Choice® Blue Menu™ (Loblaw Brands Limited, Canada)	Canada	2004-2007	56±4	11	Normal, 10	50	NS	Bread, 2h	Standard	Capillary, whole blood	YSI	UO[7]
1735	Tomato soup, condensed, prepared with water (Campbell's Soup Company, Camden, NJ, USA)	USA	2002	52±4	10	Normal, 10	50	326.8	Glucose, 2h	Standard	Capillary, whole blood	HemoCue	UO[5]
1736	Tomato & Herb, President's Choice® Blue Menu™ Soupreme (Loblaw Brands Limited, Canada)	Canada	2007	47±5	9	Normal, 10	50	892.9	Bread, 2h	Standard	Capillary, whole blood	YSI	UO[7]
1737	Vegetable Couscous Instant soup, low-fat, President's Choice® Blue Menu™ (Loblaw Brands Limited, Canada)	Canada	2004-2007	57±4	11	Normal, 10	50	NS	Bread, 2h	Standard	Capillary, whole blood	YSI	UO[7]
1738	Vegetarian Chili Instant soup, low-fat, President's Choice® Blue Menu™ (Loblaw Brands Limited, Canada)	Canada	2004-2007	36±4	7	Normal, 10	50	NS	Bread, 2h	Standard	Capillary, whole blood	YSI	UO[7]
1739	Winter Squash, President's Choice® Blue Menu™ Soupreme (Loblaw Brands Limited, Canada)	Canada	2007	41±4	8	Normal, 10	50	961.5	Bread, 2h	Standard	Capillary, whole blood	YSI	UO[7]
	**SUGARS AND SYRUPS**												
	Average carbohydrate portion = 5 g, this value was used to deter ine the nominal GL for each item in this tegory.												
	**Agave cactus nectar, high-fructose**												
1740	Organic Agave Cactus Nectar, light, 90% fructose (Western Commerce Corp., City of Industry, CA, USA)	USA	2001	11±1	1	Normal, 9	25	NS	Bread, 2h	Standard	Capillary, plasma	Enzymatic	UO[5]

1741	Organic Agave Cactus Nectar, light, 97% fructose (Western Commerce Corp., USA)	USA	2001	10±1	1	Normal, 9	25	NS	Bread, 2h	Standard	Capillary, plasma	Enzymatic	UO[5]
1742	Premium Agave nectar (Sweet Cactus Farms, USA)	USA	2005	19±4	1	Normal, 9	50	62.7	Glucose, 2h	Standard	Capillary, plasma	Enzymatic	UO[5]
	Agave nectar, mean of three foods			13									

**Food Number and Item**		**Country of food production**	**Year of test[1]**	**GI[2] ± SEM (Glu = 100)**	**GL**	**Subjects (type & number)**	**Avail carb (Test portion)**	**Test portion[3] (g)**	**Reference food & time period**	**Timepoints**	**Sample collection**	**Sample analysis method[4]**	**Ref.**
	**Fructose**												
1743	Fructose	China	2005	23±1	1	Normal, 10	50	NS	Glucose, 2h	Standard	Venous, NS	NS	12
1744	Fructose from corn (Despar, Italy)	Italy	2013	25±4	1	Normal, 10	25	25	Glucose, 2h	Standard	Capillary, whole blood	YSI	3
	Fructose, mean of two studies			24									
1745	High fructose corn syrup	Australia	2014*	56±5	3	Normal 10	50	65	Glucose, 2h	Standard	Capillary, plasma	Enzymatic	17
1746	High fructose corn syrup with 3.3 g Filtered Molasses Concentrate added per 100 g (4.16 g FMC/100 g carbohydrate)	Australia	2014*	50±3	3	Normal 10	50	62	Glucose, 2h	Standard	Capillary, plasma	Enzymatic	17
	**Glucose**												
1747	Glucose syrup	Australia	2014*	107±7	5	Normal 10	50	61	Glucose, 2h	Standard	Capillary, plasma	Enzymatic	17
	**Honey**												
1748	Yellow box honey (46% fructose)	Australia	2001	35±4	2	Normal, 10	25	67.3	Glucose, 2h	Standard	Capillary, plasma	Enzymatic	UO[5]
1749	Stringy Bark honey (52% fructose)	Australia	2001	44±4	2	Normal, 9	25	67.3	Glucose, 2h	Standard	Capillary, plasma	Enzymatic	UO[5]
1750	Red Gum honey (35% fructose)	Australia	2001	46±3	2	Normal, 9	25	66.0	Glucose, 2h	Standard	Capillary, plasma	Enzymatic	UO[5]
1751	Iron Bark honey (34% fructose)	Australia	2001	48±3	2	Normal, 10	25	83.5	Glucose, 2h	Standard	Capillary, plasma	Enzymatic	UO[5]
1752	Capilano Premium Honey, blend of eucalypt & floral honeys (Capilano Honey Limited, QLD, Australia)	Australia	2006	51±3	3	Normal, 11	50	60.2	Glucose, 2h	Standard	Capillary, plasma	Enzymatic	UO[5]

Food Number and Item		Country of food production	Year of test[1]	GI[2] ± SEM (Glu = 100)	GL	Subjects (type & number)	Avail carb (Test portion)	Test portion[3] (g)	Reference food & time period	Timepoints	Sample collection	Sample analysis method[4]	Ref.
1753	Yapunya honey (42 % fructose)	Australia	2001	52±5	3	Normal, 9	25	73.9	Glucose, 2h	Standard	Capillary, plasma	Enzymatic	UO[5]
1754	HDI Clover Honey with Bee Pollen (PT. Harmoni Dinamik)	New Zealand	2020	53±5	3	Normal, 10	50	60.2	Glucose, 2h	Standard	Capillary, plasma	Enzymatic	UO[5]
1755	Leabrook Farms Yellow box honey (Spring Gully Foods Pty Ltd, SA, Australia)	Australia	2006	60±4	3	Normal, 11	50	58.6	Glucose, 2h	Standard	Capillary, plasma	Enzymatic	UO[5]
	Commercial Blend honey (38% fructose) (WA blend, 1756 Capilano Honey Ltd., Australia)	Australia	2001	62±3	3	Normal, 9	25	71.2	Glucose, 2h	Standard	Capillary, plasma	Enzymatic	UO[5]
1757	Salvation Jane honey (32% fructose)	Australia	2001	64±5	3	Normal, 10	25	81.2	Glucose, 2h	Standard	Capillary, plasma	Enzymatic	UO[5]
1758	Manuka honey, MGO 440+ (Manuka Health New Zealand Ltd)	New Zealand	2011	65±7	3	Normal, 10	25	32.1	Glucose, 2h	Standard	Capillary, whole blood	HemoCue	113
1759	Clover honey, ratio of fructose: glucose, 1.09 (Vazza Farms, Hermiston, OR, USA)[16]	USA	2006	69±8	3	Normal, 12	50	58.3	Glucose, 2h	Standard	Capillary, plasma	Enzymatic	114
	Commercial Blend honey (28% fructose) (NSW blend, 1760 Capilano Honey Ltd., Australia)	Australia	2001	72±6	4	Normal, 9	25	99.2	Glucose, 2h	Standard	Capillary, plasma	Enzymatic	UO[5]
1761	Buckwheat honey, ratio of fructose:glucose, 1.12 Vazza Farms, USA)[16]	USA	2006	73±6	4	Normal, 11	50	58.4	Glucose, 2h	Standard	Capillary, plasma	Enzymatic	114
1762	Cotton honey, ratio of fructose:glucose, 1.03 (Gene Brandi Apiaries, Los Banos, CA, USA)[16]	USA	2006	74±7	4	Normal, 11	50	57.0	Glucose, 2h	Standard	Capillary, plasma	Enzymatic	114
1763	Honey, NS	China	2005	74±4	4	Normal, 10	50	NS	Glucose, 2h	Standard	Venous, NS	NS	12
	Tupelo honey, ratio of fructose:glucose, 1.54 (Tropical 1764 Blossom Honey Co, Edgewater, FL, USA)	USA	2006	74±8	4	Normal, 12	50	59.2	Glucose, 2h	Standard	Capillary, plasma	Enzymatic	114
	Honey, mean of 17 types of honey			60									
	**Lactose**												
1765	Lactose	China	2005	46±1	2	Normal, 10	50	NS	Glucose, 2h	Standard	Venous, NS	NS	12

Food Number	and Item	Country of food production	Year of test[1]	GI[2] ± SEM (Glu = 100)	GL	Subjects (type & number)	Avail carb (Test portion)	Test portion[3] (g)	Reference food & time period	Timepoints	Sample collection	Sample analysis method[4]	Ref.
	**Maltose**												
1766	Maltose	China	2005	75±6	4	Normal, 10	50	NS	Glucose, 2h	Standard	Venous, NS	NS	12
1767	Maltose	China	2005	105±2	5	Normal, 10	50	NS	Glucose, 2h	Standard	Venous, NS	NS	12
	Maltose, mean of two studies			90									
	**Sucrose**												
1768	Sucrose	Australia	2007	59±4	3	Normal, 11	50	50	Glucose, 2h	Standard	Capillary, plasma	Enzymatic	UO[5]
1769	Sucrose, cane sugar	Australia	2016	60±4	3	Normal, 10	50	50	Glucose, 2h	Standard	Capillary, plasma	Enzymatic	UO[5]
1770	Sucrose	Australia	2007	61±4	3	Normal, 11	50	50	Glucose, 2h	Standard	Capillary, plasma	Enzymatic	UO[5]
1771	Sucrose	Germany	2004	64±4	3	Normal, 11	50	50	Glucose, 2h	Standard	Capillary, plasma	Enzymatic	UO[5]
1772	Sucrose	Germany	2002	66±3	3	Normal, 10	50	50	Glucose, 2h	Standard	Capillary, plasma	Enzymatic	UO[5]
1773	Sugar, cane (Panela)	Italy	2005	69±10	3	Normal, 9	25	25	Glucose, 2h	Standard	Capillary, whole blood	YSI	3
1774	Sucrose	China	2005	84±4	4	Normal, 10	50	NS	Glucose, 2h	Standard	Venous, NS	NS	12
	Sucrose, mean of seven studies			66									
1775	Sucrose (LoGiCane) (Horizon Science, Australia)[13]	Australia	2014*	60±3	3	Normal, European, 31	50	NS	Glucose, 2h	Standard	Capillary, whole blood	HemoCue	55
1776	Sucrose (LoGiCane) (Horizon Science, Australia)[13]	Australia	2014*	62±4	3	Normal, Chinese, 32	50	NS	Glucose, 2h	Standard	Capillary, whole blood	HemoCue	55
1777	Sugar, refined (Sadam-Eridania, Italy)	Italy	2013	91±10	5	Normal, 10	25	25	Glucose, 2h	Standard	Capillary, whole blood	YSI	3

	Food Number and Item	Country of food production	Year of[1]	GI[2] ± SEM (Glu = 100)	GL	Subjects (type & number)	Avail carb (Test portion)	Test portion (g)[3]	Reference food & time period	Sample collection	Sample analysis method[4]	Ref.
	**Other sugars & syrups**											
1778	Arabinose Sugar (95% sucrose + 5% L-arabinose) (Healtang Biotech Co., Ltd, China)	China	2020	52±4	3	Normal, 10	25	26.4	Glucose, 2h	Standard	Capillary, plasma	Enzymatic	UO[18]
1779	Coconut sugar, organic, Loving Earth™ brand	Indonesia	2014	54±6	3	Normal, 9	50	50.0	Glucose, 2h	Standard	Capillary, plasma	Enzymatic	UO[5]
1780	Dates Syrup, Gurun Emas (Omni Mal Agencies Sdn. Bhd, Malaysia)	Malaysia	2018	54±4	3	Normal, 10	50	67.4	Glucose, 2h	Standard	Capillary, plasma	Enzymatic	UO[5]
1781	Fruit Up sweetener syrup (Rudolf Wild Gmbh & Co, Germany)	Germany	2008	39±4	2	Normal, 10	50	70.4	Glucose, 2h	Standard	Capillary, plasma	Enzymatic	UO[5]
1782	Golden Syrup	Australia	2003	63±6	3	Normal, 9	50	59.9	Glucose, 2h	Standard	Capillary, plasma	Enzymatic	UO[5]
1783	Karo Dark Corn Syrup	USA	2013	90±6	5	Normal, 10	50	66.1	Glucose, 2h	Standard	Capillary, plasma	Enzymatic	UO[5]
1784	Maple flavored syrup (Cottee's Foods, Australia)	Australia	2003	68±5	3	Normal, 9	50	55.7	Glucose, 2h	Standard	Capillary, plasma	Enzymatic	UO[5]
1785	Maple syrup, pure Canadian (Queen Foods, Australia)	Canada	2003	54±6	3	Normal, 10	50	68.2	Glucose, 2h	Standard	Capillary, plasma	Enzymatic	UO[5]
1786	Rice malt syrup (Pure Harvest Organic, Australia)	Australia	2013	98±6	5	Normal, 10	50	62.5	Glucose, 2h	Standard	Capillary, plasma	Enzymatic	UO[5]
1787	Sugar, from organic fruits (Dolcedì, zucchero da frutta biologica, Rigoni di Asiago, Italy)	Italy	2009	23±5	1	Normal, 10	50	72	Glucose, 2h	Standard	Capillary, whole blood	YSI	3
1788	Snow Pear Red Ginseng Brown Sugar, contains 2.2% ® (Queen's Joy, Wuzuo Technology, Tianjin, China) Phytolin	China	2020	45±3	2	Normal, 12	25	27.3	Glucose, 2h	Standard	Capillary, plasma	Enzymatic	UO[18]
1789	Xylose Sugar (90% sucrose + 10% D-Xylose) (Healtang Biotech Co., Ltd, China)	China	2020	52±3	3	Normal, 10	25	27.8	Glucose, 2h	Standard	Capillary, plasma	Enzymatic	UO[18]

	**VEGETABLES**												
	Average carbohydrate portion = 20 g, this value was used to dete mine the nominal GL for each item in this category, except for green peas, pumpkin, carrot, parsnip, and tomato sauce products where 10 g was used.												
	**Green banana/plantain (*Musa sapientum*)**												
1790	Green banana, peeled, boiled 10 min	Jamaica	2005	37±5	7	Normal, 10	50	225.2	Glucose, 2h	Standard	Capillary, plasma	Enzymatic	88
1791	Green banana, peeled, fried in vegetable oil	Jamaica	2005	35±3	7	Normal, 10	50	195.3	Glucose, 2h	Standard	Capillary, plasma	Enzymatic	88
1792	Green plantain (*Musa paradisiaca*), peeled, boiled 10 min	Jamaica	2005	39±4	8	Normal, 10	50	259.2	Glucose, 2h	Standard	Capillary, plasma	Enzymatic	88
1793	Green plantain (*Musa paradisiaca*), peeled, fried in vegetable oil	Jamaica	2005	40±3	8	Normal, 10	50	175.9	Glucose, 2h	Standard	Capillary, plasma	Enzymatic	88
1794	Plantain (*Musa* spp. Ameletiha) chips (made from green plantain), sliced 0.8-1.2 mm thick, deep-fried	Côte d'Ivoire	2017*	45±0.3	9	Normal, 28	50	93.8	Glucose, 2h	Standard	Capillary, whole blood	Glucometer[9]	115
	Green banana, mean of five foods			39									
	**Ripe banana/plantain (*Musa sapientum*)**												
1795	Ripe plantain (*Musa paradisiaca*), peeled, boiled 10 min	Jamaica	2005	66±2	13	Normal, 10	50	308.6	Glucose, 2h	Standard	Capillary, plasma	Enzymatic	88
1796	Ripe plantain (*Musa paradisiaca*), peeled, fried in vegetable oil	Jamaica	2005	90±6	18	Normal, 10	50	211.6	Glucose, 2h	Standard	Capillary, plasma	Enzymatic	88
	**Green peas**												
1797	Peas, plain and frozen (Talleys Group Ltd, New Zealand)[13]	New Zealand	2011*	29±2	3	Normal, 30	25	NS	Glucose, 2h	Standard	Capillary, whole blood	HemoCue	69
1798	Peas, frozen, heated in the microwave (McCain Foods Aust. Pty Ltd, Australia)	Australia	2020	42±4	4	Normal, 10	25	238.1	Glucose, 2h	Standard	Capillary, plasma	Enzymatic	UO[5]
	Peas, mean of two studies			36									
	**Pumpkin**												
1799	Butternut pumpkin, boiled	Australia	2006	51±6	5	Normal, 10	25	352.1	Glucose, 2h	Standard	Capillary, plasma	Enzymatic	UO[5]
1800	Pumpkin (*Cucurbita moschata*), peeled, cubed, boiled 30 min	Jamaica	2005	66±4	7	Normal, 10	50	223.8	Glucose, 2h	Standard	Capillary, plasma	Enzymatic	88
	**Sweet corn**												
1801	Sweet corn, cooked in microwave for 1.5 min	Australia	2015	51±4	10	Normal, 10	25	185.2	Glucose, 2h	Standard	Capillary, plasma	Enzymatic	UO[5]

Food Number and Item		Country of food production	Year of test[1]	GI[2] ± SEM (Glu = 100)	GL	Subjects (type & number)	Avail carb (Test portion)	Test portion[3] (g)	Reference food & time period	Timepoints	Sample collection	Sample analysis method[4]	Ref.
1802	Sweet corn	China	2005	55±2	11	Normal, 10	50	NS	Glucose, 2h	Standard	Venous, NS	NS	12
	Sweet corn, mean of two foods			53									
	**Root vegetables**												
1803	Eddoe (*Colocasia esculenta var. antiquorum*), peeled, boiled 30 min, refrigerated, reheated for 1 min	Canada	2003	61±10	12	Normal, 10	50	221	Bread, 2h	Standard	Capillary, plasma	Enzymatic	116
1804	Carrots, unpeeled, boiled	Australia	2020	32±4	3	Normal, 10	25	409.8	Glucose, 2h	Standard	Capillary, plasma	Enzymatic	UO[5]
1805	Carrots, diced, frozen (Talleys Group Ltd) [13]	New Zealand	2011*	31±2	3	Normal, 30	25	NS	Glucose, 2h	Standard	Capillary, whole blood	HemoCue	69
	Carrots, mean of two foods			32									
1806	Parsnips, peeled, boiled	Australia	2007	52±5	5	Normal, 10	25	250	Glucose, 2h	Standard	Capillary, plasma	Enzymatic	UO[5]
1807	Tannia (*Xanthosoma sagittifolium*), peeled, boiled 30 min, refrigerated, reheated for 1 min	Canada	2003	60±5	12	Normal, 10	50	135	Bread, 2h	Standard	Capillary, plasma	Enzymatic	116
	**Potato**												
	**Baked potato**												
1808	Irish potato (*Solanum tuberosum*), peeled, baked 45 min	Jamaica	2005	83±6	17	Normal, 10	50	249.6	Glucose, 2h	Standard	Capillary, plasma	Enzymatic	88
1809	Pontiac potato, peeled and baked	Australia	1998	93±11	19	Normal, 10	50	414	Bread, 2h	Standard	Capillary, plasma	Enzymatic	117
	**Boiled potato**												
1810	Bintje potato, peeled, cut into 5 cm by 5 cm slices, at a thickness of 0.5 cm, boiled for 8-9 min	Australia	2012	94±8	19	Normal, 10	25	189	Glucose, 2h	Standard	Capillary, plasma	Enzymatic	118
1811	Carisma potato, peeled, cut into 5 cm by 5 cm slices, at a thickness of 0.5 cm, boiled for 8-9 min	Australia	2011	53±7	11	Normal, 10	25	238	Glucose, 2h	Standard	Capillary, plasma	Enzymatic	118
1812	Carisma potato, baby, peeled, cut into slices 1 cm thick, boiled for 8 min	Australia	2016	57±5	11	Normal, 10	25	277.8	Glucose, 2h	Standard	Capillary, plasma	Enzymatic	UO[5]
1813	Charlotte potato, peeled, quartered, boiled 15 min	UK	2005	66±5	13	Normal, 10	50	NS	Glucose, 2h	Standard	Capillary, whole blood	Glucometer[9]	119

Food Number and Item	Country of food production	Year of test[1]	GI[2] ± SEM (Glu = 100)	GL	Subjects (type & number)	Avail carb (Test portion)	Test portion[3] (g)	Reference food & time period	Timepoints	Sample collection	Sample analysis method[4]	Ref.
1814 Charlotte potato, unpeeled, cut into slices 1 cm thick, boiled for 8 min	Australia	2009	68±6	14	Normal, 11	50	359.7	Glucose, 2h	Standard	Capillary, plasma	Enzymatic	UO[5]
1815 Desiree potato, peeled, boiled 35 min	Australia	1998	101±15	20	Normal, 10	50	446	Bread, 2h	Standard	Capillary, plasma	Enzymatic	117
1816 Desiree potato, peeled, cut into 5 cm by 5 cm slices, at a thickness of 0.5 cm, boiled for 8-9 min	Australia	2011	74±8	15	Normal, 10	25	197	Glucose, 2h	Standard	Capillary, plasma	Enzymatic	118
1817 Desiree potato, peeled, cut into 5 cm by 5 cm slices, at a thickness of 0.5 cm, boiled for 8-9 min	Australia	2016	79±6	16	Normal, 10	25	196.9	Glucose, 2h	Standard	Capillary, plasma	Enzymatic	UO[5]
1818 Estima potato, peeled, quartered, boiled 15 min	UK	2005	66±5	13	Normal, 10	50	NS	Glucose, 2h	Standard	Capillary, whole blood	Glucometer[9]	119
1819 King Edward potato, peeled, quartered, boiled 15 min in unsalted water	UK	2005	75±10	15	Normal, 10	50	NS	Glucose, 2h	Standard	Capillary, whole blood	Glucometer[9]	119
1820 Maiflower potato, peeled, cut into 5 cm by 5 cm slices, at a thickness of 0.5 cm, boiled for 8-9 min	Australia	2012	103±8	21	Normal, 10	25	189	Glucose, 2h	Standard	Capillary, plasma	Enzymatic	118
1821 Marfona potato, peeled, quartered, boiled 15 min in unsalted water	UK	2005	56±3	11	Normal, 10	50	NS	Glucose, 2h	Standard	Capillary, whole blood	Glucometer[9]	119
1822 Maris Piper potato, peeled, quartered, boiled 15 min in unsalted water	UK	2005	85±4	17	Normal, 10	50	NS	Glucose, 2h	Standard	Capillary, whole blood	Glucometer[9]	119
1823 Nicola potato, peeled, quartered, boiled 15 min	UK	2005	59±7	12	Normal, 10	50	NS	Glucose, 2h	Standard	Capillary, whole blood	Glucometer[9]	119
1824 Nicola potato, unpeeled, boiled whole for 15 min	Australia	2006	58±3	12	Normal, 10	50	471.7	Glucose, 2h	Standard	Capillary, plasma	Enzymatic	UO[5]
1825 Nicola potato, peeled, cut into 5 cm by 5 cm slices, at a thickness of 0.5 cm, boiled for 8-9 min	Australia	2012	69±5	14	Normal, 10	25	195	Glucose, 2h	Standard	Capillary, plasma	Enzymatic	118
1826 Pontiac potato, peeled, boiled 35 min	Australia	1998	88±9	18	Normal, 10	50	414	Bread, 2h	Standard	Capillary, plasma	Enzymatic	117
1827 Russet Burbank potato, peeled, cut into 5 cm by 5 cm slices, at a thickness of 0.5 cm, boiled for 8-9 min	Australia	2012	82±3	16	Normal, 10	25	154	Glucose, 2h	Standard	Capillary, plasma	Enzymatic	118

Food Number and Item		Country of food production	Year of test[1]	GI[2] ± SEM (Glu = 100)	GL	Subjects (type & number)	Avail carb (Test portion)	Test portion[3] (g)	Reference food & time period	Timepoints	Sample collection	Sample analysis method[4]	Ref.
1828	Sebago potato, peeled, boiled for 35 min	Australia	1998	87±7	17	Normal, 10	50	454	Bread, 2h	Standard	Capillary, plasma	Enzymatic	117
1829	Virginia Rose potato, peeled, cut into 5 cm by 5 cm slices, at a thickness of 0.5 cm, boiled for 8-9 min	Australia	2012	93±10	19	Normal, 10	25	255	Glucose, 2h	Standard	Capillary, plasma	Enzymatic	118
1830	Potato, novel clone, selection 1 (CV92056-4, grown to maturity in Alberta, harvested Oct. 2008), unpeeled, cut into 2.5-3 cm cubes, boiled in salted water for 15 min	Canada	2009	60±5	12	Normal, 9	50	345	Bread, 2h	Standard	Capillary, whole blood	YSI	110
1831	Potato, novel clone, selection 1 (CV92056-4, grown to maturity in Alberta; harvested Oct. 2008), boiled for 15 min, reheated in microwave for 2-3 min	Canada	2009	75±5	15	Normal, 10	50	345	Bread, 2h	Standard	Capillary, whole blood	YSI	110
1832	Potato, novel clone, selection 1 (CV92056-4, grown in Ontario; harvested Oct. 2009), unpeeled, cut into 2.5-3 cm cubes, boiled in salted water for 15 min	Canada	2009-2010	82±7	16	Normal, 9	50	345	Bread, 2h	Standard	Capillary, whole blood	YSI	110
1833	Potato, novel clone, selection 2 (CV1102-1, grown in Alberta; harvested prior to maturity Oct. 2009), unpeeled, cut into 2.53 cm cubes, boiled in salted water for 15 min	Canada	2009-2010	38±6	8	Normal, 10	50	360	Bread, 2h	Standard	Capillary, whole blood	YSI	110
1834	Potato, novel clone, selection 3 (F03031, grown in New Brunswick), unpeeled, cut into 2.5-3 cm cubes, boiled in salted water for 15 min	Canada	2009	79±9	16	Normal, 10	50	321	Bread, 2h	Standard	Capillary, whole blood	YSI	110
1835	Potato, novel clone, selection 3 (F03031, grown in New Brunswick), boiled 15 min, reheated in microwave 2-3 min	Canada	2009	79±4	16	Normal, 10	50	321	Bread, 2h	Standard	Capillary, whole blood	YSI	110
1836	Potato, novel clone, selection 3 (F03031, grown to maturity in Ontario), boiled 15 min, reheated in microwave 2-3 min	Canada	2009-2010	73±6	15	Normal, 10	50	321	Bread, 2h	Standard	Capillary, whole blood	YSI	110
1837	Potato, novel clone, selection 4 (F03028, grown in New Brunswick), unpeeled, cut into 2.5-3 cm cubes, boiled in salted water for 15 min	Canada	2009	72±8	14	Normal, 10	50	394	Bread, 2h	Standard	Capillary, whole blood	YSI	110
1838	Potato, novel clone, selection 4 (F03028, grown in New Brunswick), boiled 15 min, reheated in microwave 2-3 min	Canada	2009	58±5	12	Normal, 10	50	394	Bread, 2h	Standard	Capillary, whole blood	YSI	110
	Boiled potato, mean of 29 studies			73									

Food Number	and Item	Country of food production	Year of test[1]	GI[2] ± SEM (Glu = 100)	GL	Subjects (type & number)	Avail carb (Test portion)	Test portion[3] (g)	Reference food & time period	Timepoints	Sample collection	Sample analysis method[4]	Ref.
	**Boiled/cooked, potato, white/NS**												
1839	Irish potato (*Solanum tuberosum*), peeled, cubed, boiled 30 min	Jamaica	2005	59±4	12	Normal, 10	50	231.0	Glucose, 2h	Standard	Capillary, plasma	Enzymatic	88
1840	Irish potato (*Solanum tuberosum*), peeled, boiled 30 min, refrigerated, reheated for 1 min	Canada	2003	71±8	14	Normal, 10	50	151	Bread, 2h	Standard	Capillary, plasma	Enzymatic	116
1841	New potato, unpeeled and boiled 20 min	Australia	1998	78±12		Normal, 10	50	358	Bread, 2h	Standard	Capillary, plasma	Enzymatic	117
1842	Potato, NS, boiled	China	2005	66±1	13	Normal, 10	50	NS	Glucose, 2h	Standard	Venous, NS	NS	12
1843	Red potatoes, boiled with skin on in salted water for 12 min	Canada	2005	89±7	18	Normal, 12	50	352	Bread, 2h	Standard	Capillary, whole blood	YSI	120
1844	Potato, frozen peeled cubes, boiled (McCain Purely Potato Cubes, McCains, Wendouree, VIC, Australia)	Australia	2010	70±4	14	Normal, 10	50	285.7	Glucose, 2h	Standard	Capillary, plasma	Enzymatic	39
	**Boiled potato cooked then cooled**												
1845	Potato, novel clone, selection 1 (CV92056-4, grown to maturity in Alberta; harvested Oct. 2008), boiled for 15 min, refrigerated at 4°C for 24-28 h, served cold	Canada	2009	55±7	11	Normal, 10	50	345	Bread, 2h	Standard	Capillary, whole blood	YSI	110
1846	Potato, novel clone, selection 1 (CV92056-4, grown in Ontario; harvested Oct. 2009), boiled for 15 min, refrigerated at 4°C for 24-28 h, served cold	Canada	2009-2010	48±6	10	Normal, 10	50	345	Bread, 2h	Standard	Capillary, whole blood	YSI	110
1847	Potato, novel clone, selection 2 (CV1102-1, grown to maturity in Alberta, harvested Oct. 2008), boiled for 15 min, refrigerated at 4°C for 24-28 h, served cold	Canada	2009	55±7	11	Normal, 9	50	360	Bread, 2h	Standard	Capillary, whole blood	YSI	110
1848	Potato, novel clone, selection 2 (CV1102-1, grown in Alberta, harvested prior to maturity Oct. 2009), boiled for 15 min, refrigerated at 4°C for 24-28 h, served cold	Canada	2009-2010	41±5	8	Normal, 10	50	360	Bread, 2h	Standard	Capillary, whole blood	YSI	110
1849	Potato, novel clone, selection 3 (F03031, grown in New Brunswick), boiled for 15 min, refrigerated at 4°C for 24-28 h, served cold	Canada	2009	40±5	8	Normal, 10	50	321	Bread, 2h	Standard	Capillary, whole blood	YSI	110
1850	Potato, novel clone, selection 3 (F03031, grown to maturity in Ontario), boiled for 15 min, refrigerated at 4°C for 24-28 h, served cold	Canada	2009-2010	49±7	10	Normal, 10	50	321	Bread, 2h	Standard	Capillary, whole blood	YSI	110

Food Number and Item		Country of food production	Year of test[1]	GI[2] ± SEM (Glu = 100)	GL	Subjects (type & number)	Avail carb (Test portion)	Test portion[3] (g)	Reference food & time period	Timepoints	Sample collection	Sample analysis method[4]	Ref.
1851	Potato, novel clone, selection 4 (F03028, grown in New Brunswick), boiled for 15 min, refrigerated at 4°C for 24-28 h, served cold	Canada	2009	44±7	9	Normal, 10	50	394	Bread, 2h	Standard	Capillary, whole blood	YSI	110
1852	Potato, red, cubed, boiled in salted water 12 min, stored overnight in refrigerator, consumed cold	Canada	2005	56±5	11	Normal, 12	50	352	Bread, 2h	Standard	Capillary, whole blood	YSI	120
	Potato cooked then cooled, mean of eight foods			49									
	**Canned potatoes**												
1853	New potatoes, canned, heated in microwave 3 min (Mint Tiny Taters™, Edgell's™ brand, Cheltenham, Australia)	Australia	1998	65±9	13	Normal, 10	50	416	Bread, 2h	Standard	Capillary, plasma	Enzymatic	117
	**French Fries**												
1854	French Fries, baked 15 min (OreIda Golden Fries, H.J. Heinz Co, Pittsburgh, PA, USA)	USA	2005	64±6	13	Normal, 12	50	233	Bread, 2h	Standard	Capillary, whole blood	YSI	120
1855	French Fries, cooked in oven for 20 min from frozen (McCain Superfries, McCains, Wendouree, VIC, Australia)	Australia	2010	65±4	13	Normal, 10	50	208.3	Glucose, 2h	Standard	Capillary, plasma	Enzymatic	39
	**Instant Mashed potato**												
1856	Instant mashed potato (Idahoan Foods, Lewisville, ID, USA)	USA	2005	88±8	18	Normal, 12	50	67.3	Bread, 2h	Standard	Capillary, whole blood	YSI	120
1857	Instant mashed potato (Idahoan Foods, Lewisville, ID, USA), prepared with water	USA	2001	92±4	18	Normal, 47	50	NS	Glucose, 2h	Standard	Capillary, NS	YSI or Enzymatic	42
1858	Instant mashed potato (Idahoan Foods, Lewisville, ID, USA), prepared with water	USA	2001	69±9	14	Normal, 21	50	NS	Glucose, 2h	Standard	Venous, NS	YSI or Enzymatic	42
1859	Instant mashed potato (Idahoan Foods, Lewisville, ID, USA)	USA	1998	97±6	19	Normal, 10	50	67.3	Glucose, 2h	Standard	Capillary, plasma	Enzymatic	UO[5]
1860	Potato mash (Homestyle, continental, Unilever, Australasia)[13]	New Zealand	2011*	72±6	14	Normal, 30	50		Glucose, 2h	Standard	Capillary, whole blood	HemoCue	69
	Instant mashed potato, mean of five studies			84									
	**Mashed potato**												
1861	Mashed potato, NS	China	2005	73±3	15	Normal, 10	50	NS	Glucose, 2h	Standard	Venous, NS	NS	12
1862	Mashed potato, Aviko B.V.	UK	2009	76±7	15	Normal, 15	25	NS	Glucose, 2h	Standard	Capillary, whole blood	HemoCue	121

1863	Mashed potato, NS	UK	2009	76±7	15	Normal, 15	25	NS	Glucose, 2h	Standard	Capillary, whole blood	HemoCue	121
1864	Potato, Folva, precooked, peeled, salted, vacuum packed and then boiled until soft to the center, drained and mashed (Superior Potet, Hoff SA, Gjøvik, Norway) [11]	Norway	2019*	81±4	16	Normal, 13	50	325.2	Glucose, 2h	Standard	Capillary, plasma	YSI	80
1865	Pontiac potato, peeled, cubed, boiled 15 min, mashed	Australia	1998	91±9	18	Normal, 10	50	414	Bread, 2h	Standard	Capillary, plasma	Enzymatic	117
	Mashed potato, mean of five studies			79									
	**Microwaved potato**												
1866	Estima potato, cooked in microwave 6 min then baked 10 min	UK	2005	93±8	19	Normal, 10	50	290.7	Glucose, 2h	Standard	Capillary, whole blood	Glucometer[9]	49
1867	Pontiac potato, peeled, cooked in microwave on high for 6-7.5 min	Australia	1998	79±9	16	Normal, 10	50	414	Bread, 2h	Standard	Capillary, plasma	Enzymatic	117
1868	Prince Edward Island white potato, cooked in microwave for 18 min	Canada	2005	73±5	15	Normal, 12	50	298	Bread, 2h	Standard	Capillary, whole blood	YSI	120
1869	Russet Burbank Norkotah potato, unpeeled, cooked in microwave for 18 min	USA	2005	77±9	15	Normal, 12	50	376	Bread, 2h	Standard	Capillary, whole blood	YSI	120
	**Potato, other preparation methods**												
1870	California white potatoes, cubed, roasted in soybean oil	USA	2005	72±8	14	Normal, 12	50	376	Bread, 2h	Standard	Capillary, whole blood	YSI	120
1871	Irish potato (*Solanum tuberosum*), peeled, fried in vegetable oil	Jamaica	2005	70±6	14	Normal, 10	50	249.6	Glucose, 2h	Standard	Capillary, plasma	Enzymatic	88
1872	Hash Browns, crispy shredded potato cakes, frozen, baked at °C for 17 min, Birds Eye™ 230	Australia	2012	56±6	11	Normal, 10	50	280.9	Glucose, 2h	Standard	Capillary, plasma	Enzymatic	UO[5]
1873	Potato, NS, steamed	China	2005	62±2	12	Normal, 10	50	NS	Glucose, 2h	Standard	Venous, NS	NS	12
	**Sweet potato/Kumara, baked**												
1874	Sweet potato (*Ipomoea batatas*), Dor cultivar, baked with skin °C for 45 min on at 175	Jamaica	2008	83±6	17	Normal, 10	50	173	Glucose, 2h	Standard	Capillary, plasma	Enzymatic	122
1875	Sweet potato (*Ipomoea batatas*), Clarendon cultivar, baked °C for 45 min with skin on at 175	Jamaica	2008	83±3	17	Normal, 10	50	162	Glucose, 2h	Standard	Capillary, plasma	Enzymatic	122

Food Number and Item		Country of food production	Year of test[1]	GI[2] ± SEM (Glu = 100)	GL	Subjects (type & number)	Avail carb (Test portion)	Test portion[3] (g)	Reference food & time period	Timepoints	Sample collection	Sample analysis method[4]	Ref.
1876	Sweet potato (*Ipomoea batatas*), Eustace cultivar, baked with skin on at 175 ºC for 45 min	Jamaica	2008	93±5	19	Normal, 10	50	182	Glucose, 2h	Standard	Capillary, plasma	Enzymatic	122
1877	Sweet potato (*Ipomoea batatas*), Fire on Land cultivar, baked with skin on at 175 ºC for 45 min	Jamaica	2008	87±4	17	Normal, 10	50	186	Glucose, 2h	Standard	Capillary, plasma	Enzymatic	122
1878	Sweet potato (*Ipomoea batatas*), Ganja cultivar, baked with skin on at 175 ºC for 45 min	Jamaica	2008	82±3	16	Normal, 10	50	162	Glucose, 2h	Standard	Capillary, plasma	Enzymatic	122
1879	Sweet potato (*Ipomoea batatas*), Ms Mac cultivar, baked with skin on at 175 ºC for 45 min	Jamaica	2008	87±4	17	Normal, 10	50	172	Glucose, 2h	Standard	Capillary, plasma	Enzymatic	122
1880	Sweet potato (*Ipomoea batatas*), Minda cultivar, baked with skin on at 175 ºC for 45 min	Jamaica	2008	91±3	18	Normal, 10	50	157	Glucose, 2h	Standard	Capillary, plasma	Enzymatic	122
1881	Sweet potato (*Ipomoea batatas*), Quarter Million cultivar, baked with skin on at 175 ºC for 45 min	Jamaica	2008	94±3	19	Normal, 10	50	183	Glucose, 2h	Standard	Capillary, plasma	Enzymatic	122
1882	Sweet potato (*Ipomoea batatas*), Watson cultivar, baked with skin on at 175 ºC for 45 min	Jamaica	2008	85±2	17	Normal, 10	50	173	Glucose, 2h	Standard	Capillary, plasma	Enzymatic	122
1883	Sweet potato (*Ipomoea batatas*), Yellow Belly cultivar, baked with skin on at 175 ºC for 45 min	Jamaica	2008	86±2	17	Normal, 10	50	171	Glucose, 2h	Standard	Capillary, plasma	Enzymatic	122
1884	Sweet potato (*Ipomoea batatas*), NS, peeled, baked 45 min	Jamaica	2005	94±8	19	Normal, 10	50	167.8	Glucose, 2h	Standard	Capillary, plasma	Enzymatic	122
	Roasted sweet potato, mean of 11 studies			88									
	**Sweet potato/Kumara, boiled**												
1885	Sweet potato (*Ipomoea batatas*), Dor cultivar, peeled, cut into 25 mm slices, boiled in water for 20 min	Jamaica	2008	47±3	9	Normal, 10	50	235	Glucose, 2h	Standard	Capillary, plasma	Enzymatic	122

Food Number and Item	Country of food production	Year of test[1]	GI[2] ± SEM (Glu = 100)	GL	Subjects (type & number)	Avail carb (Test portion)	Test portion[3] (g)	Reference food & time period	Timepoints	Sample collection	Sample analysis method[4]	Ref.
1886 Sweet potato (*Ipomoea batatas*), Clarendon cultivar, peeled, cut into 25 mm slices, boiled in water for 20 min	Jamaica	2008	46±5	9	Normal, 10	50	218	Glucose, 2h	Standard	Capillary, plasma	Enzymatic	122
1887 Sweet potato (*Ipomoea batatas*), Eustace cultivar, peeled, cut into 25 mm slices, boiled in water for 20 min	Jamaica	2008	49±5	10	Normal, 10	50	272	Glucose, 2h	Standard	Capillary, plasma	Enzymatic	122
1888 Sweet potato (*Ipomoea batatas*), Fire on Land cultivar, peeled, cut into 25 mm slices, boiled in water for 20 min	Jamaica	2008	46±4	9	Normal, 10	50	244	Glucose, 2h	Standard	Capillary, plasma	Enzymatic	122
1889 Sweet potato (*Ipomoea batatas*), Ganja cultivar, peeled, cut into 25 mm slices, boiled in water for 20 min	Jamaica	2008	41±5	8	Normal, 10	50	232	Glucose, 2h	Standard	Capillary, plasma	Enzymatic	122
1890 Sweet potato (*Ipomoea batatas*), Ms Mac cultivar, peeled, cut into 25 mm slices, boiled in water for 20 min	Jamaica	2008	45±3	9	Normal, 10	50	252	Glucose, 2h	Standard	Capillary, plasma	Enzymatic	122
1891 Sweet potato (*Ipomoea batatas*), Minda cultivar, peeled, cut into 25 mm slices, boiled in water for 20 min	Jamaica	2008	49±4	10	Normal, 10	50	233	Glucose, 2h	Standard	Capillary, plasma	Enzymatic	122
1892 Sweet potato (*Ipomoea batatas*), Quarter Million cultivar, peeled, cut into 25 mm slices, boiled in water for 20 min	Jamaica	2008	49±4	10	Normal, 10	50	252	Glucose, 2h	Standard	Capillary, plasma	Enzymatic	122
1893 Sweet potato (*Ipomoea batatas*), Watson cultivar, peeled, cut into 25 mm slices, boiled in water for 20 min	Jamaica	2008	43±4	9	Normal, 10	50	263	Glucose, 2h	Standard	Capillary, plasma	Enzymatic	122
1894 Sweet potato (*Ipomoea batatas*), Yellow Belly cultivar, peeled, cut into 25 mm slices, boiled in water for 20 min	Jamaica	2008	50±3	10	Normal, 10	50	346	Glucose, 2h	Standard	Capillary, plasma	Enzymatic	122
1895 Sweet potato (*Ipomoea batatas*), NS, peeled, cubed, boiled 30 min	Jamaica	2005	46±5	9	Normal, 10	50	234.6	Glucose, 2h	Standard	Capillary, plasma	Enzymatic	122
1896 Sweet potato, orange flesh, NS, peeled, cut into pieces, boiled for 8 min	Australia	2006	61±6	12	Normal, 10	50	427.4	Glucose, 2h	Standard	Capillary, plasma	Enzymatic	UO[5]
1897 Sweet potato, purple skin white flesh, peeled, cut into pieces, boiled for 8 min	Australia	2006	75±5	15	Normal, 9	50	255.1	Glucose, 2h	Standard	Capillary, plasma	Enzymatic	UO[5]
Boiled sweet potato, mean of 13 studies			46									
**Sweet potato/Kumara, cooked**												
1898 Red sweet potato (*Ipomoea batatas*), NS, cooking method[13] NS	New Zealand	2011*	84±6	17	Normal, 30	50	NS	Glucose, 2h	Standard	Capillary, whole blood	HemoCue	69

Food Number and Item		Country of food production	Year of test[1]	GI[2] ± SEM (Glu = 100)	GL	Subjects (type & number)	Avail carb (Test portion)	Test portion[3] (g)	Reference food & time period	Timepoints	Sample collection	Sample analysis method[4]	Ref.
1899	Sweet potato, kumara, cooking method NS	New Zealand	2000	77±12	15	Normal, 9	50	NS	Glucose, 2h	Standard	Venous, plasma	Enzymatic	27
	**Sweet potato, fried**												
1900	Sweet potato (*Ipomoea batatas*), Dor cultivar, peeled, cut into 10 mm wedges, fried in pre-heated vegetable oil	Jamaica	2008	76±4	15	Normal, 10	50	173	Glucose, 2h	Standard	Capillary, plasma	Enzymatic	122
1901	Sweet potato (*Ipomoea batatas*), Clarendon cultivar, peeled, cut into 10 mm wedges, fried in pre-heated vegetable oil	Jamaica	2008	73±3	15	Normal, 10	50	162	Glucose, 2h	Standard	Capillary, plasma	Enzymatic	122
1902	Sweet potato (*Ipomoea batatas*), Eustace cultivar, peeled, cut into 10 mm wedges, fried in pre-heated vegetable oil	Jamaica	2008	77±4	15	Normal, 10	50	182	Glucose, 2h	Standard	Capillary, plasma	Enzymatic	122
1903	Sweet potato (*Ipomoea batatas*), Fire on Land cultivar, peeled, cut into 10 mm wedges, fried in pre-heated vegetable oil	Jamaica	2008	75±3	15	Normal, 10	50	186	Glucose, 2h	Standard	Capillary, plasma	Enzymatic	122
1904	Sweet potato (*Ipomoea batatas*), Ganja cultivar, peeled, cut into 10 mm wedges, fried in pre-heated vegetable oil	Jamaica	2008	69±3	14	Normal, 10	50	162	Glucose, 2h	Standard	Capillary, plasma	Enzymatic	122
1905	Sweet potato (*Ipomoea batatas*), Ms Mac cultivar, peeled, cut into 10 mm wedges, fried in pre-heated vegetable oil	Jamaica	2008	63±2	13	Normal, 10	50	172	Glucose, 2h	Standard	Capillary, plasma	Enzymatic	122
1906	Sweet potato (*Ipomoea batatas*), Minda cultivar, peeled, cut into 10 mm wedges, fried in pre-heated vegetable oil	Jamaica	2008	68±3	14	Normal, 10	50	157	Glucose, 2h	Standard	Capillary, plasma	Enzymatic	122
1907	Sweet potato (*Ipomoea batatas*), Quarter Million cultivar, peeled, cut into 10 mm wedges, fried in pre-heated vegetable oil	Jamaica	2008	70±6	14	Normal, 10	50	183	Glucose, 2h	Standard	Capillary, plasma	Enzymatic	122
1908	Sweet potato (*Ipomoea batatas*), Watson cultivar, peeled, cut into 10 mm wedges, fried in pre-heated vegetable oil	Jamaica	2008	67±4	13	Normal, 10	50	173	Glucose, 2h	Standard	Capillary, plasma	Enzymatic	122
1909	Sweet potato (*Ipomoea batatas*), Yellow Belly cultivar, peeled, cut into 10 mm wedges, fried in pre-heated vegetable oil	Jamaica	2008	72±4	14	Normal, 10	50	171	Glucose, 2h	Standard	Capillary, plasma	Enzymatic	122
1910	Sweet potato (*Ipomoea batatas*), NS, peeled, fried in vegetable oil	Jamaica	2005	76±7	15	Normal, 10	50	167.8	Glucose, 2h	Standard	Capillary, plasma	Enzymatic	88
	Fried sweet potato, mean of 11 studies			71									

Food Number and Item	Country of food production	Year of test[1]	GI[2] ± SEM (Glu = 100)	GL	Subjects (type & number)	Avail carb (Test portion)	Test portion[3] (g)	Reference food & time period	Timepoints	Sample collection	Sample analysis method[4]	Ref.
**Sweet potato/Kumara, roasted**												
1911 Sweet potato (*Ipomoea batatas*), Dor cultivar, skin on, roasted on pre-heated charcoal for 45 min	Jamaica	2008	86±4	17	Normal, 10	50	173	Glucose, 2h	Standard	Capillary, plasma	Enzymatic	122
1912 Sweet potato (*Ipomoea batatas*), Clarendon cultivar, skin on, roasted on pre-heated charcoal for 45 min	Jamaica	2008	81±4	16	Normal, 10	50	162	Glucose, 2h	Standard	Capillary, plasma	Enzymatic	122
1913 Sweet potato (*Ipomoea batatas*), Eustace cultivar, skin on, roasted on pre-heated charcoal for 45 min	Jamaica	2008	93±2	19	Normal, 10	50	182	Glucose, 2h	Standard	Capillary, plasma	Enzymatic	122
1914 Sweet potato (*Ipomoea batatas*), Fire on Land cultivar, skin on, roasted on pre-heated charcoal for 45 min	Jamaica	2008	90±3	18	Normal, 10	50	186	Glucose, 2h	Standard	Capillary, plasma	Enzymatic	122
1915 Sweet potato (*Ipomoea batatas*), Ganja cultivar, skin on, roasted on pre-heated charcoal for 45 min	Jamaica	2008	79±4	16	Normal, 10	50	162	Glucose, 2h	Standard	Capillary, plasma	Enzymatic	122
1916 Sweet potato (*Ipomoea batatas*), Ms Mac cultivar, skin on, roasted on pre-heated charcoal for 45 min	Jamaica	2008	85±4	17	Normal, 10	50	172	Glucose, 2h	Standard	Capillary, plasma	Enzymatic	122
1917 Sweet potato (*Ipomoea batatas*), Minda cultivar, skin on, roasted on pre-heated charcoal for 45 min	Jamaica	2008	89±3	18	Normal, 10	50	157	Glucose, 2h	Standard	Capillary, plasma	Enzymatic	122
1918 Sweet potato (*Ipomoea batatas*), Quarter Million cultivar, skin on, roasted on pre-heated charcoal for 45 min	Jamaica	2008	91±2	18	Normal, 10	50	183	Glucose, 2h	Standard	Capillary, plasma	Enzymatic	122
1919 Sweet potato (*Ipomoea batatas*), Watson cultivar, skin on, roasted on pre-heated charcoal for 45 min	Jamaica	2008	87±2	17	Normal, 10	50	173	Glucose, 2h	Standard	Capillary, plasma	Enzymatic	122
1920 Sweet potato (*Ipomoea batatas*), Yellow Belly cultivar, skin on, roasted on pre-heated charcoal for 45 min	Jamaica	2008	85±2	17	Normal, 10	50	171	Glucose, 2h	Standard	Capillary, plasma	Enzymatic	122
1921 Sweet potato (*Ipomoea batatas*), NS, peeled, roasted on preheated charcoal	Jamaica	2005	82±5	16	Normal, 10	50	167.8	Glucose, 2h	Standard	Capillary, plasma	Enzymatic	88
Roasted sweet potato, mean of 11 studies			86									
1922 Sweet potato lattices, processed sweet potato product, baked °C for 18 min (Simplot Australia Pty Ltd, Australia) at 210	Australia	2012	51±4	10	Normal, 10	50	233.6	Glucose, 2h	Standard	Capillary, plasma	Enzymatic	UO[5]

	**Taro /Dasheen**												
1923	Dasheen (Taro), peeled, cubed, boiled 30 min	Jamaica	2005	72±5	14	Normal, 10	50	279.3	Glucose, 2h	Standard	Capillary, plasma	Enzymatic	88
1924	Dasheen, peeled, boiled 30 min, refrigerated, reheated for 1 min	Canada	2003	77±10	15	Normal, 10	50	131	Bread, 2h	Standard	Capillary, plasma	Enzymatic	116
	**Yam**												
1925	Coco yam (*Xanthosoma spp.*), peeled, cubed, boiled 30 min	Jamaica	2005	61±5	12	Normal, 10	50	482.6	Glucose, 2h	Standard	Capillary, plasma	Enzymatic	88
1926	Lucea Yam *(Dioscorea rotundata)*, peeled, cubed, boiled 30 min	Jamaica	2005	74±7	15	Normal, 10	50	274.4	Glucose, 2h	Standard	Capillary, plasma	Enzymatic	88
1927	Lucea Yam *(Dioscorea rotundata)*, peeled, roasted on preheated charcoal	Jamaica	2005	77±5	15	Normal, 10	50	198.2	Glucose, 2h	Standard	Capillary, plasma	Enzymatic	88
1928	Negro yam *(Dioscorea rotundata)*, peeled, cubed, boiled 30 min	Jamaica	2005	73±4	15	Normal, 10	50	235.1	Glucose, 2h	Standard	Capillary, plasma	Enzymatic	88
1929	Negro yam *(Dioscorea rotundata)*, peeled, roasted on preheated charcoal	Jamaica	2005	73±6	15	Normal, 10	50	194.3	Glucose, 2h	Standard	Capillary, plasma	Enzymatic	88
1930	Round leaf yellow yam *(Dioscorea cayenensis)*, peeled, cubed, boiled 30 min	Jamaica	2005	68±3	14	Normal, 10	50	223.0	Glucose, 2h	Standard	Capillary, plasma	Enzymatic	88
1931	Round leaf yellow yam *(Dioscorea cayenensis)*, peeled, roasted on preheated charcoal	Jamaica	2005	80±7	16	Normal, 10	50	186.4	Glucose, 2h	Standard	Capillary, plasma	Enzymatic	88
1932	Sweet yam *(Dioscorea alata)*, peeled, cubed, boiled 30 min	Jamaica	2005	79±4	16	Normal, 10	50	298.0	Glucose, 2h	Standard	Capillary, plasma	Enzymatic	88
1933	Sweet yam *(Dioscorea alata)*, peeled, roasted on preheated charcoal	Jamaica	2005	82±7	16	Normal, 10	50	192.5	Glucose, 2h	Standard	Capillary, plasma	Enzymatic	88
1934	White yam *(Dioscorea alata)*, peeled, cubed, boiled 30 min	Jamaica	2005	75±6	15	Normal, 10	50	239.0	Glucose, 2h	Standard	Capillary, plasma	Enzymatic	88
1935	White yam *(Dioscorea alata)*, peeled, roasted on preheated charcoal	Jamaica	2005	80±6	16	Normal, 10	50	214.1	Glucose, 2h	Standard	Capillary, plasma	Enzymatic	88
1936	White yam (*Dioscorea alata*), peeled, boiled 30 min, refrigerated, reheated for 1 min	Canada	2003	62±6	12	Normal, 10	50	300	Bread, 2h	Standard	Capillary, plasma	Enzymatic	116
1937	Yam (Chinese sweet potato; *Ipomoea batatas* L. Lam), skin peeled, cut into 5 cm cubes, steamed by rice cooker [22]	Taiwan	2010*	37±0.2	7	Normal, 10	50	70	Bread, 2h	Standard	Capillary, whole blood	Enzymatic	60

Food Number and Item		Country of food production	Year of test[1]	GI[2] ± SEM (Glu = 100)	GL	Subjects (type & number)	Avail carb (Test portion)	Test portion[3] (g)	Reference food & time period	Timepoints	Sample collection	Sample analysis method[4]	Ref.
1938	Yam (*Dioscorea cayenensis)*, round leaf yellow yam, cut into 25 mm chunks, boiled for 20 min, simmered for 10 min	Jamaica	2008*	68±3	14	Normal, 10	50	NS	Glucose, 2h	Standard	Capillary, whole blood	Enzymatic	123
1939	Yam (*Dioscorea alata*), white yam, cut into 25 mm chunks, boiled for 20 min, simmered for 10 min	Jamaica	2008*	75±6	15	Normal, 10	50	NS	Glucose, 2h	Standard	Capillary, whole blood	Enzymatic	123
1940	Yam (*Dioscorea esculenta)*, Chinese yam, cut into 25 mm chunks, boiled for 20 min, simmered for 10 min	Jamaica	2008*	97±10	19	Normal, 10	50	NS	Glucose, 2h	Standard	Capillary, whole blood	Enzymatic	123
1941	Yam (*Dioscorea rotundata)*, Lucea yam, cut into 25 mm chunks, boiled for 20 min, simmered for 10 min	Jamaica	2008*	74±7	15	Normal, 10	50	NS	Glucose, 2h	Standard	Capillary, whole blood	Enzymatic	123
	Yam, mean of 21 studies			68									
1942	Yam, cooked, NS	China	2005	54±2	11	Normal, 10	50	NS	Glucose, 2h	Standard	Venous, NS	NS	12
1943	Yam, peeled, boiled	New Zealand	2000	35±5	7	Normal, 14	50	NS	Glucose, 2h	Standard	Venous, plasma	Enzymatic	27
1944	Yam, steamed	China	2005	51±4	10	Normal, 10	50	NS	Glucose, 2h	Standard	Venous, NS	NS	12
	**Vegetable products**												
1945	Tomato sauce, with extra cheese (Pasta Bake, Dolmio, Mars [13] Foods, New Zealand)	New Zealand	2011*	35±3	4	Normal, 30	25	NS	Glucose, 2h	Standard	Capillary, whole blood	HemoCue	69
1946	Tomato and vegetable pasta sauce, commercially-prepared	France	2007	23±3	2	Normal, 10	50	595.2	Glucose, 2h	Standard	Capillary, plasma	Enzymatic	UO[5]
	**REGIONAL OR TRADITIONAL FOODS**												
	Average carbohydrate portion = 35 g, this value was used to determine the nominal GL for each item in this category.												
	**AFRICAN**												
1947	Aloco, plantain (*Musa* spp. Agnrin) slices (yellow stage of ripeness aag6), salted and fried in vegetable oil	Côte d'Ivoire	2017*	39±0.3	14	Normal, 28	50	99.0	Glucose, 2h	Standard	Capillary, whole blood	Glucometer[9]	115
1948	Aloco, plantain (*Musa* spp. Agnrin) slices (yellow with black spots stage of ripeness aag7), salted and fried in vegetable oil	Côte d'Ivoire	2017*	39±1	14	Normal, 28	50	97.0	Glucose, 2h	Standard	Capillary, whole blood	Glucometer[9]	115
1949	Banane braise (blissi), unripe or half-ripe plantain (*Musa* spp. Afoto), roasted on heated charcoal	Côte d'Ivoire	2017*	88±2	31	Normal, 28	50	122.9	Glucose, 2h	Standard	Capillary, whole blood	Glucometer[9]	115
1950	Klaclo, plantain fritters made from blended over-ripe plantain (*Musa* spp. Ameletiha) mixed with cornflour, fried in vegetable oil	Côte d'Ivoire	2017*	44±0.3	15	Normal, 28	50	101.8	Glucose, 2h	Standard	Capillary, whole blood	Glucometer[9]	115

Food Number and Item	Country of food production	Year of test[1]	GI[2] ± SEM (Glu = 100)	GL	Subjects (type & number)	Avail carb (Test portion)	Test portion[3] (g)	Reference food & time period	Timepoints	Sample collection	Sample analysis method[4]	Ref.
1951 Meat and okra soup, with fermented cassava dough	Nigeria	2015*	75±0.5	26	Normal, 100	50	276.1	Glucose, 2h	Standard	Venous, plasma	Enzymatic	124
1952 Meat, beans and corn pottage with spinach	Nigeria	2015*	33±0.1	12	Normal, 100	50	520.3	Glucose, 2h	Standard	Venous, plasma	Enzymatic	124
1953 Pap and Bambara nut pudding	Nigeria	2015*	40±0.3	14	Normal, 100	50	455.4	Glucose, 2h	Standard	Venous, plasma	Enzymatic	124
1954 Ugali porridge (corn foofoo, pap) made from whole maize flour, cooked for 15 min	India	2019*	71±5	25	Normal, 15	50	63.3	Glucose, 2h	Standard	Capillary, whole blood	HemoCue	79
1955 Ugali porridge (corn foofoo, pap) made from refined maize flour, cooked for 15 min	India	2019*	75±7	26	Normal, 15	50	69.4	Glucose, 2h	Standard	Capillary, whole blood	HemoCue	79
**ARABIC AND TURKISH**												
1956 Arabic bread (baked bread)	UAE	2017*	67±5	23	Normal, 25	50	78.8	Glucose, 2h	Standard	Capillary, whole blood	HemoCue	125
1957 Arseyah (Basmati rice with chicken)	UAE	2017*	72±4	25	Normal, 15	50	507.6	Glucose, 2h	Standard	Capillary, whole blood	HemoCue	125
1958 Balalet (Sweet vermicelli)	UAE	2017*	63±5	22	Normal, 15	50	179.1	Glucose, 2h	Standard	Capillary, whole blood	HemoCue	125
1959 Batheetha (Khalas date paste)	UAE	2017*	59±4	21	Normal, 20	50	130.9	Glucose, 2h	Standard	Capillary, whole blood	HemoCue	125
1960 Biryani, chicken (Basmati rice with chicken)	UAE	2017*	52±4	18	Normal, 15	50	253.9	Glucose, 2h	Standard	Capillary, whole blood	HemoCue	125
1961 Boiled vermicelli (Swayweih/Ballaleet), cooked with sugar, served with Omani coffee (130 mL, 225 mg caffeine)	Oman	2020*	64±5	22	Normal, 12	50	NS	Glucose, 2h	Standard	Capillary, whole blood	Glucometer[9]	6
1962 Boiled vermicelli (Swayweih/Ballaleet), cooked with sugar, served with water	Oman	2020*	67±5	23	Normal, 12	50	NS	Glucose, 2h	Standard	Capillary, whole blood	Glucometer[9]	6
1963 Chami (cottage cheese)	UAE	2017*	60±9	21	Normal, 16	25	472.2	Glucose, 2h	Standard	Capillary, whole blood	HemoCue	125
1964 Chebab bread (Emirati pancake)	UAE	2017*	54±8	19	Normal, 15	50	109.1	Glucose, 2h	Standard	Capillary, whole blood	HemoCue	125
1965 Croissant, served with Omani coffee (130 mL, 225 mg caffeine)	Oman	2020*	65±4	23	Normal, 12	50	NS	Glucose, 2h	Standard	Capillary, whole blood	Glucometer[9]	6

Food Number and Item	Country of food production	Year of test[1]	GI[2] ± SEM (Glu = 100)	GL	Subjects (type & number)	Avail carb (Test portion)	Test portion[3] (g)	Reference food & time period	Timepoints	Sample collection	Sample analysis method[4]	Ref.
1966 Dates, Khalas (Rutab, soft early ripened) (91.1 g), eaten with full-fat plain yoghurt (125 g)	UAE	2002	37±5	13	Normal, 9	50	216.1	Glucose, 2h	NS	Capillary, whole blood	Glucometer[9]	91
1967 Dates, Khalas variety (Al Saad date factory, Al Ain), served with Arabic coffee	UAE	2010	53±6	19	Normal, 13	50	NS	Glucose, 2h	Standard	Capillary, whole blood	Glucometer[9]	126
1968 Doughnut, served with Omani coffee (130 mL, 225 mg caffeine)	Oman	2020*	77±3	27	Normal, 12	50	NS	Glucose, 2h	Standard	Capillary, whole blood	Glucometer[9]	6
1969 Fendal (boiled sweet potato, dates added to cooking water)	UAE	2017*	74±7	26	Normal, 20	50	157.9	Glucose, 2h	Standard	Capillary, whole blood	HemoCue	125
1970 Fried vermicelli (Swayweih/Ballaleet), cooked with sugar, served with Omani coffee (130 mL, 225 mg caffeine)	Oman	2020*	65±5	23	Normal, 12	50	NS	Glucose, 2h	Standard	Capillary, whole blood	Glucometer[9]	6

Food Number and Item	Country of food production	Year of test[1]	GI[2] ± SEM (Glu = 100)	GL	Subjects (type & number)	Avail carb (Test portion)	Test portion[3] (g)	Reference food & time period	Timepoints	Sample collection	Sample analysis method[4]	Ref.
1971 Fried vermicelli (Swayweih/Ballaleet), cooked with sugar, served with water	Oman	2020*	72±4	25	Normal, 12	50	NS	Glucose, 2h	Standard	Capillary, whole blood	Glucometer[9]	6
1972 Habba Hamra (red seed drink, containing evaporated milk)	UAE	2017*	47±3	16	Normal, 15	50	314.1	Glucose, 2h	Standard	Capillary, whole blood	HemoCue	125
1973 Harees, beef (whole wheat served with meat)	UAE	2017*	42±2	15	Normal, 15	50	323.0	Glucose, 2h	Standard	Capillary, whole blood	HemoCue	125
1974 Khabisa (semolina pudding with cardamom)	UAE	2017*	67±4	23	Normal, 15	50	89.0	Glucose, 2h	Standard	Capillary, whole blood	HemoCue	125
1975 Khameer bread (baked bread)	UAE	2017*	47±3	16	Normal, 15	50	91.1	Glucose, 2h	Standard	Capillary, whole blood	HemoCue	125
1976 Khanfaroosh (Doughnut cake)	UAE	2017*	45±3	16	Normal, 15	50	126.3	Glucose, 2h	Standard	Capillary, whole blood	HemoCue	125
1977 Legemat (Doughnut cake)	UAE	2017*	44±4	15	Normal, 15	50	113.1	Glucose, 2h	Standard	Capillary, whole blood	HemoCue	125
1978 Machbous, fish (Basmati rice with fish)	UAE	2017*	60±3	21	Normal, 20	50	277.8	Glucose, 2h	Standard	Capillary, whole blood	HemoCue	125
1979 Muhalla bread (Emirati sweet crepe)	UAE	2017*	77±2	27	Normal, 15	50	73.9	Glucose, 2h	Standard	Capillary, whole blood	HemoCue	125
1980 Regag bread (Thin crispy crepe)	UAE	2017*	76±7	27	Normal, 25	50	112.9	Glucose, 2h	Standard	Capillary, whole blood	HemoCue	125

Food Number and Item		Country of food production	Year of test[1]	GI[2] ± SEM (Glu = 100)	GL	Subjects (type & number)	Avail carb (Test portion)	Test portion[3] (g)	Reference food & time period	Timepoints	Sample collection	Sample analysis method[4]	Ref.
1981	Sambosa vegetable, fried in oil, served with water	Oman	2020*	60±5	21	Normal, 12	50	NS	Glucose, 2h	Standard	Capillary, whole blood	Glucometer[9]	6
1982	Sambosa vegetable, fried in oil, served with Omani coffee (130 mL, 225 mg caffeine)	Oman	2020*	57±4	20	Normal, 12	50	NS	Glucose, 2h	Standard	Capillary, whole blood	Glucometer[9]	6
1983	Thareed, beef (Regag bread served with meat stew)	UAE	2017*	74±3	26	Normal, 15	50	460.0	Glucose, 2h	Standard	Capillary, whole blood	HemoCue	125
	**ASIAN**												
1984	Baked Barbecued Pork Puff, instant (Hoixe Cake Shop, H.K.)	China	2010*	55±8	19	Normal, 15	50	161	Glucose, 2h	Standard	Capillary, whole blood	YSI	127
1985	Barbecue pork bun, instant (Maxim's MX, H.K.)	China	2010*	69±9	24	Normal, 15	50	119	Glucose, 2h	Standard	Capillary, whole blood	YSI	127
1986	Barley Drink (ready-to-drink packet drink made from pearl barley and dried winter melon) (F&N Foods Private Limited, Singapore)	Singapore	2015*	62±6	22	Normal, 11	50	657.9	Glucose, 2h	Standard	Capillary, whole blood	HemoCue	18
	Beehoon (rice vermicelli), cooked for 1.5 min (NTUC 1987 Fairprice, Singapore)	Singapore	2015*	35±3	12	Normal, 11	50	61.9	Glucose, 2h	Standard	Capillary, whole blood	HemoCue	18
1988	Bread, Garden milk bar bun	China	2010*	73±8	26	Normal, 10	50	105	Glucose, 2h	Standard	Capillary, whole blood	HemoCue	28
1989	Bun, white, filled with red bean paste	Singapore	2018*	58±4	20	Normal, 19	50	106	Glucose, 2h	Standard	Capillary, whole blood	HemoCue	57
1990	Bun, steamed, made from wheat flour	China	2016	85±7	30	Normal, 10	25	47.6	Glucose, 2h	Standard	Capillary, whole blood	Enzymatic	UO[18]
1991	Bun, white, steamed Chinese	Malaysia	2018*	58±3	20	Normal, 19	50	88	Glucose, 2h	Standard	Capillary, whole blood	HemoCue	57
1992	Butter rice, warm white rice and 10 g butter[16]	Japan	2003	79±12	28	Normal, 10	50	157	Rice[17], 2h	Standard	Capillary, whole blood	Glucometer[9]	45
1993	Cake crisp	China	2005	59±2	21	Normal, 10	50	NS	Glucose, 2h	Standard	Venous, NS	NS	12
	Cereal Solid Beverage, common type (LVSHOU Health 1994 Indystry Group, Guangzhou, China)	China	2020	53±5	19	Normal, 12	25	62.5	Glucose, 2h	Standard	Capillary, plasma	Enzymatic	UO[18]

	Cereal Solid Beverage, fortified type (LVSHOU Health 1995 Indystry Group, Guangzhou, China)	China	2020	42±4	15	Normal, 12	25	71.4	Glucose, 2h	Standard	Capillary, plasma	Enzymatic	UO[18]
1996	Char Siew Pau, steamed barbecue pork bun (Tanjong Rhu Pau & Confectionary, Singapore)	Singapore	2015*	66±7	23	Normal, 10	50	154.8	Glucose, 2h	Standard	Capillary, whole blood	HemoCue	18
1997	Chee Cheong Fun, rice noodle roll or steamed rice roll, plain served with sweet soya sauce (Hock Seng Food Private Ltd, Singapore)	Singapore	2015*	81±7	28	Normal, 10	50	277.8	Glucose, 2h	Standard	Capillary, whole blood	HemoCue	18
1998	Chinese Carrot Cake, turnip cake, daikon cake or radish cake, pan-fired shredded radish with plain rice flour (Wei Cian Foods Private Ltd, Singapore)	Singapore	2015*	77±8	27	Normal, 10	50	316.5	Glucose, 2h	Standard	Capillary, whole blood	HemoCue	18
1999	Chinese herbal jelly, instant, prepared freshly in the morning (Guang Jian Tang Brand, Kwong Tai Agency Co., Ltd., China)	China	2010*	47±3	16	Normal, 15	50	333	Glucose, 2h	Standard	Capillary, whole blood	YSI	127
2000	Chinese Yam Cake, taro cake or pan-fried shredded yam with plan rice four (Wei Cian Foods Private Limited)	Singapore	2015*	86±11	30	Normal, 10	50	392.2	Glucose, 2h	Standard	Capillary, whole blood	HemoCue	18
2001	Curry Puff, deep-fried pastry dough stuffed with curry, chicken and potatoes (Old Chang Kee (S) Private Limited)	Singapore	2015*	41±4	14	Normal, 11	50	129.6	Glucose, 2h	Standard	Capillary, whole blood	HemoCue	18
2002	Curry rice[16]	Japan	2003	67±8	23	Normal, 10	50	224	Rice[17], 2h	Standard	Capillary, whole blood	Glucometer[9]	45
2003	Curry rice with processed cheese[16]	Japan	2003	55±9	19	Normal, 10	50	255	Rice[17], 2h	Standard	Capillary, whole blood	Glucometer[9]	45
2004	Dumpling, containing meat and shallots	China	2005	28±3	10	Normal, 10	50	NS	Glucose, 2h	Standard	Venous, NS	NS	12
2005	Dumpling, highland buckwheat flour & beef vegetables fillings (Tibet Jingu Agricultural High Tech. Tibet, China)	China	2020	43±4	15	Normal, 12	50	224.1	Glucose, 2h	Standard	Capillary, whole blood	Enzymatic	UO[18]

Food Number and Item		Country of food production	Year of test[1]	GI[2] ± SEM (Glu = 100)	GL	Subjects (type & number)	Avail carb (Test portion)	Test portion[3] (g)	Reference food & time period	Timepoints	Sample collection	Sample analysis method[4]	Ref.
2006	Egg tart, instant, freshly prepared in the morning (Maxim's MX, Hong Kong)	China	2010*	45±3	16	Normal, 15	50	143	Glucose, 2h	Standard	Capillary, whole blood	YSI	127
2007	Fried Fritter, instant (Ocean Empire International Ltd., H.K.)	China	2010*	69±9	24	Normal, 15	50	139	Glucose, 2h	Standard	Capillary, whole blood	YSI	127
2008	Fried Rice in Yangzhou-style, instant	China	2010*	80±6	28	Normal, 15	50	217	Glucose, 2h	Standard	Capillary, whole blood	YSI	127

2009	Fried rice vermicelli in Singapore-style	China	2010*	54±6	19	Normal, 15	50	333	Glucose, 2h	Standard	Capillary, whole blood	YSI	127
2010	Fried rice vermicelli in Singapore-style	China	2010*	58±9	20	Normal, 15	25	167	Glucose, 2h	Standard	Capillary, whole blood	YSI	127
2011	Glutaminous rice flour (Satou Co. Ltd), instant, served warm with roasted ground soybean[16]	Japan	2003	65±4	23	Normal, 9	50	122	Rice[17], 2h	Standard	Capillary, whole blood	Glucometer[9]	45
2012	Glutinous rice, white, cooked in rice cooker (Bangsue Chia Meng Rice Co., Bangkok, Thailand)	Thailand	2000	94±6	33	Normal, 11	50	65.5	Glucose, 2h	Standard	Capillary, plasma	Enzymatic	74
2013	Glutinous rice ball with cut glutinous cake (mochi)[16]	Japan	2003	48±5	17	Normal, 9	50	132	Rice[17], 2h	Standard	Capillary, whole blood	Glucometer[9]	45
2014	Glutinous rice ball (Lee Chun Brand, Lee Chun Food Ltd., H.K.; Boiled	China	2010*	61±10	21	Normal, 15	50	115	Glucose, 2h	Standard	Capillary, whole blood	YSI	127
2015	Green Bean dessert, instant (NISSIN Brand, Nissin Foods Co., LTD., China)	China	2010*	54±6	19	Normal, 15	50	333	Glucose, 2h	Standard	Capillary, whole blood	YSI	127
2016	Hokkien noodles (Fantastic Fresh, Australia)	Australia	2015	58±3	20	Normal, 10	50	135.8	Glucose, 2h	Standard	Capillary, plasma	Enzymatic	UO[5]
2017	Instant cereal, Quaker oatmeal (anchovy flavor), 200 mL boiled water added	China	2010*	67±5	23	Normal, 10	50	75	Glucose, 2h	Standard	Capillary, whole blood	HemoCue	28
2018	Instant cereal, Nestlé Vita (red date flavor, high FE), 180 mL boiled water added	China	2010*	94±12	33	Normal, 10	50	64,6	Glucose, 2h	Standard	Capillary, whole blood	HemoCue	28
2019	Instant cereal, Nestlé Vita (green tea flavor, high Ca), 180 mL boiled water added	China	2010*	108±8	38	Normal, 10	50	64,4	Glucose, 2h	Standard	Capillary, whole blood	HemoCue	28
2020	Kaya Butter Toast (Just Acia Private Limited)	Singapore	2015*	49±4	17	Normal, 11	50	108.7	Glucose, 2h	Standard	Capillary, whole blood	HemoCue	18
2021	Lo Mai Gai (Hock Seng Food Private Limited)	Singapore	2015*	106±12	37	Normal, 12	50	176.6	Glucose, 2h	Standard	Capillary, whole blood	HemoCue	18
2022	Low-protein white rice with dried sea algae[16]	Japan	2003	70±7	25	Normal, 10	50	125	Rice[17], 2h	Standard	Capillary, whole blood	Glucometer[9]	45
2023	"Mai-Lai" Cake, Instant, prepared freshly in the morning	China	2010*	61±8	21	Normal, 15	50	114	Glucose, 2h	Standard	Capillary, whole blood	YSI	127

Food Number and Item		Country of food production	Year of test[1]	GI[2] ± SEM (Glu = 100)	GL	Subjects (type & number)	Avail carb (Test portion)	Test portion[3] (g)	Reference food & time period	Timepoints	Sample collection	Sample analysis method[4]	Ref.
2024	Moon cake, Maxim's Low Sugar Lotus Seed Paste (salted egg yolk) (Maxim's Caterers Limited, Hong Kong)	Hong Kong	2014	30±4	11	Normal, 10	50	96.7	Glucose, 2h	Standard	Capillary, plasma	Enzymatic	UO[5]
2025	Moon cake, Maxim's Low Sugar White Lotus Seed Paste (salted egg yolk) (Maxim's Caterers Limited, Hong Kong)	Hong Kong	2014	29±5	10	Normal, 10	50	102.0	Glucose, 2h	Standard	Capillary, plasma	Enzymatic	UO[5]
2026	Moon cake, Maxim's Low Sugar Lotus Seed Paste (with pine nut) (Maxim's Caterers Limited, Hong Kong)	Hong Kong	2014	40±4	14	Normal, 10	50	91.4	Glucose, 2h	Standard	Capillary, plasma	Enzymatic	UO[5]
2027	Moon cake, Maxim's Low Sugar White Lotus Seed Paste (with pine nut) (Maxim's Caterers Limited, Hong Kong)	Hong Kong	2014	33±3	12	Normal, 10	50	82.1	Glucose, 2h	Standard	Capillary, plasma	Enzymatic	UO[5]
2028	Moon cakes, Instant, prepared freshly in the morning (Kee Wah Bakery Shop, Hong Kong)	China	2010*	56±7	20	Normal, 15	50	80	Glucose, 2h	Standard	Capillary, whole blood	YSI	127
2029	Mung bean noodles (Lungkow bean thread or vermicelli noodles), dried, boiled	China	2000	39±9	14	Normal, 12	50	60.4	Glucose, 2h	Standard	Capillary, plasma	Enzymatic	74
2030	Mung bean noodles, glass or cellophane noodles, boiled (Longkow Company, Taipei, Taiwan)[22]	Taiwan	2010*	20±0.4	7	Normal, 10	50	64	Bread, 2h	Standard	Capillary, whole blood	Enzymatic	60
2031	Nasi Lemak (Old Chang Kee (S) Private Limited)	Singapore	2015*	66±5	23	Normal, 12	50	179.2	Glucose, 2h	Standard	Capillary, whole blood	HemoCue	18
	Noodles, sau tao chicken-flavored Sichuan spicy noodles 2032 (wheat flour), boiled 3 min	China	2010*	65±4	23	Normal, 10	50	75,4	Glucose, 2h	Standard	Capillary, whole blood	HemoCue	28
2033	Noodles, doll fried noodles (wheat flour and tapioca starch), boiled 3 min	China	2010*	88±8	31	Normal, 10	50	105,2	Glucose, 2h	Standard	Capillary, whole blood	HemoCue	28
2034	Noodles, potato, served with meat, NS	China	2005	17±3	6	Normal, 9	50	NS	Glucose, 2h	Standard	Venous, NS	NS	12
2035	Pandan Waffle (waffle batter prepared with pandan leaves, coconut milk, eggs, and oil) (Dilys Creation, Singapore)	Singapore	2015*	46±6	16	Normal, 11	50	148.1	Glucose, 2h	Standard	Capillary, whole blood	HemoCue	18
2036	Pill potato and yam powder	China	2005	35±4	12	Normal, 9	50	NS	Glucose, 2h	Standard	Venous, NS	NS	12
2037	"Pineapple" bun, instant (Hoixe Cake Shop, H.K.)	China	2010*	65±8	23	Normal, 15	50	91	Glucose, 2h	Standard	Capillary, whole blood	YSI	127

Food Number and Item		Country of food production	Year of test[1]	GI[2] ± SEM (Glu = 100)	GL	Subjects (type & number)	Avail carb (Test portion)	Test portion[3] (g)	Reference food & time period	Timepoints	Sample collection	Sample analysis method[4]	Ref.
2038	Pink Rice Cake (teochew rice cake or Png Kueh), steamed rice pastry stuffed with pan-fried glutinous rice (Dilys Creation, Singapore)	Singapore	2015*	97±12	34	Normal, 11	50	184.7	Glucose, 2h	Standard	Capillary, whole blood	HemoCue	18
2039	Plain steamed vermicelli roll, instant	China	2010*	90±8	32	Normal, 15	50	238	Glucose, 2h	Standard	Capillary, whole blood	YSI	127
2040	Potato noodles with meat	China	2005	17±3	6	Normal, 9	50	NS	Glucose, 2h	Standard	Venous, NS	NS	12
2041	Red bean dessert, instant (Nissin Brand, Nissin Foods Co., LTD., China)	China	2010*	75±8	26	Normal, 15	50	263	Glucose, 2h	Standard	Capillary, whole blood	YSI	127
2042	Rice gruel with dried algae (Satou Co Ltd., Japan)[16]	Japan	2003	81±10	28	Normal, 10	50	659	Rice[17], 2h	Standard	Capillary, whole blood	Glucometer[9]	45
2043	Roasted rice ball (Satou Co Ltd., Japan)[16]	Japan	2003	77±6	27	Normal, 9	50	140	Rice[17], 2h	Standard	Capillary, whole blood	Glucometer[9]	45
2044	Salted meat rice dumpling, instant (Ocean Empire International Ltd., H.K.)	China	2010*	69±8	24	Normal, 15	50	200	Glucose, 2h	Standard	Capillary, whole blood	YSI	127
2045	Salted meat rice dumpling, instant (Ocean Empire International Ltd., H.K.)	China	2010*	81±7	28	Normal, 15	25	100	Glucose, 2h	Standard	Capillary, whole blood	YSI	127
2046	Soba noodles, instant, reheated in hot water, served with soup	Japan	2003	46±9	16	Normal, 9	50	185	Rice[17], 2h	Standard	Capillary, whole blood	Glucometer[9]	45
2047	Spring roll, instant	China	2010*	50±5	18	Normal, 15	25	114	Glucose, 2h	Standard	Capillary, whole blood	YSI	127
2048	Stuffed bun, with shallots and meat, steamed	China	2005	39±4	14	Normal, 10	50	NS	Glucose, 2h	Standard	Venous, NS	NS	12
2049	Sushi, brown rice, vegetable and tofu	Australia	2010	45±3	16	Normal, 10	50	239.9	Glucose, 2h	Standard	Capillary, plasma	Enzymatic	UO[5]
2050	Sushi, salmon ('I Love Sushi', Sydney, Australia)	Australia	2001	48±8	17	Normal, 10	25	69.0	Glucose, 2h	Standard	Capillary, plasma	Enzymatic	UO[5]
2051	Steamed glutinous rice roll, instant	China	2010*	89±8	31	Normal, 15	50	109	Glucose, 2h	Standard	Capillary, whole blood	YSI	127

Food Number and Item		Country of food production	Year of test[1]	GI[2] ± SEM (Glu = 100)	GL	Subjects (type & number)	Avail carb (Test portion)	Test portion[3] (g)	Reference food & time period	Timepoints	Sample collection	Sample analysis method[4]	Ref.
2052	Sticky rice wrapped in lotus leaf, instant (Maxim's MX, H.K.)	China	2010*	83±5	29	Normal, 15	50	167	Glucose, 2h	Standard	Capillary, whole blood	YSI	127
2053	Sweet milky bun, instant	China	2010*	67±5	23	Normal, 15	50	114	Glucose, 2h	Standard	Capillary, whole blood	YSI	127
2054	Sweet milky bun, frozen, steamed (AMOY Brand, Amoy Food Ltd., H.K.)	China	2010*	72±8	25	Normal, 15	50	114	Glucose, 2h	Standard	Capillary, whole blood	YSI	127
2055	Taro (*Colocasia esculenta* L. Schott), skin peeled, cut into 5 cm cubes, steamed by rice cooker[22]	Taiwan	2010*	49±0.2	17	Normal, 10	50	62	Bread, 2h	Standard	Capillary, whole blood	Enzymatic	60
2056	Tuna Fish Bun, instant (Maxim's MX, H.K.)	China	2010*	46±4	16	Normal, 15	50	139	Glucose, 2h	Standard	Capillary, whole blood	YSI	127
2057	Udon noodles, instant, with sauce and fried bean curd (Nishin Shokuhin, Japan)[16]	Japan	2003	48±5	17	Normal, 9	50	191	Rice[17], 2h	Standard	Capillary, whole blood	Glucometer[9]	45
2058	White rice (Satou Co. Ltd, Japan), dried sea algae and milk (Snow Brand, Japan), eaten together[16]	Japan	203	48±8	17	Normal, 9	50	219	Rice[17], 2h	Standard	Capillary, whole blood	Glucometer[9]	45
2059	White rice (Satou Co. Ltd, Japan), dried sea algae and milk (Snow Brand, Japan), milk eaten before rice[16]	Japan	203	56±7	20	Normal, 9	50	219	Rice[17], 2h	Standard	Capillary, whole blood	Glucometer[9]	45
2060	White rice (Satou Co. Ltd, Japan), dried sea algae and milk (Snow Brand, Japan), milk eaten after rice[16]	Japan	2003	55±4	19	Normal, 9	50	219	Rice[17], 2h	Standard	Capillary, whole blood	Glucometer[9]	45
	White rice (Satou Co. Ltd, Japan), dried sea algae and milk, mean of three types			56									
2061	White rice (Satou Co. Ltd, Japan) with fermented soybean (natto)[16]	Japan	2003	56±8	20	Normal, 10	50	174	Rice[17], 2h	Standard	Capillary, whole blood	Glucometer[9]	45
2062	White rice (Satou Co. Ltd with instant miso soup (soybean paste soup)[16]	Japan	2003	61±4	21	Normal, 10	50	160	Rice[17], 2h	Standard	Capillary, whole blood	Glucometer[9]	45
2063	White rice (Satou Co. Ltd, Japan) with low-fat milk Kemrakuren, Japan)[16]	Japan	2003	69±10	24	Normal, 9	50	289	Rice[17], 2h	Standard	Capillary, whole blood	Glucometer[9]	45
2064	White rice (Satou Co. Ltd, Japan) and non-sugar yoghurt (Meiji Dairy, Japan), yoghurt eaten before rice[16]	Japan	2003	59±7	21	Normal, 10	50	232	Rice[17], 2h	Standard	Capillary, whole blood	Glucometer[9]	45

2065	White rice (Satou Co. Ltd, Japan) and non-sugar yoghurt (Meiji Dairy, Japan), yoghurt eaten after rice[16]	Japan	2003	58±6	20	Normal, 10	50	232	Rice[17], 2h	Standard	Capillary, whole blood	Glucometer[9]	45
2066	White rice (Satou Co. Ltd, Japan) with pickled vinegar and pickled cucumber, pickled foods eaten before rice[16]	Japan	2003	60±8	21	Normal, 9	50	173	Rice[17], 2h	Standard	Capillary, whole blood	Glucometer[9]	45
2067	White rice (Satou Co. Ltd, Japan) with pickled vinegar and pickled cucumber, consumed together[16]	Japan	2003	61±6	21	Normal, 11	50	173	Rice[17], 2h	Standard	Capillary, whole blood	Glucometer[9]	45
2068	White rice (Satou Co. Ltd, Japan) with roasted ground soybean (Mitake Food Ind. Japan) [16]	Japan	2003	56±4	20	Normal, 9	50	147	Rice[17], 2h	Standard	Capillary, whole blood	Glucometer[9]	45
2069	White rice (Satou Co. Ltd, Japan) with salted dried plum (umeboshi) [16]	Japan	2003	80±13	28	Normal, 10	50	152	Rice[17], 2h	Standard	Capillary, whole blood	Glucometer[9]	45
2070	WoTao, made from corn and wheat	China	2005	65±5	23	Normal, 10	50	NS	Glucose, 2h	Standard	Venous, NS	NS	12
2071	Yoghurt, Yitangping (Beijing Sanyuan Foods, Beijing, China)	China	2020	21±4	7	Normal, 11	25	454.5	Glucose, 2h	Standard	Capillary, plasma	Enzymatic	UO[18]
2072	Youtiao (deep-fried breadstick) (Koufu Private Ltd, Singapore)	Singapore	2015*	55±4	19	Normal, 11	50	109.6	Glucose, 2h	Standard	Capillary, whole blood	HemoCue	18
	**ASIAN INDIAN**												
2073	Basmati rice pilau, with onion and curry powder	UK	2015*	60±6	21	Normal, 11	50	NS	Glucose, 2h	Standard	Capillary, whole blood	HemoCue	68
2074	Bun	Malaysia	2016*	82±5	29	Normal, 10	50	72	Glucose, 2h	Standard	Capillary, plasma	Glucometer[9]	128
2075	Bun with 10% fenugreek	Malaysia	2016*	51±7	18	Normal, 10	50	91.1	Glucose, 2h	Standard	Capillary, plasma	Glucometer[9]	128
	**Chapatti**												
2076	Chapatti, frozen, heated in dry pan for 3 min until browned (Kawan Food Manufacturing, Malaysia)	Malaysia	2019	50±3	18	Normal, 10	50	132.3	Glucose, 2h	Standard	Capillary, plasma	Enzymatic	UO[5]

**Food Number and Item**		**Country of food production**	**Year of test[1]**	**GI[2] ± SEM (Glu = 100)**	**GL**	**Subjects (type & number)**	**Avail carb (Test portion)**	**Test portion[3] (g)**	**Reference food & time period**	**Timepoints**	**Sample collection**	**Sample analysis method[4]**	**Ref.**
2077	Chapatti (made from Chapatti flour (Elephant Atta Medium Chapatti flour, Rank Hovis Ltd., Manchester, UK)	UK	2015*	68±8	24	Normal, 10	50	NS	Glucose, 2h	Standard	Capillary, whole blood	HemoCue	68
2078	Chapatti	UK	2011*	58±6	20	Normal, 10	50	NS	Glucose, 2h	Standard	Capillary, whole blood	HemoCue	129
2079	Chapatti with 4% ß-glucan	UK	2011*	55±5	19	Normal, 10	50	NS	Glucose, 2h	Standard	Capillary, whole blood	HemoCue	129
2080	Chapatti with 8% ß-glucan	UK	2011*	52±10	18	Normal, 10	50	NS	Glucose, 2h	Standard	Capillary, whole blood	HemoCue	129

2081	Chapati, flatbread	Malaysia	2016*	63±4	22	Normal, 10	50	71.4	Glucose, 2h	Standard	Capillary, plasma	Glucometer[9]	128
2082	Chapati, flatbread with 10% fenugreek	Malaysia	2016*	43±5	15	Normal, 10	50	74	Glucose, 2h	Standard	Capillary, plasma	Glucometer[9]	128
2083	Naan bread (True Foods Pty Ltd, VIC, Australia), heated in microwave for 25 sec	Australia	2013	71±5	25	Normal, 10	50	117.9	Glucose, 2h	Standard	Capillary, plasma	Enzymatic	UO[5]
2084	Paratha, frozen, heated in dry pan for 1.5 min until browned (Kawan Food Manufacturing, Malaysia)	Malaysia	2019	53±5	19	Normal, 10	50	125.3	Glucose, 2h	Standard	Capillary, plasma	Enzymatic	UO[5]
2085	Pilaf porridge, whole grain (minimally processed whole grains: oats, long grain brown rice, rye, hard red winter wheat, triticale, buckwheat, barley, sesame seeds), cooked for 30 min	India	2019*	59±5	21	Normal, 15	50	73.5	Glucose, 2h	Standard	Capillary, whole blood	HemoCue	79
2086	Rice dosa	India	2018*	76±5	27	Normal, 19	50	193	Glucose, 2h	Standard	Capillary, whole blood	HemoCue	57
2087	Rice idli (commercially available dry mix)	India	2018*	85±4	30	Normal, 19	50	162	Glucose, 2h	Standard	Capillary, whole blood	HemoCue	57
	**Upma**												
2088	Finger millet, decorticated, with lower degree of polish, in the form of *upma* (savory meal and traditional Indian breakfast choice)	India	2018*	85±8	30	Normal, 16	50	174.2	Glucose, 2h	Standard	Capillary, whole blood	HemoCue	109
2089	Finger millet flakes in the form of upma	India	2018*	82±7	29	Normal, 16	50	217.4	Glucose, 2h	Standard	Capillary, whole blood	HemoCue	109
2090	Finger millet vermicelli in the form of upma	India	2018*	66±6	23	Normal, 16	50	245.1	Glucose, 2h	Standard	Capillary, whole blood	HemoCue	109
2091	Upma	India	2018*	71±6	25	Normal, 19	50	310	Glucose, 2h	Standard	Capillary, whole blood	HemoCue	57

Abbreviations for Supplemental Table S1: GI, Glycemic index; SEM, standard error of mean; GL, Glycemic load; Avail carb, available carbohydrate; Ref., Reference; NS, not specified.

Footnotes for Supplemental Table S1.

1. Year of test is based on testing year as reported in paper if available, manuscript submission date, or paper publication year *.
2. GI values reported on the glucose scale = 100. Values determined using white bread as the reference food were converted using a factor of 0.71.
3. Test portion weight as reported in the original article, may refer to dry or wet weight.
4. Sample analysis method: Recommended analysis methods according to the ISO Standard (26642:2010) are spectrophotometry or electrochemical detection-coupled enzyme systems. Enzymatic refers to either a glucose oxidase or glucose hexokinase assay performed on a spectrophotometric chemistry analyzer or using a benchtop assay. YSI refers to a Model 2300 STAT glucose analyzer (YSI Inc, Yellow Springs, OH, USA). HemoCue refers to HemoCue 201+ analyzer, HemoCue AB (Angelholm, Sweden).
5. Sydney University's Glycemic Index Research Service (Sydney, Australia), unpublished observations.
6. Year of test and participant number provided by the authors of the original paper.
7. INQUIS Clinical Research (formerly GI Labs Ltd.) (Toronto, Canada), unpublished observations.

8. SEM provided by the authors of the original paper.
9. Glucometer that was used in the study in accordance with the ISO Standard (ISO 26642:2010), with a laboratory inter-assay CV <3.6% on standard solutions.
10. International Diabetes Institute (Melbourne, Australia), unpublished observations, 2006-2007.
11. SEM calculated from standard deviation reported in original paper.
12. Information provided by authors of original paper.
13. SEM calculated from 95% confidence interval reported in original paper, using the formula: (upper limit-lower limit)/2xtinv(1-0.95,n-1) (where n = subject number).
14. F Brighenti, unpublished observations.
15. B Spratt (George Weston Foods, Australia), unpublished observation.
16. Test portion calculated from nutritional information reported in the original paper.
17. White rice was used as the reference food for this study. Glucose also tested and had a GI value of 122 compared to the white rice. The reported GI and SEM were multiplied by 0.82 to convert values reported in the original paper to a GI = 100 glucose scale.
18. China National Research Institute of Food and Fermentation Industries GI Lab (Beijing, China), unpublished observations.
19. Participant number provided by researchers.
20. University of Otago (Dunedin, New Zealand), unpublished observations.
21. Original paper used 120 min incremental area under curve (iAUC) data to determined GI value.
22. Test portion size provided by authors of the original paper.
23. V Lang (Danone Vitapole Company, Le Plessis-Robinson, France), unpublished observations, 1996-2000.
24. J Dzieniszewski, J Ciok (National Food and Nutrition Institute, Poland), unpublished observations, 1996-2001.
25. GI value and SEM provided by authors of original paper.
26. C Dalaudier (Nairn’s Oatcakes Ltd, Scotland), unpublished observations, 2007.
